# SARS-COV-2 protein NSP9 promotes cytokine production by targeting TBK1

**DOI:** 10.3389/fimmu.2023.1211816

**Published:** 2023-10-02

**Authors:** Yihua Zhang, Bowen Xin, Yinan Liu, Wenyi Jiang, Wendong Han, Jian Deng, Peihui Wang, Xiaowu Hong, Dapeng Yan

**Affiliations:** ^1^ Department of Immunology, School of Basic Medical Sciences, Shanghai Institute of Infectious Disease and Biosecurity & Shanghai Public Health Clinical Center, Fudan University, Shanghai, China; ^2^ Biosafety Level 3 Laboratory, Fudan University, Shanghai, China; ^3^ Key Laboratory for Experimental Teratology of Ministry of Education and Advanced Medical Research Institute, Cheeloo College of Medicine, Shandong University, Jinan, China

**Keywords:** SARS-CoV-2, type I interferon, cytokine storm, TBK1, antiviral immunity

## Abstract

SARS-COV-2 infection-induced excessive or uncontrolled cytokine storm may cause injury of host tissue or even death. However, the mechanism by which SARS-COV-2 causes the cytokine storm is unknown. Here, we demonstrated that SARS-COV-2 protein NSP9 promoted cytokine production by interacting with and activating TANK-binding kinase-1 (TBK1). With an rVSV-NSP9 virus infection model, we discovered that an NSP9-induced cytokine storm exacerbated tissue damage and death in mice. Mechanistically, NSP9 promoted the K63-linked ubiquitination and phosphorylation of TBK1, which induced the activation and translocation of IRF3, thereby increasing downstream cytokine production. Moreover, the E3 ubiquitin ligase Midline 1 (MID1) facilitated the K48-linked ubiquitination and degradation of NSP9, whereas virus infection inhibited the interaction between MID1 and NSP9, thereby inhibiting NSP9 degradation. Additionally, we identified Lys59 of NSP9 as a critical ubiquitin site involved in the degradation. These findings elucidate a previously unknown mechanism by which a SARS-COV-2 protein promotes cytokine storm and identifies a novel target for COVID-19 treatment.

## Introduction

COVID-19, caused by the severe acute respiratory syndrome coronavirus 2 (SARS-COV-2), is a serious infectious disease that poses a threat to human health and results in significant loss of human life and social problems. It has resulted in widespread global morbidity and mortality, as well as a massive public health crisis since December 2019 (1). Until June 2023, SARS-COV-2 has affected most of the world’s countries, infecting approximately 481.4 million people and killing over 6.1 million. SARS-CoV-2 is a member of the Betacoronaviruses genus, the Orthocoronavirinae subfamily, and the Coronaviridae family, which also includes severe acute respiratory syndrome coronavirus (SARS-CoV) and middle east respiratory syndrome coronavirus (MERS-CoV), and caused severe epidemics in 2002 and 2012, respectively (2). SARS-CoV-2 is the second Sarbecovirus virus originated in bats and capable of infecting humans, the first is SARS-CoV (3). SARS-CoV-2 shares 79% sequence identity with SARS-CoV and 50% sequence identity with MERS-CoV (4). Initial symptoms of COVID-19 include fever, cough, myalgia, and fatigue, similar to those of SARS and MERS. Dyspnea may occur in the later stage of the disease and progress to acute respiratory distress syndrome or multiple organ failure (MOF) (5). Recently, several variant SARS-CoV-2, such as α, β, γ, δ, λ, and o, have added a new epidemic to the world, evading the vaccine’s protective effect and causing increased morbidity. The frequent mutation of SARS-COV-2 significantly complicates virus prevention and vaccine development (6).

Human beings have evolved a robust and complex immune system to protect themselves from various pathogens throughout their evolutionary history. The innate immune system is activated as the first line of defense by recognizing pathogen-associated molecular patterns (PAMPs) via a number of pattern recognition receptors (PRRs), which play the important role in initiate of immune defense and ultimate elimination of pathogens (7). A rapid and well-coordinated immune response may protect the human body from infection, whereas an uncontrolled and overactive immune response may result in tissue damage or death (8, 9). Cytokines are thought to play a critical role in the immune response to viral infection. At the early stage of viral infection, the immune system promotes the production of pro-inflammatory cytokines such as IFN-α, IFN-β, TNF-α, IL-6 and promotes the production of anti-inflammatory cytokines to maintain homeostasis and prevent tissue damage caused by constantly excessive inflammatory cytokines known as a cytokine storm at the late stage of infection. Uncontrolled cytokine storms are potentially fatal immune phenomena characterized by excessive immune cell activation and the production of massive amounts of inflammatory cytokines and chemical mediators (10). The SARS-CoV-2 infection has been reported to trigger a cytokine storm in some infected individuals, including elevated levels of circulating cytokines such as TNF-α, IL-1, IL-6, IL-12, IFN-α, IFN-β, IFN-γ, MCP-1, and IL-8, which induce severe inflammation and massive immune cell infiltration in multiple organs. Cytokine storm is thought to be the primary cause of chronic obstructive pulmonary disease and MOF in COVID-19 patients (11, 12). However, the mechanism by which the cytokine storm develops during SARS-CoV-2 infection remains unknown.

The SARS-COV-2 genome is approximately 29.7 kb in length and contains a short untranslated region in the 5’ and 3’ terminus and encodes four structural proteins (spike (S), envelop (E), membrane (M), and nucleocapsid (N)), seven accessory proteins (ORF3a, ORF3b, ORF6, ORF7a, ORF7b, ORF8, and ORF9b), and 16 nonstructural proteins (NSP1–16). Several studies have demonstrated that structural and accessory proteins can modulate the host response to facilitate infection and pathogenesis, whereas accessory and nonstructural proteins can regulate host inflammation and type I IFN production in various ways (13, 14). For example, ORF9b suppressed type I IFN responses by targeting TOM70 (15), whereas Nsp6, Nsp13, and Nsp15 suppressed the type I IFN pathway by targeting TBK1 (16, 17). Additionally, ORF6, ORF8, N protein, M protein, NSP5, and NSP14 may all inhibit the production of type I IFNs via distinct mechanisms (18–21). At the early stage of SARS-COV-2 infection, the appropriate pro-inflammatory cytokines and type I IFNs inhibit infection, whereas constant production of pro-inflammatory cytokines and restrained production of anti-inflammatory cytokines results in a cytokine storm at the late stage of infection, which can result in the immune response losing control and attacking multiple host organs.

MID1 (midline 1), also known as TRIM18 (tripartite motif 18), is an E3 ubiquitin ligase that catalyzes the RING (Really Interesting New Gene)-containing proteins encoded by the MID1 gene on the X chromosome’s short arm (22). Apart from its critical role in embryonic midline development, MID1 is also involved in neurodegeneration and cancer (23). Furthermore, MID1 is a microtubule-associated protein that plays a role in cell adhesion and migration by regulating microtubule dynamics (24). MID1 plays a critical role in signal transduction in various physiological processes and acts as a strong connector between the mTORC1 and sonic hedgehog (Shh) signaling pathways (25). There is some evidence that MID1 contributes to the development of allergic airway diseases such as asthma by inhibiting NF-κB and p38 activation via PP2A inhibition (26). Several additional studies demonstrate that MID1 regulates the exocytosis and polarization of lytic granules in mouse T cells via microtubule (27). However, the role of MID1 in viral infection has been reported infrequently.

In the present study, we found that the SARS-COV-2 protein NSP9 positively regulated cytokine production by targeting TBK1. Mechanistically, NSP9 promoted the K63-linked ubiquitination and phosphorylation of TBK1. Meanwhile, we discovered that increased NSP9 expression was due to decreased NSP9 degradation mediated by MID1, which resulted in activation of signal transduction and cytokine production.

## Materials and methods

### Ethics statement

All animal related studies were approved by the Institutional Animal Care and Use Committee of School of Basic Medical Sciences, Fudan University (protocol number: 20220125002).

### Reagents and plasmids

The following chemical reagents and antibodies were used in this study: anti-phospho-TBK1 (5483), anti-phospho-IRF3 (29047), anti-phospho-IRF7 (24129), anti-phospho-p65 (3033), anti-phospho-p38 (9215), anti-phospho-Erk1/2 (9101), anti-phospho- SAPK/JNK (4668; all from Cell Signaling Technology); anti-Flag M2 Affinity Gel (A2220), anti-HA (H9658), anti-HA (H6908), and anti-Flag (F7425; all from Sigma-Aldrich); anti-GST (CW0084M) and anti-His (CW0286; both from Cwbio); and Protein G Sepharose 4 Fast Flow (GE Healthcare). Expression constructs for Flag-RIG-I-N, Flag-TBK1, HA-TBK1, HA-IRF3, Flag-IRF3-5D, Flag-MAVS, HA-TRAF3 and HA-Ubs were obtained from Dr. B. Ge (Tongji University, Shanghai, China). Site-directed point mutagenesis of NSP9 was performed using the 2 × Phanta Flash Master Mix (P510-01, Vazyme) according to the manufacturer’s instructions.

### Cells, viruses and mouse strains

L929 and HEK293T cells were maintained in Dulbecco’s Modified Eagle’s Medium (DMEM; Hyclone) supplemented with 10% (v/v) heat-inactivated FBS (Gibco) and 100 U/ml penicillin and streptomycin (Hyclone). Vero cells were cultured in DMEM supplemented with 3% (v/v) heat-inactivated FBS (Gibco). Six-week-old WT mice were used in the experiments. All animal studies were approved by the Institutional Animal Care and Use Committee of Fudan University.

### Virus infection

For *in vitro* virus infection, L929, and HEK293T cells were cultured in DMEM for 12 h and infected with vesicular stomatitis virus (VSV) (MOI = 1) or Sendai virus (SEV) (100 HAU/ml) for the indicated times. The replication-competent recombinant VSVs (rVSV) system contains five plasmids (nucleoprotein N, phosphoprotein P, matrixprotein M, glycoprotein G, and polymerase L), flanked by the bacteriophage T7 promoter, the VSV leader, the hepatitis delta virus ribozyme, and the T7 terminator sequence. We cloned NSP9 of SARS-CoV-2 into a unique linker site (XhoI-NheI), which was between G and the L genes in T7 plasmid to construct T7-NSP9 plasmid. Then, we transfected these plasmids into HEK293T cells to produce rVSV-NSP9 virus. For *in vivo* virus infection, 6-week-old WT mice were injected with rVSV or rVSV-NSP9 (5 × 10^8^ PFU/mouse) for the indicated times.

### Transfection

HEK293T cells were transiently transfected with polyethylenimine (PEI; 23966-2; Polysciences) and L929 cells were transfected with Lipofectamine 3000 (L3000; Invitrogen). Plasmids were mixed together in DMEM for 5mins. PEI or L3000 was three times more than total amount of plasmids and dissolved in DMEM for 5mins. Then, these two solution were well-mixed for 15mins before added into cell culture medium. according to the manufacturer’s instructions.

### Quantitative real-time PCR

Cells were transfected with indicated plasmids for 48h and then infected with the viruses for the indicated times. Total RNA was isolated using RNAiso Plus (9109; Takara) according to the manufacturer’s instructions. Then, 1 µg of RNA was reverse-transcribed using the PrimeScript™ RT Reagent Kit (RR037; Takara) to generate cDNA. A LightCycler (LC480; Roche) and a ChamQ SYBR qPCR Master Mix (Q311-02QPK-212; Vazyme) were used for quantitative real-time PCR analysis. Real-time PCR relative quantification analysis was performed using the ΔΔCt method. Gene expression was normalized to that of GAPDH. The primer sequences used to amplify human and mouse genes are described in **Table S1** (28).

### Immunoprecipitation and western blotting

Cells were transfected with the indicated plasmids. After 48 h, cells were lysed in a lysis buffer (50 mM Tris (pH 7.4), 150 mM NaCl, 1% Triton X-100, and 1 mM EDTA (pH 8.0)) supplemented with a protease inhibitors cocktail (04693159001; Roche) consisting of 1 mM PMSF, 1 mM Na_3_VO_4_, and 1 mM NaF for 30 min on ice. The lysates were centrifuged at 13,200 rpm for 15 min at 4°C to remove the debris. Cell lysates were incubated with anti-Flag M2 Affinity Gel or Protein G Sepharose 4 Fast Flow plus prespecified antibodies at 4°C overnight. The sepharose samples were centrifuged and washed three times with ice-cold PBST buffer (1% Triton X-100 in PBS). Precipitates or cell lysates were boiled in 1× SDS loading buffer at 100°C for 10 min and then analyzed by immunoblotting.

### Native PAGE

L929 cells were transfected with indicated plasmids for 48 h and infected with virus for the indicated times. Cells were harvested using 100 µl ice-cold lysis buffer (50 mM Tris, pH 7.4; 150 mM NaCl; 1% Triton X-100; 1 mM EDTA, pH 8.0; and a protease inhibitor cocktail consisting of 1 mM PMSF, 1 mM Na_3_VO_4_, and 1 mM NaF). After centrifugation at 13,000 × g for 15 min at 4 °C, the supernatants were quantified and diluted with 2 × native PAGE sample buffer (125 mM Tris-HCl, pH 6.8; 30% glycerol; and 0.1% bromophenol blue). Then, 30 µg of protein was applied to a pre-run 7.5% native gel. After electrophoresis, the proteins were transferred onto a nitrocellulose membrane for immunoblotting.

### GST pull-down

His or GST fusion proteins were expressed in BL-21(DE3) (CB105-02; Tiangen Biotech) according to the manufacturer’s instructions. His fusion protein was purified with Ni Sepharose affinity chromatography. GST fusion proteins were lysed by ultrasonication, incubated with GST beads for 4 h at 4°C, and then washed three times with TBST to collect the precipitates. Then, purified His fusion protein incubated with the collected precipitates for 12 h at 4°C, centrifugation and washing, the beads were boiled in 1× SDS loading buffer at 100°C for 10 min and then analyzed by immunoblotting.

### Dual-luciferase reporter assay

HEK293T cells were transiently transfected with pRL-IFN-β–Luc or pRL-ISRE–Luc, pRL-TK, and the indicated plasmids for 24 h. Dual-luciferase reporter assay system (RG028; Beyotime) was used to detect the luciferase activity according to the manufacturer’s instructions.

### Statistical analysis

Data are expressed as mean ± standard error of the mean (SEM). Prism 6 (GraphPad) was used for statistical analyses. The statistical tests conducted in this study are indicated in the figure legends as follows: *P < 0.05; **P < 0.01; ***P < 0.001, two-tailed unpaired Student’s t-test.

## Results

### NSP9 promotes cellular antiviral innate immune response

**Figure 1:**
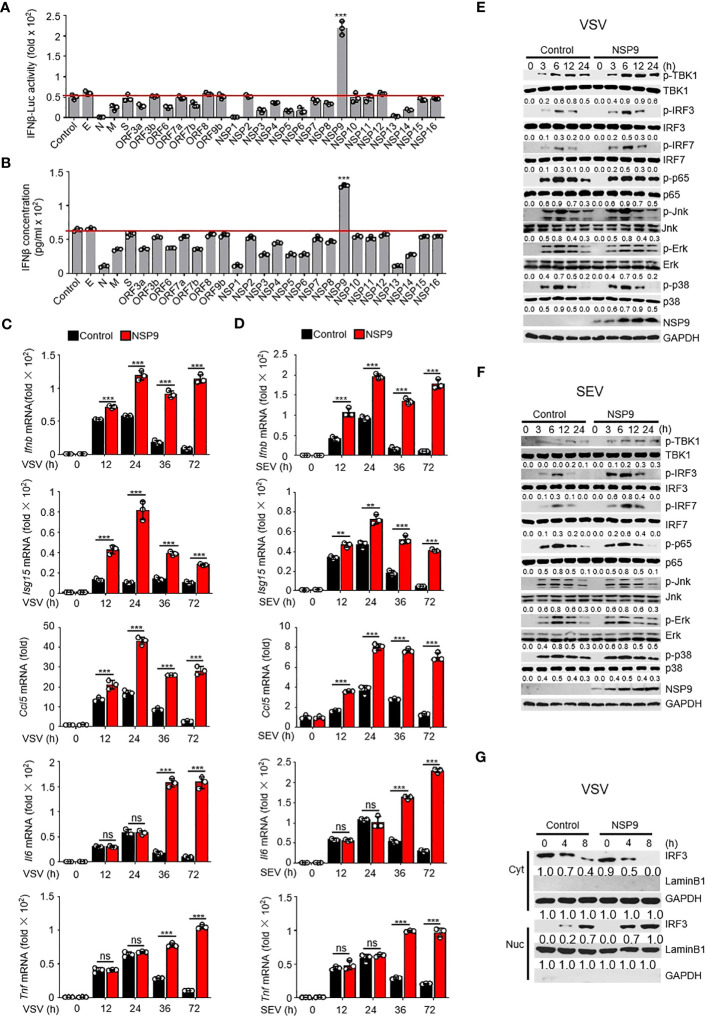
NSP9 promotes cellular antiviral innate immune response. **(A)** Luciferase assay of IFN-β activation in HEK293T cells expressing various vectors and stimulated with VSV for 12 hours. **(B)** ELISA assay of IFN-β expression in L929 cells expressing various vectors and stimulated with VSV for 12 hours. **(C, D)** *Ifnb*, *Isg15*, *Ccl5*, *Tnf* and *Il6* mRNA levels in L929 cells transfected with control or NSP9 vectors and stimulated with VSV **(C)** or SEV **(D)** for indicated times. **(E, F)** Immunoblot of lysates of L929 cells transfected with control or NSP9 vectors and infected with VSV **(E)** or SEV **(F)** for indicated times. **(G)** Immunoblot analysis of nuclear and cytoplasmic fractions in L929 cells transfected with control or NSP9 vectors and stimulated with VSV for indicated times. Data are representative of at least three independent experiments (mean ± SEM in **C**, **D**). ns > 0.05, **P < 0.01, and ***P < 0.001, two-tailed unpaired Student’s *t*-test.

Excessive cytokine storm is one of the main causes of death in COVID-19 patients. We surmised whether any SARS-CoV-2 proteins could induce the persistent production of inflammatory cytokines. Due to the irreplaceable and critical role of IFNs in virus control (29), numerous studies have examined the role of type I IFNs in SARS-CoV-2 infection. In order to determine the effect of SARS-CoV-2 proteins on type I IFN production, we used a dual-luciferase assay to determine which SARS-CoV-2 protein promoted the immune response by monitoring the activity of an IFN-β luciferase reporter. We cloned each SARS-CoV-2 gene into the pcDNA3.0 expression vector and discovered that NSP9 can significantly enhance the activation of IFN-β (**Figure 1A**). To further confirm the function of SARS-CoV-2 proteins in producing the expression of IFN-β, we estimated the production of IFN-β at protein level through ELISA, as well as the *Ifnb* at mRNA level through RT-qPCR (**Figures 1B**, **S1A**). We found that NSP9 significantly produce the expression of IFN-β both in protein level and mRNA level. Next, NSP9 overexpression in L929 cells and stimulation with SEV or VSV were used to verify its role in the antiviral innate immune response. These results indicated that *Ifnb*, *Isg15*, and *Ccl5* levels were significantly increased in NSP9-overexpressed cells, whereas *Il6* and *Tnf* levels remained unchanged at 12 and 24 hours but increased significantly at 36 and 72 hours (**Figures 1C, D**). In response to these atypical findings, we surmised that the *Il6* and *Tnf* levels differed during the early and late stages of infection, respectively. Typically, the type I IFN pathway is activated primarily during viral infection, and our results indicated that antiviral genes induced by the type I IFN pathway were significantly increased by NSP9 throughout the infection. Thus, we hypothesized that the differential expression of *Il6* and *Tnf* during the late stage of infection was not induced directly by NSP9 but rather by other antiviral genes (such as *Ifnb*, *Isg15*, and *Ccl5*) produced via the activated type I IFN pathway.

To test our hypothesis, we examined the activation of the major viral infection pathways, including type I IFN, NF-κB, and MAPK. We observed that infection with VSV and SEV increased TBK1 phosphorylation, IRF3 phosphorylation, and IRF7 phosphorylation in NSP9-overexpressed L929 cells compared to control vector-overexpressed cells (**Figures 1E, F**). However, the phosphorylation of p65, p38, Erk, and Jnk remained unaffected, indicating that NSP9 promotes the type I IFN signaling pathway rather than the NF-κB or MAPK pathways during the early stages of infection. Furthermore, the induction of antiviral genes and activation of the type I IFN pathway may further activate other inflammatory pathways and promote the production of inflammatory cytokines such as *Il6* and *Tnf*, which may explain the difference in *Il6* and *Tnf* expression between early and late stages of infection. Since IRF3 dimerization and translocation are required to activate the type I IFN pathway and the production of IFN-β during virus infection (30), NSP9 overexpression was performed in L929 cells and IRF3 dimerization and translocation were detected following VSV or SEV infection. As expected, IRF3 translocation (**Figures 1G**, **S1B**) and dimerization (**Figures S1C, D**) was significantly increased in NSP9-overexpressed L929 cells. These findings indicated that NSP9 positively regulated the antiviral innate immune response. In order to detect the cytotoxicity of NSP9-overexpression, we examined the survival cells transfected with vector or NSP9 for 3 days and 5 days. Our results indicated that NSP9 overexpression didn’t effect the viability of cells compared with control group (**Figure S1E**).

### NSP9 exacerbated tissue damage and death *in vivo*

**Figure 2:**
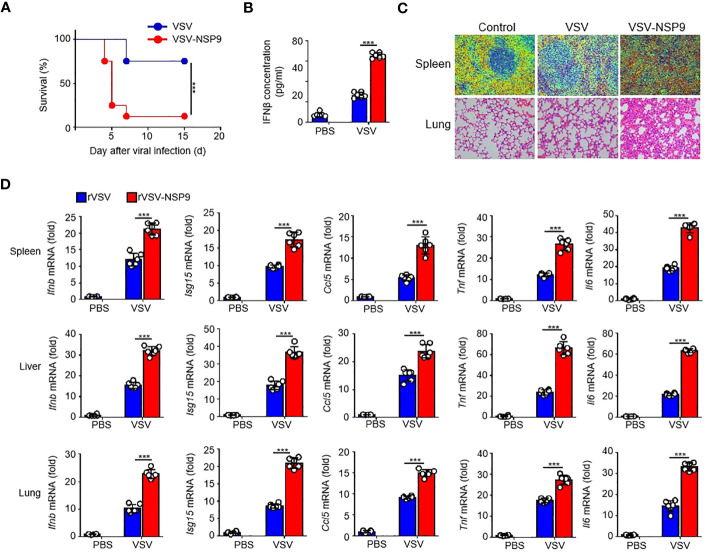
NSP9 aggravated the tissue damage and death *in vivo*. **(A)** Survival of WT (n = 6 per group) infected intraperitoneally with rVSV or rVSV-NSP9 (5 × 10^8^ PFU/mouse) and monitored for 15 days. **(B)** ELISA assay of IFN-β in blood of WT mice (n = 6) intraperitoneally injected with rVSV or rVSV-NSP9 (5 × 10^8^ PFU/mouse) for 4 days. **(C)** Microscopy of hematoxylin-and-eosin-stained lung and spleen sections as in **(A)**. **(D)** *Ifnb*, *Ccl5*, *Isg15, Tnf* and *Il6* mRNA levels in the spleens, livers, and lungs of WT mice (n = 6) intraperitoneally injected with rVSV or rVSV-NSP9 (5 × 10^8^ PFU/mouse) for 4 days. Data are representative of at least three independent experiments (Kaplan–Meier analysis in **A** and mean ± SEM in **D**). ***P < 0.001, two-tailed unpaired Student’s *t*-test.

Next, to investigate NSP9’s *in vivo* function, we constructed an rVSV-NSP9 virus using rVSV system, an rVSV strain containing NSP9 that could infect mice and replicate in cells (31). We infected 6-week-old homozygous mice with rVSV or rVSV-NSP9 (5 × 10^8^ PFU/mouse) and compared their survival rates. We observed that mice infected with rVSV-NSP9 were more susceptible to infection and died at a higher rate than their counterparts (**Figure 2A**). To ascertain the cause of the high mortality observed in the rVSV-NSP9 infection group, we infected mice intraperitoneally for four days and isolated the blood for IFN-β production, livers, spleens, and lungs to assess tissue damage and cytokine production. We found that IFN-β was significantly increased in rVSV-NSP9-infected mice than rVSV-infected mice (**Figure 2B**). Besides, we observed greater injury in the spleens and lungs of mice infected with rVSV-NSP9 than in those infected with rVSV (**Figure 2C**). Additionally, we assessed the expression of *Ifnb*, *Isg15*, *Ccl5, Il6*, and *Tnf* in various tissues and discovered that these cytokines were significantly more abundant in the spleens, livers, and lungs of rVSV-NSP9-infected mice than in those of rVSV-infected mice (**Figure 2D**). These *in vivo* findings suggest that NSP9 promoted the production of pro-inflammatory cytokines and induced a cytokine storm, which ultimately resulted in tissue damage and death in mice.

### NSP9 interacts with TBK1

**Figure 3:**
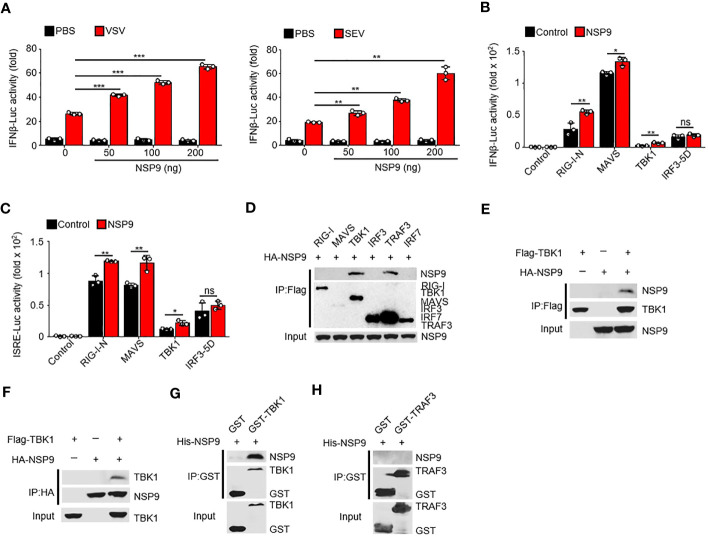
NSP9 interacts with TBK1. **(A)** Luciferase assay of IFN-β activation in HEK293T cells expressing various vectors and stimulated with VSV (left) or SEV (right) for 12 hours. **(B, C)** Luciferase assay of IFN-β **(B)** and ISRE **(C)** activation in HEK293T cells expressing various vectors. **(D–F)** Immunoassay of lysates of HEK293T cells expressing various vectors. **(G, H)** Direct binding of His–NSP9 with GST-TBK1 **(G)** or GST-TRAF3 **(H)**. Data are representative of at least three independent experiments (mean ± SEM in **A**–**C**). ns > 0.05, *P < 0.05, **P < 0.01, and ***P < 0.001, two-tailed unpaired Student’s *t*-test.

Given that NSP9 positively regulates the immune response, it is unknown how NSP9 activates the immune response during infection. To determine the role of NSP9 in IFN-β production, we used a dual-luciferase assay in HEK293T cells and infected with VSV or SEV. We discovered that NSP9 activated the activity of virus-induced IFN-β luciferase reporters in a dose-dependent manner (**Figure 3A**). Next, we sought to elucidate the mechanism by which NSP9 promotes type I IFN signaling. We used the dual-luciferase assay to identify the protein involved in type I IFN signaling that was affected by NSP9 in HEK293T cells in the presence or absence of RIG-I-N, MAVS, TBK1, or constitutively active IRF3 (hereafter referred to as “IRF3-5D”). Our results indicated that overexpression of NSP9 increased the activity of luciferase reporters for IFN-β and ISRE activation induced by the signaling proteins mentioned above, except for IRF3-5D (**Figures 3B, C**). These findings suggested that NSP9 promoted the activation of signaling proteins upstream of IRF3. Subsequently, we co-transfected HA-NSP9 with key type I IFN pathway regulators, including RIG-I, MAVS, TBK1, IRF3, TRAF3, and IRF7, into HEK293T cells. This co-immunoprecipitation (Co-IP) screen established that NSP9 interacted with TBK1 and TRAF3 but not with other types of IFN pathway proteins (**Figure 3D**). Forward and reverse co-immunoprecipitation was used to confirm the interaction between NSP9 and TBK1 (**Figures 3E, F**). We then expressed and purified GST-TBK1, GST-TRAF3, and His-NSP9 in *E. coli* BL21 to determine whether GST-TBK1 or GST-TRAF3 interacted directly with His-NSP9. Using these purified proteins in an *in vitro* GST precipitation assay, we discovered that NSP9 binds directly to TBK1 but not to TRAF3 (**Figures 3G, H**). These findings suggested that NSP9 may exert control over the type I IFN pathway via TBK1.

### NSP9 promotes the activation of TBK1

**Figure 4:**
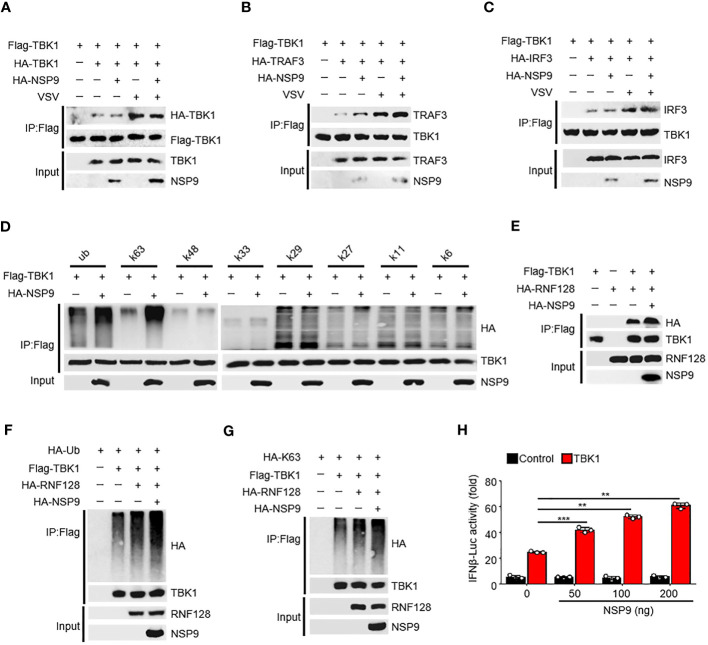
NSP9 promotes the activation of TBK1. **(A–C)** Cell lysates from HEK293T cells transfected with various vectors for 48h and stimulated with VSV for 12 hours, were treated with Flag-beads to IP Flag-TBK1, then immunoassay of IP solution and input as we described in “MATERIALS AND METHODS” section, to detect whether TBK1 dimerization **(A)**, TBK1-TRAF3 interaction **(B)**, or TBK1-IRF3 interaction **(C)** were affected by NSP9 and virus infection. **(D)** Cell lysates from HEK293T cells transfected with various vectors were divided into IP and input part to detect which polyubiquitin chain-linked ubiquitination of TBK1 was influenced by NSP9. **(E)** Immunoassay of cell lysates from HEK293T cells transfected with various vectors and treated as in **A** to detect whether TBK1-RNF128 interaction was affected by NSP9. **(F, G)** Immunoassay of cell lysates from HEK293T cells transfected with various vectors and treated as in **(D)** to detect whether total ubiquitination **(F)** or K63-linked **(G)** of TBK1 was influenced by NSP9. **(H)** Luciferase assay of IFN-β activation in HEK293T cells expressing various vectors. Data are representative of at least three independent experiments (mean ± SEM in **H**). **P < 0.01, and ***P < 0.001, two-tailed unpaired Student’s *t*-test.

The activation of TBK1 is tightly regulated (32). TBK1 typically dimerizes first, followed by ubiquitination by the E3 ubiquitin ligase and phosphorylation at Ser172, which induces IRF3 dimerization and translocation and ultimately promotes IFN-β production (33). Since NSP9 interacts with TBK1, we sought to determine the effect of NSP9 on TBK1 activation. We found that NSP9 had no effect on TBK1 dimerization, although VSV infection induced this dimerization (**Figure 4A**). We then wondered whether NSP9 affected the signaling transduction of TBK1. NSP9 promoted the interaction of TBK1 and TRAF3 (**Figure 4B**) in the presence or absence of VSV infection but had no effect on the interaction of TBK1 and IRF3 (**Figure 4C**). Given that TBK1 is ubiquitinated prior to phosphorylation, we next sought to determine whether NSP9 affected TBK1 ubiquitination. As we know, there are seven distinct polyubiquitin chains, namely K6, K11, K27, K29, K33, K48, and K63. Typically, classical K63-linked ubiquitination is associated with protein activation, whereas K48-linked ubiquitination is associated with proteasomal degradation (34). We examined all of these types of ubiquitination of TBK1 and discovered that NSP9 significantly increased both the K63-linked and total ubiquitination of TBK1 (**Figure 4D**), which is associated with TBK1 activation, implying that NSP9 promoted TBK1 activation by increasing K63-linked ubiquitination. RNF128 has been shown to promote the K63-linked ubiquitination of TBK1 (35). We wondered whether NSP9 facilitates TBK1 activation via RNF128. As a result, we first identified the interaction of RNF128 and TBK1 in the presence and absence of NSP9 and discovered that NSP9 promoted the interaction of RNF128 and TBK1 (**Figure 4E**). Then, we sought to determine whether NSP9 affects the ubiquitination and K63-linked ubiquitination of TBK1 mediated by RNF128. As expected, NSP9 enhanced the ubiquitination and K63-linked ubiquitination of TBK1 regulated by RNF128 (**Figures 4F, G**), implying that NSP9 may promote TBK1 activation via RNF128. Additionally, the luciferase assay demonstrated that NSP9 promoted the activation of the IFN-β promoters triggered by TBK1 in a dose-dependent manner (**Figure 4H**). The above results indicated that NSP9 promoted TBK1 activation by increasing TBK1’s K63-linked ubiquitination.

### MID1 contributed to the degradation of NSP9

**Figure 5:**
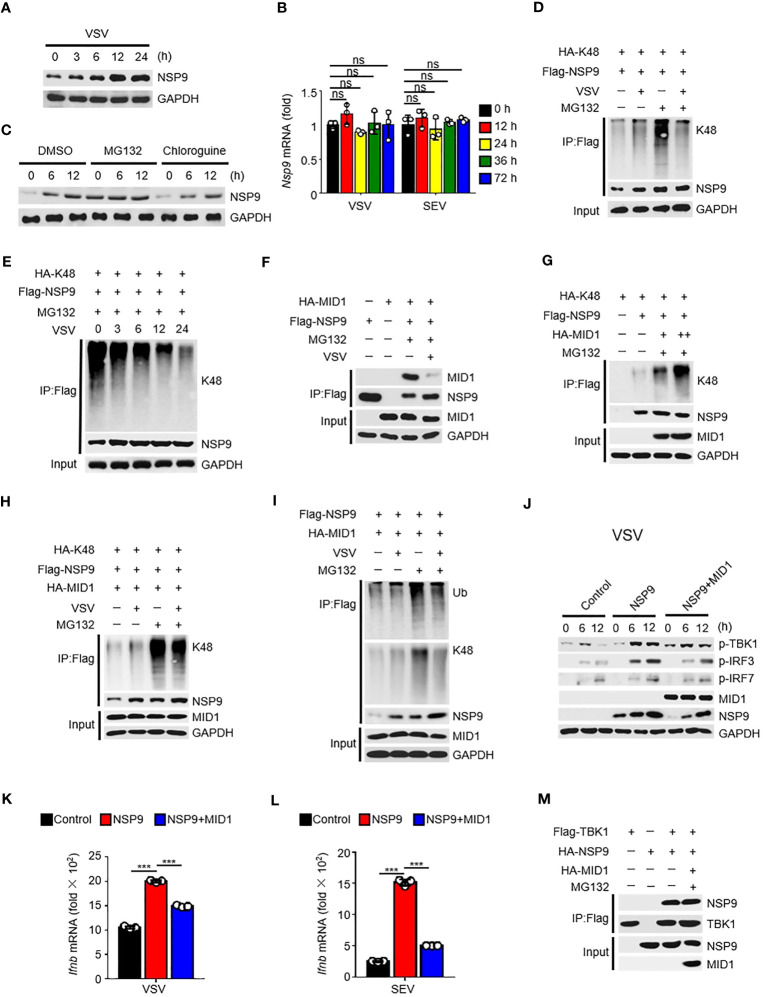
MID1 contributed to the degradation of NSP9. **(A)** Immunoassay of cell lysates from HEK293T cells transfected with NSP9 vector and stimulated with VSV for indicated times. **(B)** *Nsp9* mRNA levels in L929 cells infected with VSV or SEV for indicated times. **(C)** Immunoassay of cell lysates from HEK293T cells transfected with various vectors and infected with VSV for indicated times, then stimulated with DMSO, MG132 or Chloroquine. **(D–I)** Immunoassay of cell lysates from HEK293T cells **(D, F-H)** or L929 cells **(E, I)** transfected with various vectors and infected with VSV for 12 hours, then stimulated with DMSO or MG132. **(J)** Immunoblot of lysates of L929 cells transfected with control or NSP9 vectors and infected with VSV for indicated times. **(K, L)** *Ifnb* mRNA levels in L929 cells transfected with control or NSP9 vectors and stimulated with VSV **(K)** or SEV **(L)** for 36 hours. **(M)** Immunoassay of lysates of HEK293T cells expressing various vectors and stimulated with DMSO or MG132. Data are representative of at least three independent experiments (mean ± SEM in **K**, **L**). ns > 0.05, ***P < 0.001, two-tailed unpaired Student’s *t*-test.

Given that NSP9 expression was increased following viral infection (**Figure 5A**), which resulted in constant activation of the type I IFN pathway and production of cytokines, the first thought that occurred to us was to verify NSP9 transcription during viral infection. Subsequently, we examined the abundance of NSP9 mRNA and discovered that it was unaffected by viral infection (**Figure 5B**). These findings prompted us to consider NSP9 post-translational modification, which might affect the protein level expression of NSP9 (36, 37). We hypothesized that infection-induced NSP9 expression was caused by decreased NSP9 degradation. Since both proteasome and lysosomal degradation are known to reduce protein expression, we detected NSP9 expression during virus infection in cells stimulated with a proteasome degradation inhibitor MG132 or a lysosomal degradation inhibitor chloroquine. We observed that NSP9 expression was increased following stimulation with MG132 rather than chloroquine (**Figure 5C**). As K48-linked ubiquitination is a common post-translational modification that occurs in cells to activate the proteasome degradation pathway (34), we examined ubiquitination of NSP9 during viral infection in HEK293T and L929 cells. We discovered that increased total and K48-linked ubiquitination of NSP9 was associated with increased NSP9 expression following infection (**Figures 5D**, **S2A**). However, when MG132 was used to inhibit NSP9 degradation, the total and K48-linked ubiquitination of NSP9 was significantly decreased following viral infection in different types of cells (**Figures 5D, E**, **S2A, B**).

In order to elucidate the mechanism regulating NSP9’s K48-linked ubiquitination, we used mass spectrometry to analyze NSP9 with or without VSV infection to identify the E3 ubiquitin ligase responsible for NSP9 ubiquitination (**Tables S2**, **S3**). The results indicated that MID1, an E3 ubiquitin ligase, was detected in NSP9-overexpressed group rather than control group, which indicated that MID1 could associate with NSP9. We also performed a co-immunoprecipitation experiment to confirm the interaction between NSP9 and MID1, but co-expression of MID1 significantly decreased NSP9 expression (**Figure S2C**). Therefore, we performed the co-immunoprecipitation assay following treatment with MG132, a compound that inhibits NSP9 degradation. Notably, we observed that NSP9 interacted with MID1 but that this interaction was diminished following virus infection (**Figure 5F**). Next, we examined the effect of MID1 on the ubiquitination of NSP9 following treatment with MG132. As expected, MID1 promoted the K48-linked and total ubiquitination of NSP9 in a dose-dependent manner in the presence of MG132 (**Figures 5G**, **S2D**), but VSV infection inhibited this ubiquitination in HEK293T cells (**Figures 5H**, **S2E**). Similarly, when NSP9 degradation was suppressed by MG132 in L929 cells, VSV infection inhibited MID1-promoted total and K48-linked ubiquitination of NSP9 (**Figure 5I**). These findings indicated that MID1 interacted with NSP9 and induced its K48-linked ubiquitination, resulting in NSP9 degradation. However, virus infection reduced the interaction between NSP9 and MID1, effectively inhibiting K48-linked ubiquitination and degradation of NSP9.

Next, we confirmed that MID1 affects NSP9 function during virus infection. Our findings indicated that overexpression of MID1 decreased NSP9 expression and phosphorylation of TBK1, IRF3, and IRF7 during VSV or SEV infection (**Figures 5J**, **S2F**). Additionally, overexpression of MID1 in L929 cells produced less *Ifnb, Isg15*, *Ccl5*, *Il6*, and *Tnf* (**Figures 5K, L**, **S2G, H**). However, MID1 had no effect on the interaction of NSP9 and TBK1 (**Figure 5M**), indicating that MID1’s inhibition of the type I IFN pathway was due to NSP9 degradation.

### Lys59 is responsible for NSP9 degradation

**Figure 6:**
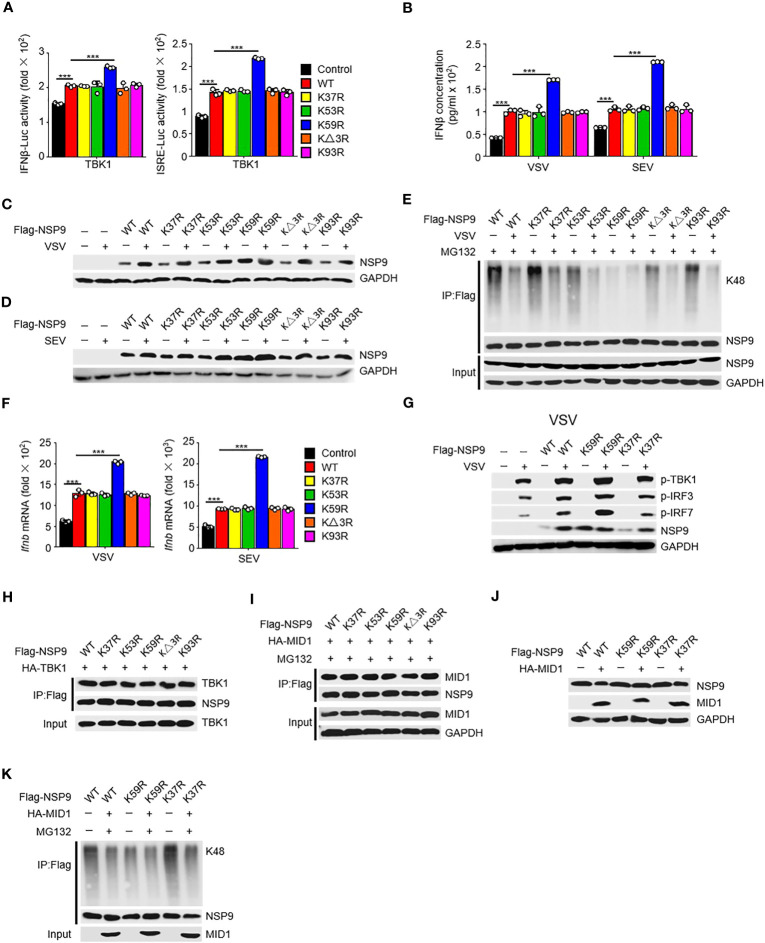
Lys59 is responsible for NSP9 degradation. **(A)** Luciferase assay of IFN-β (left) and ISRE (right) activation in HEK293T cells transfected with various vectors. **(B)** ELISA assay of IFN-β expression in L929 cells transfected with various vectors and stimulated with VSV or SEV for 12 hours. **(C, D)** Immunoassay of cell lysates from L929 cells transfected with various vectors and infected with VSV **(C)** or SEV **(D)** for 12 hours. **(E)** Immunoassay of cell lysates from HEK293T cells transfected with various vectors and infected with VSV for 12 hours, then stimulated with MG132. **(F)** *Ifnb* mRNA levels in L929 cells transfected with control or NSP9 vectors and stimulated with VSV (left) or SEV (right) for 36 hours. **(G)** Immunoblot of lysates of L929 cells transfected with control or NSP9 vectors and infected with VSV for 12 hours. **(H)** Immunoassay of cell lysates from HEK293T cells transfected with various vectors. **(I)** Immunoassay of cell lysates from HEK293T cells transfected with various vectors and stimulated with DMSO or MG132. **(J)** Immunoassay of cell lysates from L929 cells transfected with various vectors. **(K)** Immunoassay of cell lysates from HEK293T cells transfected with various vectors and stimulated with DMSO or MG132. Data are representative of at least three independent experiments (mean ± SEM in **A**, **F**). ***P < 0.001, two-tailed unpaired Student’s *t*-test.

To further investigate the mechanism of NSP9 degradation, we aimed to find the specific site at NSP9 which participated in the ubiquitination of NSP9. Given that ubiquitination typically occurs at lysine residues, we next want to determine which lysine residues in NSP9 are required for K48-linked ubiquitination and degradation. We substituted arginine for each lysine in NSP9 and generated five mutants: K37R, K53R, K59R, KΔ3R (replaced Lys82, Lys85, and Lys87), and K93R. First, we wondered whether these lysines effect the function of NSP9 in activation of type I IFN pathway. We overexpressed WT and mutant NSP9 in HEK293T cells and examined the effect of NSP9 mutants on type I IFN signaling using a TBK1-triggered IFN-β and ISRE luciferase reporter system. Our results indicated that K59R enhanced the activation of IFN-β and ISRE luciferase activity, whereas the other mutants did not differ significantly from WT NSP9 (**Figure 6A**). To further confirm the influence of NSP9 mutants on the production of IFN-β, we tested the expression of IFN-β through ELISA assay and found that K59R but not other NSP9 mutants promoted the production of IFN-β (**Figure 6B**).

We then examined NSP9 mutants’ expression and ubiquitination during viral infection. We discovered that substituting arginine for Lys59 significantly enhanced the expression of NSP9, which was unaffected by infection with VSV (**Figures 6C, D**). Consistently, K59R significantly decreased K48-linked and total ubiquitination activity, which was unaffected by infection with VSV (**Figures 6E**, **S3F**). These findings suggested that Lys59 might be a critical ubiquitination site for NSP9, resulting in its degradation. As a result, we set out to determine the role of K59R in the antiviral immune response. We overexpressed K59R in L929 cells along with WT NSP9 and K37R as controls and discovered that K59R further enhanced the production of *Ifnb, Isg15, Ccl5, Il6*, and *Tnf* (**Figures 6F**, **S3B, C**), as well as the phosphorylation of TBK1, IRF3, and IRF7 (**Figure 6G**) during VSV or SEV infection. Mechanically, we observed that NSP9 mutants had the same ability to interact with TBK1 as WT NSP9, implying that the interaction between NSP9 and TBK1 was independent of NSP9 Lys59 (**Figure 6H**). These findings suggested that Lys59 might be a critical ubiquitination site for NSP9, preventing its degradation and thus enhancing type I IFN pathway activation and cytokine production.

Given that MID1 interacted with NSP9 and induced its K48-linked ubiquitination and degradation, we wondered whether MID1 modified NSP9’s Lys59. We first determined whether MID1 and NSP9 mutants still interacted and discovered that the mutants had no effect on the interaction (**Figure 6I**). Following that, we examined whether MID1 affected the degradation and ubiquitination of NSP9 mutants. We discovered that MID1 promoted the degradation and ubiquitination of WT NSP9 and K37R but not K59R (**Figures 6J, K**, **S3D, E**), implying that MID1 influenced NSP9 ubiquitination via Lys59. Altogether, these results indicated that MID1 was involved in the K48-linked ubiquitination of NSP9 at Lys59, which resulted in NSP9 degradation.

## Discussion

Since December 2019, SARS-CoV-2 has become a global public health crisis, causing millions of deaths and maintaining high infectivity. SARS-CoV-2 mutants have spread a new global epidemic recently, posing a greater threat to public health (38). Adaptive mutations in the SARS-CoV-2 genome not only alter its pathogenic potential, but also increase the difficulty for drug and vaccine development (39). The various mutations retain some of the original strain’s characteristics, such as fever and cough (40). Meanwhile, the primary cause of death in patients infected with various mutations is an excessively produced cytokine storm, highlighting the critical need for further research into the pathogenic mechanism underlying the development of cytokine storm (41). Our findings demonstrated that NSP9 could continuously activate the type I IFN pathway and promote the production of IFNs, thereby activating additional inflammatory signaling pathways and cytokine production, such as IL-6 and TNF-α. Moreover, we established an rVSV-NSP9 virus system that retained the virus’s replication ability and was capable of expressing NSP9 *in vivo*. We observed a significant increase in cytokine production in the rVSV-NSP9 group compared to the rVSV group, along with increased tissue damage and mortality, indicating that these excessive inflammatory cytokines might attack normal tissue and cause death in mice. Thus, we assumed that the deaths were caused by the cytokine storm induced by NSP9.

To elucidate how NSP9 regulated the antiviral immune response, we detected the activation of three major signaling pathways during virus infection, including the type I IFN pathway, the NF-κB, and the MAPK pathways, and discovered that NSP9 promoted the activation of the type I IFN pathway but had no effect on the activation of the other two pathways. To elucidate the precise mechanism by which NSP9 activated the type I IFN pathway, we examined the interaction of NSP9 with several proteins involved in the type I IFN pathway’s transduction and discovered that NSP9 could directly bind to TBK1. Additionally, we observed that NSP9 facilitated TBK1 phosphorylation by promoting its K63-linked ubiquitination, thereby expediting TBK1 activation and subsequent phosphorylation of IRF3 (42). While RNF128 was previously described as an E3 ubiquitination ligase capable of promoting the ubiquitination of TBK1 via the K63-linked ubiquitination, our results indicated that NSP9 could facilitate the interaction of RNF128 and TBK1, as well as the ubiquitination of TBK1. However, the critical residues in TBK1 that were influenced by NSP9 remain unknown.

The conflict between virus and host is considerably more complicated (43–45). In our study, we discovered that MID1 ubiquitinated and degraded NSP9 via Lys59, through K48-linked ubiquitination (46). However, virus infection reduced the interaction between NSP9 and MID1, which could not degrade NSP9 but promoted its expression. Thus, NSP9 could normally facilitate the activation of IFN-β signaling and the antiviral immune response during the early stages of infection. Due to the high level of expression of NSP9, it was able to continuously promote the activation of the type I IFN pathway following infection, which induced the other inflammatory signaling pathways at the late stage of infection. We hypothesize that this degradation was a strategy of immune defense developed during the co-evolution of hosts and viruses to rapidly eliminate viruses (47). Conversely, virus infection-induced inhibition of NSP9 degradation was a clever strategy for viruses to better replication. NSP9 induced cytokine storm, which included a robust production of cytokines and attacked the host cells, could induce the cell death and tissue damage (12). These findings indicated that the host might be able to eliminate NSP9 via MID1, while the interaction between NSP9 and MID1 was weakened following infection, demonstrating how viruses and hosts fight each other via accurate post-modification (48–50). Additionally, we hypothesized that Lys59 is a critical residue involved in the ubiquitination and degradation of NSP9 based on the results of mass spectrometry analysis (data not shown). Compared to WT NSP9, the ubiquitination-defective mutant of NSP9 (K59R) enhanced IFN-β signaling activation. Thus, ubiquitination of NSP9 at Lys59 might be critical in mediating NSP9 degradation. However, our analyses were conducted by overexpressing the protein and using replication competent recombinant VSVs (rVSVs) system to detect the *in vivo* function of NSP9, which was different from real SARS-CoV-2 infection and this is a limitation of our research study.

Besides, there were several studies reported that NSP9 was shown to be essential for virus replication through its ability to bind RNA and could bind to the 7SL RNA in the signal recognition particle and interfere with protein trafficking to the cell membrane upon infection, which suppressed the interferon response to viral infection (51). NSP9 could activate carbamoyl-phosphate synthetase, aspartate transcarbamoylase, and dihydroorotase (CAD) that catalyzes the rate-limiting steps of the *de novo* pyrimidine synthesis which influence the SARS-CoV-2 infection, as pyrimidine synthesis enzyme restores inflammatory response and depletes the nucleotide pool to impede SARS-CoV-2 infection (52). And, TBK1 was reported to bind with membrane (M) protein of SARS-CoV-2, which induced the K48-linked ubiquitination and degradation of TBK1 and negative regulate the production of IFN-I (53). Unfortunately, we didn’t find any connection between TBK1 and 7SL RNA, carbamoyl-phosphate synthetase, aspartate transcarbamoylase, CAD, or a relationship between NSP9 and M. While, confocal imaging identified that NSP9 was largely localized in close proximity to the endoplasmic reticulum and connect with nucleoporin 62 (NUP62) (54). TBK1 could recruit to STING or MAVS during viral infection, which localized closely to the endoplasmic reticulum. Our findings identified a novel mechanism that regulated the TBK1 pathway through SARS-COV-2 protein NSP9, which might occurred on the endoplasmic reticulum.

By regulating TBK1 activation and pro-inflammatory cytokine production, our study identified NSP9 as a positive regulator of precise control of the cellular antiviral immune response. These findings shed new light on how SARS-COV-2 and the host interact during infection, discovering a previously unknown mechanism of host-microbe interaction and the possibility of developing novel drug targets for treating COVID-19.

## Data availability statement

The raw data supporting the conclusions of this article will be made available by the authors, without undue reservation.

## Ethics statement

The animal study was approved by the Animal Research Ethics Committee of the Faculty of Fudan University. The study was conducted in accordance with the local legislation and institutional requirements.

## Author contributions

YZ and DY designed this study. YZ performed the experiments assisted by BX, WJ, WH and JD. PW and XH contributed to discussions and agreement with the conclusions. YZ and DY analyzed the data and wrote the manuscript. All authors contributed to the article and approved the submitted version.

## Supplementary material

Supplementary Table 1 Primer sequences used to amplify human gene and mouse gene in real-time quantitative PCR.

Supplementary Table 2 MS-Control.

Supplementary Table 3 MS-NSP9.
