# Supplementary material for: SARS-COV-2 protein NSP9 promotes cytokine production by targeting TBK1
Source: Front Immunol. 2023 Oct 2;14:1211816. doi: 10.3389/fimmu.2023.1211816 (PMC10580797; doi:10.3389/fimmu.2023.1211816)
Supplement: Supplementary Table 1 — Primer sequences used to amplify human gene and mouse gene in real-time quantitative PCR. [file DataSheet_1.docx]

Supplementary Material

SARS-COV-2 protein NSP9 promotes cytokine production by targeting TBK1

Yihua Zhang^1^, Bowen Xin^1^, Wenyi Jiang^1^, Wendong Han^2^, Jian Deng^3^, Peihui Wang^3^, Xiaowu Hong^1^ & Dapeng Yan^1^*

*** Correspondence:** Dapeng Yan: [dapengyan@fudan.edu.cn](mailto:dapengyan@fudan.edu.cn)

**FIGURE S1. Overexpression of NSP9 promotes cellular antiviral immune response.** (**A**) *Ifnb* mRNA levels in L929 cells transfected with various vectors and stimulated with VSV for 12 hours. **(B)** Immunoblot analysis of nuclear and cytoplasmic fractions in L929 cells transfected with control or NSP9 vectors and stimulated with SEV for indicated times. **(C, D)** Immunoblot analysis of monomeric and dimeric IRF3 in L929 cells transfected with control or NSP9 vectors and stimulated with VSV **(C)** or SEV **(D)** for indicated times. (**E**) CCK-8 assay for cell viability in L929 cells transfected with vector or NSP9 for 3 days or 5 days. ***P < 0.001, two-tailed unpaired Student’s *t*-test.

**FIGURE S2. MID1 contributed to the K48-linked ubiquitination and degradation of NSP9.** (**A**, **B**) Immunoassay of cell lysates from HEK293T cells (**A**) or L929 cells (**B**) transfected with various vectors and infected with VSV for 12 hours, then stimulated with DMSO or MG132. (**C**) Immunoassay of cell lysates from HEK293T cells transfected with various vectors. (**D**) Immunoassay of cell lysates from HEK293T cells transfected with various vectors and stimulated with DMSO or MG132. (**E**) Immunoassay of cell lysates from L929 cells transfected with various vectors and infected with VSV for 12 hours and stimulated with DMSO or MG132. (**F**) Immunoblot of lysates of L929 cells transfected with control or NSP9 vectors and infected with VSV for indicated times. (**G**, **H**) *Isg15*, *Ccl5*, *Il6* and *Tnf* mRNA levels in L929 cells transfected with control or NSP9 vectors and stimulated with VSV (**G**) or SEV (**H**) for 36 hours. Data are representative of at least three independent experiments (mean ± SEM in **G**, **H**). ***P < 0.001, two-tailed unpaired Student’s *t*-test.

**FIGURE S3. Lys59 was responsible for NSP9 degradation.** (**A**) Immunoassay of cell lysates from HEK293T cells transfected with various vectors and infected with VSV for 12 hours and stimulated with MG132. (**B**, **C**) *Isg15*, *Ccl5*, *Il6* and *Tnf* mRNA levels in L929 cells transfected with the indicated vectors and stimulated with VSV (**B**) or SEV (**C**) for 36 hours. (**D**) Immunoassay of cell lysates from HEK293T cells transfected with various vectors. (**E**) Immunoassay of cell lysates from HEK293T cells transfected with various vectors and stimulated with DMSO or MG132. Data are representative of at least three independent experiments (mean ± SEM in **B**, **C**). ***P < 0.001, two-tailed unpaired Student’s *t*-test.

**FIGURE S4. Diagram depicting the positive regulation of NSP9 on TBK1-mediated cytokine production.**

**S1 Table. Primer sequences used to amplify human gene and mouse gene in real-time quantitative PCR.**

| Human *Ifnb* | forward | TCTGGCACAACAGGTAGTAGGC |
| --- | --- | --- |
|  | reverse | GAGAAGCACAACAGGAG |
| Human *Cxcl10* | forward | GGAACCTCCAGTCTCAGCACCA |
|  | reverse | AGACATCTCTTCTCACCCTTC |
| Human *Isg15* | forward | TTTGCCAGTACAGGAGCTTGTG |
|  | reverse | GGGTGATCTGCGCCTTCA |
| Human *Il6* | forward | TCCAGTTGCCTTCTTGGGAC |
|  | reverse | GTGTAATTAAGCCTCCGACTTG |
| Human *Tnf* | forward | CACAGTGAAGTGCTGGCAAC |
|  | reverse | AGGAAGGCCTAAGGTCCACT |
| Human *GAPDH* | forward | GCAAATTCCATGGCACCGT |
|  | reverse | GCCCCACTTGATTTTGGAGG |
| mouse *Ifnb* | forward | AGTTACACTGCCTTTGCC |
|  | reverse | GTTGAGGACATCTCCCAC |
| mouse *Cxcl10* | forward | CCAAGTGCTGCCGTCATTTT |
|  | reverse | GATAGGCTCGCAGGGATGAT |
| mouse *Isg15* | forward | GGTGTCCGTGACTAACTCCAT |
|  | reverse | TGGAAAGGGTAAGACCGTCCT |
| mouse *Il6* | forward | ACCCCCAATAAATATAGGACTGGA |
|  | reverse | TCTTCTCCTGGGGGTACTGG |
| mouse *Tnf* | forward | TTCTGTCTACTGAACTTCGGGGTGATCGGTCC |
|  | reverse | GTATGAGATAGCAAATCGGCTGACGGTGTGGG |
| mouse *Gapdh* | forward | CCCACTAACATCAAATGGGG |
|  | reverse | CCTTCCACAATGCCAAAGTT |
