# Supplementary material for: SARS-COV-2 protein NSP9 promotes cytokine production by targeting TBK1
Source: Front Immunol. 2023 Oct 2;14:1211816. doi: 10.3389/fimmu.2023.1211816 (PMC10580797; doi:10.3389/fimmu.2023.1211816)
Supplement: Supplementary file 2 [file Presentation_1.pptx]

## Slide 1
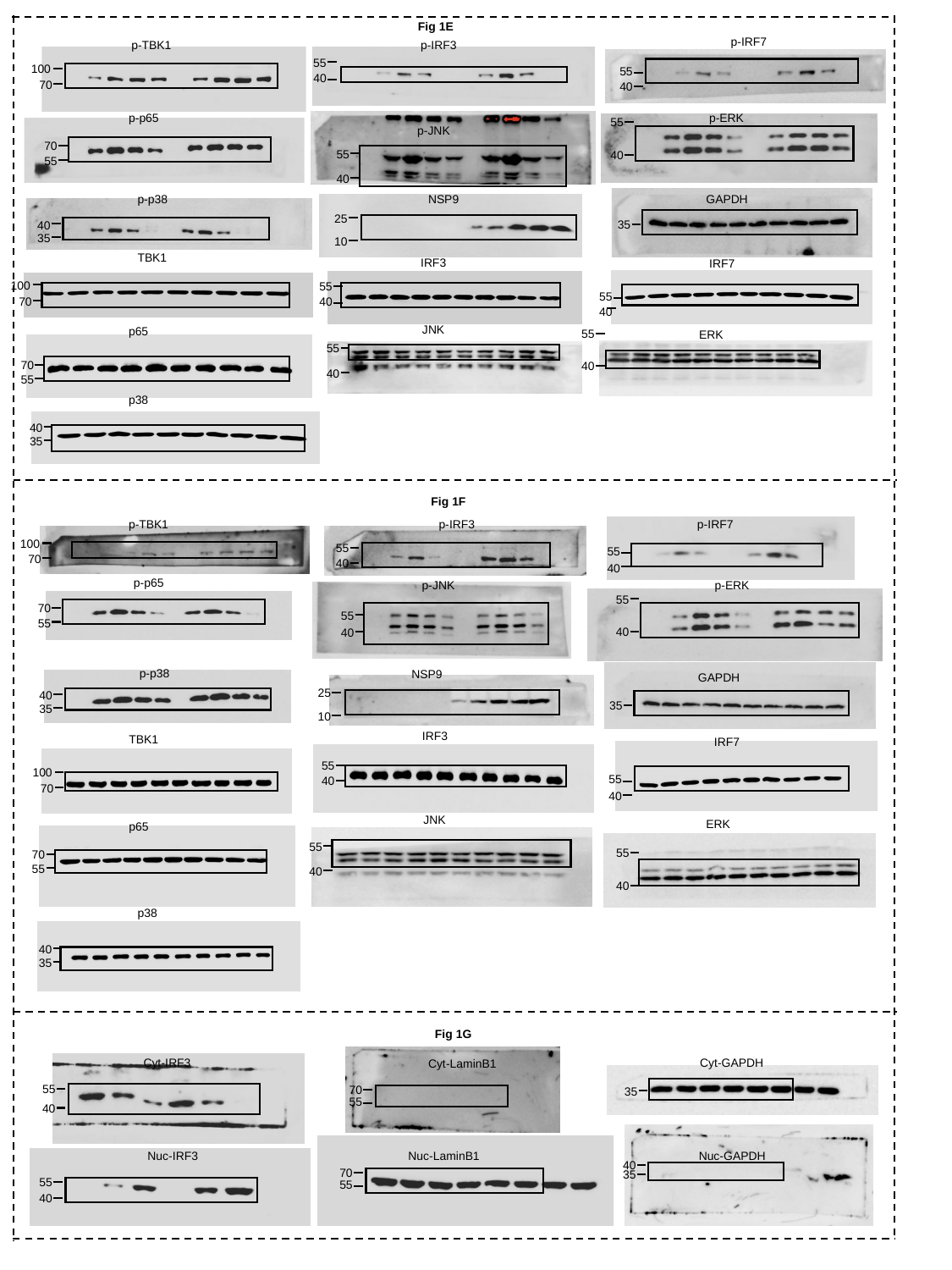

Fig 1E
p-IRF7
p-TBK1
p-IRF3
55
100
55
40
70
40
p-p65
p-ERK
55
p-JNK
70
55
40
55
40
p-p38
NSP9
GAPDH
25
35
40
35
10
TBK1
IRF3
IRF7
100
55
55
70
40
40
JNK
p65
ERK
55
55
70
40
40
55
p38
40
35
Fig 1F
p-TBK1
p-IRF3
p-IRF7
100
55
55
70
40
40
p-p65
p-JNK
p-ERK
55
70
55
55
40
40
p-p38
NSP9
GAPDH
25
40
35
35
10
IRF3
TBK1
IRF7
55
100
55
40
70
40
JNK
ERK
p65
55
55
70
55
40
40
p38
40
35
Fig 1G
Cyt-IRF3
Cyt-GAPDH
Cyt-LaminB1
55
70
35
55
40
Nuc-IRF3
Nuc-LaminB1
Nuc-GAPDH
40
70
35
55
55
40

## Slide 2
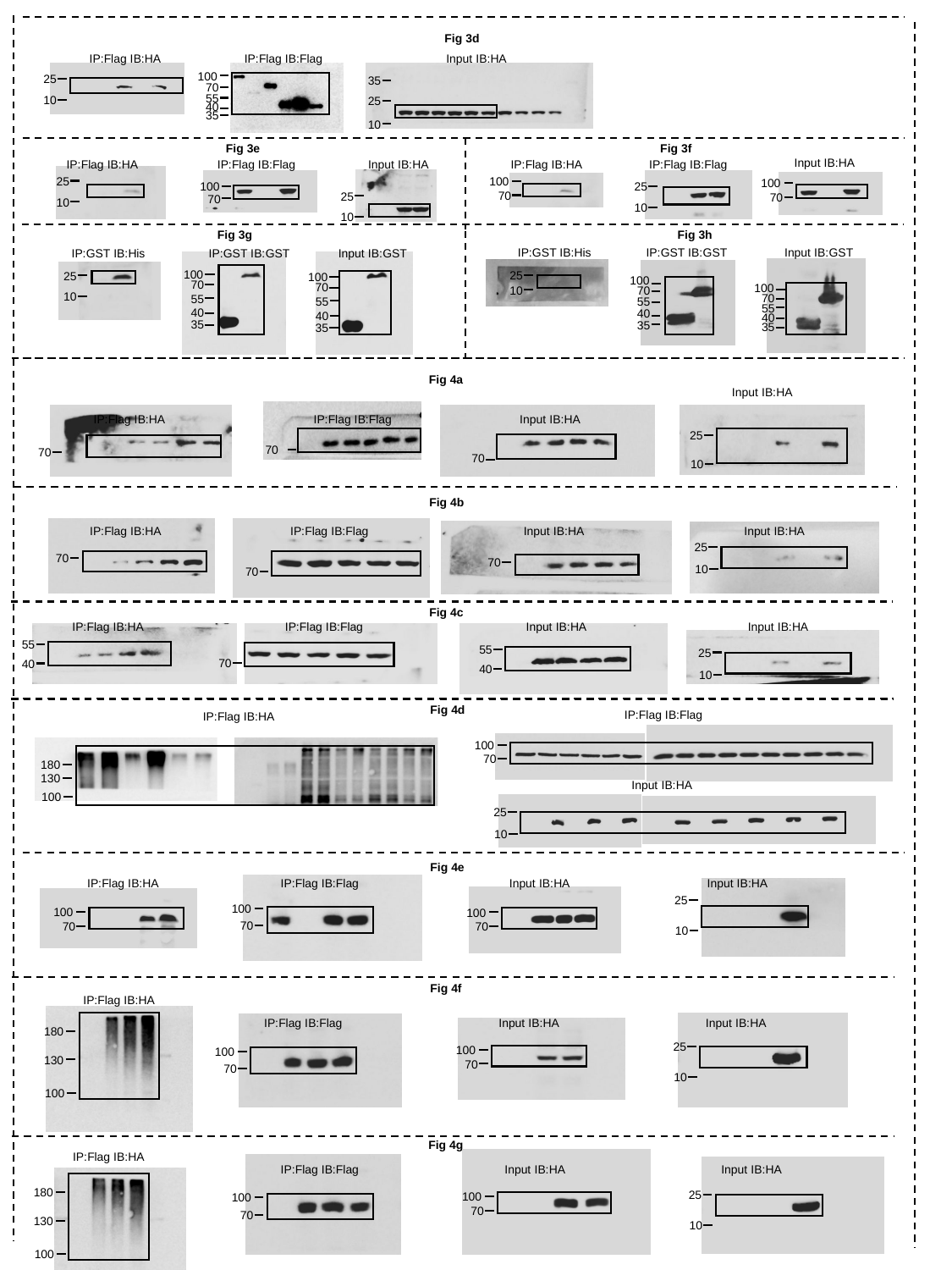

40
Fig 3d
IP:Flag IB:HA
IP:Flag IB:Flag
Input IB:HA
100
25
35
70
55
10
25
40
35
10
Fig 3e
Fig 3f
Input IB:HA
IP:Flag IB:HA
IP:Flag IB:Flag
Input IB:HA
IP:Flag IB:HA
IP:Flag IB:Flag
25
100
100
100
25
70
25
70
70
10
10
10
Fig 3g
Fig 3h
IP:GST IB:His
IP:GST IB:GST
Input IB:GST
IP:GST IB:His
IP:GST IB:GST
Input IB:GST
100
25
25
100
100
70
70
100
10
70
10
70
55
55
55
55
40
40
40
40
35
35
35
35
Fig 4a
Input IB:HA
IP:Flag IB:HA
IP:Flag IB:Flag
Input IB:HA
25
70
70
70
10
Fig 4b
IP:Flag IB:HA
IP:Flag IB:Flag
Input IB:HA
Input IB:HA
25
70
70
10
70
Fig 4c
IP:Flag IB:HA
IP:Flag IB:Flag
Input IB:HA
Input IB:HA
55
55
25
70
40
40
10
Fig 4d
IP:Flag IB:Flag
IP:Flag IB:HA
100
70
180
130
Input IB:HA
100
25
10
Fig 4e
IP:Flag IB:HA
IP:Flag IB:Flag
Input IB:HA
Input IB:HA
25
100
100
100
70
70
70
10
Fig 4f
IP:Flag IB:HA
IP:Flag IB:Flag
Input IB:HA
Input IB:HA
180
25
100
100
130
70
70
10
100
Fig 4g
IP:Flag IB:HA
IP:Flag IB:Flag
Input IB:HA
Input IB:HA
180
25
100
100
70
70
130
10
100

## Slide 3
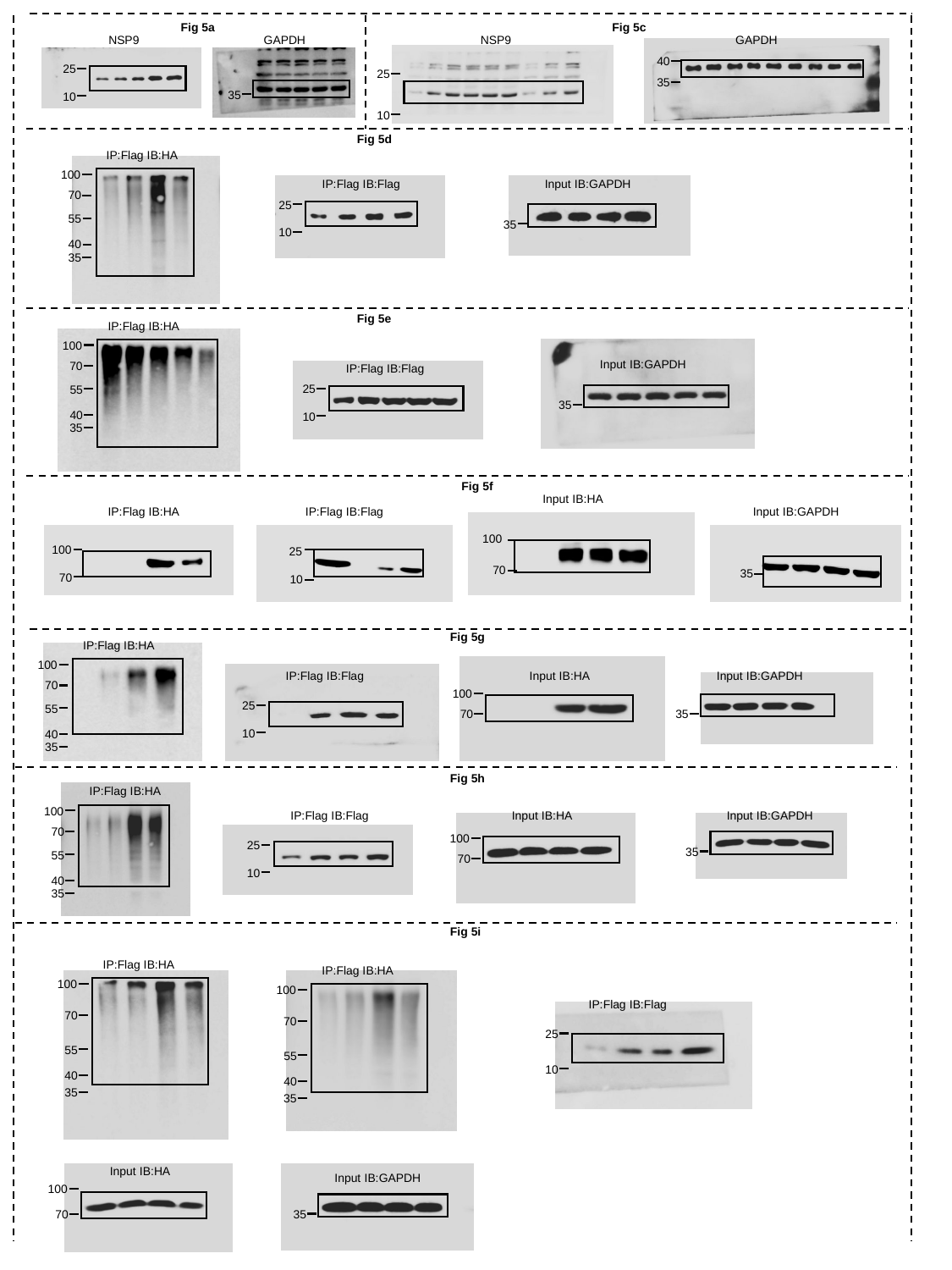

Fig 5a
Fig 5c
NSP9
GAPDH
NSP9
GAPDH
40
25
25
35
35
10
10
Fig 5d
IP:Flag IB:HA
100
IP:Flag IB:Flag
Input IB:GAPDH
70
25
55
35
10
40
35
Fig 5e
IP:Flag IB:HA
100
Input IB:GAPDH
IP:Flag IB:Flag
70
25
55
35
40
10
35
Fig 5f
Input IB:HA
Input IB:GAPDH
IP:Flag IB:HA
IP:Flag IB:Flag
100
100
100
25
70
70
35
70
10
Fig 5g
IP:Flag IB:HA
100
IP:Flag IB:Flag
Input IB:HA
Input IB:GAPDH
70
100
25
55
35
70
10
40
35
Fig 5h
IP:Flag IB:HA
IP:Flag IB:Flag
Input IB:HA
Input IB:GAPDH
100
70
100
25
35
55
70
10
40
35
Fig 5i
IP:Flag IB:HA
IP:Flag IB:HA
100
100
IP:Flag IB:Flag
70
70
25
55
55
10
40
40
35
35
Input IB:HA
Input IB:GAPDH
100
35
70

## Slide 4
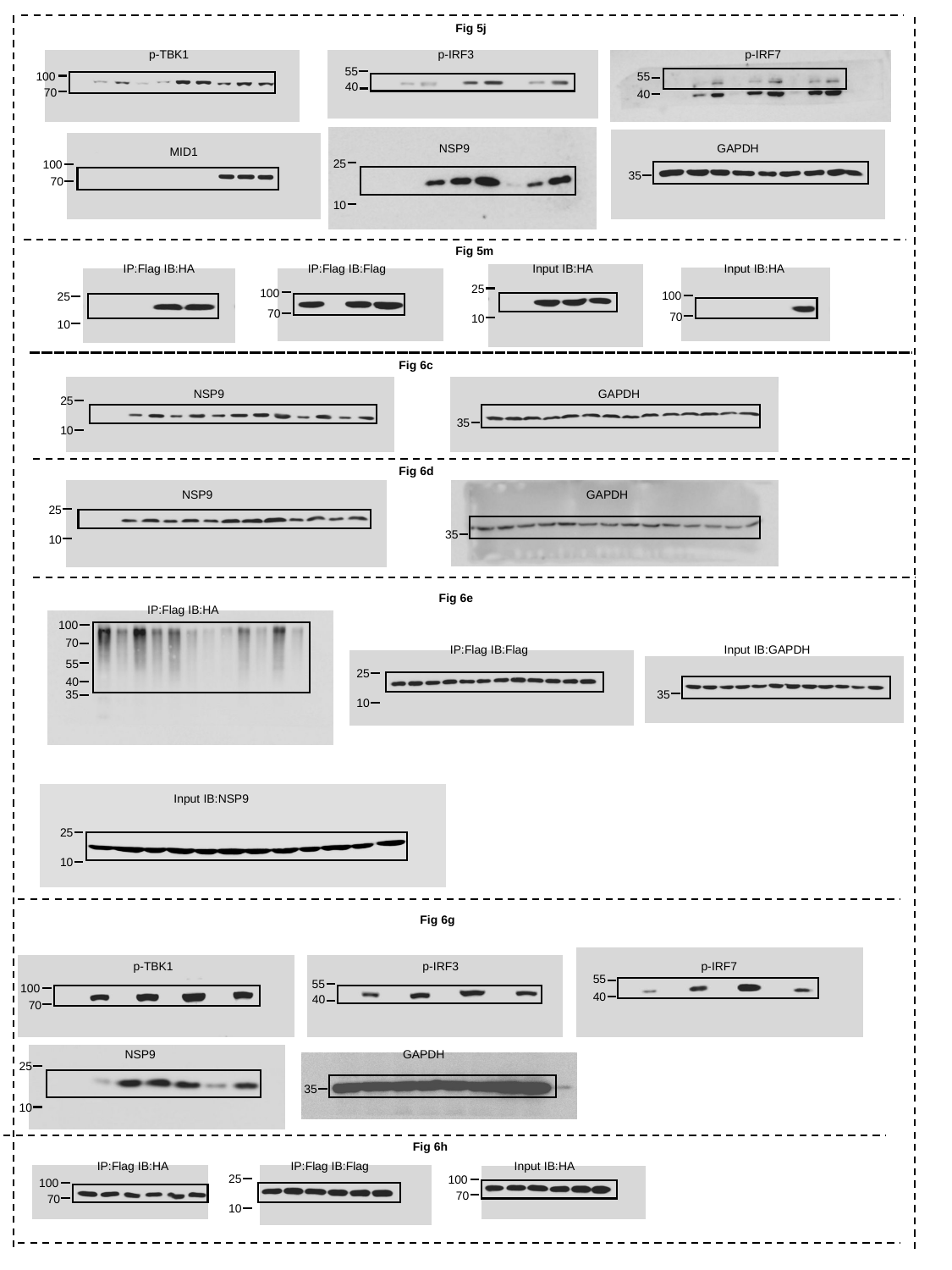

Fig 5j
p-TBK1
p-IRF3
p-IRF7
55
55
100
40
70
40
NSP9
GAPDH
MID1
25
100
35
70
10
Fig 5m
IP:Flag IB:HA
IP:Flag IB:Flag
Input IB:HA
Input IB:HA
25
100
100
25
70
70
10
10
Fig 6c
NSP9
GAPDH
25
35
10
Fig 6d
NSP9
GAPDH
25
35
10
Fig 6e
IP:Flag IB:HA
100
70
IP:Flag IB:Flag
Input IB:GAPDH
55
25
40
35
35
10
Input IB:NSP9
25
10
Fig 6g
p-TBK1
p-IRF3
p-IRF7
55
55
100
40
40
70
NSP9
GAPDH
25
35
10
Fig 6h
IP:Flag IB:HA
IP:Flag IB:Flag
Input IB:HA
25
100
100
70
70
10

## Slide 5
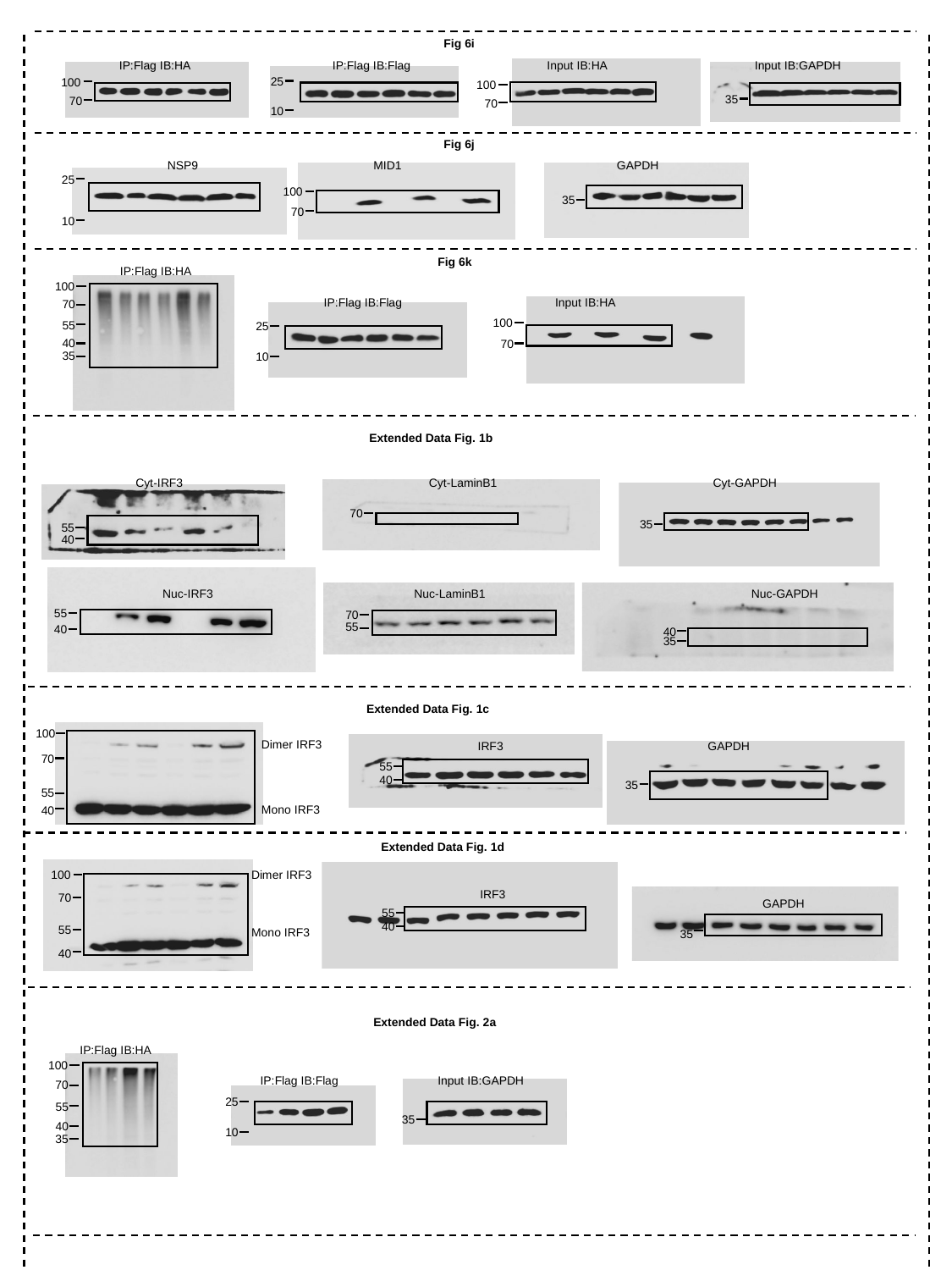

Fig 6i
IP:Flag IB:HA
IP:Flag IB:Flag
Input IB:HA
Input IB:GAPDH
25
100
100
35
70
70
10
Fig 6j
NSP9
MID1
GAPDH
25
100
35
70
10
Fig 6k
IP:Flag IB:HA
100
IP:Flag IB:Flag
Input IB:HA
70
100
55
25
40
70
35
10
Extended Data Fig. 1b
Cyt-IRF3
Cyt-LaminB1
Cyt-GAPDH
70
35
55
40
Nuc-IRF3
Nuc-LaminB1
Nuc-GAPDH
55
70
55
40
40
35
Extended Data Fig. 1c
100
Dimer IRF3
IRF3
GAPDH
70
55
40
35
55
Mono IRF3
40
Extended Data Fig. 1d
Dimer IRF3
100
IRF3
GAPDH
70
55
Mono IRF3
40
55
35
40
Extended Data Fig. 2a
IP:Flag IB:HA
100
IP:Flag IB:Flag
Input IB:GAPDH
70
25
55
35
40
10
35

## Slide 6
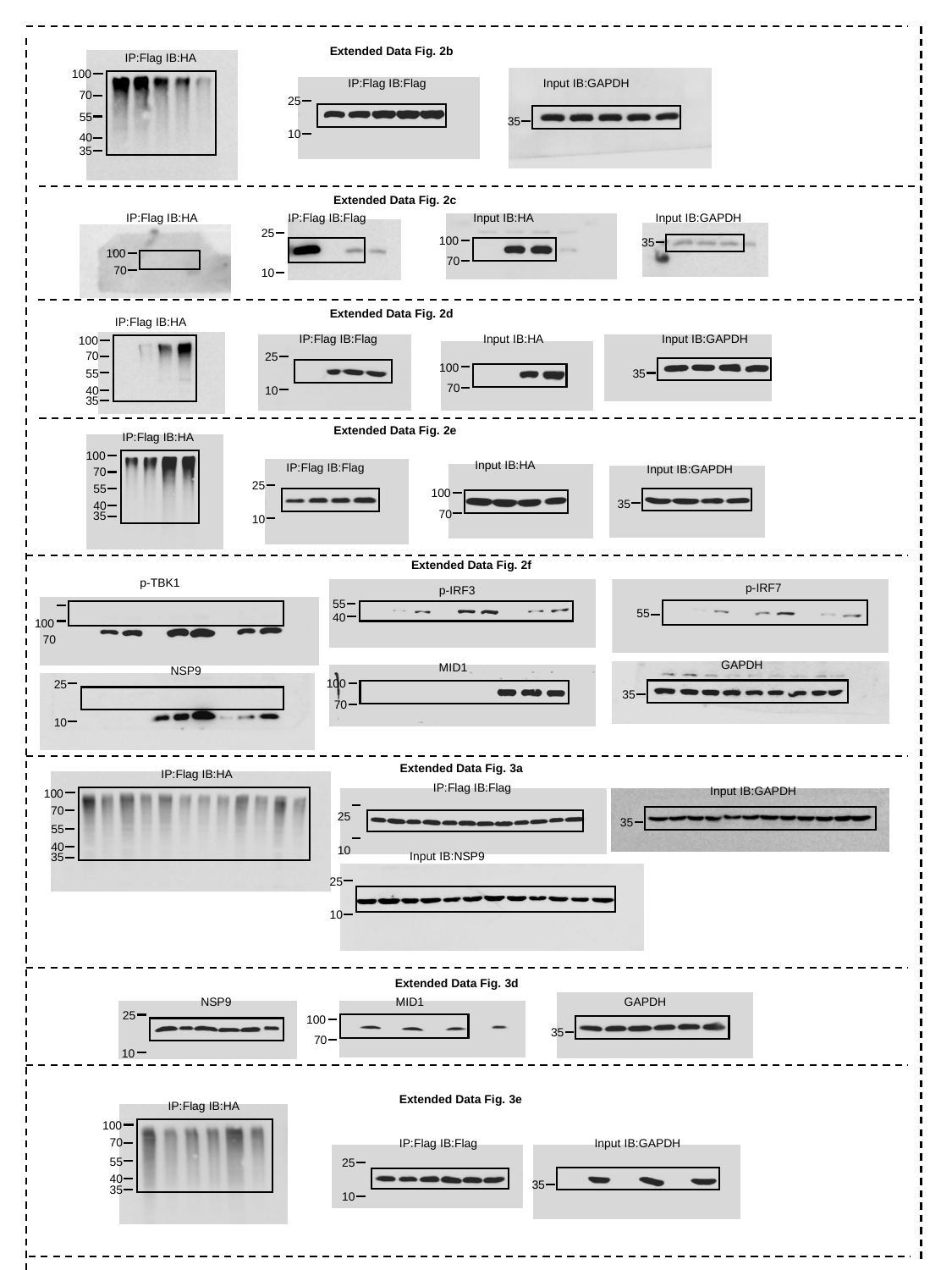

Extended Data Fig. 2b
IP:Flag IB:HA
100
IP:Flag IB:Flag
Input IB:GAPDH
70
25
55
35
10
40
35
Extended Data Fig. 2c
IP:Flag IB:HA
IP:Flag IB:Flag
Input IB:HA
Input IB:GAPDH
25
100
35
100
70
70
10
Extended Data Fig. 2d
IP:Flag IB:HA
IP:Flag IB:Flag
Input IB:HA
Input IB:GAPDH
100
70
25
100
35
55
70
40
10
35
Extended Data Fig. 2e
IP:Flag IB:HA
100
Input IB:HA
IP:Flag IB:Flag
Input IB:GAPDH
70
25
55
100
35
40
70
35
10
Extended Data Fig. 2f
p-TBK1
p-IRF7
p-IRF3
55
55
40
100
70
GAPDH
MID1
NSP9
100
25
35
70
10
Extended Data Fig. 3a
IP:Flag IB:HA
IP:Flag IB:Flag
Input IB:GAPDH
100
70
25
35
55
40
10
Input IB:NSP9
35
25
10
Extended Data Fig. 3d
NSP9
MID1
GAPDH
25
100
35
70
10
Extended Data Fig. 3e
IP:Flag IB:HA
100
IP:Flag IB:Flag
Input IB:GAPDH
70
55
25
40
35
35
10
