# Supplementary material for: SARS-COV-2 protein NSP9 promotes cytokine production by targeting TBK1
Source: Front Immunol. 2023 Oct 2;14:1211816. doi: 10.3389/fimmu.2023.1211816 (PMC10580797; doi:10.3389/fimmu.2023.1211816)
Supplement: Supplementary file 3 [file Presentation_2.pdf]

Protein FDR	Accession	Description	Sum PEP Score
High	P35579	Myosin-9 OS=Homo sapiens OX=9606 GN=MYH9 PE=1 S\	920.5495552
High	P35580	Myosin-10 OS=Homo sapiens OX=9606 GN=MYH10 PE=1	774.3371903
High	P11586	C-1-tetrahydrofolate synthase, cytoplasmic OS=Homo sa	582.6465208
High	P49327	Fatty acid synthase OS=Homo sapiens OX=9606 GN=FASI	373.0007357
High	P21333	Filamin-A OS=Homo sapiens OX=9606 GN=FLNA PE=1 SV	361.8212785
High	P08238	Heat shock protein HSP 90-beta OS=Homo sapiens OX=9	354.327325
High	P13639	Elongation factor 2 OS=Homo sapiens OX=9606 GN=EEF2	341.7309536
High	Q7Z406	Myosin-14 OS=Homo sapiens OX=9606 GN=MYH14 PE=1	305.7724931
High	P07900	Heat shock protein HSP 90-alpha OS=Homo sapiens OX=9	304.6047965
High	P42704	Leucine-rich PPR motif-containing protein, mitochondria	257.1717346
High	O75369	Filamin-B OS=Homo sapiens OX=9606 GN=FLNB PE=1 SV	254.5793077
High	Q00610	Clathrin heavy chain 1 OS=Homo sapiens OX=9606 GN=C	254.4509508
High	P0DMV9	Heat shock 70 kDa protein 1B OS=Homo sapiens OX=960	251.9204516
High	P22314	Ubiquitin-like modifier-activating enzyme 1 OS=Homo sa	247.2944551
High	P60709	Actin, cytoplasmic 1 OS=Homo sapiens OX=9606 GN=ACT	239.3568553
High	P04264	Keratin, type II cytoskeletal 1 OS=Homo sapiens OX=960	236.5919599
High	P04406	Glyceraldehyde-3-phosphate dehydrogenase OS=Homo s	225.8974959
High	P68371	Tubulin beta-4B chain OS=Homo sapiens OX=9606 GN=T	223.8522046
High	P07437	Tubulin beta chain OS=Homo sapiens OX=9606 GN=TUBE	223.2709726
High	P07814	Bifunctional glutamate/proline--tRNA ligase OS=Homo sa	221.7942901
High	P78527	DNA-dependent protein kinase catalytic subunit OS=Hom	209.2278013
High	P13645	Keratin, type I cytoskeletal 10 OS=Homo sapiens OX=960	208.0753163
High	Q7KZF4	Staphylococcal nuclease domain-containing protein 1 OS	204.4431691
High	P11142	Heat shock cognate 71 kDa protein OS=Homo sapiens OX	198.8341631
High	Q92616	eIF-2-alpha kinase activator GCN1 OS=Homo sapiens OX=	197.2616186
High	P41252	Isoleucine--tRNA ligase, cytoplasmic OS=Homo sapiens C	196.103965
High	Q14697	Neutral alpha-glucosidase AB OS=Homo sapiens OX=960	194.3124704
High	P09874	Poly [ADP-ribose] polymerase 1 OS=Homo sapiens OX=9	191.3006722
High	P14618	Pyruvate kinase PKM OS=Homo sapiens OX=9606 GN=PK	189.678516
High	Q13885	Tubulin beta-2A chain OS=Homo sapiens OX=9606 GN=T	187.4616208
High	P49588	Alanine--tRNA ligase, cytoplasmic OS=Homo sapiens OX=	184.8359887
High	P35527	Keratin, type I cytoskeletal 9 OS=Homo sapiens OX=9606	183.8133765
High	P06733	Alpha-enolase OS=Homo sapiens OX=9606 GN=ENO1 PE=	183.3634183
High	P04350	Tubulin beta-4A chain OS=Homo sapiens OX=9606 GN=T	180.3924146
High	P33993	DNA replication licensing factor MCM7 OS=Homo sapien	177.4680699
High	Q86VP6	Cullin-associated NEDD8-dissociated protein 1 OS=Homo	176.15468
High	Q9P2J5	Leucine--tRNA ligase, cytoplasmic OS=Homo sapiens OX=	174.1428779
High	P35908	Keratin, type II cytoskeletal 2 epidermal OS=Homo sapie	172.5647524
High	P52732	Kinesin-like protein KIF11 OS=Homo sapiens OX=9606 GI	170.4929305
High	O14980	Exportin-1 OS=Homo sapiens OX=9606 GN=XPO1 PE=1 S	168.7866632
High	P33991	DNA replication licensing factor MCM4 OS=Homo sapien	166.1308995
High	P55060	Exportin-2 OS=Homo sapiens OX=9606 GN=CSE1L PE=1 S	164.1998595
High	P27708	CAD protein OS=Homo sapiens OX=9606 GN=CAD PE=1 S	159.1506325
High	Q9BQE3	Tubulin alpha-1C chain OS=Homo sapiens OX=9606 GN=T	153.6838492
High	P11021	Endoplasmic reticulum chaperone BiP OS=Homo sapiens	150.6935388
High	P68363	Tubulin alpha-1B chain OS=Homo sapiens OX=9606 GN=T	148.3700574

High	P22102	Trifunctional purine biosynthetic protein adenosine-3 OS	146.4388705
High	Q9Y4L1	Hypoxia up-regulated protein 1 OS=Homo sapiens OX=96	143.4348807
High	Q9UJZ1	Stomatin-like protein 2, mitochondrial OS=Homo sapiens	140.070893
High	Q71U36	Tubulin alpha-1A chain OS=Homo sapiens OX=9606 GN=T	139.6996094
High	P49368	T-complex protein 1 subunit gamma OS=Homo sapiens O	138.0946601
High	P14625	Endoplasmin OS=Homo sapiens OX=9606 GN=HSP90B1 P	137.3122175
High	P55786	Puromycin-sensitive aminopeptidase OS=Homo sapiens (	137.2100872
High	Q13263	Transcription intermediary factor 1-beta OS=Homo sapie	136.0757811
High	Q00839	Heterogeneous nuclear ribonucleoprotein U OS=Homo s	130.4806817
High	P49915	GMP synthase [glutamine-hydrolyzing] OS=Homo sapien	127.6449423
High	Q14974	Importin subunit beta-1 OS=Homo sapiens OX=9606 GN=	127.448672
High	P26640	Valine--tRNA ligase OS=Homo sapiens OX=9606 GN=VAR	126.0991452
High	P05023	Sodium/potassium-transporting ATPase subunit alpha-1	125.9154949
High	P41250	Glycine--tRNA ligase OS=Homo sapiens OX=9606 GN=GA	123.5212798
High	O00410	Importin-5 OS=Homo sapiens OX=9606 GN=IPO5 PE=1 SV	120.4459169
High	P27824	Calnexin OS=Homo sapiens OX=9606 GN=CANX PE=1 SV=	120.0033
High	P68104	Elongation factor 1-alpha 1 OS=Homo sapiens OX=9606 (	112.4411751
High	Q14566	DNA replication licensing factor MCM6 OS=Homo sapien	112.0475909
High	Q4VCS5	Angiomotin OS=Homo sapiens OX=9606 GN=AMOT PE=1	111.4171729
High	P68032	Actin, alpha cardiac muscle 1 OS=Homo sapiens OX=960(	109.1392008
High	P49736	DNA replication licensing factor MCM2 OS=Homo sapien	108.2357553
High	Q9NSE4	Isoleucine--tRNA ligase, mitochondrial OS=Homo sapiens	106.8720908
High	P25205	DNA replication licensing factor MCM3 OS=Homo sapien	106.5618367
High	Q04637	Eukaryotic translation initiation factor 4 gamma 1 OS=Ho	106.2651108
High	P34931	Heat shock 70 kDa protein 1-like OS=Homo sapiens OX=9	106.1410655
High	Q13200	26S proteasome non-ATPase regulatory subunit 2 OS=Ho	104.4126776
High	P38646	Stress-70 protein, mitochondrial OS=Homo sapiens OX=9	101.3413245
High	P53618	Coatomer subunit beta OS=Homo sapiens OX=9606 GN=	100.636416
High	O15067	Phosphoribosylformylglycinamidine synthase OS=Homo	99.43638055
High	Q14008	Cytoskeleton-associated protein 5 OS=Homo sapiens OX=	99.20809168
High	O95373	Importin-7 OS=Homo sapiens OX=9606 GN=IPO7 PE=1 SV	98.95985918
High	P56192	Methionine--tRNA ligase, cytoplasmic OS=Homo sapiens	97.45165627
High	P46940	Ras GTPase-activating-like protein IQGAP1 OS=Homo sap	96.2796502
High	P49411	Elongation factor Tu, mitochondrial OS=Homo sapiens O	93.80464359
High	O95347	Structural maintenance of chromosomes protein 2 OS=H	92.69948329
High	Q92841	Probable ATP-dependent RNA helicase DDX17 OS=Homo	89.55172774
High	P60842	Eukaryotic initiation factor 4A-I OS=Homo sapiens OX=96	87.73587154
High	Q14166	Tubulin--tyrosine ligase-like protein 12 OS=Homo sapiens	87.33803715
High	P16615	Sarcoplasmic/endoplasmic reticulum calcium ATPase 2 C	86.44630557
High	O00429	Dynamin-1-like protein OS=Homo sapiens OX=9606 GN=	86.33970183
High	P22626	Heterogeneous nuclear ribonucleoproteins A2/B1 OS=Ho	86.21298367
High	Q08J23	tRNA (cytosine(34)-C(5))-methyltransferase OS=Homo sa	85.63641354
High	Q9Y2L1	Exosome complex exonuclease RRP44 OS=Homo sapiens	85.45624722
High	Q08211	ATP-dependent RNA helicase A OS=Homo sapiens OX=96	84.47884026
High	P19338	Nucleolin OS=Homo sapiens OX=9606 GN=NCL PE=1 SV=	83.08182299
High	P23246	Splicing factor, proline- and glutamine-rich OS=Homo sap	80.53115439
High	Q15365	Poly(rC)-binding protein 1 OS=Homo sapiens OX=9606 G	80.09716314

High	P12268	Inosine-5'-monophosphate dehydrogenase 2 OS=Homo s	79.92046028
High	P34932	Heat shock 70 kDa protein 4 OS=Homo sapiens OX=9606	79.46299094
High	Q8TEX9	Importin-4 OS=Homo sapiens OX=9606 GN=IPO4 PE=1 SV	78.50808901
High	Q6P2Q9	Pre-mRNA-processing-splicing factor 8 OS=Homo sapiens	77.96977669
High	Q9BUF5	Tubulin beta-6 chain OS=Homo sapiens OX=9606 GN=TU	75.89490776
High	P10809	60 kDa heat shock protein, mitochondrial OS=Homo sapi	74.7376022
High	O75533	Splicing factor 3B subunit 1 OS=Homo sapiens OX=9606 (	74.56883964
High	P02533	Keratin, type I cytoskeletal 14 OS=Homo sapiens OX=960	74.5574444
High	P17987	T-complex protein 1 subunit alpha OS=Homo sapiens OX	74.52436881
High	Q96QK1	Vacuolar protein sorting-associated protein 35 OS=Homo	73.58989244
High	P52272	Heterogeneous nuclear ribonucleoprotein M OS=Homo s	73.39902629
High	P17066	Heat shock 70 kDa protein 6 OS=Homo sapiens OX=9606	71.12339736
High	P54652	Heat shock-related 70 kDa protein 2 OS=Homo sapiens C	70.50751482
High	Q15366	Poly(rC)-binding protein 2 OS=Homo sapiens OX=9606 G	69.69950551
High	P28331	NADH-ubiquinone oxidoreductase 75 kDa subunit, mitoc	69.64440361
High	Q92945	Far upstream element-binding protein 2 OS=Homo sapie	69.28093194
High	P08865	40S ribosomal protein SA OS=Homo sapiens OX=9606 GN	69.11260397
High	P43243	Matrin-3 OS=Homo sapiens OX=9606 GN=MATR3 PE=1 S	69.0860798
High	O60763	General vesicular transport factor p115 OS=Homo sapien	67.87505621
High	P48643	T-complex protein 1 subunit epsilon OS=Homo sapiens O	67.26109835
High	Q14315	Filamin-C OS=Homo sapiens OX=9606 GN=FLNC PE=1 SV=	67.19809386
High	Q15021	Condensin complex subunit 1 OS=Homo sapiens OX=960	66.50961475
High	P33176	Kinesin-1 heavy chain OS=Homo sapiens OX=9606 GN=KI	66.4831308
High	Q00341	Vigilin OS=Homo sapiens OX=9606 GN=HDLBP PE=1 SV=2	65.98311082
High	Q15029	116 kDa U5 small nuclear ribonucleoprotein component	65.76403991
High	P47897	Glutamine--tRNA ligase OS=Homo sapiens OX=9606 GN=	65.6432104
High	P54886	Delta-1-pyrroline-5-carboxylate synthase OS=Homo sapie	65.42905523
High	P61978	Heterogeneous nuclear ribonucleoprotein K OS=Homo sa	64.75847833
High	P09651	Heterogeneous nuclear ribonucleoprotein A1 OS=Homo	64.47954791
High	Q13620	Cullin-4B OS=Homo sapiens OX=9606 GN=CUL4B PE=1 SV	63.79282557
High	Q16643	Drebrin OS=Homo sapiens OX=9606 GN=DBN1 PE=1 SV=	63.53722428
High	O75694	Nuclear pore complex protein Nup155 OS=Homo sapiens	63.03486951
High	P26641	Elongation factor 1-gamma OS=Homo sapiens OX=9606 (	62.8498087
High	P63244	Receptor of activated protein C kinase 1 OS=Homo sapie	62.69382133
High	P25705	ATP synthase subunit alpha, mitochondrial OS=Homo sap	62.47757167
High	Q93009	Ubiquitin carboxyl-terminal hydrolase 7 OS=Homo sapien	62.27045454
High	P04843	Dolichyl-diphosphooligosaccharide--protein glycosyltrans	61.33862093
High	O00571	ATP-dependent RNA helicase DDX3X OS=Homo sapiens (	60.2792716
High	P40939	Trifunctional enzyme subunit alpha, mitochondrial OS=H	59.42715661
High	Q14152	Eukaryotic translation initiation factor 3 subunit A OS=Hc	59.09083534
High	O75643	U5 small nuclear ribonucleoprotein 200 kDa helicase OS=	59.05368209
High	Q12931	Heat shock protein 75 kDa, mitochondrial OS=Homo sap	58.05836812
High	Q9BQG0	Myb-binding protein 1A OS=Homo sapiens OX=9606 GN=	57.9018095
High	O96008	Mitochondrial import receptor subunit TOM40 homolog	57.64960431
High	Q14568	Heat shock protein HSP 90-alpha A2 OS=Homo sapiens C	57.52864406
High	Q92499	ATP-dependent RNA helicase DDX1 OS=Homo sapiens OX	56.56662424
High	P14866	Heterogeneous nuclear ribonucleoprotein L OS=Homo sa	56.37527529

High	Q96P70	Importin-9 OS=Homo sapiens OX=9606 GN=IPO9 PE=1 SV	56.26851173
High	Q14203	Dynactin subunit 1 OS=Homo sapiens OX=9606 GN=DCTI	56.11541913
High	O94832	Unconventional myosin-Id OS=Homo sapiens OX=9606 G	55.86208231
High	A5YKK6	CCR4-NOT transcription complex subunit 1 OS=Homo sap	55.7542634
High	P55265	Double-stranded RNA-specific adenosine deaminase OS=	55.5804048
High	Q5VYK3	Proteasome adapter and scaffold protein ECM29 OS=Hor	55.07528799
High	P53621	Coatomer subunit alpha OS=Homo sapiens OX=9606 GN=	54.90377591
High	P78371	T-complex protein 1 subunit beta OS=Homo sapiens OX=	54.80785805
High	P43246	DNA mismatch repair protein Msh2 OS=Homo sapiens O	54.75916411
High	P15924	Desmoplakin OS=Homo sapiens OX=9606 GN=DSP PE=1	54.34440193
High	Q14694	Ubiquitin carboxyl-terminal hydrolase 10 OS=Homo sapie	54.07864368
High	P52701	DNA mismatch repair protein Msh6 OS=Homo sapiens O	53.67661972
High	Q9Y3F4	Serine-threonine kinase receptor-associated protein OS=	52.61387482
High	P55072	Transitional endoplasmic reticulum ATPase OS=Homo sa	52.59997197
High	Q9UQE7	Structural maintenance of chromosomes protein 3 OS=H	52.54477734
High	P24752	Acetyl-CoA acetyltransferase, mitochondrial OS=Homo s	52.17708939
High	P06737	Glycogen phosphorylase, liver form OS=Homo sapiens O	52.05230653
High	P26639	Threonine--tRNA ligase, cytoplasmic OS=Homo sapiens C	51.98457235
High	P08195	4F2 cell-surface antigen heavy chain OS=Homo sapiens C	51.83370595
High	P78344	Eukaryotic translation initiation factor 4 gamma 2 OS=Hc	51.45287131
High	O60716	Catenin delta-1 OS=Homo sapiens OX=9606 GN=CTNND1	51.3241013
High	Q8WUM4	Programmed cell death 6-interacting protein OS=Homo s	50.77580418
High	O60749	Sorting nexin-2 OS=Homo sapiens OX=9606 GN=SNX2 PE	50.72356189
High	P04075	Fructose-bisphosphate aldolase A OS=Homo sapiens OX=	50.71426703
High	Q29RF7	Sister chromatid cohesion protein PDS5 homolog A OS=H	50.56278967
High	Q16891	MICOS complex subunit MIC60 OS=Homo sapiens OX=96	50.39326128
High	Q96N67	Dedicator of cytokinesis protein 7 OS=Homo sapiens OX=	49.66549972
High	Q12906	Interleukin enhancer-binding factor 3 OS=Homo sapiens	49.53323864
High	P55884	Eukaryotic translation initiation factor 3 subunit B OS=Hc	49.52136036
High	P11940	Polyadenylate-binding protein 1 OS=Homo sapiens OX=9	49.30751534
High	Q9HAV4	Exportin-5 OS=Homo sapiens OX=9606 GN=XPO5 PE=1 S	49.29475216
High	P46379	Large proline-rich protein BAG6 OS=Homo sapiens OX=9	48.77451108
High	Q562R1	Beta-actin-like protein 2 OS=Homo sapiens OX=9606 GN	48.7631356
High	Q92900	Regulator of nonsense transcripts 1 OS=Homo sapiens O	48.08690643
High	P50990	T-complex protein 1 subunit theta OS=Homo sapiens OX	48.06531072
High	O43175	D-3-phosphoglycerate dehydrogenase OS=Homo sapiens	47.90464335
High	P50570	Dynamin-2 OS=Homo sapiens OX=9606 GN=DNM2 PE=1	47.39481216
High	P19367	Hexokinase-1 OS=Homo sapiens OX=9606 GN=HK1 PE=1	47.17030207
High	P28340	DNA polymerase delta catalytic subunit OS=Homo sapier	46.26149651
High	P78347	General transcription factor II-I OS=Homo sapiens OX=96	46.25367976
High	Q9NVI1	Fanconi anemia group I protein OS=Homo sapiens OX=96	46.09247861
High	P13010	X-ray repair cross-complementing protein 5 OS=Homo sa	45.84816993
High	Q14683	Structural maintenance of chromosomes protein 1A OS=	45.61950919
High	P36578	60S ribosomal protein L4 OS=Homo sapiens OX=9606 GN	45.60926542
High	P05141	ADP/ATP translocase 2 OS=Homo sapiens OX=9606 GN=	45.14104091
High	P52789	Hexokinase-2 OS=Homo sapiens OX=9606 GN=HK2 PE=1	44.87224056
High	P11216	Glycogen phosphorylase, brain form OS=Homo sapiens C	44.80616755

High	O95202	Mitochondrial proton/calcium exchanger protein OS=Ho	44.14314236
High	P17812	CTP synthase 1 OS=Homo sapiens OX=9606 GN=CTPS1 P	44.06928042
High	P53396	ATP-citrate synthase OS=Homo sapiens OX=9606 GN=AC	43.74193314
High	Q9BXW7	Haloacid dehalogenase-like hydrolase domain-containing	43.52115958
High	Q05639	Elongation factor 1-alpha 2 OS=Homo sapiens OX=9606 (	43.20870214
High	Q9UNF1	Melanoma-associated antigen D2 OS=Homo sapiens OX=	43.04049087
High	O43390	Heterogeneous nuclear ribonucleoprotein R OS=Homo sa	43.03504488
High	P57737	Coronin-7 OS=Homo sapiens OX=9606 GN=CORO7 PE=1	42.75925001
High	Q9Y490	Talin-1 OS=Homo sapiens OX=9606 GN=TLN1 PE=1 SV=3	42.16181546
High	P17844	Probable ATP-dependent RNA helicase DDX5 OS=Homo s	41.90541269
High	Q8IXI1	Mitochondrial Rho GTPase 2 OS=Homo sapiens OX=9606	41.90271992
High	O94906	Pre-mRNA-processing factor 6 OS=Homo sapiens OX=960	41.72148452
High	Q15233	Non-POU domain-containing octamer-binding protein OS	41.70658084
High	Q92797	Symplekin OS=Homo sapiens OX=9606 GN=SYMPK PE=1	41.61377533
High	Q8N0X7	Spartin OS=Homo sapiens OX=9606 GN=SPART PE=1 SV=	41.40294094
High	Q9C0C9	(E3-independent) E2 ubiquitin-conjugating enzyme OS=H	41.25216066
High	P20700	Lamin-B1 OS=Homo sapiens OX=9606 GN=LMNB1 PE=1 S	41.02403627
High	P51991	Heterogeneous nuclear ribonucleoprotein A3 OS=Homo	40.98651736
High	P23921	Ribonucleoside-diphosphate reductase large subunit OS=	40.95538816
High	P12277	Creatine kinase B-type OS=Homo sapiens OX=9606 GN=(	40.82986084
High	Q5JTZ9	Alanine--tRNA ligase, mitochondrial OS=Homo sapiens O	40.76659405
High	Q15386	Ubiquitin-protein ligase E3C OS=Homo sapiens OX=9606	40.63048224
High	Q9BPX3	Condensin complex subunit 3 OS=Homo sapiens OX=960	40.57476513
High	P61204	ADP-ribosylation factor 3 OS=Homo sapiens OX=9606 GN	40.53207271
High	Q02809	Procollagen-lysine,2-oxoglutarate 5-dioxygenase 1 OS=H	40.2929819
High	P31939	Bifunctional purine biosynthesis protein PURH OS=Homo	39.8066703
High	O43143	Pre-mRNA-splicing factor ATP-dependent RNA helicase D	39.39402933
High	P54577	Tyrosine--tRNA ligase, cytoplasmic OS=Homo sapiens OX	39.22415514
High	Q06210	Glutamine--fructose-6-phosphate aminotransferase [isor	39.20845105
High	Q6UB35	Monofunctional C1-tetrahydrofolate synthase, mitochon	39.18462699
High	P08779	Keratin, type I cytoskeletal 16 OS=Homo sapiens OX=960	39.11238557
High	P00558	Phosphoglycerate kinase 1 OS=Homo sapiens OX=9606 G	39.09055962
High	Q92973	Transportin-1 OS=Homo sapiens OX=9606 GN=TNPO1 PE	38.77844753
High	P06576	ATP synthase subunit beta, mitochondrial OS=Homo sapi	38.76569014
High	P07195	L-lactate dehydrogenase B chain OS=Homo sapiens OX=9	38.71749339
High	O75534	Cold shock domain-containing protein E1 OS=Homo sapi	38.49941181
High	Q96GX5	Serine/threonine-protein kinase greatwall OS=Homo sap	38.44262861
High	P62820	Ras-related protein Rab-1A OS=Homo sapiens OX=9606 (	38.43410992
High	Q9NZI8	Insulin-like growth factor 2 mRNA-binding protein 1 OS=I	38.11994453
High	P30876	DNA-directed RNA polymerase II subunit RPB2 OS=Homo	38.03767168
High	Q15393	Splicing factor 3B subunit 3 OS=Homo sapiens OX=9606 (	37.83928842
High	P04844	Dolichyl-diphosphooligosaccharide--protein glycosyltrans	37.62731752
High	P45974	Ubiquitin carboxyl-terminal hydrolase 5 OS=Homo sapien	37.58260321
High	Q9H0D6	5'-3' exoribonuclease 2 OS=Homo sapiens OX=9606 GN=	37.53945673
High	P51617	Interleukin-1 receptor-associated kinase 1 OS=Homo sap	37.51393705
High	Q9BSJ8	Extended synaptotagmin-1 OS=Homo sapiens OX=9606 (	37.42610538
High	Q9BQ52	Zinc phosphodiesterase ELAC protein 2 OS=Homo sapien	37.294002

High	A0AVT1	Ubiquitin-like modifier-activating enzyme 6 OS=Homo sa	37.28053574
High	Q9BXP5	Serrate RNA effector molecule homolog OS=Homo sapie	37.20408311
High	Q14C86	GTPase-activating protein and VPS9 domain-containing p	37.0776214
High	P12956	X-ray repair cross-complementing protein 6 OS=Homo sa	36.71108509
High	O43707	Alpha-actinin-4 OS=Homo sapiens OX=9606 GN=ACTN4 P	36.38835753
High	O14654	Insulin receptor substrate 4 OS=Homo sapiens OX=9606	36.34488078
High	P51812	Ribosomal protein S6 kinase alpha-3 OS=Homo sapiens O	36.24017402
High	P18085	ADP-ribosylation factor 4 OS=Homo sapiens OX=9606 GN	36.04265854
High	O15397	Importin-8 OS=Homo sapiens OX=9606 GN=IPO8 PE=1 SV	35.97699948
High	P05388	60S acidic ribosomal protein P0 OS=Homo sapiens OX=96	35.82678691
High	Q14160	Protein scribble homolog OS=Homo sapiens OX=9606 GN	35.80922053
High	Q99798	Aconitate hydratase, mitochondrial OS=Homo sapiens OX	35.37500889
High	P06748	Nucleophosmin OS=Homo sapiens OX=9606 GN=NPM1 P	35.2253347
High	Q02878	60S ribosomal protein L6 OS=Homo sapiens OX=9606 GN	35.08512704
High	P36776	Lon protease homolog, mitochondrial OS=Homo sapiens	34.87593846
High	P08670	Vimentin OS=Homo sapiens OX=9606 GN=VIM PE=1 SV=	34.86493877
High	P00338	L-lactate dehydrogenase A chain OS=Homo sapiens OX=9	34.85588925
High	Q8WWM7	Ataxin-2-like protein OS=Homo sapiens OX=9606 GN=AT	34.83520341
High	P11498	Pyruvate carboxylase, mitochondrial OS=Homo sapiens O	34.76828728
High	P13647	Keratin, type II cytoskeletal 5 OS=Homo sapiens OX=960	34.70285615
High	Q9BTW9	Tubulin-specific chaperone D OS=Homo sapiens OX=960	34.65720685
High	P52888	Thimet oligopeptidase OS=Homo sapiens OX=9606 GN=T	34.59876017
High	O94966	Ubiquitin carboxyl-terminal hydrolase 19 OS=Homo sapie	34.57492323
High	P18206	Vinculin OS=Homo sapiens OX=9606 GN=VCL PE=1 SV=4	34.26981017
High	Q96PK6	RNA-binding protein 14 OS=Homo sapiens OX=9606 GN=	34.23146376
High	P23396	40S ribosomal protein S3 OS=Homo sapiens OX=9606 GN	34.06600427
High	Q99460	26S proteasome non-ATPase regulatory subunit 1 OS=Ho	33.99507945
High	Q15418	Ribosomal protein S6 kinase alpha-1 OS=Homo sapiens O	33.83606265
High	P84085	ADP-ribosylation factor 5 OS=Homo sapiens OX=9606 GN	33.83472992
High	P40227	T-complex protein 1 subunit zeta OS=Homo sapiens OX=	33.5452605
High	Q9NTJ3	Structural maintenance of chromosomes protein 4 OS=H	33.42830965
High	O43795	Unconventional myosin-Ib OS=Homo sapiens OX=9606 G	33.34009931
High	Q2NKX8	DNA excision repair protein ERCC-6-like OS=Homo sapier	33.2972545
High	Q9UBF2	Coatomer subunit gamma-2 OS=Homo sapiens OX=9606	33.22585853
High	Q2TAL8	Glutamine-rich protein 1 OS=Homo sapiens OX=9606 GN	32.81456448
High	Q8N1F7	Nuclear pore complex protein Nup93 OS=Homo sapiens	32.49908433
High	O95433	Activator of 90 kDa heat shock protein ATPase homolog	32.49601085
High	Q92621	Nuclear pore complex protein Nup205 OS=Homo sapiens	32.4286534
High	Q14126	Desmoglein-2 OS=Homo sapiens OX=9606 GN=DSG2 PE=	32.2248039
High	Q7Z5K2	Wings apart-like protein homolog OS=Homo sapiens OX=	31.84845901
High	P50991	T-complex protein 1 subunit delta OS=Homo sapiens OX=	31.45112176
High	Q9UHB9	Signal recognition particle subunit SRP68 OS=Homo sapie	31.32487733
High	P31943	Heterogeneous nuclear ribonucleoprotein H OS=Homo s	31.16877007
High	O94874	E3 UFM1-protein ligase 1 OS=Homo sapiens OX=9606 GN	31.09908151
High	O75155	Cullin-associated NEDD8-dissociated protein 2 OS=Homo	30.98119576
High	Q13435	Splicing factor 3B subunit 2 OS=Homo sapiens OX=9606 (	30.97404451
High	O95831	Apoptosis-inducing factor 1, mitochondrial OS=Homo sap	30.68782947

High	Q93008	Probable ubiquitin carboxyl-terminal hydrolase FAF-X OS	30.5234322
High	Q8TEQ6	Gem-associated protein 5 OS=Homo sapiens OX=9606 G	30.48094456
High	P38919	Eukaryotic initiation factor 4A-III OS=Homo sapiens OX=9	30.43486766
High	Q06830	Peroxiredoxin-1 OS=Homo sapiens OX=9606 GN=PRDX1	30.38628903
High	P38159	RNA-binding motif protein, X chromosome OS=Homo sap	30.2620956
High	P17858	ATP-dependent 6-phosphofructokinase, liver type OS=Ho	30.18644689
High	Q9NSD9	Phenylalanine--tRNA ligase beta subunit OS=Homo sapie	30.14335013
High	P33992	DNA replication licensing factor MCM5 OS=Homo sapien	29.99043958
High	P04259	Keratin, type II cytoskeletal 6B OS=Homo sapiens OX=96	29.97648789
High	P27695	DNA-(apurinic or apyrimidinic site) lyase OS=Homo sapie	29.94211871
High	P35232	Prohibitin OS=Homo sapiens OX=9606 GN=PHB PE=1 SV=	29.88944642
High	Q9UIA9	Exportin-7 OS=Homo sapiens OX=9606 GN=XPO7 PE=1 S	29.78263726
High	P16402	Histone H1.3 OS=Homo sapiens OX=9606 GN=HIST1H1D	29.70441755
High	Q9NR30	Nucleolar RNA helicase 2 OS=Homo sapiens OX=9606 GN	29.12457524
High	O75153	Clustered mitochondria protein homolog OS=Homo sapi	28.77409022
High	Q86Y56	Dynein assembly factor 5, axonemal OS=Homo sapiens O	28.66164263
High	Q9Y265	RuvB-like 1 OS=Homo sapiens OX=9606 GN=RUVBL1 PE=	28.40613991
High	Q9UI26	Importin-11 OS=Homo sapiens OX=9606 GN=IPO11 PE=1	28.21657828
High	Q9Y678	Coatomer subunit gamma-1 OS=Homo sapiens OX=9606	28.00375895
High	Q9H3U1	Protein unc-45 homolog A OS=Homo sapiens OX=9606 G	27.91426626
High	Q13148	TAR DNA-binding protein 43 OS=Homo sapiens OX=9606	27.53259003
High	P62826	GTP-binding nuclear protein Ran OS=Homo sapiens OX=9	27.52753179
High	Q05193	Dynamin-1 OS=Homo sapiens OX=9606 GN=DNM1 PE=1	27.52308312
High	P07197	Neurofilament medium polypeptide OS=Homo sapiens O	27.25395861
High	Q5VUA4	Zinc finger protein 318 OS=Homo sapiens OX=9606 GN=Z	27.19559878
High	Q8NE71	ATP-binding cassette sub-family F member 1 OS=Homo s	27.15949908
High	P18621	60S ribosomal protein L17 OS=Homo sapiens OX=9606 G	27.10546755
High	Q92667	A-kinase anchor protein 1, mitochondrial OS=Homo sapi	26.84091926
High	P13797	Plastin-3 OS=Homo sapiens OX=9606 GN=PLS3 PE=1 SV=	26.74829531
High	Q9H0U4	Ras-related protein Rab-1B OS=Homo sapiens OX=9606 G	26.49602283
High	O75152	Zinc finger CCCH domain-containing protein 11A OS=Hor	26.24654855
High	Q9BWF3	RNA-binding protein 4 OS=Homo sapiens OX=9606 GN=R	26.22186661
High	P51665	26S proteasome non-ATPase regulatory subunit 7 OS=Ho	26.15897176
High	O60566	Mitotic checkpoint serine/threonine-protein kinase BUB1	26.05273466
High	Q9ULT8	E3 ubiquitin-protein ligase HECTD1 OS=Homo sapiens OX	25.93116807
High	Q7L2E3	ATP-dependent RNA helicase DHX30 OS=Homo sapiens O	25.87723373
High	O76094	Signal recognition particle subunit SRP72 OS=Homo sapie	25.83791008
High	O00159	Unconventional myosin-Ic OS=Homo sapiens OX=9606 G	25.77596216
High	P51570	Galactokinase OS=Homo sapiens OX=9606 GN=GALK1 PE	25.6558615
High	P35606	Coatomer subunit beta' OS=Homo sapiens OX=9606 GN=	25.62384857
High	P46459	Vesicle-fusing ATPase OS=Homo sapiens OX=9606 GN=N	25.56827551
High	P09211	Glutathione S-transferase P OS=Homo sapiens OX=9606	25.56592051
High	P45880	Voltage-dependent anion-selective channel protein 2 OS	25.54808578
High	P54136	Arginine--tRNA ligase, cytoplasmic OS=Homo sapiens OX	25.49864042
High	Q9Y5L0	Transportin-3 OS=Homo sapiens OX=9606 GN=TNPO3 PE	25.35772566
High	P11177	Pyruvate dehydrogenase E1 component subunit beta, mi	25.28870935
High	Q9H3P7	Golgi resident protein GCP60 OS=Homo sapiens OX=9606	25.21726461

High	O60502	Protein O-GlcNAcase OS=Homo sapiens OX=9606 GN=O(	25.09061819
High	Q13310	Polyadenylate-binding protein 4 OS=Homo sapiens OX=9	24.98640615
High	P27635	60S ribosomal protein L10 OS=Homo sapiens OX=9606 G	24.93454347
High	Q8NBJ5	Procollagen galactosyltransferase 1 OS=Homo sapiens O	24.73551753
High	P23378	Glycine dehydrogenase (decarboxylating), mitochondrial	24.73111364
High	Q15181	Inorganic pyrophosphatase OS=Homo sapiens OX=9606 (	24.63286839
High	Q14204	Cytoplasmic dynein 1 heavy chain 1 OS=Homo sapiens O	24.43460835
High	P42285	Exosome RNA helicase MTR4 OS=Homo sapiens OX=960(	24.37892766
High	P62081	40S ribosomal protein S7 OS=Homo sapiens OX=9606 GN	24.31669725
High	P02545	Prelamin-A/C OS=Homo sapiens OX=9606 GN=LMNA PE=	24.21363876
High	Q13813	Spectrin alpha chain, non-erythrocytic 1 OS=Homo sapie	24.16089445
High	Q5T9A4	ATPase family AAA domain-containing protein 3B OS=Ho	23.86512077
High	Q58FG1	Putative heat shock protein HSP 90-alpha A4 OS=Homo s	23.85224903
High	Q9UQ16	Dynamin-3 OS=Homo sapiens OX=9606 GN=DNM3 PE=1	23.75803166
High	O60313	Dynamin-like 120 kDa protein, mitochondrial OS=Homo s	23.67603296
High	P51148	Ras-related protein Rab-5C OS=Homo sapiens OX=9606 (	23.67579451
High	Q09161	Nuclear cap-binding protein subunit 1 OS=Homo sapiens	23.61409092
High	Q96RP9	Elongation factor G, mitochondrial OS=Homo sapiens OX	23.59525556
High	P05787	Keratin, type II cytoskeletal 8 OS=Homo sapiens OX=960(	23.52946692
High	Q9NQW7	Xaa-Pro aminopeptidase 1 OS=Homo sapiens OX=9606 G	23.29098446
High	Q99623	Prohibitin-2 OS=Homo sapiens OX=9606 GN=PHB2 PE=1	23.21752633
High	Q3ZCQ8	Mitochondrial import inner membrane translocase subu	23.05200198
High	O00469	Procollagen-lysine,2-oxoglutarate 5-dioxygenase 2 OS=H	22.91718407
High	Q9Y230	RuvB-like 2 OS=Homo sapiens OX=9606 GN=RUVBL2 PE=	22.76465914
High	Q96T76	MMS19 nucleotide excision repair protein homolog OS=I	22.63225597
High	O95163	Elongator complex protein 1 OS=Homo sapiens OX=9606	22.59118877
High	Q92598	Heat shock protein 105 kDa OS=Homo sapiens OX=9606	22.49902954
High	P13667	Protein disulfide-isomerase A4 OS=Homo sapiens OX=96	22.43846597
High	Q9UHD1	Cysteine and histidine-rich domain-containing protein 1 (	22.29011753
High	O43684	Mitotic checkpoint protein BUB3 OS=Homo sapiens OX=9	21.98626921
High	Q96KR1	Zinc finger RNA-binding protein OS=Homo sapiens OX=96	21.76963936
High	O95573	Long-chain-fatty-acid--CoA ligase 3 OS=Homo sapiens OX	21.66710379
High	P15170	Eukaryotic peptide chain release factor GTP-binding subu	21.51096947
High	P48735	Isocitrate dehydrogenase [NADP], mitochondrial OS=Hor	21.47483935
High	P52597	Heterogeneous nuclear ribonucleoprotein F OS=Homo sa	21.46383031
High	P11310	Medium-chain specific acyl-CoA dehydrogenase, mitocho	21.35694991
High	P62979	Ubiquitin-40S ribosomal protein S27a OS=Homo sapiens	21.29094409
High	Q9BW92	Threonine--tRNA ligase, mitochondrial OS=Homo sapiens	21.28497866
High	Q9NVI7	ATPase family AAA domain-containing protein 3A OS=Ho	21.2373471
High	Q9UG63	ATP-binding cassette sub-family F member 2 OS=Homo s	21.21623742
High	Q27J81	Inverted formin-2 OS=Homo sapiens OX=9606 GN=INF2 I	21.08450938
High	P47985	Cytochrome b-c1 complex subunit Rieske, mitochondrial	21.06915476
High	Q9H9B4	Sideroflexin-1 OS=Homo sapiens OX=9606 GN=SFXN1 PE	21.05426455
High	Q13838	Spliceosome RNA helicase DDX39B OS=Homo sapiens OX	20.91676497
High	Q15031	Probable leucine--tRNA ligase, mitochondrial OS=Homo s	20.88124959
High	P57678	Gem-associated protein 4 OS=Homo sapiens OX=9606 G	20.84204019
High	Q10567	AP-1 complex subunit beta-1 OS=Homo sapiens OX=960(	20.78813027

High	Q9BXJ9	N-alpha-acetyltransferase 15, NatA auxiliary subunit OS=	20.65601808
High	O43837	Isocitrate dehydrogenase [NAD] subunit beta, mitochonc	20.5369611
High	Q9Y613	FH1/FH2 domain-containing protein 1 OS=Homo sapiens	20.49073634
High	P23528	Cofilin-1 OS=Homo sapiens OX=9606 GN=CFL1 PE=1 SV=:	20.43625214
High	P61026	Ras-related protein Rab-10 OS=Homo sapiens OX=9606 (	20.31284144
High	P51149	Ras-related protein Rab-7a OS=Homo sapiens OX=9606 (	20.12095388
High	O14974	Protein phosphatase 1 regulatory subunit 12A OS=Homo	20.0874327
High	Q8N766	ER membrane protein complex subunit 1 OS=Homo sapi(	20.04006674
High	Q8TEU7	Rap guanine nucleotide exchange factor 6 OS=Homo sap	19.78697582
High	Q71RC2	La-related protein 4 OS=Homo sapiens OX=9606 GN=LAF	19.49872357
High	Q14669	E3 ubiquitin-protein ligase TRIP12 OS=Homo sapiens OX=	19.43582116
High	P46782	40S ribosomal protein S5 OS=Homo sapiens OX=9606 GN	19.35550758
High	Q96A65	Exocyst complex component 4 OS=Homo sapiens OX=96	19.28511985
High	P22695	Cytochrome b-c1 complex subunit 2, mitochondrial OS=H	19.22619042
High	O43592	Exportin-T OS=Homo sapiens OX=9606 GN=XPOT PE=1 S\	19.22372085
High	Q9H583	HEAT repeat-containing protein 1 OS=Homo sapiens OX=	19.19950634
High	P63104	14-3-3 protein zeta/delta OS=Homo sapiens OX=9606 GN	19.11042796
High	Q15020	Squamous cell carcinoma antigen recognized by T-cells 3	19.06587207
High	Q04760	Lactoylglutathione lyase OS=Homo sapiens OX=9606 GN=	19.05958351
High	O14828	Secretory carrier-associated membrane protein 3 OS=Ho	19.00214523
High	Q8TD19	Serine/threonine-protein kinase Nek9 OS=Homo sapiens	18.97826432
High	P30153	Serine/threonine-protein phosphatase 2A 65 kDa regulat	18.8964145
High	P32969	60S ribosomal protein L9 OS=Homo sapiens OX=9606 GN	18.75061749
High	Q01813	ATP-dependent 6-phosphofructokinase, platelet type OS	18.74832064
High	Q9Y4W6	AFG3-like protein 2 OS=Homo sapiens OX=9606 GN=AFG	18.61770644
High	Q14997	Proteasome activator complex subunit 4 OS=Homo sapie	18.58370652
High	O00411	DNA-directed RNA polymerase, mitochondrial OS=Homo	18.57238885
High	Q8IW35	Centrosomal protein of 97 kDa OS=Homo sapiens OX=96	18.54603994
High	Q8IXI2	Mitochondrial Rho GTPase 1 OS=Homo sapiens OX=9606	18.51539377
High	P02768	Serum albumin OS=Homo sapiens OX=9606 GN=ALB PE=	18.47218406
High	O94826	Mitochondrial import receptor subunit TOM70 OS=Hom	18.44192085
High	Q12756	Kinesin-like protein KIF1A OS=Homo sapiens OX=9606 GI	18.43206166
High	P52292	Importin subunit alpha-1 OS=Homo sapiens OX=9606 GN	18.4102441
High	Q9BTV4	Transmembrane protein 43 OS=Homo sapiens OX=9606	18.40204948
High	P20340	Ras-related protein Rab-6A OS=Homo sapiens OX=9606 (	18.38904162
High	P50213	Isocitrate dehydrogenase [NAD] subunit alpha, mitochon	18.35923535
High	Q66K74	Microtubule-associated protein 1S OS=Homo sapiens OX	18.34357269
High	Q9NTI5	Sister chromatid cohesion protein PDS5 homolog B OS=H	18.30768467
High	Q8WVB6	Chromosome transmission fidelity protein 18 homolog C	18.27824202
High	P62701	40S ribosomal protein S4, X isoform OS=Homo sapiens O	18.27522943
High	P62269	40S ribosomal protein S18 OS=Homo sapiens OX=9606 G	18.23922635
High	P08133	Annexin A6 OS=Homo sapiens OX=9606 GN=ANXA6 PE=1	18.21256264
High	Q15459	Splicing factor 3A subunit 1 OS=Homo sapiens OX=9606 (	18.18228819
High	O60506	Heterogeneous nuclear ribonucleoprotein Q OS=Homo s	18.09731975
High	P13804	Electron transfer flavoprotein subunit alpha, mitochondr	18.09538397
High	Q99832	T-complex protein 1 subunit eta OS=Homo sapiens OX=9	18.09318571
High	P60891	Ribose-phosphate pyrophosphokinase 1 OS=Homo sapie	18.05803303

High	P49790	Nuclear pore complex protein Nup153 OS=Homo sapiens	18.01760569
High	P26038	Moesin OS=Homo sapiens OX=9606 GN=MSN PE=1 SV=3	17.87144594
High	Q15758	Neutral amino acid transporter B(0) OS=Homo sapiens O	17.85574494
High	Q6PKG0	La-related protein 1 OS=Homo sapiens OX=9606 GN=LAR	17.82125499
High	Q92888	Rho guanine nucleotide exchange factor 1 OS=Homo sap	17.75489091
High	P14324	Farnesyl pyrophosphate synthase OS=Homo sapiens OX=	17.57957315
High	P61106	Ras-related protein Rab-14 OS=Homo sapiens OX=9606 (	17.5684204
High	Q7Z460	CLIP-associating protein 1 OS=Homo sapiens OX=9606 G	17.47076578
High	Q13347	Eukaryotic translation initiation factor 3 subunit I OS=Ho	17.35303738
High	P07910	Heterogeneous nuclear ribonucleoproteins C1/C2 OS=Ho	17.25992253
High	O00154	Cytosolic acyl coenzyme A thioester hydrolase OS=Homo	17.22344929
High	Q99504	Eyes absent homolog 3 OS=Homo sapiens OX=9606 GN=	17.21151354
High	Q14671	Pumilio homolog 1 OS=Homo sapiens OX=9606 GN=PUM	17.13358188
High	P42166	Lamina-associated polypeptide 2, isoform alpha OS=Hom	17.03249524
High	P62805	Histone H4 OS=Homo sapiens OX=9606 GN=HIST1H4A PI	16.99853853
High	Q99873	Protein arginine N-methyltransferase 1 OS=Homo sapien	16.82625996
High	P15311	Ezrin OS=Homo sapiens OX=9606 GN=EZR PE=1 SV=4	16.80996082
High	Q99497	Protein/nucleic acid deglycase DJ-1 OS=Homo sapiens O)	16.80918372
High	P49591	Serine--tRNA ligase, cytoplasmic OS=Homo sapiens OX=9	16.71101761
High	O43156	TELO2-interacting protein 1 homolog OS=Homo sapiens (	16.71000755
High	Q96SB4	SRSF protein kinase 1 OS=Homo sapiens OX=9606 GN=SF	16.66701166
High	Q32P28	Prolyl 3-hydroxylase 1 OS=Homo sapiens OX=9606 GN=P	16.61215385
High	Q16531	DNA damage-binding protein 1 OS=Homo sapiens OX=96	16.6086069
High	Q92615	La-related protein 4B OS=Homo sapiens OX=9606 GN=LA	16.49794658
High	P34897	Serine hydroxymethyltransferase, mitochondrial OS=Hor	16.45572374
High	P49755	Transmembrane emp24 domain-containing protein 10 O	16.4323008
High	P06493	Cyclin-dependent kinase 1 OS=Homo sapiens OX=9606 G	16.42277142
High	O60341	Lysine-specific histone demethylase 1A OS=Homo sapien	16.2694421
High	P38606	V-type proton ATPase catalytic subunit A OS=Homo sapie	16.10917846
High	Q9H2M9	Rab3 GTPase-activating protein non-catalytic subunit OS	16.10415497
High	P31930	Cytochrome b-c1 complex subunit 1, mitochondrial OS=H	16.09449581
High	P39023	60S ribosomal protein L3 OS=Homo sapiens OX=9606 GN	15.96512585
High	P27816	Microtubule-associated protein 4 OS=Homo sapiens OX=	15.94644689
High	Q8IYD1	Eukaryotic peptide chain release factor GTP-binding subu	15.92722616
High	P14868	Aspartate--tRNA ligase, cytoplasmic OS=Homo sapiens O	15.87956931
High	Q15003	Condensin complex subunit 2 OS=Homo sapiens OX=960	15.87243145
High	Q9BQ39	ATP-dependent RNA helicase DDX50 OS=Homo sapiens (	15.86382719
High	P26599	Polypyrimidine tract-binding protein 1 OS=Homo sapiens	15.69853282
High	Q15046	Lysine--tRNA ligase OS=Homo sapiens OX=9606 GN=KAR	15.68761468
High	O60610	Protein diaphanous homolog 1 OS=Homo sapiens OX=96	15.65657765
High	P31040	Succinate dehydrogenase [ubiquinone] flavoprotein subu	15.5999288
High	P61224	Ras-related protein Rap-1b OS=Homo sapiens OX=9606 (	15.54225006
High	Q8NBF2	NHL repeat-containing protein 2 OS=Homo sapiens OX=9	15.47801989
High	P36873	Serine/threonine-protein phosphatase PP1-gamma catal	15.46370047
High	P62136	Serine/threonine-protein phosphatase PP1-alpha catalyt	15.4182732
High	P63010	AP-2 complex subunit beta OS=Homo sapiens OX=9606 (	15.41773571
High	P42345	Serine/threonine-protein kinase mTOR OS=Homo sapien	15.37939671

High	P12814	Alpha-actinin-1 OS=Homo sapiens OX=9606 GN=ACTN1 F	15.34075874
High	Q15042	Rab3 GTPase-activating protein catalytic subunit OS=Hor	15.33383434
High	Q7L576	Cytoplasmic FMR1-interacting protein 1 OS=Homo sapier	15.26865725
High	Q86UP2	Kinectin OS=Homo sapiens OX=9606 GN=KTN1 PE=1 SV=	15.10908972
High	P10586	Receptor-type tyrosine-protein phosphatase F OS=Homo	14.98741093
High	Q7Z2W4	Zinc finger CCCH-type antiviral protein 1 OS=Homo sapie	14.82183357
High	Q7Z2T5	TRMT1-like protein OS=Homo sapiens OX=9606 GN=TRM	14.80841317
High	Q2NL82	Pre-rRNA-processing protein TSR1 homolog OS=Homo sa	14.77134219
High	P61221	ATP-binding cassette sub-family E member 1 OS=Homo s	14.66828759
High	P55084	Trifunctional enzyme subunit beta, mitochondrial OS=Ho	14.66232444
High	Q07020	60S ribosomal protein L18 OS=Homo sapiens OX=9606 G	14.66144552
High	Q00325	Phosphate carrier protein, mitochondrial OS=Homo sapie	14.64951953
High	P39748	Flap endonuclease 1 OS=Homo sapiens OX=9606 GN=FEI	14.64313647
High	P40938	Replication factor C subunit 3 OS=Homo sapiens OX=960	14.5831245
High	O43242	26S proteasome non-ATPase regulatory subunit 3 OS=Ho	14.47510165
High	P62333	26S proteasome regulatory subunit 10B OS=Homo sapier	14.46421796
High	Q7L014	Probable ATP-dependent RNA helicase DDX46 OS=Homo	14.46320998
High	Q02978	Mitochondrial 2-oxoglutarate/malate carrier protein OS=	14.45781446
High	P62277	40S ribosomal protein S13 OS=Homo sapiens OX=9606 G	14.4304064
High	Q15907	Ras-related protein Rab-11B OS=Homo sapiens OX=9606	14.39758476
High	P49321	Nuclear autoantigenic sperm protein OS=Homo sapiens (	14.22989848
High	P12532	Creatine kinase U-type, mitochondrial OS=Homo sapiens	14.20019004
High	Q9Y3Z3	Deoxynucleoside triphosphate triphosphohydrolase SAM	14.17101206
High	P30041	Peroxiredoxin-6 OS=Homo sapiens OX=9606 GN=PRDX6	14.07701211
High	Q9ULX6	A-kinase anchor protein 8-like OS=Homo sapiens OX=960	14.06331641
High	O95757	Heat shock 70 kDa protein 4L OS=Homo sapiens OX=960	14.01854213
High	P53985	Monocarboxylate transporter 1 OS=Homo sapiens OX=96	13.99075976
High	P48147	Prolyl endopeptidase OS=Homo sapiens OX=9606 GN=PF	13.97006267
High	O75122	CLIP-associating protein 2 OS=Homo sapiens OX=9606 G	13.83769885
High	Q9HB07	UPF0160 protein MYG1, mitochondrial OS=Homo sapien	13.83116123
High	Q9Y6D9	Mitotic spindle assembly checkpoint protein MAD1 OS=H	13.77983809
High	Q96I24	Far upstream element-binding protein 3 OS=Homo sapie	13.73415907
High	O75477	Erlin-1 OS=Homo sapiens OX=9606 GN=ERLIN1 PE=1 SV=	13.62571113
High	Q14558	Phosphoribosyl pyrophosphate synthase-associated prot	13.53333255
High	Q9H3S7	Tyrosine-protein phosphatase non-receptor type 23 OS=	13.49893171
High	P10768	S-formylglutathione hydrolase OS=Homo sapiens OX=96	13.39105067
High	B5ME19	Eukaryotic translation initiation factor 3 subunit C-like pr	13.37951857
High	P07741	Adenine phosphoribosyltransferase OS=Homo sapiens O	13.32124454
High	P20020	Plasma membrane calcium-transporting ATPase 1 OS=Ho	13.1356842
High	A0A075B6S	Immunoglobulin kappa variable 2D-29 OS=Homo sapiens	13.10233218
High	O15084	Serine/threonine-protein phosphatase 6 regulatory anky	13.08449104
High	O14981	TATA-binding protein-associated factor 172 OS=Homo sa	13.05703962
High	O60814	Histone H2B type 1-K OS=Homo sapiens OX=9606 GN=HI	13.0256319
High	O14744	Protein arginine N-methyltransferase 5 OS=Homo sapien	12.99963182
High	P42566	Epidermal growth factor receptor substrate 15 OS=Homo	12.95406182
High	Q9NUQ8	ATP-binding cassette sub-family F member 3 OS=Homo s	12.95346561
High	P38117	Electron transfer flavoprotein subunit beta OS=Homo sa	12.9264991

High	O00425	Insulin-like growth factor 2 mRNA-binding protein 3 OS=I	12.88161299
High	Q8IZL8	Proline-, glutamic acid- and leucine-rich protein 1 OS=Ho	12.8410432
High	Q8TAT6	Nuclear protein localization protein 4 homolog OS=Homo	12.73944804
High	P61981	14-3-3 protein gamma OS=Homo sapiens OX=9606 GN=Y	12.73570406
High	A0A1B0GTU	Zinc finger CCCH domain-containing protein 11B OS=Hon	12.71896819
High	Q96ST3	Paired amphipathic helix protein Sin3a OS=Homo sapiens	12.69126306
High	Q99829	Copine-1 OS=Homo sapiens OX=9606 GN=CPNE1 PE=1 SV	12.68688375
High	P31946	14-3-3 protein beta/alpha OS=Homo sapiens OX=9606 G	12.67994775
High	Q9Y4C2	TRPM8 channel-associated factor 1 OS=Homo sapiens OX	12.67896453
High	P29401	Transketolase OS=Homo sapiens OX=9606 GN=TKT PE=1	12.67464881
High	Q9UQ80	Proliferation-associated protein 2G4 OS=Homo sapiens C	12.66860122
High	O43776	Asparagine--tRNA ligase, cytoplasmic OS=Homo sapiens (	12.65137879
High	P06730	Eukaryotic translation initiation factor 4E OS=Homo sapie	12.62051384
High	Q9C0C7	Activating molecule in BECN1-regulated autophagy prote	12.58251711
High	P12004	Proliferating cell nuclear antigen OS=Homo sapiens OX=9	12.56535246
High	O43719	HIV Tat-specific factor 1 OS=Homo sapiens OX=9606 GN=	12.54960816
High	Q12905	Interleukin enhancer-binding factor 2 OS=Homo sapiens	12.51876811
High	P46783	40S ribosomal protein S10 OS=Homo sapiens OX=9606 G	12.50967323
High	Q5JPE7	Nodal modulator 2 OS=Homo sapiens OX=9606 GN=NOM	12.47391774
High	P61006	Ras-related protein Rab-8A OS=Homo sapiens OX=9606 (	12.46952741
High	Q9NRZ9	Lymphoid-specific helicase OS=Homo sapiens OX=9606 G	12.46815675
High	O43264	Centromere/kinetochore protein zw10 homolog OS=Hor	12.46368521
High	Q9Y5P6	Mannose-1-phosphate guanyltransferase beta OS=Homo	12.45426807
High	Q5H9R7	Serine/threonine-protein phosphatase 6 regulatory subu	12.37674987
High	P05198	Eukaryotic translation initiation factor 2 subunit 1 OS=Ho	12.34512126
High	O75131	Copine-3 OS=Homo sapiens OX=9606 GN=CPNE3 PE=1 SV	12.30724787
High	Q9UBB4	Ataxin-10 OS=Homo sapiens OX=9606 GN=ATXN10 PE=1	12.29855564
High	Q9BWH6	RNA polymerase II-associated protein 1 OS=Homo sapier	12.29637681
High	Q9NT62	Ubiquitin-like-conjugating enzyme ATG3 OS=Homo sapie	12.19952003
High	P00403	Cytochrome c oxidase subunit 2 OS=Homo sapiens OX=9	12.16559272
High	P10253	Lysosomal alpha-glucosidase OS=Homo sapiens OX=9606	12.15497077
High	Q02543	60S ribosomal protein L18a OS=Homo sapiens OX=9606	12.08355526
High	P31942	Heterogeneous nuclear ribonucleoprotein H3 OS=Homo	12.0800356
High	Q13155	Aminoacyl tRNA synthase complex-interacting multifunc	12.07049917
High	Q14139	Ubiquitin conjugation factor E4 A OS=Homo sapiens OX=	12.06555303
High	P14923	Junction plakoglobin OS=Homo sapiens OX=9606 GN=JU	12.03014522
High	Q14839	Chromodomain-helicase-DNA-binding protein 4 OS=Hom	12.02340772
High	Q9NS69	Mitochondrial import receptor subunit TOM22 homolog	11.9667196
High	Q9Y2J2	Band 4.1-like protein 3 OS=Homo sapiens OX=9606 GN=E	11.92723724
High	Q9Y450	HBS1-like protein OS=Homo sapiens OX=9606 GN=HBS1L	11.77438853
High	Q9BXW6	Oxysterol-binding protein-related protein 1 OS=Homo sa	11.67503879
High	Q9Y4E8	Ubiquitin carboxyl-terminal hydrolase 15 OS=Homo sapie	11.6402273
High	Q7LBC6	Lysine-specific demethylase 3B OS=Homo sapiens OX=96	11.60770094
High	Q99700	Ataxin-2 OS=Homo sapiens OX=9606 GN=ATXN2 PE=1 SV	11.5090235
High	Q9UHD8	Septin-9 OS=Homo sapiens OX=9606 GN=SEPT9 PE=1 SV=	11.37509136
High	Q13045	Protein flightless-1 homolog OS=Homo sapiens OX=9606	11.36402078
High	Q6PI48	Aspartate--tRNA ligase, mitochondrial OS=Homo sapiens	11.31697233

High	P07355	Annexin A2 OS=Homo sapiens OX=9606 GN=ANXA2 PE=1	11.28259017
High	Q04323	UBX domain-containing protein 1 OS=Homo sapiens OX=	11.17826728
High	Q96RG2	PAS domain-containing serine/threonine-protein kinase (	11.17145534
High	Q1KMD3	Heterogeneous nuclear ribonucleoprotein U-like protein	11.16477294
High	Q16576	Histone-binding protein RBBP7 OS=Homo sapiens OX=96	11.15409609
High	Q9NY33	Dipeptidyl peptidase 3 OS=Homo sapiens OX=9606 GN=D	11.13927814
High	P68400	Casein kinase II subunit alpha OS=Homo sapiens OX=960	11.02258133
High	Q8NG31	Kinetochore scaffold 1 OS=Homo sapiens OX=9606 GN=K	10.97186859
High	Q8NI27	THO complex subunit 2 OS=Homo sapiens OX=9606 GN=	10.94556344
High	Q14739	Lamin-B receptor OS=Homo sapiens OX=9606 GN=LBR PI	10.93683435
High	P35573	Glycogen debranching enzyme OS=Homo sapiens OX=96	10.926902
High	O43252	Bifunctional 3'-phosphoadenosine 5'-phosphosulfate syn	10.92237768
High	Q99956	Dual specificity protein phosphatase 9 OS=Homo sapiens	10.9214599
High	Q13409	Cytoplasmic dynein 1 intermediate chain 2 OS=Homo sap	10.9050651
High	Q9Y2A7	Nck-associated protein 1 OS=Homo sapiens OX=9606 GN	10.86134862
High	Q13576	Ras GTPase-activating-like protein IQGAP2 OS=Homo sap	10.83177103
High	P62241	40S ribosomal protein S8 OS=Homo sapiens OX=9606 GN	10.82466968
High	Q96CW5	Gamma-tubulin complex component 3 OS=Homo sapien	10.75395538
High	Q6Y7W6	GRB10-interacting GYF protein 2 OS=Homo sapiens OX=9	10.72043964
High	Q9NPI6	mRNA-decapping enzyme 1A OS=Homo sapiens OX=960(	10.71215033
High	O75955	Flotillin-1 OS=Homo sapiens OX=9606 GN=FLOT1 PE=1 SV	10.6343419
High	O75306	NADH dehydrogenase [ubiquinone] iron-sulfur protein 2,	10.59021506
High	P48047	ATP synthase subunit O, mitochondrial OS=Homo sapien	10.54899815
High	P00387	NADH-cytochrome b5 reductase 3 OS=Homo sapiens OX=	10.51753376
High	Q96AG4	Leucine-rich repeat-containing protein 59 OS=Homo sapi	10.43792581
High	O43290	U4/U6.U5 tri-snRNP-associated protein 1 OS=Homo sapi	10.43383
High	O75179	Ankyrin repeat domain-containing protein 17 OS=Homo	10.405629
High	Q96HY7	Probable 2-oxoglutarate dehydrogenase E1 component I	10.40104677
High	Q8WUM0	Nuclear pore complex protein Nup133 OS=Homo sapiens	10.38625005
High	P54709	Sodium/potassium-transporting ATPase subunit beta-3 C	10.38552322
High	Q07666	KH domain-containing, RNA-binding, signal transduction-	10.38034989
High	P62714	Serine/threonine-protein phosphatase 2A catalytic subur	10.33601837
High	P24534	Elongation factor 1-beta OS=Homo sapiens OX=9606 GN	10.32150028
High	Q96EK7	Constitutive coactivator of peroxisome proliferator-activ	10.30009633
High	P29992	Guanine nucleotide-binding protein subunit alpha-11 OS=	10.28029798
High	O75874	Isocitrate dehydrogenase [NADP] cytoplasmic OS=Homo	10.27830455
High	Q15738	Sterol-4-alpha-carboxylate 3-dehydrogenase, decarboxyl	10.23170767
High	P84103	Serine/arginine-rich splicing factor 3 OS=Homo sapiens C	10.21697357
High	O95671	N-acetylserotonin O-methyltransferase-like protein OS=H	10.205654
High	P84098	60S ribosomal protein L19 OS=Homo sapiens OX=9606 G	10.16031786
High	P50750	Cyclin-dependent kinase 9 OS=Homo sapiens OX=9606 G	10.13904362
High	P14735	Insulin-degrading enzyme OS=Homo sapiens OX=9606 GI	10.1381549
High	P46087	Probable 28S rRNA (cytosine(4447)-C(5))-methyltransfer	10.06302309
High	P55196	Afadin OS=Homo sapiens OX=9606 GN=AFDN PE=1 SV=3	10.05620783
High	Q96FW1	Ubiquitin thioesterase OTUB1 OS=Homo sapiens OX=960	10.04203389
High	O60343	TBC1 domain family member 4 OS=Homo sapiens OX=96	10.03796421
High	Q7Z4H7	HAUS augmin-like complex subunit 6 OS=Homo sapiens (	10.03011821

High	Q9Y2D5	A-kinase anchor protein 2 OS=Homo sapiens OX=9606 G	10.00648664
High	P83731	60S ribosomal protein L24 OS=Homo sapiens OX=9606 G	9.995950086
High	P62750	60S ribosomal protein L23a OS=Homo sapiens OX=9606	9.968050001
High	P62899	60S ribosomal protein L31 OS=Homo sapiens OX=9606 G	9.923918308
High	Q9UNM6	26S proteasome non-ATPase regulatory subunit 13 OS=H	9.899040184
High	P62258	14-3-3 protein epsilon OS=Homo sapiens OX=9606 GN=Y	9.855240236
High	Q9BSD7	Cancer-related nucleoside-triphosphatase OS=Homo sap	9.849411183
High	Q13724	Mannosyl-oligosaccharide glucosidase OS=Homo sapiens	9.847753711
High	Q7Z5L2	Coiled-coil domain-containing protein R3HCC1L OS=Hom	9.845576027
High	O95239	Chromosome-associated kinesin KIF4A OS=Homo sapien	9.786046412
High	Q9NVE7	Pantothenate kinase 4 OS=Homo sapiens OX=9606 GN=F	9.749937545
High	O60547	GDP-mannose 4,6 dehydratase OS=Homo sapiens OX=96	9.720085273
High	P34949	Mannose-6-phosphate isomerase OS=Homo sapiens OX=	9.697589407
High	Q13596	Sorting nexin-1 OS=Homo sapiens OX=9606 GN=SNX1 PE	9.655858192
High	Q13151	Heterogeneous nuclear ribonucleoprotein A0 OS=Homo	9.633832091
High	O14802	DNA-directed RNA polymerase III subunit RPC1 OS=Hom	9.61019693
High	Q8N3U4	Cohesin subunit SA-2 OS=Homo sapiens OX=9606 GN=ST	9.593686194
High	Q9BQA1	Methylosome protein 50 OS=Homo sapiens OX=9606 GN	9.58990334
High	O75821	Eukaryotic translation initiation factor 3 subunit G OS=Ho	9.575403866
High	Q14157	Ubiquitin-associated protein 2-like OS=Homo sapiens OX	9.559804651
High	Q9UJS0	Calcium-binding mitochondrial carrier protein Aralar2 OS	9.557231842
High	Q9NRN7	L-aminoadipate-semialdehyde dehydrogenase-phosphop	9.540733054
High	P62829	60S ribosomal protein L23 OS=Homo sapiens OX=9606 G	9.451651863
High	Q9HC07	Transmembrane protein 165 OS=Homo sapiens OX=9606	9.43944579
High	P21127	Cyclin-dependent kinase 11B OS=Homo sapiens OX=9606	9.352877854
High	P56134	ATP synthase subunit f, mitochondrial OS=Homo sapiens	9.314435687
High	O00264	Membrane-associated progesterone receptor componer	9.312036286
High	P17980	26S proteasome regulatory subunit 6A OS=Homo sapiens	9.302132862
High	P60174	Triosephosphate isomerase OS=Homo sapiens OX=9606	9.291517798
High	Q96EP5	DAZ-associated protein 1 OS=Homo sapiens OX=9606 GN	9.276070065
High	Q13617	Cullin-2 OS=Homo sapiens OX=9606 GN=CUL2 PE=1 SV=2	9.253068096
High	Q9UKG1	DCC-interacting protein 13-alpha OS=Homo sapiens OX=9	9.214902624
High	Q9UBT2	SUMO-activating enzyme subunit 2 OS=Homo sapiens OX	9.203554379
High	O14979	Heterogeneous nuclear ribonucleoprotein D-like OS=Hor	9.199425987
High	P30050	60S ribosomal protein L12 OS=Homo sapiens OX=9606 G	9.137957636
High	P51659	Peroxisomal multifunctional enzyme type 2 OS=Homo sa	9.100362443
High	O60841	Eukaryotic translation initiation factor 5B OS=Homo sapi	9.077514634
High	P36871	Phosphoglucomutase-1 OS=Homo sapiens OX=9606 GN=	9.009452862
High	Q96T37	RNA-binding protein 15 OS=Homo sapiens OX=9606 GN=	8.992303344
High	Q13162	Peroxiredoxin-4 OS=Homo sapiens OX=9606 GN=PRDX4	8.974532643
High	P35241	Radixin OS=Homo sapiens OX=9606 GN=RDX PE=1 SV=1	8.971029821
High	P25786	Proteasome subunit alpha type-1 OS=Homo sapiens OX=	8.951760849
High	Q86WJ1	Chromodomain-helicase-DNA-binding protein 1-like OS=	8.944152454
High	P25325	3-mercaptopyruvate sulfurtransferase OS=Homo sapiens	8.910833287
High	Q9H6E5	Speckle targeted PIP5K1A-regulated poly(A) polymerase	8.883666964
High	Q92878	DNA repair protein RAD50 OS=Homo sapiens OX=9606 G	8.883441844
High	Q9C0C2	182 kDa tankyrase-1-binding protein OS=Homo sapiens C	8.883311481

High	P50402	Emerin OS=Homo sapiens OX=9606 GN=EMD PE=1 SV=1	8.822130844
High	O60488	Long-chain-fatty-acid--CoA ligase 4 OS=Homo sapiens OX	8.775447417
High	Q14103	Heterogeneous nuclear ribonucleoprotein D0 OS=Homo	8.773705791
High	Q5VTR2	E3 ubiquitin-protein ligase BRE1A OS=Homo sapiens OX=	8.7446749
High	Q5D862	Filaggrin-2 OS=Homo sapiens OX=9606 GN=FLG2 PE=1 SV	8.744133781
High	P0DN79	Cystathionine beta-synthase-like protein OS=Homo sapie	8.742095271
High	Q8IWZ3	Ankyrin repeat and KH domain-containing protein 1 OS=I	8.73184456
High	Q9UKF6	Cleavage and polyadenylation specificity factor subunit 3	8.7202538
High	Q92889	DNA repair endonuclease XPF OS=Homo sapiens OX=960	8.690652216
High	Q7Z2Z2	Elongation factor-like GTPase 1 OS=Homo sapiens OX=96	8.684204544
High	Q96FC9	ATP-dependent DNA helicase DDX11 OS=Homo sapiens (	8.641734232
High	Q68EM7	Rho GTPase-activating protein 17 OS=Homo sapiens OX=	8.641611124
High	Q6P2E9	Enhancer of mRNA-decapping protein 4 OS=Homo sapier	8.64021426
High	O75146	Huntingtin-interacting protein 1-related protein OS=Hom	8.625735695
High	Q07955	Serine/arginine-rich splicing factor 1 OS=Homo sapiens C	8.597065305
High	P11233	Ras-related protein Ral-A OS=Homo sapiens OX=9606 GN	8.597000431
High	O15479	Melanoma-associated antigen B2 OS=Homo sapiens OX=	8.589077037
High	O94973	AP-2 complex subunit alpha-2 OS=Homo sapiens OX=960	8.550208967
High	Q96AE4	Far upstream element-binding protein 1 OS=Homo sapie	8.538253059
High	P62917	60S ribosomal protein L8 OS=Homo sapiens OX=9606 GN	8.515238359
High	P31689	DnaJ homolog subfamily A member 1 OS=Homo sapiens	8.490389728
High	Q96KG9	N-terminal kinase-like protein OS=Homo sapiens OX=960	8.449512371
High	P46977	Dolichyl-diphosphooligosaccharide--protein glycosyltrans	8.428122485
High	P32119	Peroxiredoxin-2 OS=Homo sapiens OX=9606 GN=PRDX2	8.4202055
High	P36915	Guanine nucleotide-binding protein-like 1 OS=Homo sapi	8.409576406
High	Q9UBV2	Protein sel-1 homolog 1 OS=Homo sapiens OX=9606 GN=	8.310107101
High	P43304	Glycerol-3-phosphate dehydrogenase, mitochondrial OS=	8.272653162
High	Q9Y5M8	Signal recognition particle receptor subunit beta OS=Hor	8.23283605
High	Q9NZ01	Very-long-chain enoyl-CoA reductase OS=Homo sapiens (	8.211516513
High	P13489	Ribonuclease inhibitor OS=Homo sapiens OX=9606 GN=F	8.198144941
High	P63241	Eukaryotic translation initiation factor 5A-1 OS=Homo sa	8.197888353
High	P39656	Dolichyl-diphosphooligosaccharide--protein glycosyltrans	8.15479406
High	Q15717	ELAV-like protein 1 OS=Homo sapiens OX=9606 GN=ELAV	8.145120643
High	Q9NVH2	Integrator complex subunit 7 OS=Homo sapiens OX=960(	8.134904131
High	P27348	14-3-3 protein theta OS=Homo sapiens OX=9606 GN=YW	8.128998985
High	Q8IXB1	DnaJ homolog subfamily C member 10 OS=Homo sapiens	8.078654645
High	Q86XL3	Ankyrin repeat and LEM domain-containing protein 2 OS	8.053697013
High	Q9NR45	Sialic acid synthase OS=Homo sapiens OX=9606 GN=NAN	8.039670522
High	Q16630	Cleavage and polyadenylation specificity factor subunit 6	8.032866299
High	O15355	Protein phosphatase 1G OS=Homo sapiens OX=9606 GN=	8.02543544
High	P00505	Aspartate aminotransferase, mitochondrial OS=Homo sa	8.006379685
High	O43747	AP-1 complex subunit gamma-1 OS=Homo sapiens OX=9	7.942366596
High	P05783	Keratin, type I cytoskeletal 18 OS=Homo sapiens OX=960	7.934830828
High	P46781	40S ribosomal protein S9 OS=Homo sapiens OX=9606 GN	7.895468349
High	Q8WXF1	Paraspeckle component 1 OS=Homo sapiens OX=9606 G	7.889253034
High	Q9UDR5	Alpha-aminoadipic semialdehyde synthase, mitochondria	7.881328293
High	P46777	60S ribosomal protein L5 OS=Homo sapiens OX=9606 GN	7.783076262

High	Q9Y223	Bifunctional UDP-N-acetylglucosamine 2-epimerase/N-ac	7.716438892
High	Q14444	Caprin-1 OS=Homo sapiens OX=9606 GN=CAPRIN1 PE=1	7.703307039
High	Q8TC07	TBC1 domain family member 15 OS=Homo sapiens OX=9	7.690448931
High	Q9UPY3	Endoribonuclease Dicer OS=Homo sapiens OX=9606 GN=	7.689859198
High	P00492	Hypoxanthine-guanine phosphoribosyltransferase OS=Ho	7.679860113
High	Q9UH99	SUN domain-containing protein 2 OS=Homo sapiens OX=	7.61506481
High	P28161	Glutathione S-transferase Mu 2 OS=Homo sapiens OX=96	7.598969111
High	Q96CX2	BTB/POZ domain-containing protein KCTD12 OS=Homo s	7.537917354
High	Q04917	14-3-3 protein eta OS=Homo sapiens OX=9606 GN=YWH	7.537278827
High	Q14738	Serine/threonine-protein phosphatase 2A 56 kDa regulat	7.512929533
High	O15164	Transcription intermediary factor 1-alpha OS=Homo sapi	7.49321716
High	Q9UBB6	Neurochondrin OS=Homo sapiens OX=9606 GN=NCDN P	7.483511958
High	O15371	Eukaryotic translation initiation factor 3 subunit D OS=Ho	7.47700692
High	Q03252	Lamin-B2 OS=Homo sapiens OX=9606 GN=LMNB2 PE=1 S	7.466771785
High	P25787	Proteasome subunit alpha type-2 OS=Homo sapiens OX=	7.447267174
High	Q9NPQ8	Synembryn-A OS=Homo sapiens OX=9606 GN=RIC8A PE=	7.437987316
High	Q13283	Ras GTPase-activating protein-binding protein 1 OS=Hom	7.414380445
High	P18124	60S ribosomal protein L7 OS=Homo sapiens OX=9606 GN	7.397719464
High	Q8WVM8	Sec1 family domain-containing protein 1 OS=Homo sapie	7.360350167
High	O75083	WD repeat-containing protein 1 OS=Homo sapiens OX=9	7.322888298
High	P49916	DNA ligase 3 OS=Homo sapiens OX=9606 GN=LIG3 PE=1	7.300357136
High	Q12797	Aspartyl/asparaginyl beta-hydroxylase OS=Homo sapiens	7.294604432
High	Q12996	Cleavage stimulation factor subunit 3 OS=Homo sapiens	7.27653267
High	O75170	Serine/threonine-protein phosphatase 6 regulatory subu	7.245878268
High	O60256	Phosphoribosyl pyrophosphate synthase-associated prot	7.244344633
High	Q96RU2	Ubiquitin carboxyl-terminal hydrolase 28 OS=Homo sapie	7.191641512
High	P27797	Calreticulin OS=Homo sapiens OX=9606 GN=CALR PE=1 S	7.185206881
High	Q02241	Kinesin-like protein KIF23 OS=Homo sapiens OX=9606 GN	7.179337815
High	P46779	60S ribosomal protein L28 OS=Homo sapiens OX=9606 G	7.165997578
High	Q9UID3	Vacuolar protein sorting-associated protein 51 homolog	7.153291855
High	Q15477	Helicase SKI2W OS=Homo sapiens OX=9606 GN=SKIV2L F	7.141180302
High	P61313	60S ribosomal protein L15 OS=Homo sapiens OX=9606 G	7.105431849
High	P62195	26S proteasome regulatory subunit 8 OS=Homo sapiens	7.101158748
High	Q5SW79	Centrosomal protein of 170 kDa OS=Homo sapiens OX=9	7.060656248
High	P19823	Inter-alpha-trypsin inhibitor heavy chain H2 OS=Homo sa	7.021413509
High	Q9H7D7	WD repeat-containing protein 26 OS=Homo sapiens OX=	7.014204689
High	Q99536	Synaptic vesicle membrane protein VAT-1 homolog OS=H	7.013823702
High	P31948	Stress-induced-phosphoprotein 1 OS=Homo sapiens OX=	7.011970615
High	Q53H12	Acylglycerol kinase, mitochondrial OS=Homo sapiens OX=	7.009306039
High	Q5VIR6	Vacuolar protein sorting-associated protein 53 homolog	6.993700262
High	A6NHR9	Structural maintenance of chromosomes flexible hinge d	6.991919888
High	O14773	Tripeptidyl-peptidase 1 OS=Homo sapiens OX=9606 GN=	6.982341059
High	P21796	Voltage-dependent anion-selective channel protein 1 OS	6.896775131
High	Q06203	Amidophosphoribosyltransferase OS=Homo sapiens OX=	6.894767376
High	O14929	Histone acetyltransferase type B catalytic subunit OS=Ho	6.880744111
High	Q6P9B9	Integrator complex subunit 5 OS=Homo sapiens OX=960	6.872993739
High	Q9UPN9	E3 ubiquitin-protein ligase TRIM33 OS=Homo sapiens OX	6.854298931

High	P61586	Transforming protein RhoA OS=Homo sapiens OX=9606 (	6.842991747
High	O15160	DNA-directed RNA polymerases I and III subunit RPAC1 C	6.821598658
High	O00203	AP-3 complex subunit beta-1 OS=Homo sapiens OX=9606	6.809629217
High	Q9UBQ7	Glyoxylate reductase/hydroxypyruvate reductase OS=Ho	6.798168798
High	Q99714	3-hydroxyacyl-CoA dehydrogenase type-2 OS=Homo sap	6.782358159
High	Q15785	Mitochondrial import receptor subunit TOM34 OS=Hom	6.778874344
High	O43823	A-kinase anchor protein 8 OS=Homo sapiens OX=9606 G	6.766767759
High	P60866	40S ribosomal protein S20 OS=Homo sapiens OX=9606 G	6.756287458
High	P37802	Transgelin-2 OS=Homo sapiens OX=9606 GN=TAGLN2 PE	6.730407209
High	Q9P035	Very-long-chain (3R)-3-hydroxyacyl-CoA dehydratase 3 C	6.724821051
High	Q9Y394	Dehydrogenase/reductase SDR family member 7 OS=Ho	6.686581536
High	O76021	Ribosomal L1 domain-containing protein 1 OS=Homo sap	6.678950532
High	Q6PGP7	Tetratricopeptide repeat protein 37 OS=Homo sapiens O	6.657817432
High	P08559	Pyruvate dehydrogenase E1 component subunit alpha, s	6.629443909
High	Q96CS3	FAS-associated factor 2 OS=Homo sapiens OX=9606 GN=	6.627880755
High	O15294	UDP-N-acetylglucosamine--peptide N-acetylglucosaminy	6.601269253
High	P14678	Small nuclear ribonucleoprotein-associated proteins B ar	6.597132273
High	Q01082	Spectrin beta chain, non-erythrocytic 1 OS=Homo sapien	6.587725193
High	Q15006	ER membrane protein complex subunit 2 OS=Homo sapi	6.570856223
High	P13861	cAMP-dependent protein kinase type II-alpha regulatory	6.566870482
High	Q8N8S7	Protein enabled homolog OS=Homo sapiens OX=9606 GN	6.556884416
High	P26368	Splicing factor U2AF 65 kDa subunit OS=Homo sapiens O	6.54660062
High	P42356	Phosphatidylinositol 4-kinase alpha OS=Homo sapiens O)	6.520904161
High	P08237	ATP-dependent 6-phosphofructokinase, muscle type OS=	6.493531582
High	Q9UNS1	Protein timeless homolog OS=Homo sapiens OX=9606 G	6.473272484
High	P53007	Tricarboxylate transport protein, mitochondrial OS=Hom	6.436803316
High	O95861	3'(2'),5'-bisphosphate nucleotidase 1 OS=Homo sapiens (	6.434742657
High	Q7L2H7	Eukaryotic translation initiation factor 3 subunit M OS=H	6.420412109
High	Q16555	Dihydropyrimidinase-related protein 2 OS=Homo sapiens	6.404666393
High	Q15436	Protein transport protein Sec23A OS=Homo sapiens OX=	6.391800591
High	P49959	Double-strand break repair protein MRE11 OS=Homo sa	6.38902062
High	O75439	Mitochondrial-processing peptidase subunit beta OS=Ho	6.376895912
High	Q86UV5	Ubiquitin carboxyl-terminal hydrolase 48 OS=Homo sapi	6.375044187
High	Q8N3R9	MAGUK p55 subfamily member 5 OS=Homo sapiens OX=	6.371573875
High	P11387	DNA topoisomerase 1 OS=Homo sapiens OX=9606 GN=T	6.370780309
High	Q6P3W7	SCY1-like protein 2 OS=Homo sapiens OX=9606 GN=SCYL	6.308942886
High	A1L0T0	Acetolactate synthase-like protein OS=Homo sapiens OX	6.307770164
High	O00178	GTP-binding protein 1 OS=Homo sapiens OX=9606 GN=G	6.2953962
High	P19174	1-phosphatidylinositol 4,5-bisphosphate phosphodiester	6.247568632
High	O43303	Centriolar coiled-coil protein of 110 kDa OS=Homo sapie	6.247050539
High	Q9BV20	Methylthioribose-1-phosphate isomerase OS=Homo sapi	6.224668607
High	O95299	NADH dehydrogenase [ubiquinone] 1 alpha subcomplex	6.215454026
High	Q9H993	Protein-glutamate O-methyltransferase OS=Homo sapier	6.210688973
High	O14776	Transcription elongation regulator 1 OS=Homo sapiens C	6.207900297
High	P36542	ATP synthase subunit gamma, mitochondrial OS=Homo s	6.146731916
High	Q9NWY4	Histone PARylation factor 1 OS=Homo sapiens OX=9606	6.142461986
High	O75396	Vesicle-trafficking protein SEC22b OS=Homo sapiens OX=	6.111764327

High	P61619	Protein transport protein Sec61 subunit alpha isoform 1	6.095288458
High	P62280	40S ribosomal protein S11 OS=Homo sapiens OX=9606 G	6.094309785
High	Q9Y277	Voltage-dependent anion-selective channel protein 3 OS	6.037119521
High	Q7Z4Q2	HEAT repeat-containing protein 3 OS=Homo sapiens OX=	5.971996385
High	Q15019	Septin-2 OS=Homo sapiens OX=9606 GN=SEPT2 PE=1 SV	5.948837441
High	Q8WUA4	General transcription factor 3C polypeptide 2 OS=Homo	5.938177145
High	Q9Y2Z0	Protein SGT1 homolog OS=Homo sapiens OX=9606 GN=S	5.935905398
High	O43615	Mitochondrial import inner membrane translocase subu	5.926853022
High	O95486	Protein transport protein Sec24A OS=Homo sapiens OX=	5.90517962
High	P49903	Selenide, water dikinase 1 OS=Homo sapiens OX=9606 G	5.904830649
High	Q9Y617	Phosphoserine aminotransferase OS=Homo sapiens OX=	5.877992108
High	Q6IN85	Serine/threonine-protein phosphatase 4 regulatory subu	5.868271711
High	P31150	Rab GDP dissociation inhibitor alpha OS=Homo sapiens C	5.866141875
High	Q96EK5	KIF1-binding protein OS=Homo sapiens OX=9606 GN=KIF	5.859806321
High	P23258	Tubulin gamma-1 chain OS=Homo sapiens OX=9606 GN=	5.841336019
High	Q9UGP8	Translocation protein SEC63 homolog OS=Homo sapiens	5.830306349
High	Q9Y383	Putative RNA-binding protein Luc7-like 2 OS=Homo sapie	5.827573712
High	Q96RT1	Erbin OS=Homo sapiens OX=9606 GN=ERBIN PE=1 SV=2	5.822395115
High	P17655	Calpain-2 catalytic subunit OS=Homo sapiens OX=9606 G	5.812482099
High	P62913	60S ribosomal protein L11 OS=Homo sapiens OX=9606 G	5.753784147
High	P08621	U1 small nuclear ribonucleoprotein 70 kDa OS=Homo sa	5.753457106
High	Q9H2G2	STE20-like serine/threonine-protein kinase OS=Homo sa	5.746191142
High	Q9Y6E2	Basic leucine zipper and W2 domain-containing protein 2	5.73652935
High	O15027	Protein transport protein Sec16A OS=Homo sapiens OX=	5.73641631
High	P51116	Fragile X mental retardation syndrome-related protein 2	5.716020716
High	P55209	Nucleosome assembly protein 1-like 1 OS=Homo sapiens	5.687707736
High	Q9Y4R8	Telomere length regulation protein TEL2 homolog OS=Ho	5.687611051
High	Q9UBC2	Epidermal growth factor receptor substrate 15-like 1 OS=	5.68318907
High	Q9Y6Y8	SEC23-interacting protein OS=Homo sapiens OX=9606 GI	5.675028983
High	Q9BUJ2	Heterogeneous nuclear ribonucleoprotein U-like protein	5.674353411
High	Q9Y266	Nuclear migration protein nudC OS=Homo sapiens OX=96	5.648745141
High	P51784	Ubiquitin carboxyl-terminal hydrolase 11 OS=Homo sapie	5.640847333
High	Q15555	Microtubule-associated protein RP/EB family member 2	5.636249294
High	Q9UHB6	LIM domain and actin-binding protein 1 OS=Homo sapien	5.632437605
High	Q9Y6C9	Mitochondrial carrier homolog 2 OS=Homo sapiens OX=9	5.598030424
High	Q8NB90	ATPase family protein 2 homolog OS=Homo sapiens OX=	5.576718228
High	Q9UII4	E3 ISG15--protein ligase HERC5 OS=Homo sapiens OX=96	5.572351629
High	Q96SI9	Spermatid perinuclear RNA-binding protein OS=Homo sa	5.569338234
High	Q96J01	THO complex subunit 3 OS=Homo sapiens OX=9606 GN=	5.554777209
High	O60264	SWI/SNF-related matrix-associated actin-dependent regu	5.55219721
High	Q16512	Serine/threonine-protein kinase N1 OS=Homo sapiens O	5.524200431
High	P52907	F-actin-capping protein subunit alpha-1 OS=Homo sapien	5.52275881
High	P26373	60S ribosomal protein L13 OS=Homo sapiens OX=9606 G	5.514749149
High	P85037	Forkhead box protein K1 OS=Homo sapiens OX=9606 GN	5.513771703
High	P51532	Transcription activator BRG1 OS=Homo sapiens OX=9606	5.511925718
High	Q14CX7	N-alpha-acetyltransferase 25, NatB auxiliary subunit OS=	5.503208684
High	P62070	Ras-related protein R-Ras2 OS=Homo sapiens OX=9606 G	5.486558516

High	O95905	Protein ecdysoneless homolog OS=Homo sapiens OX=96	5.485850866
High	Q9P258	Protein RCC2 OS=Homo sapiens OX=9606 GN=RCC2 PE=1	5.482279569
High	P11766	Alcohol dehydrogenase class-3 OS=Homo sapiens OX=96	5.479429198
High	Q9UNL2	Translocon-associated protein subunit gamma OS=Homo	5.474178048
High	Q9Y4C1	Lysine-specific demethylase 3A OS=Homo sapiens OX=96	5.461551948
High	Q9BV79	Enoyl-[acyl-carrier-protein] reductase, mitochondrial OS=	5.459045191
High	P33121	Long-chain-fatty-acid--CoA ligase 1 OS=Homo sapiens OX	5.453148135
High	P35249	Replication factor C subunit 4 OS=Homo sapiens OX=960	5.428215831
High	Q4KMP7	TBC1 domain family member 10B OS=Homo sapiens OX=	5.407705077
High	Q9NRF8	CTP synthase 2 OS=Homo sapiens OX=9606 GN=CTPS2 P	5.393068054
High	Q8N163	Cell cycle and apoptosis regulator protein 2 OS=Homo sa	5.390868302
High	P51553	Isocitrate dehydrogenase [NAD] subunit gamma, mitoch	5.388663928
High	Q5VUJ6	Leucine-rich repeat and calponin homology domain-cont	5.383193981
High	P50416	Carnitine O-palmitoyltransferase 1, liver isoform OS=Hor	5.381575329
High	P49720	Proteasome subunit beta type-3 OS=Homo sapiens OX=9	5.352128235
High	Q9Y5V3	Melanoma-associated antigen D1 OS=Homo sapiens OX=	5.338138873
High	A7E2V4	Zinc finger SWIM domain-containing protein 8 OS=Homo	5.324947371
High	P13674	Prolyl 4-hydroxylase subunit alpha-1 OS=Homo sapiens O	5.309918385
High	Q10570	Cleavage and polyadenylation specificity factor subunit 1	5.272214826
High	Q96T88	E3 ubiquitin-protein ligase UHRF1 OS=Homo sapiens OX=	5.263598796
High	Q96H79	Zinc finger CCCH-type antiviral protein 1-like OS=Homo s	5.261060969
High	Q16836	Hydroxyacyl-coenzyme A dehydrogenase, mitochondrial	5.248598864
High	O14545	TRAF-type zinc finger domain-containing protein 1 OS=H	5.247490599
High	P29966	Myristoylated alanine-rich C-kinase substrate OS=Homo	5.247348279
High	Q9NZB2	Constitutive coactivator of PPAR-gamma-like protein 1 O	5.246171446
High	Q8WTT2	Nucleolar complex protein 3 homolog OS=Homo sapiens	5.236056401
High	P62424	60S ribosomal protein L7a OS=Homo sapiens OX=9606 G	5.21690748
High	Q66K14	TBC1 domain family member 9B OS=Homo sapiens OX=9	5.205557965
High	O00139	Kinesin-like protein KIF2A OS=Homo sapiens OX=9606 GI	5.200892791
High	Q08379	Golgin subfamily A member 2 OS=Homo sapiens OX=960	5.156852469
High	P40926	Malate dehydrogenase, mitochondrial OS=Homo sapiens	5.149381698
High	P49770	Translation initiation factor eIF-2B subunit beta OS=Hom	5.143661002
High	P08708	40S ribosomal protein S17 OS=Homo sapiens OX=9606 G	5.140294067
High	Q8IZH2	5'-3' exoribonuclease 1 OS=Homo sapiens OX=9606 GN=	5.131238042
High	Q5ST30	Valine--tRNA ligase, mitochondrial OS=Homo sapiens OX	5.121388942
High	Q13363	C-terminal-binding protein 1 OS=Homo sapiens OX=9606	5.116843832
High	P52630	Signal transducer and activator of transcription 2 OS=Hor	5.107126593
High	P01023	Alpha-2-macroglobulin OS=Homo sapiens OX=9606 GN=A	5.099670457
High	O95232	Luc7-like protein 3 OS=Homo sapiens OX=9606 GN=LUC7	5.070427821
High	Q14746	Conserved oligomeric Golgi complex subunit 2 OS=Homo	5.063737658
High	E9PAV3	Nascent polypeptide-associated complex subunit alpha, r	5.061730517
High	Q8IZ69	tRNA (uracil-5-)-methyltransferase homolog A OS=Homo	5.052376818
High	Q6AI08	HEAT repeat-containing protein 6 OS=Homo sapiens OX=	5.008419543
High	O00116	Alkyldihydroxyacetonephosphate synthase, peroxisomal	5.006782515
High	Q15084	Protein disulfide-isomerase A6 OS=Homo sapiens OX=96	4.999130675
High	Q9P2E9	Ribosome-binding protein 1 OS=Homo sapiens OX=9606	4.990549104
High	Q9BV86	N-terminal Xaa-Pro-Lys N-methyltransferase 1 OS=Homo	4.98625794

High	P35250	Replication factor C subunit 2 OS=Homo sapiens OX=960	4.981715692
High	P52747	Zinc finger protein 143 OS=Homo sapiens OX=9606 GN=Z	4.977206653
High	Q92538	Golgi-specific brefeldin A-resistance guanine nucleotide e	4.97617428
High	Q6PJG6	BRCA1-associated ATM activator 1 OS=Homo sapiens OX	4.97495602
High	Q96QU8	Exportin-6 OS=Homo sapiens OX=9606 GN=XPO6 PE=1 S	4.953309857
High	Q96E17	Ras-related protein Rab-3C OS=Homo sapiens OX=9606 (	4.940254459
High	Q01968	Inositol polyphosphate 5-phosphatase OCRL-1 OS=Homo	4.918418107
High	Q9P2I0	Cleavage and polyadenylation specificity factor subunit 2	4.90963806
High	Q9ULK4	Mediator of RNA polymerase II transcription subunit 23 (	4.901765569
High	P62847	40S ribosomal protein S24 OS=Homo sapiens OX=9606 G	4.901010361
High	O60306	RNA helicase aquarius OS=Homo sapiens OX=9606 GN=A	4.894489815
High	P35611	Alpha-adducin OS=Homo sapiens OX=9606 GN=ADD1 PE	4.893125109
High	Q99570	Phosphoinositide 3-kinase regulatory subunit 4 OS=Hom	4.87484417
High	Q96DV4	39S ribosomal protein L38, mitochondrial OS=Homo sapi	4.872765581
High	P37837	Transaldolase OS=Homo sapiens OX=9606 GN=TALDO1 P	4.87086838
High	P23526	Adenosylhomocysteinase OS=Homo sapiens OX=9606 GI	4.8686945
High	P52565	Rho GDP-dissociation inhibitor 1 OS=Homo sapiens OX=9	4.851397345
High	Q99729	Heterogeneous nuclear ribonucleoprotein A/B OS=Homo	4.84924356
High	Q93034	Cullin-5 OS=Homo sapiens OX=9606 GN=CUL5 PE=1 SV=4	4.784098187
High	Q9UHD2	Serine/threonine-protein kinase TBK1 OS=Homo sapiens	4.736600669
High	Q9H0W5	Coiled-coil domain-containing protein 8 OS=Homo sapier	4.730487056
High	P42765	3-ketoacyl-CoA thiolase, mitochondrial OS=Homo sapien	4.725842151
High	P28482	Mitogen-activated protein kinase 1 OS=Homo sapiens O)	4.696563181
High	Q6DKJ4	Nucleoredoxin OS=Homo sapiens OX=9606 GN=NXN PE=	4.670195478
High	Q9UMS4	Pre-mRNA-processing factor 19 OS=Homo sapiens OX=96	4.659555885
High	Q9BZL6	Serine/threonine-protein kinase D2 OS=Homo sapiens O.	4.650046102
High	Q8TC12	Retinol dehydrogenase 11 OS=Homo sapiens OX=9606 G	4.643591673
High	P04908	Histone H2A type 1-B/E OS=Homo sapiens OX=9606 GN=	4.63201974
High	Q8IX18	Probable ATP-dependent RNA helicase DHX40 OS=Homo	4.627925741
High	Q9UHI6	Probable ATP-dependent RNA helicase DDX20 OS=Homo	4.585360853
High	P06744	Glucose-6-phosphate isomerase OS=Homo sapiens OX=9	4.575405811
High	P61254	60S ribosomal protein L26 OS=Homo sapiens OX=9606 G	4.555672848
High	P48507	Glutamate--cysteine ligase regulatory subunit OS=Homo	4.544005721
High	Q96S59	Ran-binding protein 9 OS=Homo sapiens OX=9606 GN=R.	4.531062194
High	Q13123	Protein Red OS=Homo sapiens OX=9606 GN=IK PE=1 SV=	4.5289434
High	O95786	Probable ATP-dependent RNA helicase DDX58 OS=Homo	4.521904616
High	O14641	Segment polarity protein dishevelled homolog DVL-2 OS=	4.505845406
High	Q5W0B1	RING finger protein 219 OS=Homo sapiens OX=9606 GN=	4.498603845
High	Q13610	Periodic tryptophan protein 1 homolog OS=Homo sapier	4.458545571
High	P00374	Dihydrofolate reductase OS=Homo sapiens OX=9606 GN	4.415668776
High	Q6P1Q9	Methyltransferase-like protein 2B OS=Homo sapiens OX=	4.414313722
High	O75175	CCR4-NOT transcription complex subunit 3 OS=Homo sa|	4.390832626
High	Q16186	Proteasomal ubiquitin receptor ADRM1 OS=Homo sapier	4.389446295
High	O75688	Protein phosphatase 1B OS=Homo sapiens OX=9606 GN=	4.320390428
High	O75521	Enoyl-CoA delta isomerase 2, mitochondrial OS=Homo sa	4.284665316
High	P14314	Glucosidase 2 subunit beta OS=Homo sapiens OX=9606 (	4.271448923
High	P22234	Multifunctional protein ADE2 OS=Homo sapiens OX=960	4.270443566

High	Q96J02	E3 ubiquitin-protein ligase Itchy homolog OS=Homo sapi	4.25904644
High	P40763	Signal transducer and activator of transcription 3 OS=Ho	4.253210168
High	P02771	Alpha-fetoprotein OS=Homo sapiens OX=9606 GN=AFP P	4.248259126
High	Q9UPU5	Ubiquitin carboxyl-terminal hydrolase 24 OS=Homo sapie	4.241898133
High	Q9NQX3	Gephyrin OS=Homo sapiens OX=9606 GN=GPHN PE=1 SV	4.234554982
High	P52434	DNA-directed RNA polymerases I, II, and III subunit RPAB	4.231954186
High	Q13547	Histone deacetylase 1 OS=Homo sapiens OX=9606 GN=H	4.2278986
High	Q9BSJ2	Gamma-tubulin complex component 2 OS=Homo sapien	4.226267414
High	Q5TAX3	Terminal uridylyltransferase 4 OS=Homo sapiens OX=960	4.226102437
High	P36507	Dual specificity mitogen-activated protein kinase kinase	4.191383965
High	P46778	60S ribosomal protein L21 OS=Homo sapiens OX=9606 G	4.186356644
High	Q3MII6	TBC1 domain family member 25 OS=Homo sapiens OX=9	4.167299529
High	Q9P1Y5	Calmodulin-regulated spectrin-associated protein 3 OS=H	4.164372834
High	Q96HE7	ERO1-like protein alpha OS=Homo sapiens OX=9606 GN=	4.151687696
High	P04792	Heat shock protein beta-1 OS=Homo sapiens OX=9606 G	4.151072287
High	Q05209	Tyrosine-protein phosphatase non-receptor type 12 OS=	4.144905049
High	Q96EK9	Protein KTI12 homolog OS=Homo sapiens OX=9606 GN=	4.144057054
High	P14635	G2/mitotic-specific cyclin-B1 OS=Homo sapiens OX=9606	4.142486585
High	Q86V85	Integral membrane protein GPR180 OS=Homo sapiens O	4.124417896
High	Q9Y263	Phospholipase A-2-activating protein OS=Homo sapiens	4.117247844
High	P62244	40S ribosomal protein S15a OS=Homo sapiens OX=9606	4.115254467
High	Q92597	Protein NDRG1 OS=Homo sapiens OX=9606 GN=NDRG1	4.104246058
High	Q9Y262	Eukaryotic translation initiation factor 3 subunit L OS=Ho	4.097833871
High	Q5T6F2	Ubiquitin-associated protein 2 OS=Homo sapiens OX=960	4.092130668
High	P35268	60S ribosomal protein L22 OS=Homo sapiens OX=9606 G	4.087671642
High	P22033	Methylmalonyl-CoA mutase, mitochondrial OS=Homo sa	4.079928876
High	O14920	Inhibitor of nuclear factor kappa-B kinase subunit beta O	4.077326432
High	Q9H270	Vacuolar protein sorting-associated protein 11 homolog	4.074378545
High	Q15102	Platelet-activating factor acetylhydrolase IB subunit gam	4.073366951
High	P61962	DDB1- and CUL4-associated factor 7 OS=Homo sapiens C	4.06606928
High	O60231	Pre-mRNA-splicing factor ATP-dependent RNA helicase D	4.061380218
High	P50914	60S ribosomal protein L14 OS=Homo sapiens OX=9606 G	4.032873419
High	O00487	26S proteasome non-ATPase regulatory subunit 14 OS=H	4.023439254
High	O00299	Chloride intracellular channel protein 1 OS=Homo sapien	4.021363052
High	Q9NUQ9	Protein FAM49B OS=Homo sapiens OX=9606 GN=FAM49	4.020360588
High	P41229	Lysine-specific demethylase 5C OS=Homo sapiens OX=96	4.017579504
High	Q3YEC7	Rab-like protein 6 OS=Homo sapiens OX=9606 GN=RABL	4.010016542
High	Q92620	Pre-mRNA-splicing factor ATP-dependent RNA helicase P	4.005858889
High	Q9BZH6	WD repeat-containing protein 11 OS=Homo sapiens OX=	4.001522697
High	P11717	Cation-independent mannose-6-phosphate receptor OS=	4.000387564
High	Q9P253	Vacuolar protein sorting-associated protein 18 homolog	3.998707077
High	Q9UBX3	Mitochondrial dicarboxylate carrier OS=Homo sapiens O	3.98088371
High	Q92995	Ubiquitin carboxyl-terminal hydrolase 13 OS=Homo sapie	3.979383106
High	Q3MHD2	Protein LSM12 homolog OS=Homo sapiens OX=9606 GN	3.966978555
High	Q99459	Cell division cycle 5-like protein OS=Homo sapiens OX=96	3.966726343
High	Q15382	GTP-binding protein Rheb OS=Homo sapiens OX=9606 G	3.948461609
High	P43487	Ran-specific GTPase-activating protein OS=Homo sapiens	3.947306058

High	Q9Y2W1	Thyroid hormone receptor-associated protein 3 OS=Hom	3.932061868
High	P54578	Ubiquitin carboxyl-terminal hydrolase 14 OS=Homo sapie	3.927382523
High	O75828	Carbonyl reductase [NADPH] 3 OS=Homo sapiens OX=96	3.916139199
High	Q13308	Inactive tyrosine-protein kinase 7 OS=Homo sapiens OX=	3.890421453
High	P51571	Translocon-associated protein subunit delta OS=Homo sa	3.885722703
High	P28066	Proteasome subunit alpha type-5 OS=Homo sapiens OX=	3.88008459
High	Q9H9E3	Conserved oligomeric Golgi complex subunit 4 OS=Homo	3.877784122
High	Q9HD33	39S ribosomal protein L47, mitochondrial OS=Homo sapi	3.86998805
High	Q12789	General transcription factor 3C polypeptide 1 OS=Homo	3.863102644
High	P46060	Ran GTPase-activating protein 1 OS=Homo sapiens OX=9	3.85886391
High	Q86VI3	Ras GTPase-activating-like protein IQGAP3 OS=Homo sap	3.85326066
High	O95372	Acyl-protein thioesterase 2 OS=Homo sapiens OX=9606 (	3.821023053
High	O14976	Cyclin-G-associated kinase OS=Homo sapiens OX=9606 G	3.819587367
High	P15880	40S ribosomal protein S2 OS=Homo sapiens OX=9606 GN	3.818755695
High	P42684	Tyrosine-protein kinase ABL2 OS=Homo sapiens OX=960	3.814741235
High	Q9H2P9	Diphthine methyl ester synthase OS=Homo sapiens OX=9	3.809388202
High	P04004	Vitronectin OS=Homo sapiens OX=9606 GN=VTN PE=1 SV	3.789413975
High	P04899	Guanine nucleotide-binding protein G(i) subunit alpha-2	3.781202002
High	P24941	Cyclin-dependent kinase 2 OS=Homo sapiens OX=9606 G	3.778985967
High	P22413	Ectonucleotide pyrophosphatase/phosphodiesterase fan	3.770318158
High	P49589	Cysteine--tRNA ligase, cytoplasmic OS=Homo sapiens OX	3.764724123
High	Q9UK59	Lariat debranching enzyme OS=Homo sapiens OX=9606 (	3.751536282
High	Q02218	2-oxoglutarate dehydrogenase, mitochondrial OS=Homo	3.747949613
High	P43686	26S proteasome regulatory subunit 6B OS=Homo sapiens	3.746904414
High	Q15437	Protein transport protein Sec23B OS=Homo sapiens OX=	3.729554092
High	P09110	3-ketoacyl-CoA thiolase, peroxisomal OS=Homo sapiens	3.71219827
High	Q7Z6Z7	E3 ubiquitin-protein ligase HUWE1 OS=Homo sapiens OX	3.710620888
High	Q8ND04	Protein SMG8 OS=Homo sapiens OX=9606 GN=SMG8 PE	3.705313376
High	Q9NVP1	ATP-dependent RNA helicase DDX18 OS=Homo sapiens (	3.696803943
High	Q96R06	Sperm-associated antigen 5 OS=Homo sapiens OX=9606	3.695294102
High	P81605	Dermcidin OS=Homo sapiens OX=9606 GN=DCD PE=1 SV	3.678194516
High	O76071	Probable cytosolic iron-sulfur protein assembly protein (	3.649308588
High	Q9Y5K5	Ubiquitin carboxyl-terminal hydrolase isozyme L5 OS=Ho	3.637706062
High	Q8TF05	Serine/threonine-protein phosphatase 4 regulatory subu	3.633633084
High	Q9H4A3	Serine/threonine-protein kinase WNK1 OS=Homo sapien	3.622875958
High	P68402	Platelet-activating factor acetylhydrolase IB subunit beta	3.619065537
High	O75494	Serine/arginine-rich splicing factor 10 OS=Homo sapiens	3.602755419
High	Q86TB9	Protein PAT1 homolog 1 OS=Homo sapiens OX=9606 GN	3.589054141
High	O43491	Band 4.1-like protein 2 OS=Homo sapiens OX=9606 GN=I	3.583463336
High	Q9H2U1	ATP-dependent DNA/RNA helicase DHX36 OS=Homo sap	3.573976984
High	Q8WUA2	Peptidyl-prolyl cis-trans isomerase-like 4 OS=Homo sapie	3.552709669
High	Q9P2R3	Rabankyrin-5 OS=Homo sapiens OX=9606 GN=ANKFY1 P	3.552221991
High	Q9GZR7	ATP-dependent RNA helicase DDX24 OS=Homo sapiens (	3.549904924
High	P35237	Serpin B6 OS=Homo sapiens OX=9606 GN=SERPINB6 PE=	3.531692556
High	Q9BY89	Uncharacterized protein KIAA1671 OS=Homo sapiens OX	3.516127546
High	Q00535	Cyclin-dependent-like kinase 5 OS=Homo sapiens OX=96	3.51446732
High	Q9BVQ7	Spermatogenesis-associated protein 5-like protein 1 OS=	3.50751899

High	O60568	Multifunctional procollagen lysine hydroxylase and glyco	3.506123889
High	Q99661	Kinesin-like protein KIF2C OS=Homo sapiens OX=9606 GI	3.496950605
High	Q16762	Thiosulfate sulfurtransferase OS=Homo sapiens OX=9606	3.482276405
High	Q9NW82	WD repeat-containing protein 70 OS=Homo sapiens OX=	3.480303233
High	Q5VZE5	N-alpha-acetyltransferase 35, NatC auxiliary subunit OS=	3.478731124
High	Q9H7Z7	Prostaglandin E synthase 2 OS=Homo sapiens OX=9606 G	3.473789996
High	P11171	Protein 4.1 OS=Homo sapiens OX=9606 GN=EPB41 PE=1	3.463694128
High	P18858	DNA ligase 1 OS=Homo sapiens OX=9606 GN=LIG1 PE=1	3.462432743
High	A4D1P6	WD repeat-containing protein 91 OS=Homo sapiens OX=	3.45259454
High	Q9NTK5	Obg-like ATPase 1 OS=Homo sapiens OX=9606 GN=OLA1	3.438064237
High	Q86YZ3	Hornerin OS=Homo sapiens OX=9606 GN=HRNR PE=1 SV	3.434860848
High	Q8IYI6	Exocyst complex component 8 OS=Homo sapiens OX=96	3.419531216
High	Q01780	Exosome component 10 OS=Homo sapiens OX=9606 GN	3.39653084
High	Q13625	Apoptosis-stimulating of p53 protein 2 OS=Homo sapiens	3.392223396
High	Q8TE77	Protein phosphatase Slingshot homolog 3 OS=Homo sapi	3.390832626
High	Q9BZF1	Oxysterol-binding protein-related protein 8 OS=Homo sa	3.390832626
High	Q8WWK9	Cytoskeleton-associated protein 2 OS=Homo sapiens OX=	3.372634143
High	Q99543	DnaJ homolog subfamily C member 2 OS=Homo sapiens	3.372429336
High	Q9HCG8	Pre-mRNA-splicing factor CWC22 homolog OS=Homo sap	3.366531544
High	P56537	Eukaryotic translation initiation factor 6 OS=Homo sapien	3.363612414
High	Q9NP79	Vacuolar protein sorting-associated protein VTA1 homol	3.362110183
High	Q9UNE7	E3 ubiquitin-protein ligase CHIP OS=Homo sapiens OX=9	3.352842452
High	Q9Y3A5	Ribosome maturation protein SBDS OS=Homo sapiens O	3.341471214
High	P29144	Tripeptidyl-peptidase 2 OS=Homo sapiens OX=9606 GN=	3.339134522
High	Q9HCN4	GPN-loop GTPase 1 OS=Homo sapiens OX=9606 GN=GPN	3.338281942
High	P82650	28S ribosomal protein S22, mitochondrial OS=Homo sapi	3.337431033
High	Q9BWU0	Kanadaptin OS=Homo sapiens OX=9606 GN=SLC4A1AP P	3.335264031
High	P98175	RNA-binding protein 10 OS=Homo sapiens OX=9606 GN=	3.329939783
High	P07737	Profilin-1 OS=Homo sapiens OX=9606 GN=PFN1 PE=1 SV	3.329105046
High	Q6DN90	IQ motif and SEC7 domain-containing protein 1 OS=Hom	3.319030282
High	Q9BRX2	Protein pelota homolog OS=Homo sapiens OX=9606 GN=	3.316412682
High	Q15648	Mediator of RNA polymerase II transcription subunit 1 O	3.316142795
High	P62249	40S ribosomal protein S16 OS=Homo sapiens OX=9606 G	3.310513552
High	O00267	Transcription elongation factor SPT5 OS=Homo sapiens C	3.2892059
High	P02786	Transferrin receptor protein 1 OS=Homo sapiens OX=960	3.277283833
High	P30048	Thioredoxin-dependent peroxide reductase, mitochondr	3.269459964
High	P62906	60S ribosomal protein L10a OS=Homo sapiens OX=9606	3.258970667
High	O96005	Cleft lip and palate transmembrane protein 1 OS=Homo	3.250650239
High	Q8WYQ5	Microprocessor complex subunit DGCR8 OS=Homo sapie	3.243896628
High	Q5JWF2	Guanine nucleotide-binding protein G(s) subunit alpha is	3.242224509
High	Q9C0E2	Exportin-4 OS=Homo sapiens OX=9606 GN=XPO4 PE=1 S	3.232473101
High	O14818	Proteasome subunit alpha type-7 OS=Homo sapiens OX=	3.226872076
High	Q13423	NAD(P) transhydrogenase, mitochondrial OS=Homo sapi	3.221921138
High	Q6P1J9	Parafibromin OS=Homo sapiens OX=9606 GN=CDC73 PE=	3.21346152
High	P40429	60S ribosomal protein L13a OS=Homo sapiens OX=9606	3.212004957
High	Q9UIU6	Homeobox protein SIX4 OS=Homo sapiens OX=9606 GN=	3.162348442
High	P67809	Nuclease-sensitive element-binding protein 1 OS=Homo	3.162105517

High	Q9NXF1	Testis-expressed protein 10 OS=Homo sapiens OX=9606	3.138525331
High	Q9Y2Q3	Glutathione S-transferase kappa 1 OS=Homo sapiens OX=	3.135548253
High	Q8IVS2	Malonyl-CoA-acyl carrier protein transacylase, mitochon	3.131355562
High	Q99567	Nuclear pore complex protein Nup88 OS=Homo sapiens	3.124244421
High	P39019	40S ribosomal protein S19 OS=Homo sapiens OX=9606 G	3.124128809
High	P41214	Eukaryotic translation initiation factor 2D OS=Homo sapi	3.117931056
High	Q9UNX4	WD repeat-containing protein 3 OS=Homo sapiens OX=9	3.105407052
High	Q9UNN5	FAS-associated factor 1 OS=Homo sapiens OX=9606 GN=	3.105185671
High	Q96EA4	Protein Spindly OS=Homo sapiens OX=9606 GN=SPDL1 P	3.0759793
High	Q6ZSR9	Uncharacterized protein FLJ45252 OS=Homo sapiens OX=	3.065501549
High	Q8WVC6	Dephospho-CoA kinase domain-containing protein OS=H	3.057198534
High	Q9Y295	Developmentally-regulated GTP-binding protein 1 OS=Hc	3.054482178
High	Q6ZXV5	Transmembrane and TPR repeat-containing protein 3 OS	3.05232126
High	O95352	Ubiquitin-like modifier-activating enzyme ATG7 OS=Hom	3.048711063
High	Q9Y2U8	Inner nuclear membrane protein Man1 OS=Homo sapier	3.038721321
High	Q7L1Q6	Basic leucine zipper and W2 domain-containing protein 1	3.037274282
High	Q86UK7	E3 ubiquitin-protein ligase ZNF598 OS=Homo sapiens OX	3.029327757
High	Q8N122	Regulatory-associated protein of mTOR OS=Homo sapier	3.02086153
High	P26358	DNA (cytosine-5)-methyltransferase 1 OS=Homo sapiens	3.016911615
High	Q96P47	Arf-GAP with GTPase, ANK repeat and PH domain-contai	3.007667441
High	P28288	ATP-binding cassette sub-family D member 3 OS=Homo s	3.005594853
High	Q13867	Bleomycin hydrolase OS=Homo sapiens OX=9606 GN=BL	3.003488328
High	Q96HA7	Tonsoku-like protein OS=Homo sapiens OX=9606 GN=TO	2.996970529
High	Q7L0Y3	tRNA methyltransferase 10 homolog C OS=Homo sapiens	2.992584128
High	Q8N201	Integrator complex subunit 1 OS=Homo sapiens OX=9606	2.985479461
High	P53004	Biliverdin reductase A OS=Homo sapiens OX=9606 GN=B	2.978810701
High	P00918	Carbonic anhydrase 2 OS=Homo sapiens OX=9606 GN=CA	2.964570262
High	Q7Z6B7	SLIT-ROBO Rho GTPase-activating protein 1 OS=Homo sa	2.962175249
High	Q7Z478	ATP-dependent RNA helicase DHX29 OS=Homo sapiens C	2.938547521
High	P42677	40S ribosomal protein S27 OS=Homo sapiens OX=9606 G	2.934420285
High	Q53EL6	Programmed cell death protein 4 OS=Homo sapiens OX=	2.933674075
High	Q9H3N1	Thioredoxin-related transmembrane protein 1 OS=Homo	2.92885471
High	P55039	Developmentally-regulated GTP-binding protein 2 OS=Hc	2.926648298
High	O94776	Metastasis-associated protein MTA2 OS=Homo sapiens C	2.923359556
High	Q9Y6X4	Soluble lamin-associated protein of 75 kDa OS=Homo sap	2.920456993
High	Q8WX92	Negative elongation factor B OS=Homo sapiens OX=9606	2.913996294
High	Q6PIW4	Fidgetin-like protein 1 OS=Homo sapiens OX=9606 GN=F	2.913996294
High	P00846	ATP synthase subunit a OS=Homo sapiens OX=9606 GN=	2.897909474
High	Q96RT8	Gamma-tubulin complex component 5 OS=Homo sapien	2.888737486
High	P18669	Phosphoglycerate mutase 1 OS=Homo sapiens OX=9606	2.886390849
High	Q15942	Zyxin OS=Homo sapiens OX=9606 GN=ZYX PE=1 SV=1	2.884722409
High	Q7Z2Y8	Interferon-induced very large GTPase 1 OS=Homo sapier	2.86710023
High	Q15054	DNA polymerase delta subunit 3 OS=Homo sapiens OX=9	2.844360366
High	O94979	Protein transport protein Sec31A OS=Homo sapiens OX=	2.838033384
High	P61421	V-type proton ATPase subunit d 1 OS=Homo sapiens OX=	2.831502516
High	Q8NCE2	Myotubularin-related protein 14 OS=Homo sapiens OX=9	2.823330067
High	O43819	Protein SCO2 homolog, mitochondrial OS=Homo sapiens	2.818728228

High	O95394	Phosphoacetylglucosamine mutase OS=Homo sapiens O)	2.81693126
High	Q9UJC3	Protein Hook homolog 1 OS=Homo sapiens OX=9606 GN	2.812730622
High	P09429	High mobility group protein B1 OS=Homo sapiens OX=96	2.808548986
High	P25490	Transcriptional repressor protein YY1 OS=Homo sapiens	2.80161787
High	Q96SU4	Oxysterol-binding protein-related protein 9 OS=Homo sa	2.799970733
High	P82673	28S ribosomal protein S35, mitochondrial OS=Homo sapi	2.796423225
High	Q15746	Myosin light chain kinase, smooth muscle OS=Homo sapi	2.791021483
High	P62937	Peptidyl-prolyl cis-trans isomerase A OS=Homo sapiens C	2.788345599
High	O15381	Nuclear valosin-containing protein-like OS=Homo sapien	2.779368981
High	Q9Y5A7	NEDD8 ultimate buster 1 OS=Homo sapiens OX=9606 GN	2.777803954
High	P31350	Ribonucleoside-diphosphate reductase subunit M2 OS=H	2.771856392
High	P28072	Proteasome subunit beta type-6 OS=Homo sapiens OX=9	2.770574152
High	Q7Z4S6	Kinesin-like protein KIF21A OS=Homo sapiens OX=9606 (	2.766242637
High	P08243	Asparagine synthetase [glutamine-hydrolyzing] OS=Hom	2.759700418
High	Q9BTE7	DCN1-like protein 5 OS=Homo sapiens OX=9606 GN=DCL	2.758204569
High	P67812	Signal peptidase complex catalytic subunit SEC11A OS=H	2.754487332
High	P42766	60S ribosomal protein L35 OS=Homo sapiens OX=9606 G	2.753994096
High	Q96IJ6	Mannose-1-phosphate guanyltransferase alpha OS=Hom	2.750513758
High	Q9H4L7	SWI/SNF-related matrix-associated actin-dependent regu	2.747891366
High	Q9BYW2	Histone-lysine N-methyltransferase SETD2 OS=Homo sap	2.743522794
High	A6NHQ2	rRNA/tRNA 2'-O-methyltransferase fibrillarin-like protein	2.739213331
High	Q9UBB9	Tuftelin-interacting protein 11 OS=Homo sapiens OX=960	2.732593581
High	Q14254	Flotillin-2 OS=Homo sapiens OX=9606 GN=FLOT2 PE=1 S\	2.714217726
High	Q00796	Sorbitol dehydrogenase OS=Homo sapiens OX=9606 GN=	2.71354353
High	P45954	Short/branched chain specific acyl-CoA dehydrogenase, r	2.709297757
High	Q9UBT7	Alpha-catulin OS=Homo sapiens OX=9606 GN=CTNNAL1	2.694863681
High	O15031	Plexin-B2 OS=Homo sapiens OX=9606 GN=PLXNB2 PE=1	2.684239509
High	Q96PU5	E3 ubiquitin-protein ligase NEDD4-like OS=Homo sapiens	2.681936665
High	Q96RS0	Trimethylguanosine synthase OS=Homo sapiens OX=960	2.677780705
High	Q8N1G4	Leucine-rich repeat-containing protein 47 OS=Homo sapi	2.677160727
High	Q93050	V-type proton ATPase 116 kDa subunit a isoform 1 OS=H	2.673459331
High	Q01804	OTU domain-containing protein 4 OS=Homo sapiens OX=	2.671824339
High	P82930	28S ribosomal protein S34, mitochondrial OS=Homo sapi	2.670602121
High	Q08170	Serine/arginine-rich splicing factor 4 OS=Homo sapiens C	2.664141089
High	P00491	Purine nucleoside phosphorylase OS=Homo sapiens OX=	2.661543506
High	Q16527	Cysteine and glycine-rich protein 2 OS=Homo sapiens OX	2.660747366
High	Q9P0I2	ER membrane protein complex subunit 3 OS=Homo sapi	2.639785387
High	Q8TCT9	Minor histocompatibility antigen H13 OS=Homo sapiens	2.632830511
High	Q9BYG3	MKI67 FHA domain-interacting nucleolar phosphoprotei	2.630413109
High	Q92990	Glomulin OS=Homo sapiens OX=9606 GN=GLMN PE=1 S\	2.625434939
High	O75832	26S proteasome non-ATPase regulatory subunit 10 OS=H	2.622147781
High	Q9NYJ8	TGF-beta-activated kinase 1 and MAP3K7-binding protei	2.61960784
High	Q96TA1	Niban-like protein 1 OS=Homo sapiens OX=9606 GN=FAN	2.614751318
High	Q9Y4J8	Dystrobrevin alpha OS=Homo sapiens OX=9606 GN=DTN	2.591929714
High	Q07065	Cytoskeleton-associated protein 4 OS=Homo sapiens OX	2.587707491
High	Q15645	Pachytene checkpoint protein 2 homolog OS=Homo sapi	2.573326112
High	Q96PU8	Protein quaking OS=Homo sapiens OX=9606 GN=QKI PE=	2.562090964

High	Q8TCJ2	Dolichyl-diphosphooligosaccharide--protein glycosyltrans	2.56082526
High	P62851	40S ribosomal protein S25 OS=Homo sapiens OX=9606 G	2.548981548
High	Q92542	Nicastrin OS=Homo sapiens OX=9606 GN=NCSTN PE=1 S\	2.541211118
High	P68431	Histone H3.1 OS=Homo sapiens OX=9606 GN=HIST1H3A	2.540402959
High	P49406	39S ribosomal protein L19, mitochondrial OS=Homo sapi	2.531948209
High	Q9BZE9	Tether containing UBX domain for GLUT4 OS=Homo sapi	2.500746315
High	Q8WX93	Palladin OS=Homo sapiens OX=9606 GN=PALLD PE=1 SV=	2.500038134
High	P04181	Ornithine aminotransferase, mitochondrial OS=Homo sa	2.500038134
High	Q5SRE5	Nucleoporin NUP188 homolog OS=Homo sapiens OX=96	2.495937117
High	Q13595	Transformer-2 protein homolog alpha OS=Homo sapiens	2.491335637
High	O43813	LanC-like protein 1 OS=Homo sapiens OX=9606 GN=LAN	2.484921325
High	Q8ND83	SLAIN motif-containing protein 1 OS=Homo sapiens OX=	2.482276405
High	Q13586	Stromal interaction molecule 1 OS=Homo sapiens OX=96	2.479254528
High	O15357	Phosphatidylinositol 3,4,5-trisphosphate 5-phosphatase	2.474436942
High	Q15345	Leucine-rich repeat-containing protein 41 OS=Homo sapi	2.459670525
High	Q9NXE4	Sphingomyelin phosphodiesterase 4 OS=Homo sapiens C	2.458795309
High	P33981	Dual specificity protein kinase TTK OS=Homo sapiens OX	2.44551084
High	P51610	Host cell factor 1 OS=Homo sapiens OX=9606 GN=HCFC1	2.441651491
High	Q9ULE4	Protein FAM184B OS=Homo sapiens OX=9606 GN=FAM1	2.420902673
High	P50851	Lipopolysaccharide-responsive and beige-like anchor pro	2.41408829
High	Q9BYD2	39S ribosomal protein L9, mitochondrial OS=Homo sapie	2.40263395
High	P51003	Poly(A) polymerase alpha OS=Homo sapiens OX=9606 GI	2.39968067
High	Q9BUT1	3-hydroxybutyrate dehydrogenase type 2 OS=Homo sapi	2.398265852
High	Q9NR31	GTP-binding protein SAR1a OS=Homo sapiens OX=9606 (	2.39631445
High	P62753	40S ribosomal protein S6 OS=Homo sapiens OX=9606 GN	2.388170521
High	O15085	Rho guanine nucleotide exchange factor 11 OS=Homo sa	2.38563016
High	Q969V6	Myocardin-related transcription factor A OS=Homo sapie	2.375614759
High	Q15363	Transmembrane emp24 domain-containing protein 2 OS	2.375614759
High	P21399	Cytoplasmic aconitate hydratase OS=Homo sapiens OX=	2.372224625
High	O75475	PC4 and SFRS1-interacting protein OS=Homo sapiens OX	2.371713269
High	Q9UBE0	SUMO-activating enzyme subunit 1 OS=Homo sapiens O)	2.370284667
High	Q9NRL3	Striatin-4 OS=Homo sapiens OX=9606 GN=STRN4 PE=1 S\	2.369063881
High	P13473	Lysosome-associated membrane glycoprotein 2 OS=Hom	2.361510743
High	P19623	Spermidine synthase OS=Homo sapiens OX=9606 GN=SR	2.360912129
High	Q8N442	Translation factor GUF1, mitochondrial OS=Homo sapien	2.355758414
High	Q9Y3Y2	Chromatin target of PRMT1 protein OS=Homo sapiens O	2.334231449
High	P17152	Transmembrane protein 11, mitochondrial OS=Homo sap	2.327625021
High	Q9BQN1	Protein FAM83C OS=Homo sapiens OX=9606 GN=FAM83	2.320481256
High	O43301	Heat shock 70 kDa protein 12A OS=Homo sapiens OX=96	2.318939756
High	P07237	Protein disulfide-isomerase OS=Homo sapiens OX=9606	2.315962963
High	Q5J8M3	ER membrane protein complex subunit 4 OS=Homo sapi	2.284414448
High	P01859	Immunoglobulin heavy constant gamma 2 OS=Homo sap	2.281332265
High	Q9UHP3	Ubiquitin carboxyl-terminal hydrolase 25 OS=Homo sapie	2.264038009
High	Q14527	Helicase-like transcription factor OS=Homo sapiens OX=	2.250650239
High	Q6P996	Pyridoxal-dependent decarboxylase domain-containing p	2.245575211
High	Q9NXS2	Glutaminyl-peptide cyclotransferase-like protein OS=Hor	2.229958446
High	P61247	40S ribosomal protein S3a OS=Homo sapiens OX=9606 G	2.22562904

High	Q6P158	Putative ATP-dependent RNA helicase DHX57 OS=Homo	2.211690188
High	Q96H55	Unconventional myosin-XIX OS=Homo sapiens OX=9606	2.209081805
High	P61353	60S ribosomal protein L27 OS=Homo sapiens OX=9606 G	2.207818504
High	O15047	Histone-lysine N-methyltransferase SETD1A OS=Homo sa	2.18276527
High	Q9Y6N7	Roundabout homolog 1 OS=Homo sapiens OX=9606 GN=	2.16481693
High	Q12792	Twinfilin-1 OS=Homo sapiens OX=9606 GN=TWF1 PE=1 S	2.160396271
High	O00541	Pescadillo homolog OS=Homo sapiens OX=9606 GN=PES	2.151072287
High	Q6YHU6	Thyroid adenoma-associated protein OS=Homo sapiens (	2.148497047
High	Q9UIW2	Plexin-A1 OS=Homo sapiens OX=9606 GN=PLXNA1 PE=1	2.147215131
High	Q8WYA6	Beta-catenin-like protein 1 OS=Homo sapiens OX=9606 G	2.13430394
High	O43164	E3 ubiquitin-protein ligase Praja-2 OS=Homo sapiens OX=	2.131825959
High	Q9UBS4	DnaJ homolog subfamily B member 11 OS=Homo sapiens	2.120617363
High	Q9NZJ7	Mitochondrial carrier homolog 1 OS=Homo sapiens OX=9	2.118786584
High	P60900	Proteasome subunit alpha type-6 OS=Homo sapiens OX=	2.117931056
High	Q68BL7	Olfactomedin-like protein 2A OS=Homo sapiens OX=9606	2.095933948
High	Q96NW4	Ankyrin repeat domain-containing protein 27 OS=Homo	2.094419972
High	P62888	60S ribosomal protein L30 OS=Homo sapiens OX=9606 G	2.088895682
High	P07384	Calpain-1 catalytic subunit OS=Homo sapiens OX=9606 G	2.08639805
High	P62263	40S ribosomal protein S14 OS=Homo sapiens OX=9606 G	2.086239114
High	Q9NX46	Poly(ADP-ribose) glycohydrolase ARH3 OS=Homo sapiens	2.07654873
High	Q15070	Mitochondrial inner membrane protein OXA1L OS=Homo	2.075772419
High	P57088	Transmembrane protein 33 OS=Homo sapiens OX=9606	2.05764465
High	Q9UPN7	Serine/threonine-protein phosphatase 6 regulatory subu	2.054334501
High	Q9UBD5	Origin recognition complex subunit 3 OS=Homo sapiens (	2.031750606
High	Q16513	Serine/threonine-protein kinase N2 OS=Homo sapiens O	2.025396208
High	P56545	C-terminal-binding protein 2 OS=Homo sapiens OX=9606	2.014259259
High	O60830	Mitochondrial import inner membrane translocase subu	2.01407978
High	Q96K76	Ubiquitin carboxyl-terminal hydrolase 47 OS=Homo sapie	2.012199714
High	P61009	Signal peptidase complex subunit 3 OS=Homo sapiens O	2.001566281
High	Q13330	Metastasis-associated protein MTA1 OS=Homo sapiens (	1.982966661
High	Q9Y3D9	28S ribosomal protein S23, mitochondrial OS=Homo sapi	1.97798426
High	Q969X5	Endoplasmic reticulum-Golgi intermediate compartment	1.974694135
High	Q7Z739	YTH domain-containing family protein 3 OS=Homo sapie	1.973058372
High	Q9Y312	Protein AAR2 homolog OS=Homo sapiens OX=9606 GN=A	1.958607315
High	Q9BY32	Inosine triphosphate pyrophosphatase OS=Homo sapiens	1.943857738
Medium	O60645	Exocyst complex component 3 OS=Homo sapiens OX=96	1.924088239
Medium	Q9UBK8	Methionine synthase reductase OS=Homo sapiens OX=9	1.924088239
Medium	Q07021	Complement component 1 Q subcomponent-binding pro	1.903437562
Medium	Q9H0A0	RNA cytidine acetyltransferase OS=Homo sapiens OX=96	1.897566294
Medium	Q05086	Ubiquitin-protein ligase E3A OS=Homo sapiens OX=9606	1.883392256
Medium	P52790	Hexokinase-3 OS=Homo sapiens OX=9606 GN=HK3 PE=1	1.864231485
Medium	P61160	Actin-related protein 2 OS=Homo sapiens OX=9606 GN=A	1.863279433
Medium	P40222	Alpha-taxilin OS=Homo sapiens OX=9606 GN=TXLNA PE=	1.853871964
Medium	Q96JG6	Syndetin OS=Homo sapiens OX=9606 GN=VPS50 PE=1 SV	1.851705903
Medium	O75127	Pentatricopeptide repeat-containing protein 1, mitochor	1.815024809
Medium	P27694	Replication protein A 70 kDa DNA-binding subunit OS=Ho	1.81417464
Medium	P09661	U2 small nuclear ribonucleoprotein A' OS=Homo sapiens	1.809108283

Medium	Q9BZE4	Nucleolar GTP-binding protein 1 OS=Homo sapiens OX=9	1.791827473
Medium	Q9UP83	Conserved oligomeric Golgi complex subunit 5 OS=Homo	1.784098187
Medium	Q86V48	Leucine zipper protein 1 OS=Homo sapiens OX=9606 GN=	1.779891912
Medium	O15042	U2 snRNP-associated SURP motif-containing protein OS=	1.777283529
Medium	P08579	U2 small nuclear ribonucleoprotein B'' OS=Homo sapiens	1.755722879
Medium	O75608	Acyl-protein thioesterase 1 OS=Homo sapiens OX=9606 (	1.727926212
Medium	Q8NEB9	Phosphatidylinositol 3-kinase catalytic subunit type 3 OS=	1.716472635
Medium	Q5JTH9	RRP12-like protein OS=Homo sapiens OX=9606 GN=RRP1	1.699839463
Medium	Q9NV70	Exocyst complex component 1 OS=Homo sapiens OX=96	1.681519275
Medium	O00505	Importin subunit alpha-4 OS=Homo sapiens OX=9606 GN	1.68006156
Medium	Q8NB78	Lysine-specific histone demethylase 1B OS=Homo sapien	1.675100503
Medium	Q9NNW5	WD repeat-containing protein 6 OS=Homo sapiens OX=9	1.666351243
Medium	Q9ULC4	Malignant T-cell-amplified sequence 1 OS=Homo sapiens	1.644740094
Medium	Q6L8Q7	2',5'-phosphodiesterase 12 OS=Homo sapiens OX=9606 (	1.64454848
Medium	P06756	Integrin alpha-V OS=Homo sapiens OX=9606 GN=ITGAV I	1.64244628
Medium	B2RTY4	Unconventional myosin-IXa OS=Homo sapiens OX=9606	1.639785387
Medium	Q9BQ69	O-acetyl-ADP-ribose deacetylase MACROD1 OS=Homo sa	1.634512015
Medium	Q99961	Endophilin-A2 OS=Homo sapiens OX=9606 GN=SH3GL1 F	1.632271454
Medium	P36957	Dihydrolipoyllysine-residue succinyltransferase compone	1.613679426
Medium	Q6NW34	Nucleolus and neural progenitor protein OS=Homo sapie	1.603277721
Medium	Q9Y697	Cysteine desulfurase, mitochondrial OS=Homo sapiens O	1.60119227
Medium	Q9BTX1	Nucleoporin NDC1 OS=Homo sapiens OX=9606 GN=NDC	1.601018933
Medium	Q9NZJ5	Eukaryotic translation initiation factor 2-alpha kinase 3 C	1.565590792
Medium	P09936	Ubiquitin carboxyl-terminal hydrolase isozyme L1 OS=Ho	1.564314862
Medium	Q01433	AMP deaminase 2 OS=Homo sapiens OX=9606 GN=AMPI	1.559878397
Medium	Q8WWY3	U4/U6 small nuclear ribonucleoprotein Prp31 OS=Homo	1.556111453
Medium	Q9GZZ1	N-alpha-acetyltransferase 50 OS=Homo sapiens OX=960(	1.552067134
Medium	Q92618	Zinc finger protein 516 OS=Homo sapiens OX=9606 GN=Z	1.546834607
Medium	P19388	DNA-directed RNA polymerases I, II, and III subunit RPAB	1.540456742
Medium	O43681	ATPase ASNA1 OS=Homo sapiens OX=9606 GN=ASNA1 P	1.518270803
Medium	P50225	Sulfotransferase 1A1 OS=Homo sapiens OX=9606 GN=SU	1.516698048
Medium	A6NKC9	SH2 domain-containing protein 7 OS=Homo sapiens OX=	1.515415471
Medium	Q9Y467	Sal-like protein 2 OS=Homo sapiens OX=9606 GN=SALL2	1.507099989
Medium	P19474	E3 ubiquitin-protein ligase TRIM21 OS=Homo sapiens OX	1.503070352
Medium	Q01081	Splicing factor U2AF 35 kDa subunit OS=Homo sapiens O	1.491335637
Medium	Q5VT52	Regulation of nuclear pre-mRNA domain-containing prot	1.490663042
Medium	O15020	Spectrin beta chain, non-erythrocytic 2 OS=Homo sapien	1.487715937
Medium	P02765	Alpha-2-HS-glycoprotein OS=Homo sapiens OX=9606 GN	1.482672118
Medium	Q9NZP5	Olfactory receptor 5AC2 OS=Homo sapiens OX=9606 GN	1.474825572
Medium	P15153	Ras-related C3 botulinum toxin substrate 2 OS=Homo sa	1.465719995
Medium	Q9UM00	Calcium load-activated calcium channel OS=Homo sapier	1.465466244
Medium	P21359	Neurofibromin OS=Homo sapiens OX=9606 GN=NF1 PE=	1.462306806
Medium	O75069	Transmembrane and coiled-coil domains protein 2 OS=H	1.449894001
Medium	P46776	60S ribosomal protein L27a OS=Homo sapiens OX=9606	1.436756299
Medium	Q96B67	Arrestin domain-containing protein 3 OS=Homo sapiens	1.435926021
Medium	Q96G21	U3 small nucleolar ribonucleoprotein protein IMP4 OS=H	1.429223631
Medium	Q8WUF5	RelA-associated inhibitor OS=Homo sapiens OX=9606 GN	1.418164076

Medium	Q9UFF9	CCR4-NOT transcription complex subunit 8 OS=Homo sap	1.417822962
Medium	Q13188	Serine/threonine-protein kinase 3 OS=Homo sapiens OX=	1.40428338
Medium	Q5TCZ1	SH3 and PX domain-containing protein 2A OS=Homo sap	1.400772137
Medium	Q9BYT8	Neurolysin, mitochondrial OS=Homo sapiens OX=9606 G	1.398483216
Medium	O43683	Mitotic checkpoint serine/threonine-protein kinase BUB1	1.387958255
Medium	Q9H2X9	Solute carrier family 12 member 5 OS=Homo sapiens OX	1.380384994
Medium	Q14966	Zinc finger protein 638 OS=Homo sapiens OX=9606 GN=Z	1.374584648
Medium	Q13033	Striatin-3 OS=Homo sapiens OX=9606 GN=STRN3 PE=1 SV	1.346594509
Medium	Q9Y5Q9	General transcription factor 3C polypeptide 3 OS=Homo	1.33733659
Medium	Q9P2R7	Succinate--CoA ligase [ADP-forming] subunit beta, mitoch	1.335170059
Medium	O75190	DnaJ homolog subfamily B member 6 OS=Homo sapiens	1.330962199
Medium	Q8WXE9	Stonin-2 OS=Homo sapiens OX=9606 GN=STON2 PE=1 SV	1.321299565
Medium	Q3SY69	Mitochondrial 10-formyltetrahydrofolate dehydrogenase	1.320662969
Medium	Q9H936	Mitochondrial glutamate carrier 1 OS=Homo sapiens OX=	1.313989709
Medium	A3KMH1	von Willebrand factor A domain-containing protein 8 OS=	1.31264943
Medium	P25788	Proteasome subunit alpha type-3 OS=Homo sapiens OX=	1.312114475
Medium	Q13615	Myotubularin-related protein 3 OS=Homo sapiens OX=96	1.291324207
Medium	Q8N0U8	Vitamin K epoxide reductase complex subunit 1-like prot	1.279179418
Medium	P02788	Lactotransferrin OS=Homo sapiens OX=9606 GN=LTF PE=	1.278024598
Medium	P07203	Glutathione peroxidase 1 OS=Homo sapiens OX=9606 GN	1.272377422
Medium	Q8NCA5	Protein FAM98A OS=Homo sapiens OX=9606 GN=FAM98	1.271808601
Medium	Q9UL03	Integrator complex subunit 6 OS=Homo sapiens OX=9606	1.262489309
Medium	O43395	U4/U6 small nuclear ribonucleoprotein Prp3 OS=Homo s	1.254300773
Medium	Q15691	Microtubule-associated protein RP/EB family member 1	1.254300773
Medium	O94988	Protein FAM13A OS=Homo sapiens OX=9606 GN=FAM13	1.253287977
Medium	P46063	ATP-dependent DNA helicase Q1 OS=Homo sapiens OX=9	1.2486439
Medium	P78316	Nucleolar protein 14 OS=Homo sapiens OX=9606 GN=NO	1.240332155
Medium	Q9Y2W6	Tudor and KH domain-containing protein OS=Homo sapie	1.217957583
Medium	Q4V328	GRIP1-associated protein 1 OS=Homo sapiens OX=9606 G	1.216310764
Medium	O43815	Striatin OS=Homo sapiens OX=9606 GN=STRN PE=1 SV=4	1.211973116
Medium	Q86YT6	E3 ubiquitin-protein ligase MIB1 OS=Homo sapiens OX=9	1.205302873
Medium	Q969S9	Ribosome-releasing factor 2, mitochondrial OS=Homo sa	1.202040356
Medium	P13798	Acylamino-acid-releasing enzyme OS=Homo sapiens OX=	1.198047765
Medium	Q9NYF8	Bcl-2-associated transcription factor 1 OS=Homo sapiens	1.196884445
Medium	Q15118	[Pyruvate dehydrogenase (acetyl-transferring)] kinase iso	1.191789027
Medium	Q9HBF4	Zinc finger FYVE domain-containing protein 1 OS=Homo s	1.176982477
Medium	Q3V6T2	Girdin OS=Homo sapiens OX=9606 GN=CCDC88A PE=1 SV	1.165833716
Medium	Q9H8M5	Metal transporter CNNM2 OS=Homo sapiens OX=9606 G	1.163739197
Medium	Q96S55	ATPase WRNIP1 OS=Homo sapiens OX=9606 GN=WRNIP	1.154467883
Medium	P51114	Fragile X mental retardation syndrome-related protein 1	1.138286412
Medium	P62266	40S ribosomal protein S23 OS=Homo sapiens OX=9606 G	1.135370275
Medium	P25685	DnaJ homolog subfamily B member 1 OS=Homo sapiens	1.113227357
Low	Q96HS1	Serine/threonine-protein phosphatase PGAM5, mitochon	1.099086932
Low	Q9BXR6	Complement factor H-related protein 5 OS=Homo sapien	1.093557206
Low	O75915	PRA1 family protein 3 OS=Homo sapiens OX=9606 GN=A	1.09350334
Low	P06280	Alpha-galactosidase A OS=Homo sapiens OX=9606 GN=G	1.089642443
Low	Q6ZUM4	Rho GTPase-activating protein 27 OS=Homo sapiens OX=	1.089375595

Low	P49756	RNA-binding protein 25 OS=Homo sapiens OX=9606 GN=	1.076238039
Low	Q93100	Phosphorylase b kinase regulatory subunit beta OS=Hom	1.056950889
Low	Q02790	Peptidyl-prolyl cis-trans isomerase FKBP4 OS=Homo sapi	1.052272273
Low	P62995	Transformer-2 protein homolog beta OS=Homo sapiens (	1.049489107
Low	P42858	Huntingtin OS=Homo sapiens OX=9606 GN=HTT PE=1 SV	1.043639446
Low	Q9H6S0	3'-5' RNA helicase YTHDC2 OS=Homo sapiens OX=9606 G	1.037299268
Low	Q8IVV2	Lipoxygenase homology domain-containing protein 1 OS	1.020633758
Low	O75717	WD repeat and HMG-box DNA-binding protein 1 OS=Hor	1.015652742
Low	Q9H2V7	Protein spinster homolog 1 OS=Homo sapiens OX=9606 (	1.012557195
Low	Q9NVU7	Protein SDA1 homolog OS=Homo sapiens OX=9606 GN=S	0.997402019
Low	E9PIF3	Nuclear pore complex-interacting protein family membe	0.991399828
Low	P61081	NEDD8-conjugating enzyme Ubc12 OS=Homo sapiens OX	0.991399828
Low	O75976	Carboxypeptidase D OS=Homo sapiens OX=9606 GN=CPD	0.990549104
Low	O94903	Pyridoxal phosphate homeostasis protein OS=Homo sapi	0.988429556
Low	A2A2Z9	Ankyrin repeat domain-containing protein 18B OS=Homo	0.986741335
Low	Q15061	WD repeat-containing protein 43 OS=Homo sapiens OX=	0.984640245
Low	Q8IY81	pre-rRNA processing protein FTSJ3 OS=Homo sapiens OX	0.977159389
Low	O95140	Mitofusin-2 OS=Homo sapiens OX=9606 GN=MFN2 PE=1	0.973466735
Low	Q8IZF6	Adhesion G-protein coupled receptor G4 OS=Homo sapie	0.973058372
Low	Q13630	GDP-L-fucose synthase OS=Homo sapiens OX=9606 GN=T	0.962175249
Low	O00567	Nucleolar protein 56 OS=Homo sapiens OX=9606 GN=NC	0.956244873
Low	Q7Z2D5	Phospholipid phosphatase-related protein type 4 OS=Ho	0.955852379
Low	Q96DT7	Zinc finger and BTB domain-containing protein 10 OS=Hc	0.943095149
Low	Q8N5V2	Ephexin-1 OS=Homo sapiens OX=9606 GN=NGEF PE=1 SV	0.942714356
Low	P51398	28S ribosomal protein S29, mitochondrial OS=Homo sapi	0.941573976
Low	Q96JB2	Conserved oligomeric Golgi complex subunit 3 OS=Homo	0.936666641
Low	Q76NI1	Kinase non-catalytic C-lobe domain-containing protein 1	0.933674075
Low	Q96EE3	Nucleoporin SEH1 OS=Homo sapiens OX=9606 GN=SEH1	0.932929144
Low	P30085	UMP-CMP kinase OS=Homo sapiens OX=9606 GN=CMPK	0.931814138
Low	Q6F5E8	Capping protein, Arp2/3 and myosin-I linker protein 2 OS	0.921543182
Low	Q8N594	MPN domain-containing protein OS=Homo sapiens OX=9	0.920818754
Low	O60884	DnaJ homolog subfamily A member 2 OS=Homo sapiens	0.920095532
Low	Q9BVK6	Transmembrane emp24 domain-containing protein 9 OS	0.919734373
Low	Q9C0D7	Probable ribonuclease ZC3H12C OS=Homo sapiens OX=9	0.919734373
