# Supplementary material for: SARS-COV-2 protein NSP9 promotes cytokine production by targeting TBK1
Source: Front Immunol. 2023 Oct 2;14:1211816. doi: 10.3389/fimmu.2023.1211816 (PMC10580797; doi:10.3389/fimmu.2023.1211816)
Supplement: Supplementary file 4 [file Presentation_3.pdf]

Protein FD	Accession	Description	Sum PEP Score
High	P35579	Myosin-9 OS=Homo sapiens OX=9606 GN=MYH9 PE=1 SV	763.0065158
High	P35580	Myosin-10 OS=Homo sapiens OX=9606 GN=MYH10 PE=1	636.8191984
High	P11586	C-1-tetrahydrofolate synthase, cytoplasmic OS=Homo sap	457.9308364
High	P78527	DNA-dependent protein kinase catalytic subunit OS=Hom	350.9197509
High	P08238	Heat shock protein HSP 90-beta OS=Homo sapiens OX=96	329.8288882
High	P07437	Tubulin beta chain OS=Homo sapiens OX=9606 GN=TUBB	324.699364
High	P14618	Pyruvate kinase PKM OS=Homo sapiens OX=9606 GN=PKI	321.5560252
High	P49327	Fatty acid synthase OS=Homo sapiens OX=9606 GN=FASN	321.3229737
High	P0DMV9	Heat shock 70 kDa protein 1B OS=Homo sapiens OX=9606	311.7574243
High	Q13885	Tubulin beta-2A chain OS=Homo sapiens OX=9606 GN=TU	310.2571808
High	Q9BVA1	Tubulin beta-2B chain OS=Homo sapiens OX=9606 GN=TU	310.198711
High	P68371	Tubulin beta-4B chain OS=Homo sapiens OX=9606 GN=TU	308.3994887
High	P13639	Elongation factor 2 OS=Homo sapiens OX=9606 GN=EEF2	306.3737239
High	P06733	Alpha-enolase OS=Homo sapiens OX=9606 GN=ENO1 PE=	274.8862641
High	P60709	Actin, cytoplasmic 1 OS=Homo sapiens OX=9606 GN=ACT	270.9515805
High	Q9BQE3	Tubulin alpha-1C chain OS=Homo sapiens OX=9606 GN=T	258.5444055
High	P04406	Glyceraldehyde-3-phosphate dehydrogenase OS=Homo sa	257.8896603
High	P04350	Tubulin beta-4A chain OS=Homo sapiens OX=9606 GN=TU	245.3052662
High	P07900	Heat shock protein HSP 90-alpha OS=Homo sapiens OX=9	245.2936182
High	Q71U36	Tubulin alpha-1A chain OS=Homo sapiens OX=9606 GN=T	241.7498715
High	Q7Z406	Myosin-14 OS=Homo sapiens OX=9606 GN=MYH14 PE=1	223.6868916
High	P68104	Elongation factor 1-alpha 1 OS=Homo sapiens OX=9606 G	216.7346862
High	P11142	Heat shock cognate 71 kDa protein OS=Homo sapiens OX	215.5153457
High	P21333	Filamin-A OS=Homo sapiens OX=9606 GN=FLNA PE=1 SV=	210.2919744
High	P49368	T-complex protein 1 subunit gamma OS=Homo sapiens O	191.1764166
High	P78371	T-complex protein 1 subunit beta OS=Homo sapiens OX=9	178.2754613
High	P33993	DNA replication licensing factor MCM7 OS=Homo sapiens	175.5052551
High	Q14204	Cytoplasmic dynein 1 heavy chain 1 OS=Homo sapiens OX	174.4626362
High	P48643	T-complex protein 1 subunit epsilon OS=Homo sapiens O	171.8082943
High	Q00610	Clathrin heavy chain 1 OS=Homo sapiens OX=9606 GN=CL	168.4384467
High	P60842	Eukaryotic initiation factor 4A-I OS=Homo sapiens OX=96	159.1127642
High	P17987	T-complex protein 1 subunit alpha OS=Homo sapiens OX=	159.0053662
High	P06576	ATP synthase subunit beta, mitochondrial OS=Homo sapie	157.438152
High	P27708	CAD protein OS=Homo sapiens OX=9606 GN=CAD PE=1 S\	157.0112785
High	P22314	Ubiquitin-like modifier-activating enzyme 1 OS=Homo sap	155.2700587
High	Q9BUF5	Tubulin beta-6 chain OS=Homo sapiens OX=9606 GN=TUE	154.8913316
High	Q13509	Tubulin beta-3 chain OS=Homo sapiens OX=9606 GN=TUE	153.0394213
High	P49411	Elongation factor Tu, mitochondrial OS=Homo sapiens OX	151.3928499
High	P50991	T-complex protein 1 subunit delta OS=Homo sapiens OX=	149.2460367
High	P07814	Bifunctional glutamate/proline--tRNA ligase OS=Homo sa	148.9980067
High	Q99832	T-complex protein 1 subunit eta OS=Homo sapiens OX=96	147.6123637
High	Q14697	Neutral alpha-glucosidase AB OS=Homo sapiens OX=9606	145.4945265
High	Q7KZF4	Staphylococcal nuclease domain-containing protein 1 OS=	144.8763281
High	P54577	Tyrosine--tRNA ligase, cytoplasmic OS=Homo sapiens OX=	142.7696105
High	P10809	60 kDa heat shock protein, mitochondrial OS=Homo sapie	141.8805499
High	P12268	Inosine-5'-monophosphate dehydrogenase 2 OS=Homo sa	138.7491148
High	P42704	Leucine-rich PPR motif-containing protein, mitochondrial	137.2241826
High	P08670	Vimentin OS=Homo sapiens OX=9606 GN=VIM PE=1 SV=4	136.5418255
High	O14980	Exportin-1 OS=Homo sapiens OX=9606 GN=XPO1 PE=1 SV	133.8085977

High	P68032	Actin, alpha cardiac muscle 1 OS=Homo sapiens OX=9606	133.1413166
High	P35908	Keratin, type II cytoskeletal 2 epidermal OS=Homo sapien	133.1074581
High	P04843	Dolichyl-diphosphooligosaccharide--protein glycosyltransf	132.0037783
High	P11021	Endoplasmic reticulum chaperone BiP OS=Homo sapiens (	129.5998109
High	P49588	Alanine--tRNA ligase, cytoplasmic OS=Homo sapiens OX=9	129.1934685
High	P33991	DNA replication licensing factor MCM4 OS=Homo sapiens	124.7991269
High	P49915	GMP synthase [glutamine-hydrolyzing] OS=Homo sapiens	124.3715131
High	P26641	Elongation factor 1-gamma OS=Homo sapiens OX=9606 G	123.7147003
High	Q9P2J5	Leucine--tRNA ligase, cytoplasmic OS=Homo sapiens OX=9	123.4467484
High	O43175	D-3-phosphoglycerate dehydrogenase OS=Homo sapiens	123.3730712
High	P55060	Exportin-2 OS=Homo sapiens OX=9606 GN=CSE1L PE=1 SV	123.3491061
High	P50990	T-complex protein 1 subunit theta OS=Homo sapiens OX=	121.5420081
High	Q14974	Importin subunit beta-1 OS=Homo sapiens OX=9606 GN=I	121.1789885
High	P13645	Keratin, type I cytoskeletal 10 OS=Homo sapiens OX=9606	120.6110816
High	P04264	Keratin, type II cytoskeletal 1 OS=Homo sapiens OX=9606	119.8277401
High	P09874	Poly [ADP-ribose] polymerase 1 OS=Homo sapiens OX=96	119.6692137
High	P25705	ATP synthase subunit alpha, mitochondrial OS=Homo sap	118.7039218
High	Q9Y285	Phenylalanine--tRNA ligase alpha subunit OS=Homo sapie	112.7571585
High	P55786	Puromycin-sensitive aminopeptidase OS=Homo sapiens C	112.0514433
High	Q86VP6	Cullin-associated NEDD8-dissociated protein 1 OS=Homo	110.6082486
High	P40227	T-complex protein 1 subunit zeta OS=Homo sapiens OX=9	110.1091121
High	Q9Y230	RuvB-like 2 OS=Homo sapiens OX=9606 GN=RUVBL2 PE=1	109.0755475
High	P14625	Endoplasmin OS=Homo sapiens OX=9606 GN=HSP90B1 PI	108.7935659
High	P41252	Isoleucine--tRNA ligase, cytoplasmic OS=Homo sapiens OX	107.9071397
High	P31943	Heterogeneous nuclear ribonucleoprotein H OS=Homo sa	105.8031063
High	P61978	Heterogeneous nuclear ribonucleoprotein K OS=Homo sa	104.233338
High	P63244	Receptor of activated protein C kinase 1 OS=Homo sapien	104.1288065
High	Q05639	Elongation factor 1-alpha 2 OS=Homo sapiens OX=9606 G	102.8332692
High	P22102	Trifunctional purine biosynthetic protein adenosine-3 OS=	98.32145708
High	Q92616	eIF-2-alpha kinase activator GCN1 OS=Homo sapiens OX=9	98.03960226
High	O75369	Filamin-B OS=Homo sapiens OX=9606 GN=FLNB PE=1 SV=	97.31980488
High	P05023	Sodium/potassium-transporting ATPase subunit alpha-1 C	97.20500836
High	P38646	Stress-70 protein, mitochondrial OS=Homo sapiens OX=96	96.70802795
High	Q15233	Non-POU domain-containing octamer-binding protein OS=	93.35266147
High	P41250	Glycine--tRNA ligase OS=Homo sapiens OX=9606 GN=GAR	92.5494451
High	P27824	Calnexin OS=Homo sapiens OX=9606 GN=CANX PE=1 SV=	92.41209312
High	P04844	Dolichyl-diphosphooligosaccharide--protein glycosyltransf	92.18452019
High	Q14566	DNA replication licensing factor MCM6 OS=Homo sapiens	89.46326761
High	Q13200	26S proteasome non-ATPase regulatory subunit 2 OS=Hor	89.02398682
High	Q00839	Heterogeneous nuclear ribonucleoprotein U OS=Homo sa	88.8277254
High	Q13263	Transcription intermediary factor 1-beta OS=Homo sapier	88.71477769
High	P08243	Asparagine synthetase [glutamine-hydrolyzing] OS=Homo	87.39089151
High	P52292	Importin subunit alpha-1 OS=Homo sapiens OX=9606 GN=	86.12484577
High	Q9NSE4	Isoleucine--tRNA ligase, mitochondrial OS=Homo sapiens	85.71780827
High	P49591	Serine--tRNA ligase, cytoplasmic OS=Homo sapiens OX=96	85.57760487
High	P12956	X-ray repair cross-complementing protein 6 OS=Homo sap	85.04203864
High	P35527	Keratin, type I cytoskeletal 9 OS=Homo sapiens OX=9606	84.7625936
High	P26599	Polypyrimidine tract-binding protein 1 OS=Homo sapiens	84.40170133
High	P52272	Heterogeneous nuclear ribonucleoprotein M OS=Homo sa	83.28537909
High	P17844	Probable ATP-dependent RNA helicase DDX5 OS=Homo sa	82.81064067

High	Q9Y265	RuvB-like 1 OS=Homo sapiens OX=9606 GN=RUVBL1 PE=1	82.62401677
High	P26640	Valine--tRNA ligase OS=Homo sapiens OX=9606 GN=VARS	82.29933203
High	P31939	Bifunctional purine biosynthesis protein PURH OS=Homo	81.91623339
High	P25205	DNA replication licensing factor MCM3 OS=Homo sapiens	81.29425726
High	P54652	Heat shock-related 70 kDa protein 2 OS=Homo sapiens O	78.98834225
High	P38919	Eukaryotic initiation factor 4A-III OS=Homo sapiens OX=96	78.92031072
High	P52732	Kinesin-like protein KIF11 OS=Homo sapiens OX=9606 GN	78.17201404
High	Q9Y4L1	Hypoxia up-regulated protein 1 OS=Homo sapiens OX=960	77.75607074
High	P12277	Creatine kinase B-type OS=Homo sapiens OX=9606 GN=C	76.59894605
High	Q92841	Probable ATP-dependent RNA helicase DDX17 OS=Homo	76.33774424
High	P17066	Heat shock 70 kDa protein 6 OS=Homo sapiens OX=9606	75.55327339
High	Q9NVI7	ATPase family AAA domain-containing protein 3A OS=Hor	73.04865482
High	Q9UJZ1	Stomatin-like protein 2, mitochondrial OS=Homo sapiens	72.55358767
High	P22695	Cytochrome b-c1 complex subunit 2, mitochondrial OS=H	71.33153527
High	P07195	L-lactate dehydrogenase B chain OS=Homo sapiens OX=96	70.59833237
High	Q08J23	tRNA (cytosine(34)-C(5))-methyltransferase OS=Homo sap	70.45592044
High	Q14166	Tubulin--tyrosine ligase-like protein 12 OS=Homo sapiens	69.63505412
High	P53618	Coatomer subunit beta OS=Homo sapiens OX=9606 GN=C	69.16512249
High	P16615	Sarcoplasmic/endoplasmic reticulum calcium ATPase 2 OS	68.45749734
High	O00571	ATP-dependent RNA helicase DDX3X OS=Homo sapiens O	68.40378682
High	P54136	Arginine--tRNA ligase, cytoplasmic OS=Homo sapiens OX=	68.39224845
High	P38159	RNA-binding motif protein, X chromosome OS=Homo sap	68.00515121
High	O00410	Importin-5 OS=Homo sapiens OX=9606 GN=IPO5 PE=1 SV	67.75145131
High	Q16891	MICOS complex subunit MIC60 OS=Homo sapiens OX=960	67.33006446
High	P47897	Glutamine--tRNA ligase OS=Homo sapiens OX=9606 GN=C	66.59142774
High	P08865	40S ribosomal protein SA OS=Homo sapiens OX=9606 GN	66.14660866
High	O94826	Mitochondrial import receptor subunit TOM70 OS=Homo	65.64726756
High	O95373	Importin-7 OS=Homo sapiens OX=9606 GN=IPO7 PE=1 SV	63.90494811
High	P00338	L-lactate dehydrogenase A chain OS=Homo sapiens OX=96	62.91043854
High	P19338	Nucleolin OS=Homo sapiens OX=9606 GN=NCL PE=1 SV=3	62.57228036
High	Q14240	Eukaryotic initiation factor 4A-II OS=Homo sapiens OX=96	62.21649834
High	P13804	Electron transfer flavoprotein subunit alpha, mitochondri	61.94501642
High	P49736	DNA replication licensing factor MCM2 OS=Homo sapiens	61.42171144
High	P31930	Cytochrome b-c1 complex subunit 1, mitochondrial OS=H	61.01865898
High	Q99623	Prohibitin-2 OS=Homo sapiens OX=9606 GN=PHB2 PE=1 S	60.90752706
High	P54886	Delta-1-pyrroline-5-carboxylate synthase OS=Homo sapie	60.42906479
High	P14866	Heterogeneous nuclear ribonucleoprotein L OS=Homo sa	60.3052613
High	P14868	Aspartate--tRNA ligase, cytoplasmic OS=Homo sapiens OX	60.2823389
High	P30153	Serine/threonine-protein phosphatase 2A 65 kDa regulato	60.17444646
High	P13797	Plastin-3 OS=Homo sapiens OX=9606 GN=PLS3 PE=1 SV=4	59.60262806
High	Q13620	Cullin-4B OS=Homo sapiens OX=9606 GN=CUL4B PE=1 SV	59.37510344
High	Q92945	Far upstream element-binding protein 2 OS=Homo sapien	59.21399502
High	P39656	Dolichyl-diphosphooligosaccharide--protein glycosyltrans	59.01812345
High	Q9NSD9	Phenylalanine--tRNA ligase beta subunit OS=Homo sapien	58.8092139
High	Q9NZI8	Insulin-like growth factor 2 mRNA-binding protein 1 OS=H	58.78245312
High	P40939	Trifunctional enzyme subunit alpha, mitochondrial OS=Ho	58.4644828
High	P24752	Acetyl-CoA acetyltransferase, mitochondrial OS=Homo sa	58.37002152
High	Q9UNF1	Melanoma-associated antigen D2 OS=Homo sapiens OX=9	57.41552487
High	P62826	GTP-binding nuclear protein Ran OS=Homo sapiens OX=96	56.90556995
High	Q02878	60S ribosomal protein L6 OS=Homo sapiens OX=9606 GN=	56.63568751

High	P36578	60S ribosomal protein L4 OS=Homo sapiens OX=9606 GN=	56.57667269
High	Q96QK1	Vacuolar protein sorting-associated protein 35 OS=Homo	56.06260118
High	Q16576	Histone-binding protein RBBP7 OS=Homo sapiens OX=960	55.16732218
High	Q15366	Poly(rC)-binding protein 2 OS=Homo sapiens OX=9606 GN	55.10433639
High	P23921	Ribonucleoside-diphosphate reductase large subunit OS=H	54.97966893
High	Q4VCS5	Angiomotin OS=Homo sapiens OX=9606 GN=AMOT PE=1	54.48024738
High	P20700	Lamin-B1 OS=Homo sapiens OX=9606 GN=LMNB1 PE=1 S	54.4533025
High	Q8WVM8	Sec1 family domain-containing protein 1 OS=Homo sapien	53.81246074
High	P34897	Serine hydroxymethyltransferase, mitochondrial OS=Hom	53.56434739
High	P55795	Heterogeneous nuclear ribonucleoprotein H2 OS=Homo s	52.09082075
High	Q99615	DnaJ homolog subfamily C member 7 OS=Homo sapiens O	51.87100993
High	Q06203	Amidophosphoribosyltransferase OS=Homo sapiens OX=9	51.37478044
High	Q8IXI1	Mitochondrial Rho GTPase 2 OS=Homo sapiens OX=9606	51.36771952
High	Q562R1	Beta-actin-like protein 2 OS=Homo sapiens OX=9606 GN=	51.34139249
High	P78344	Eukaryotic translation initiation factor 4 gamma 2 OS=Ho	51.04826056
High	Q96I24	Far upstream element-binding protein 3 OS=Homo sapien	51.04353766
High	P22626	Heterogeneous nuclear ribonucleoproteins A2/B1 OS=Ho	50.69736032
High	Q08211	ATP-dependent RNA helicase A OS=Homo sapiens OX=960	50.65881767
High	O43776	Asparagine--tRNA ligase, cytoplasmic OS=Homo sapiens O	50.58286761
High	P13861	cAMP-dependent protein kinase type II-alpha regulatory s	50.05139185
High	P17980	26S proteasome regulatory subunit 6A OS=Homo sapiens	49.9102155
High	P56192	Methionine--tRNA ligase, cytoplasmic OS=Homo sapiens O	48.90995126
High	Q92499	ATP-dependent RNA helicase DDX1 OS=Homo sapiens OX	48.54397178
High	P52597	Heterogeneous nuclear ribonucleoprotein F OS=Homo sa	48.41785877
High	P11310	Medium-chain specific acyl-CoA dehydrogenase, mitocho	47.91789253
High	Q14008	Cytoskeleton-associated protein 5 OS=Homo sapiens OX=	47.80825055
High	P04181	Ornithine aminotransferase, mitochondrial OS=Homo sap	47.71075803
High	P62081	40S ribosomal protein S7 OS=Homo sapiens OX=9606 GN=	47.69362829
High	P11908	Ribose-phosphate pyrophosphokinase 2 OS=Homo sapien	47.56491829
High	Q9Y3I0	tRNA-splicing ligase RtcB homolog OS=Homo sapiens OX=	47.54957628
High	P17812	CTP synthase 1 OS=Homo sapiens OX=9606 GN=CTPS1 PE	47.26926603
High	P62820	Ras-related protein Rab-1A OS=Homo sapiens OX=9606 G	47.26323876
High	O60749	Sorting nexin-2 OS=Homo sapiens OX=9606 GN=SNX2 PE=	47.17461458
High	Q13148	TAR DNA-binding protein 43 OS=Homo sapiens OX=9606	47.16723534
High	Q13838	Spliceosome RNA helicase DDX39B OS=Homo sapiens OX=	46.91757072
High	P61221	ATP-binding cassette sub-family E member 1 OS=Homo sa	46.74669227
High	P43246	DNA mismatch repair protein Msh2 OS=Homo sapiens OX	46.68961812
High	P00558	Phosphoglycerate kinase 1 OS=Homo sapiens OX=9606 G	46.51783985
High	Q15365	Poly(rC)-binding protein 1 OS=Homo sapiens OX=9606 GN	46.44290707
High	Q14254	Flotillin-2 OS=Homo sapiens OX=9606 GN=FLOT2 PE=1 SV	46.37608562
High	Q16643	Drebrin OS=Homo sapiens OX=9606 GN=DBN1 PE=1 SV=4	46.30627148
High	P42167	Lamina-associated polypeptide 2, isoforms beta/gamma O	45.98244377
High	O75534	Cold shock domain-containing protein E1 OS=Homo sapie	45.84624316
High	P48735	Isocitrate dehydrogenase [NADP], mitochondrial OS=Hom	45.7879505
High	Q04637	Eukaryotic translation initiation factor 4 gamma 1 OS=Ho	45.69115894
High	O15067	Phosphoribosylformylglycinamidine synthase OS=Homo s	45.3658176
High	Q969V3	Nicalin OS=Homo sapiens OX=9606 GN=NCLN PE=1 SV=2	45.18636692
High	P26639	Threonine--tRNA ligase, cytoplasmic OS=Homo sapiens OX	45.11873883
High	P51991	Heterogeneous nuclear ribonucleoprotein A3 OS=Homo s	45.06364285
High	Q15181	Inorganic pyrophosphatase OS=Homo sapiens OX=9606 G	45.03752352

High	P61204	ADP-ribosylation factor 3 OS=Homo sapiens OX=9606 GN=	45.00480748
High	O75874	Isocitrate dehydrogenase [NADP] cytoplasmic OS=Homo s	44.95287682
High	P09651	Heterogeneous nuclear ribonucleoprotein A1 OS=Homo s	44.93371352
High	Q9UG63	ATP-binding cassette sub-family F member 2 OS=Homo sa	44.39844348
High	P27797	Calreticulin OS=Homo sapiens OX=9606 GN=CALR PE=1 SV	44.24568676
High	P28331	NADH-ubiquinone oxidoreductase 75 kDa subunit, mitoch	44.19069666
High	P16402	Histone H1.3 OS=Homo sapiens OX=9606 GN=HIST1H1D P	43.89763325
High	P51812	Ribosomal protein S6 kinase alpha-3 OS=Homo sapiens O)	43.82595701
High	Q9Y262	Eukaryotic translation initiation factor 3 subunit L OS=Hor	43.64232852
High	P10768	S-formylglutathione hydrolase OS=Homo sapiens OX=960	43.16838122
High	P30837	Aldehyde dehydrogenase X, mitochondrial OS=Homo sapi	43.03968472
High	Q96CS3	FAS-associated factor 2 OS=Homo sapiens OX=9606 GN=F	42.91287449
High	Q02750	Dual specificity mitogen-activated protein kinase kinase 1	42.84759208
High	P33992	DNA replication licensing factor MCM5 OS=Homo sapiens	42.47898916
High	Q15813	Tubulin-specific chaperone E OS=Homo sapiens OX=9606	42.33637836
High	Q02790	Peptidyl-prolyl cis-trans isomerase FKBP4 OS=Homo sapie	42.22164913
High	O00429	Dynamin-1-like protein OS=Homo sapiens OX=9606 GN=D	42.14854523
High	P42166	Lamina-associated polypeptide 2, isoform alpha OS=Hom	42.14721484
High	Q9H9B4	Sideroflexin-1 OS=Homo sapiens OX=9606 GN=SFXN1 PE=	42.07719786
High	Q15645	Pachytene checkpoint protein 2 homolog OS=Homo sapie	42.05768829
High	Q9Y2L1	Exosome complex exonuclease RRP44 OS=Homo sapiens	41.92542664
High	O95831	Apoptosis-inducing factor 1, mitochondrial OS=Homo sap	41.86836496
High	O43242	26S proteasome non-ATPase regulatory subunit 3 OS=Hor	41.63273902
High	P60891	Ribose-phosphate pyrophosphokinase 1 OS=Homo sapien	41.39530505
High	Q8WUM4	Programmed cell death 6-interacting protein OS=Homo sa	41.1627557
High	O14744	Protein arginine N-methyltransferase 5 OS=Homo sapiens	41.15468721
High	P36507	Dual specificity mitogen-activated protein kinase kinase 2	41.13099011
High	P23246	Splicing factor, proline- and glutamine-rich OS=Homo sapi	40.19693041
High	Q99460	26S proteasome non-ATPase regulatory subunit 1 OS=Hor	40.12929618
High	Q5T9A4	ATPase family AAA domain-containing protein 3B OS=Hor	39.80380312
High	Q9UBB4	Ataxin-10 OS=Homo sapiens OX=9606 GN=ATXN10 PE=1 S	39.60177237
High	P05141	ADP/ATP translocase 2 OS=Homo sapiens OX=9606 GN=S	39.52032527
High	P23396	40S ribosomal protein S3 OS=Homo sapiens OX=9606 GN=	39.25759755
High	O60763	General vesicular transport factor p115 OS=Homo sapiens	39.12233997
High	P05787	Keratin, type II cytoskeletal 8 OS=Homo sapiens OX=9606	38.99143009
High	O60701	UDP-glucose 6-dehydrogenase OS=Homo sapiens OX=960	38.88307422
High	O95202	Mitochondrial proton/calcium exchanger protein OS=Hon	38.83147677
High	Q6PI48	Aspartate--tRNA ligase, mitochondrial OS=Homo sapiens (	38.66238093
High	P11413	Glucose-6-phosphate 1-dehydrogenase OS=Homo sapiens	38.55182497
High	Q8N0X7	Spartin OS=Homo sapiens OX=9606 GN=SPART PE=1 SV=1	38.47420926
High	P50570	Dynamin-2 OS=Homo sapiens OX=9606 GN=DNM2 PE=1 S	38.22828779
High	P19623	Spermidine synthase OS=Homo sapiens OX=9606 GN=SRM	38.08018606
High	Q86Y56	Dynein assembly factor 5, axonemal OS=Homo sapiens O)	37.76060173
High	P06493	Cyclin-dependent kinase 1 OS=Homo sapiens OX=9606 GN	37.57907353
High	P46060	Ran GTPase-activating protein 1 OS=Homo sapiens OX=96	37.24149839
High	O75131	Copine-3 OS=Homo sapiens OX=9606 GN=CPNE3 PE=1 SV	37.11783888
High	P55265	Double-stranded RNA-specific adenosine deaminase OS=H	37.10931035
High	P12004	Proliferating cell nuclear antigen OS=Homo sapiens OX=9(	36.97474962
High	P55072	Transitional endoplasmic reticulum ATPase OS=Homo sap	36.93491073
High	P36871	Phosphoglucomutase-1 OS=Homo sapiens OX=9606 GN=F	36.87198809

High	Q9UJS0	Calcium-binding mitochondrial carrier protein Aralar2 OS=	36.84405685
High	Q8N1F7	Nuclear pore complex protein Nup93 OS=Homo sapiens O	36.66195807
High	P62195	26S proteasome regulatory subunit 8 OS=Homo sapiens O	36.58469168
High	P51149	Ras-related protein Rab-7a OS=Homo sapiens OX=9606 G	36.51310702
High	P35613	Basigin OS=Homo sapiens OX=9606 GN=BSG PE=1 SV=2	36.40922434
High	P43686	26S proteasome regulatory subunit 6B OS=Homo sapiens	36.36559455
High	O75694	Nuclear pore complex protein Nup155 OS=Homo sapiens	36.1596629
High	P08195	4F2 cell-surface antigen heavy chain OS=Homo sapiens OX	36.14252125
High	P39748	Flap endonuclease 1 OS=Homo sapiens OX=9606 GN=FEN	36.08617493
High	P33176	Kinesin-1 heavy chain OS=Homo sapiens OX=9606 GN=KIF	36.01807748
High	Q9H3P7	Golgi resident protein GCP60 OS=Homo sapiens OX=9606	35.80444927
High	Q53H12	Acylglycerol kinase, mitochondrial OS=Homo sapiens OX=	35.58453112
High	Q6P2Q9	Pre-mRNA-processing-splicing factor 8 OS=Homo sapiens	35.46767021
High	P62714	Serine/threonine-protein phosphatase 2A catalytic subun	35.40792154
High	Q9H0U4	Ras-related protein Rab-1B OS=Homo sapiens OX=9606 G	35.40121726
High	P32969	60S ribosomal protein L9 OS=Homo sapiens OX=9606 GN=	35.37346737
High	Q06210	Glutamine--fructose-6-phosphate aminotransferase [isom	35.32816788
High	Q9UMS4	Pre-mRNA-processing factor 19 OS=Homo sapiens OX=96	35.32318757
High	Q58FG0	Putative heat shock protein HSP 90-alpha A5 OS=Homo sa	35.1984227
High	P49419	Alpha-aminoadipic semialdehyde dehydrogenase OS=Hon	35.00700066
High	P18085	ADP-ribosylation factor 4 OS=Homo sapiens OX=9606 GN=	34.68608733
High	Q5JTZ9	Alanine--tRNA ligase, mitochondrial OS=Homo sapiens OX	34.68001211
High	P05388	60S acidic ribosomal protein P0 OS=Homo sapiens OX=96	34.58743485
High	P62701	40S ribosomal protein S4, X isoform OS=Homo sapiens OX	34.06610777
High	P53621	Coatomer subunit alpha OS=Homo sapiens OX=9606 GN=	33.9281775
High	P60228	Eukaryotic translation initiation factor 3 subunit E OS=Hon	33.88967511
High	O43252	Bifunctional 3'-phosphoadenosine 5'-phosphosulfate synt	33.86395334
High	Q9Y3F4	Serine-threonine kinase receptor-associated protein OS=H	33.74504477
High	P31948	Stress-induced-phosphoprotein 1 OS=Homo sapiens OX=9	33.32430037
High	O76094	Signal recognition particle subunit SRP72 OS=Homo sapie	33.27381016
High	Q02809	Procollagen-lysine,2-oxoglutarate 5-dioxygenase 1 OS=Ho	33.2648679
High	O43143	Pre-mRNA-splicing factor ATP-dependent RNA helicase DH	33.2581351
High	Q01813	ATP-dependent 6-phosphofructokinase, platelet type OS=	33.20170911
High	P08559	Pyruvate dehydrogenase E1 component subunit alpha, so	33.19092973
High	P30419	Glycylpeptide N-tetradecanoyltransferase 1 OS=Homo sap	33.15414022
High	P55084	Trifunctional enzyme subunit beta, mitochondrial OS=Hon	32.90953272
High	Q92973	Transportin-1 OS=Homo sapiens OX=9606 GN=TNPO1 PE=	32.39302239
High	P46940	Ras GTPase-activating-like protein IQGAP1 OS=Homo sapi	32.31963952
High	Q15029	116 kDa U5 small nuclear ribonucleoprotein component C	32.25140269
High	A6NHL2	Tubulin alpha chain-like 3 OS=Homo sapiens OX=9606 GN	31.98036211
High	P61106	Ras-related protein Rab-14 OS=Homo sapiens OX=9606 G	31.96225561
High	P62191	26S proteasome regulatory subunit 4 OS=Homo sapiens O	31.89903567
High	P07237	Protein disulfide-isomerase OS=Homo sapiens OX=9606 G	31.76670003
High	Q9UHB9	Signal recognition particle subunit SRP68 OS=Homo sapie	31.72720885
High	Q13451	Peptidyl-prolyl cis-trans isomerase FKBP5 OS=Homo sapie	31.63079125
High	P46782	40S ribosomal protein S5 OS=Homo sapiens OX=9606 GN=	30.99513183
High	Q96GX5	Serine/threonine-protein kinase greatwall OS=Homo sapie	30.99174867
High	Q06830	Peroxiredoxin-1 OS=Homo sapiens OX=9606 GN=PRDX1 P	30.87527755
High	O14929	Histone acetyltransferase type B catalytic subunit OS=Hon	30.85168131
High	O00148	ATP-dependent RNA helicase DDX39A OS=Homo sapiens O	30.83268048

High	O75955	Flotillin-1 OS=Homo sapiens OX=9606 GN=FLOT1 PE=1 SV	30.80499455
High	O95433	Activator of 90 kDa heat shock protein ATPase homolog 1	30.75338689
High	P11177	Pyruvate dehydrogenase E1 component subunit beta, mit	30.66535144
High	Q96FW1	Ubiquitin thioesterase OTUB1 OS=Homo sapiens OX=9606	30.49698583
High	P45974	Ubiquitin carboxyl-terminal hydrolase 5 OS=Homo sapien	30.42865178
High	P29401	Transketolase OS=Homo sapiens OX=9606 GN=TKT PE=1 S	30.34059202
High	P46459	Vesicle-fusing ATPase OS=Homo sapiens OX=9606 GN=NS	30.33516134
High	P15880	40S ribosomal protein S2 OS=Homo sapiens OX=9606 GN=	30.28211008
High	Q99829	Copine-1 OS=Homo sapiens OX=9606 GN=CPNE1 PE=1 SV	30.14828017
High	O76031	ATP-dependent Clp protease ATP-binding subunit clpX-like	30.09488695
High	Q8NC51	Plasminogen activator inhibitor 1 RNA-binding protein OS	30.09470576
High	P11940	Polyadenylate-binding protein 1 OS=Homo sapiens OX=96	29.88408498
High	O75306	NADH dehydrogenase [ubiquinone] iron-sulfur protein 2,	29.47838848
High	O75643	U5 small nuclear ribonucleoprotein 200 kDa helicase OS=	29.47230733
High	Q96T76	MMS19 nucleotide excision repair protein homolog OS=H	29.46979068
High	Q12905	Interleukin enhancer-binding factor 2 OS=Homo sapiens O	29.32758889
High	Q6UB35	Monofunctional C1-tetrahydrofolate synthase, mitochond	29.22912693
High	P62424	60S ribosomal protein L7a OS=Homo sapiens OX=9606 GN	29.21944561
High	P61011	Signal recognition particle 54 kDa protein OS=Homo sapie	29.17561174
High	Q96PK6	RNA-binding protein 14 OS=Homo sapiens OX=9606 GN=R	29.12894105
High	P31040	Succinate dehydrogenase [ubiquinone] flavoprotein subun	28.85813769
High	P51659	Peroxisomal multifunctional enzyme type 2 OS=Homo sap	28.60617468
High	Q9UNM6	26S proteasome non-ATPase regulatory subunit 13 OS=Ho	28.55818391
High	P31689	DnaJ homolog subfamily A member 1 OS=Homo sapiens O	28.42204295
High	P42765	3-ketoacyl-CoA thiolase, mitochondrial OS=Homo sapiens	28.3747606
High	Q9UQE7	Structural maintenance of chromosomes protein 3 OS=Ho	28.23744727
High	Q14C86	GTPase-activating protein and VPS9 domain-containing pr	28.18448177
High	Q15418	Ribosomal protein S6 kinase alpha-1 OS=Homo sapiens O)	28.10520195
High	P39023	60S ribosomal protein L3 OS=Homo sapiens OX=9606 GN=	28.04813996
High	O95394	Phosphoacetylglucosamine mutase OS=Homo sapiens OX	28.00422918
High	P26368	Splicing factor U2AF 65 kDa subunit OS=Homo sapiens OX	27.96052532
High	Q12931	Heat shock protein 75 kDa, mitochondrial OS=Homo sapie	27.74815717
High	O96008	Mitochondrial import receptor subunit TOM40 homolog O	27.6022116
High	P18621	60S ribosomal protein L17 OS=Homo sapiens OX=9606 GN	27.59788916
High	P35606	Coatomer subunit beta' OS=Homo sapiens OX=9606 GN=C	27.54891651
High	O60716	Catenin delta-1 OS=Homo sapiens OX=9606 GN=CTNND1	27.52458612
High	P30876	DNA-directed RNA polymerase II subunit RPB2 OS=Homo	27.29284765
High	P62136	Serine/threonine-protein phosphatase PP1-alpha catalytic	27.25940808
High	O94832	Unconventional myosin-Id OS=Homo sapiens OX=9606 GN	27.21401364
High	Q9Y3Z3	Deoxynucleoside triphosphate triphosphohydrolase SAMH	27.21273689
High	P10155	60 kDa SS-A/Ro ribonucleoprotein OS=Homo sapiens OX=	27.01988828
High	Q13547	Histone deacetylase 1 OS=Homo sapiens OX=9606 GN=HD	27.01731446
High	Q15021	Condensin complex subunit 1 OS=Homo sapiens OX=9606	26.94092658
High	P63151	Serine/threonine-protein phosphatase 2A 55 kDa regulato	26.93223137
High	A5YKK6	CCR4-NOT transcription complex subunit 1 OS=Homo sap	26.87085683
High	P06748	Nucleophosmin OS=Homo sapiens OX=9606 GN=NPM1 PI	26.75964807
High	P61163	Alpha-centractin OS=Homo sapiens OX=9606 GN=ACTR1A	26.71265519
High	P61026	Ras-related protein Rab-10 OS=Homo sapiens OX=9606 G	26.70949252
High	P27694	Replication protein A 70 kDa DNA-binding subunit OS=Hor	26.70234722
High	Q9Y450	HBS1-like protein OS=Homo sapiens OX=9606 GN=HBS1L	26.65206441

High	P09914	Interferon-induced protein with tetratricopeptide repeats	26.50923811
High	O43390	Heterogeneous nuclear ribonucleoprotein R OS=Homo sa	26.36859967
High	Q15046	Lysine--tRNA ligase OS=Homo sapiens OX=9606 GN=KARS	26.24703503
High	Q99536	Synaptic vesicle membrane protein VAT-1 homolog OS=H	26.20669612
High	Q99504	Eyes absent homolog 3 OS=Homo sapiens OX=9606 GN=E	25.9546134
High	P45880	Voltage-dependent anion-selective channel protein 2 OS=	25.84049202
High	Q9UBF2	Coatomer subunit gamma-2 OS=Homo sapiens OX=9606 (	25.74921557
High	O43592	Exportin-T OS=Homo sapiens OX=9606 GN=XPOT PE=1 SV	25.69361663
High	P18124	60S ribosomal protein L7 OS=Homo sapiens OX=9606 GN=	25.44036176
High	P08133	Annexin A6 OS=Homo sapiens OX=9606 GN=ANXA6 PE=1	25.42772765
High	P51148	Ras-related protein Rab-5C OS=Homo sapiens OX=9606 G	25.32321982
High	P54578	Ubiquitin carboxyl-terminal hydrolase 14 OS=Homo sapie	25.31554748
High	Q6P1J9	Parafibromin OS=Homo sapiens OX=9606 GN=CDC73 PE=	25.26272572
High	P49755	Transmembrane emp24 domain-containing protein 10 OS	25.22708616
High	O75533	Splicing factor 3B subunit 1 OS=Homo sapiens OX=9606 G	25.21584382
High	P09622	Dihydrolipoyl dehydrogenase, mitochondrial OS=Homo sa	25.156965
High	Q92597	Protein NDRG1 OS=Homo sapiens OX=9606 GN=NDRG1 P	25.10037116
High	Q16658	Fascin OS=Homo sapiens OX=9606 GN=FSCN1 PE=1 SV=3	25.06019354
High	O00116	Alkyldihydroxyacetonephosphate synthase, peroxisomal (	24.92692196
High	P43243	Matrin-3 OS=Homo sapiens OX=9606 GN=MATR3 PE=1 SV	24.73934218
High	Q9BW92	Threonine--tRNA ligase, mitochondrial OS=Homo sapiens	24.61634151
High	Q9NUU7	ATP-dependent RNA helicase DDX19A OS=Homo sapiens (	24.50307588
High	Q9BTV4	Transmembrane protein 43 OS=Homo sapiens OX=9606 G	24.35334923
High	P0DN79	Cystathionine beta-synthase-like protein OS=Homo sapier	24.30851587
High	O43615	Mitochondrial import inner membrane translocase subun	24.17992303
High	P23258	Tubulin gamma-1 chain OS=Homo sapiens OX=9606 GN=T	23.92849845
High	P63104	14-3-3 protein zeta/delta OS=Homo sapiens OX=9606 GN	23.89268005
High	P35998	26S proteasome regulatory subunit 7 OS=Homo sapiens O	23.87517054
High	P61158	Actin-related protein 3 OS=Homo sapiens OX=9606 GN=A	23.84586554
High	Q8NE71	ATP-binding cassette sub-family F member 1 OS=Homo sa	23.8453777
High	Q15758	Neutral amino acid transporter B(0) OS=Homo sapiens OX	23.70634734
High	Q9BQ52	Zinc phosphodiesterase ELAC protein 2 OS=Homo sapiens	23.45364051
High	O75083	WD repeat-containing protein 1 OS=Homo sapiens OX=96	23.32540982
High	P06280	Alpha-galactosidase A OS=Homo sapiens OX=9606 GN=GL	23.30300983
High	Q9H993	Protein-glutamate O-methyltransferase OS=Homo sapien	23.04752541
High	Q9Y6G9	Cytoplasmic dynein 1 light intermediate chain 1 OS=Homo	22.97762316
High	P20839	Inosine-5'-monophosphate dehydrogenase 1 OS=Homo sa	22.96523965
High	P15170	Eukaryotic peptide chain release factor GTP-binding subu	22.93175016
High	P49643	DNA primase large subunit OS=Homo sapiens OX=9606 G	22.79550185
High	P18754	Regulator of chromosome condensation OS=Homo sapier	22.79221162
High	P02768	Serum albumin OS=Homo sapiens OX=9606 GN=ALB PE=1	22.7654008
High	P62333	26S proteasome regulatory subunit 10B OS=Homo sapien	22.75852058
High	P52701	DNA mismatch repair protein Msh6 OS=Homo sapiens OX	22.74035223
High	Q9UQ16	Dynamin-3 OS=Homo sapiens OX=9606 GN=DNM3 PE=1 S	22.64189577
High	Q00325	Phosphate carrier protein, mitochondrial OS=Homo sapie	22.48181309
High	O14828	Secretory carrier-associated membrane protein 3 OS=Hor	22.38725643
High	Q9UIA9	Exportin-7 OS=Homo sapiens OX=9606 GN=XPO7 PE=1 SV	22.3866624
High	Q96P70	Importin-9 OS=Homo sapiens OX=9606 GN=IPO9 PE=1 SV	22.30634321
High	P78347	General transcription factor II-I OS=Homo sapiens OX=96(	22.25962971
High	Q9NQW7	Xaa-Pro aminopeptidase 1 OS=Homo sapiens OX=9606 GI	22.16614243

High	Q02978	Mitochondrial 2-oxoglutarate/malate carrier protein OS=I	22.05390675
High	P28340	DNA polymerase delta catalytic subunit OS=Homo sapiens	21.99166064
High	Q8TAT6	Nuclear protein localization protein 4 homolog OS=Homo	21.93525079
High	Q9Y678	Coatomer subunit gamma-1 OS=Homo sapiens OX=9606 (	21.92276875
High	P49189	4-trimethylaminobutyraldehyde dehydrogenase OS=Hom	21.9090708
High	Q53EL6	Programmed cell death protein 4 OS=Homo sapiens OX=9	21.84826646
High	Q93009	Ubiquitin carboxyl-terminal hydrolase 7 OS=Homo sapien	21.54790823
High	P27635	60S ribosomal protein L10 OS=Homo sapiens OX=9606 GI	21.51263412
High	Q9H4M9	EH domain-containing protein 1 OS=Homo sapiens OX=96	21.51128697
High	O95671	N-acetylserotonin O-methyltransferase-like protein OS=H	21.43832184
High	P62140	Serine/threonine-protein phosphatase PP1-beta catalytic	21.37455376
High	Q969Z0	FAST kinase domain-containing protein 4 OS=Homo sapie	21.36313621
High	Q09028	Histone-binding protein RBBP4 OS=Homo sapiens OX=960	21.2826302
High	O94874	E3 UFM1-protein ligase 1 OS=Homo sapiens OX=9606 GN	21.24099348
High	O00303	Eukaryotic translation initiation factor 3 subunit F OS=Hor	21.14677071
High	Q14694	Ubiquitin carboxyl-terminal hydrolase 10 OS=Homo sapie	21.14545871
High	P13647	Keratin, type II cytoskeletal 5 OS=Homo sapiens OX=9606	21.14504608
High	P18031	Tyrosine-protein phosphatase non-receptor type 1 OS=Hc	21.14189246
High	P05783	Keratin, type I cytoskeletal 18 OS=Homo sapiens OX=9606	21.09467842
High	Q9NR30	Nucleolar RNA helicase 2 OS=Homo sapiens OX=9606 GN=	20.99721201
High	P36776	Lon protease homolog, mitochondrial OS=Homo sapiens (	20.91070721
High	Q16555	Dihydropyrimidinase-related protein 2 OS=Homo sapiens	20.88100518
High	Q9NTJ3	Structural maintenance of chromosomes protein 4 OS=Hc	20.80928298
High	P00387	NADH-cytochrome b5 reductase 3 OS=Homo sapiens OX=	20.76544399
High	O00231	26S proteasome non-ATPase regulatory subunit 11 OS=Hc	20.71947243
High	O95747	Serine/threonine-protein kinase OSR1 OS=Homo sapiens (	20.71879015
High	Q8N1G4	Leucine-rich repeat-containing protein 47 OS=Homo sapie	20.64769351
High	Q13283	Ras GTPase-activating protein-binding protein 1 OS=Hom	20.6110282
High	P40937	Replication factor C subunit 5 OS=Homo sapiens OX=9606	20.59089433
High	P36873	Serine/threonine-protein phosphatase PP1-gamma cataly	20.54183127
High	Q9Y6C9	Mitochondrial carrier homolog 2 OS=Homo sapiens OX=9	20.53725522
High	Q9UQ80	Proliferation-associated protein 2G4 OS=Homo sapiens O	20.53008132
High	P55039	Developmentally-regulated GTP-binding protein 2 OS=Hor	20.52902695
High	O43795	Unconventional myosin-Ib OS=Homo sapiens OX=9606 GI	20.47385926
High	O60506	Heterogeneous nuclear ribonucleoprotein Q OS=Homo sa	20.42452271
High	Q13098	COP9 signalosome complex subunit 1 OS=Homo sapiens (	20.26315635
High	O95347	Structural maintenance of chromosomes protein 2 OS=Hc	20.2388075
High	Q07065	Cytoskeleton-associated protein 4 OS=Homo sapiens OX=	20.16299725
High	P56545	C-terminal-binding protein 2 OS=Homo sapiens OX=9606	20.15683339
High	P35232	Prohibitin OS=Homo sapiens OX=9606 GN=PHB PE=1 SV=1	20.0672958
High	P48444	Coatomer subunit delta OS=Homo sapiens OX=9606 GN=/	20.06251436
High	Q99497	Protein/nucleic acid deglycase DJ-1 OS=Homo sapiens OX	20.04474666
High	Q7Z2W4	Zinc finger CCCH-type antiviral protein 1 OS=Homo sapier	19.9753519
High	Q9BXP5	Serrate RNA effector molecule homolog OS=Homo sapien	19.94651817
High	Q9Y6M1	Insulin-like growth factor 2 mRNA-binding protein 2 OS=H	19.93215476
High	P46977	Dolichyl-diphosphooligosaccharide--protein glycosyltransf	19.87904452
High	Q96RP9	Elongation factor G, mitochondrial OS=Homo sapiens OX=	19.760154
High	P05091	Aldehyde dehydrogenase, mitochondrial OS=Homo sapier	19.69789918
High	P23526	Adenosylhomocysteinase OS=Homo sapiens OX=9606 GN	19.642426
High	Q13363	C-terminal-binding protein 1 OS=Homo sapiens OX=9606	19.57477692

High	P17858	ATP-dependent 6-phosphofructokinase, liver type OS=Hoi	19.56896506
High	P62979	Ubiquitin-40S ribosomal protein S27a OS=Homo sapiens (	19.55680372
High	O94906	Pre-mRNA-processing factor 6 OS=Homo sapiens OX=960	19.49325291
High	O14654	Insulin receptor substrate 4 OS=Homo sapiens OX=9606 G	19.4460861
High	Q13151	Heterogeneous nuclear ribonucleoprotein A0 OS=Homo s	19.44161825
High	Q96RG2	PAS domain-containing serine/threonine-protein kinase C	19.41048152
High	P26196	Probable ATP-dependent RNA helicase DDX6 OS=Homo sa	19.3852614
High	Q9H3U1	Protein unc-45 homolog A OS=Homo sapiens OX=9606 GI	19.28652191
High	P52294	Importin subunit alpha-5 OS=Homo sapiens OX=9606 GN=	19.27031518
High	P62241	40S ribosomal protein S8 OS=Homo sapiens OX=9606 GN=	19.23852027
High	O15111	Inhibitor of nuclear factor kappa-B kinase subunit alpha O	19.22493662
High	P28288	ATP-binding cassette sub-family D member 3 OS=Homo sa	19.20502412
High	P62805	Histone H4 OS=Homo sapiens OX=9606 GN=HIST1H4A PE	19.14906295
High	Q9P035	Very-long-chain (3R)-3-hydroxyacyl-CoA dehydratase 3 OS	19.11354943
High	P30041	Peroxiredoxin-6 OS=Homo sapiens OX=9606 GN=PRDX6 F	19.0927603
High	P62258	14-3-3 protein epsilon OS=Homo sapiens OX=9606 GN=YV	18.92495013
High	Q13895	Bystin OS=Homo sapiens OX=9606 GN=BYSL PE=1 SV=3	18.90524749
High	Q16222	UDP-N-acetylhexosamine pyrophosphorylase OS=Homo s	18.8328757
High	O15294	UDP-N-acetylglucosamine--peptide N-acetylglucosaminylt	18.811273
High	Q14498	RNA-binding protein 39 OS=Homo sapiens OX=9606 GN=F	18.77334871
High	P55036	26S proteasome non-ATPase regulatory subunit 4 OS=Hoi	18.71023952
High	P06730	Eukaryotic translation initiation factor 4E OS=Homo sapie	18.61058236
High	Q99873	Protein arginine N-methyltransferase 1 OS=Homo sapiens	18.56214875
High	O75390	Citrate synthase, mitochondrial OS=Homo sapiens OX=96	18.54863779
High	Q15008	26S proteasome non-ATPase regulatory subunit 6 OS=Hoi	18.52420994
High	P11172	Uridine 5'-monophosphate synthase OS=Homo sapiens O	18.5193311
High	A0A075B6	Immunoglobulin kappa variable 2D-29 OS=Homo sapiens	18.5039633
High	P61019	Ras-related protein Rab-2A OS=Homo sapiens OX=9606 G	18.49870873
High	Q9BXW7	Haloacid dehalogenase-like hydrolase domain-containing	18.43916684
High	P02533	Keratin, type I cytoskeletal 14 OS=Homo sapiens OX=9606	18.3432116
High	P13489	Ribonuclease inhibitor OS=Homo sapiens OX=9606 GN=RI	18.30773535
High	P30101	Protein disulfide-isomerase A3 OS=Homo sapiens OX=960	18.2674419
High	Q9GZZ9	Ubiquitin-like modifier-activating enzyme 5 OS=Homo sap	18.22946107
High	Q96IJ6	Mannose-1-phosphate guanyltransferase alpha OS=Homo	18.22924731
High	P38117	Electron transfer flavoprotein subunit beta OS=Homo sap	18.22352219
High	Q04760	Lactoylglutathione lyase OS=Homo sapiens OX=9606 GN=	18.18885974
High	Q15019	Septin-2 OS=Homo sapiens OX=9606 GN=SEPT2 PE=1 SV=	18.17732381
High	Q8TEX9	Importin-4 OS=Homo sapiens OX=9606 GN=IPO4 PE=1 SV	18.12659361
High	P53396	ATP-citrate synthase OS=Homo sapiens OX=9606 GN=ACL	18.11754031
High	Q05193	Dynamin-1 OS=Homo sapiens OX=9606 GN=DNM1 PE=1 S	18.10660925
High	Q8NBF2	NHL repeat-containing protein 2 OS=Homo sapiens OX=96	18.08034135
High	Q5TDH0	Protein DDI1 homolog 2 OS=Homo sapiens OX=9606 GN=	17.99482262
High	O95376	E3 ubiquitin-protein ligase ARIH2 OS=Homo sapiens OX=9	17.98937235
High	Q92667	A-kinase anchor protein 1, mitochondrial OS=Homo sapie	17.98034209
High	Q93008	Probable ubiquitin carboxyl-terminal hydrolase FAF-X OS=	17.82344074
High	Q9BZZ5	Apoptosis inhibitor 5 OS=Homo sapiens OX=9606 GN=API	17.79700793
High	A0AVT1	Ubiquitin-like modifier-activating enzyme 6 OS=Homo sap	17.70693502
High	P49593	Protein phosphatase 1F OS=Homo sapiens OX=9606 GN=I	17.6609318
High	O00425	Insulin-like growth factor 2 mRNA-binding protein 3 OS=H	17.63597113
High	O75746	Calcium-binding mitochondrial carrier protein Aralar1 OS=	17.62490351

High	A1L0T0	Acetolactate synthase-like protein OS=Homo sapiens OX=	17.60229801
High	P38606	V-type proton ATPase catalytic subunit A OS=Homo sapie	17.54269066
High	P51570	Galactokinase OS=Homo sapiens OX=9606 GN=GALK1 PE=	17.52019703
High	P58107	Epiplakin OS=Homo sapiens OX=9606 GN=EPPK1 PE=1 SV	17.45575378
High	O00469	Procollagen-lysine,2-oxoglutarate 5-dioxygenase 2 OS=Hc	17.41114142
High	P24941	Cyclin-dependent kinase 2 OS=Homo sapiens OX=9606 GI	17.37245663
High	Q9BRX2	Protein pelota homolog OS=Homo sapiens OX=9606 GN=I	17.35404212
High	P40926	Malate dehydrogenase, mitochondrial OS=Homo sapiens	17.23691977
High	Q9Y4C2	TRPM8 channel-associated factor 1 OS=Homo sapiens OX	17.17242286
High	Q9P289	Serine/threonine-protein kinase 26 OS=Homo sapiens OX	17.1433719
High	O94905	Erlin-2 OS=Homo sapiens OX=9606 GN=ERLIN2 PE=1 SV=1	17.10054376
High	P62917	60S ribosomal protein L8 OS=Homo sapiens OX=9606 GN=	16.96321373
High	Q9H845	Acyl-CoA dehydrogenase family member 9, mitochondrial	16.89371063
High	Q14315	Filamin-C OS=Homo sapiens OX=9606 GN=FLNC PE=1 SV=	16.88418115
High	Q12906	Interleukin enhancer-binding factor 3 OS=Homo sapiens C	16.87329488
High	Q6DKJ4	Nucleoredoxin OS=Homo sapiens OX=9606 GN=NXN PE=1	16.81838933
High	O15371	Eukaryotic translation initiation factor 3 subunit D OS=Ho	16.78273887
High	Q96D46	60S ribosomal export protein NMD3 OS=Homo sapiens O.	16.76182946
High	O60814	Histone H2B type 1-K OS=Homo sapiens OX=9606 GN=HIS	16.73429188
High	Q99956	Dual specificity protein phosphatase 9 OS=Homo sapiens	16.66575243
High	P49005	DNA polymerase delta subunit 2 OS=Homo sapiens OX=96	16.65572297
High	Q14152	Eukaryotic translation initiation factor 3 subunit A OS=Hoi	16.62988376
High	Q8N684	Cleavage and polyadenylation specificity factor subunit 7	16.62359558
High	Q9BVP2	Guanine nucleotide-binding protein-like 3 OS=Homo sapie	16.56104182
High	P61247	40S ribosomal protein S3a OS=Homo sapiens OX=9606 GI	16.4205613
High	O00232	26S proteasome non-ATPase regulatory subunit 12 OS=Hc	16.36237974
High	P51617	Interleukin-1 receptor-associated kinase 1 OS=Homo sapi	16.3234028
High	Q92900	Regulator of nonsense transcripts 1 OS=Homo sapiens OX	16.22871142
High	Q16822	Phosphoenolpyruvate carboxykinase [GTP], mitochondria	16.22165803
High	Q9BWF3	RNA-binding protein 4 OS=Homo sapiens OX=9606 GN=RI	16.19941211
High	Q6YN16	Hydroxysteroid dehydrogenase-like protein 2 OS=Homo s	16.17295036
High	Q9P258	Protein RCC2 OS=Homo sapiens OX=9606 GN=RCC2 PE=1	16.16055012
High	O95340	Bifunctional 3'-phosphoadenosine 5'-phosphosulfate synt	16.11364597
High	P49642	DNA primase small subunit OS=Homo sapiens OX=9606 G	16.08534238
High	Q9Y697	Cysteine desulfurase, mitochondrial OS=Homo sapiens O)	16.05188816
High	P13010	X-ray repair cross-complementing protein 5 OS=Homo sa	15.94329532
High	P00403	Cytochrome c oxidase subunit 2 OS=Homo sapiens OX=96	15.92896831
High	O94925	Glutaminase kidney isoform, mitochondrial OS=Homo sap	15.87851723
High	Q15717	ELAV-like protein 1 OS=Homo sapiens OX=9606 GN=ELAV	15.80827704
High	Q03252	Lamin-B2 OS=Homo sapiens OX=9606 GN=LMNB2 PE=1 S	15.79933835
High	Q00341	Vigilin OS=Homo sapiens OX=9606 GN=HDLBP PE=1 SV=2	15.73749004
High	O15269	Serine palmitoyltransferase 1 OS=Homo sapiens OX=9606	15.71147483
High	P55010	Eukaryotic translation initiation factor 5 OS=Homo sapien	15.58960105
High	Q9BXJ9	N-alpha-acetyltransferase 15, NatA auxiliary subunit OS=H	15.54091118
High	P47985	Cytochrome b-c1 complex subunit Rieske, mitochondrial (	15.48875037
High	Q13409	Cytoplasmic dynein 1 intermediate chain 2 OS=Homo sap	15.46988188
High	Q9Y312	Protein AAR2 homolog OS=Homo sapiens OX=9606 GN=A	15.43885646
High	P25325	3-mercaptopyruvate sulfurtransferase OS=Homo sapiens	15.29086699
High	Q96F86	Enhancer of mRNA-decapping protein 3 OS=Homo sapien	15.25039928
High	Q9C0C9	(E3-independent) E2 ubiquitin-conjugating enzyme OS=Hc	15.2427039

High	Q15084	Protein disulfide-isomerase A6 OS=Homo sapiens OX=960	15.21263783
High	P16152	Carbonyl reductase [NADPH] 1 OS=Homo sapiens OX=960	15.20405399
High	Q8TD19	Serine/threonine-protein kinase Nek9 OS=Homo sapiens (	15.18448891
High	Q9Y5L0	Transportin-3 OS=Homo sapiens OX=9606 GN=TNPO3 PE=	15.14566576
High	O60884	DnaJ homolog subfamily A member 2 OS=Homo sapiens (	15.11797531
High	Q9NZN3	EH domain-containing protein 3 OS=Homo sapiens OX=96	15.05772858
High	P61981	14-3-3 protein gamma OS=Homo sapiens OX=9606 GN=Y\	15.05729689
High	Q13347	Eukaryotic translation initiation factor 3 subunit I OS=Hon	15.04863642
High	P52888	Thimet oligopeptidase OS=Homo sapiens OX=9606 GN=TI	15.03705744
High	Q7Z2T5	TRMT1-like protein OS=Homo sapiens OX=9606 GN=TRM	15.03614366
High	Q8IUI8	Cytokine receptor-like factor 3 OS=Homo sapiens OX=960	14.96029127
High	P13674	Prolyl 4-hydroxylase subunit alpha-1 OS=Homo sapiens O	14.92030497
High	Q8TC12	Retinol dehydrogenase 11 OS=Homo sapiens OX=9606 GN	14.91775681
High	P46783	40S ribosomal protein S10 OS=Homo sapiens OX=9606 GI	14.91595507
High	Q14739	Lamin-B receptor OS=Homo sapiens OX=9606 GN=LBR PE	14.88993333
High	P53985	Monocarboxylate transporter 1 OS=Homo sapiens OX=96	14.85331167
High	P05198	Eukaryotic translation initiation factor 2 subunit 1 OS=Hor	14.84762675
High	P67809	Nuclease-sensitive element-binding protein 1 OS=Homo s	14.78905863
High	Q04323	UBX domain-containing protein 1 OS=Homo sapiens OX=9	14.76918214
High	P53007	Tricarboxylate transport protein, mitochondrial OS=Homo	14.70965965
High	Q9NVE7	Pantothenate kinase 4 OS=Homo sapiens OX=9606 GN=P/	14.67367167
High	Q8IUF8	Ribosomal oxygenase 2 OS=Homo sapiens OX=9606 GN=F	14.66416824
High	O43684	Mitotic checkpoint protein BUB3 OS=Homo sapiens OX=9	14.59405982
High	O43172	U4/U6 small nuclear ribonucleoprotein Prp4 OS=Homo sa	14.50874884
High	Q09161	Nuclear cap-binding protein subunit 1 OS=Homo sapiens (	14.48591132
High	O75821	Eukaryotic translation initiation factor 3 subunit G OS=Ho	14.45384885
High	Q92930	Ras-related protein Rab-8B OS=Homo sapiens OX=9606 G	14.36604348
High	Q92797	Symplekin OS=Homo sapiens OX=9606 GN=SYMPK PE=1 S	14.36546968
High	Q92769	Histone deacetylase 2 OS=Homo sapiens OX=9606 GN=HI	14.35873078
High	Q9UNE7	E3 ubiquitin-protein ligase CHIP OS=Homo sapiens OX=96	14.30328149
High	P62244	40S ribosomal protein S15a OS=Homo sapiens OX=9606 G	14.27740071
High	O60488	Long-chain-fatty-acid--CoA ligase 4 OS=Homo sapiens OX=	14.24232715
High	Q8N766	ER membrane protein complex subunit 1 OS=Homo sapie	14.23000615
High	Q9BSJ8	Extended synaptotagmin-1 OS=Homo sapiens OX=9606 G	14.21433507
High	P31946	14-3-3 protein beta/alpha OS=Homo sapiens OX=9606 GN	14.15617676
High	Q9H0C8	Integrin-linked kinase-associated serine/threonine phosph	14.04991677
High	Q12874	Splicing factor 3A subunit 3 OS=Homo sapiens OX=9606 G	13.99335801
High	Q92615	La-related protein 4B OS=Homo sapiens OX=9606 GN=LAI	13.84877647
High	Q9Y5M8	Signal recognition particle receptor subunit beta OS=Hom	13.84105705
High	P27348	14-3-3 protein theta OS=Homo sapiens OX=9606 GN=YWI	13.81266304
High	P62269	40S ribosomal protein S18 OS=Homo sapiens OX=9606 GI	13.80119209
High	Q06124	Tyrosine-protein phosphatase non-receptor type 11 OS=H	13.78905964
High	P52789	Hexokinase-2 OS=Homo sapiens OX=9606 GN=HK2 PE=1 S	13.78849892
High	Q5JPE7	Nodal modulator 2 OS=Homo sapiens OX=9606 GN=NOM	13.71377408
High	Q9BQ67	Glutamate-rich WD repeat-containing protein 1 OS=Homo	13.64524651
High	Q53GQ0	Very-long-chain 3-oxoacyl-CoA reductase OS=Homo sapie	13.60733293
High	Q8NBJ5	Procollagen galactosyltransferase 1 OS=Homo sapiens OX	13.59807358
High	Q8NI60	Atypical kinase COQ8A, mitochondrial OS=Homo sapiens (	13.55742544
High	P11498	Pyruvate carboxylase, mitochondrial OS=Homo sapiens O	13.5552288
High	P22234	Multifunctional protein ADE2 OS=Homo sapiens OX=9606	13.52483764

High	Q9Y4W6	AFG3-like protein 2 OS=Homo sapiens OX=9606 GN=AFG3	13.49701433
High	Q9NR31	GTP-binding protein SAR1a OS=Homo sapiens OX=9606 G	13.45867835
High	O95573	Long-chain-fatty-acid--CoA ligase 3 OS=Homo sapiens OX=	13.41991082
High	Q92917	G-patch domain and KOW motifs-containing protein OS=H	13.38027582
High	P42025	Beta-centractin OS=Homo sapiens OX=9606 GN=ACTR1B	13.37998261
High	Q7L0Y3	tRNA methyltransferase 10 homolog C OS=Homo sapiens	13.31574193
High	Q8IXI2	Mitochondrial Rho GTPase 1 OS=Homo sapiens OX=9606	13.30420587
High	Q8NF37	Lysophosphatidylcholine acyltransferase 1 OS=Homo sapi	13.12053593
High	P57737	Coronin-7 OS=Homo sapiens OX=9606 GN=CORO7 PE=1 S	13.0940837
High	Q9H9P8	L-2-hydroxyglutarate dehydrogenase, mitochondrial OS=H	13.05983592
High	Q9NRF8	CTP synthase 2 OS=Homo sapiens OX=9606 GN=CTPS2 PE	12.96028388
High	O75152	Zinc finger CCCH domain-containing protein 11A OS=Hom	12.9499376
High	P11216	Glycogen phosphorylase, brain form OS=Homo sapiens O.	12.87259187
High	O60684	Importin subunit alpha-7 OS=Homo sapiens OX=9606 GN=	12.86092565
High	P04075	Fructose-bisphosphate aldolase A OS=Homo sapiens OX=9	12.85095287
High	Q29RF7	Sister chromatid cohesion protein PDS5 homolog A OS=H	12.81662591
High	O75155	Cullin-associated NEDD8-dissociated protein 2 OS=Homo	12.81030596
High	P46778	60S ribosomal protein L21 OS=Homo sapiens OX=9606 GN	12.77190744
High	Q5T6V5	Queuosine salvage protein OS=Homo sapiens OX=9606 G	12.71832042
High	P06744	Glucose-6-phosphate isomerase OS=Homo sapiens OX=96	12.67688037
High	P04637	Cellular tumor antigen p53 OS=Homo sapiens OX=9606 G	12.65845348
High	O75439	Mitochondrial-processing peptidase subunit beta OS=Hon	12.52455135
High	P46781	40S ribosomal protein S9 OS=Homo sapiens OX=9606 GN=	12.48779164
High	Q9UHD8	Septin-9 OS=Homo sapiens OX=9606 GN=SEPT9 PE=1 SV=	12.48452518
High	O00299	Chloride intracellular channel protein 1 OS=Homo sapiens	12.47407245
High	Q9NPQ8	Synembryn-A OS=Homo sapiens OX=9606 GN=RIC8A PE=1	12.47113108
High	P34932	Heat shock 70 kDa protein 4 OS=Homo sapiens OX=9606	12.4697613
High	P61619	Protein transport protein Sec61 subunit alpha isoform 1 C	12.43851692
High	Q15031	Probable leucine--tRNA ligase, mitochondrial OS=Homo sa	12.40655658
High	Q9BPX3	Condensin complex subunit 3 OS=Homo sapiens OX=9606	12.35400963
High	Q9NVH2	Integrator complex subunit 7 OS=Homo sapiens OX=9606	12.30752218
High	P20339	Ras-related protein Rab-5A OS=Homo sapiens OX=9606 G	12.19256087
High	P61160	Actin-related protein 2 OS=Homo sapiens OX=9606 GN=A	12.13233296
High	O75477	Erlin-1 OS=Homo sapiens OX=9606 GN=ERLIN1 PE=1 SV=1	12.13027081
High	Q13162	Peroxiredoxin-4 OS=Homo sapiens OX=9606 GN=PRDX4 F	11.9970948
High	Q9H2U2	Inorganic pyrophosphatase 2, mitochondrial OS=Homo sa	11.99335903
High	O14879	Interferon-induced protein with tetratricopeptide repeats	11.93458046
High	P51114	Fragile X mental retardation syndrome-related protein 1 (	11.93444288
High	E9PAV3	Nascent polypeptide-associated complex subunit alpha, m	11.91955993
High	Q96A33	Coiled-coil domain-containing protein 47 OS=Homo sapie	11.88916789
High	Q9P2R7	Succinate--CoA ligase [ADP-forming] subunit beta, mitoch	11.88538993
High	Q9Y6E2	Basic leucine zipper and W2 domain-containing protein 2	11.80146408
High	Q71RC2	La-related protein 4 OS=Homo sapiens OX=9606 GN=LARI	11.79357413
High	P21796	Voltage-dependent anion-selective channel protein 1 OS=	11.77606224
High	P02545	Prelamin-A/C OS=Homo sapiens OX=9606 GN=LMNA PE=1	11.74650963
High	Q95604	HLA class I histocompatibility antigen, Cw-17 alpha chain (	11.72453431
High	Q92696	Geranylgeranyl transferase type-2 subunit alpha OS=Hom	11.62822807
High	P61224	Ras-related protein Rap-1b OS=Homo sapiens OX=9606 G	11.62702177
High	Q96KR1	Zinc finger RNA-binding protein OS=Homo sapiens OX=96	11.60318349
High	P53041	Serine/threonine-protein phosphatase 5 OS=Homo sapier	11.4904102

High	Q6NUK1	Calcium-binding mitochondrial carrier protein SCaMC-1 O	11.45898223
High	O60841	Eukaryotic translation initiation factor 5B OS=Homo sapie	11.40036535
High	O14981	TATA-binding protein-associated factor 172 OS=Homo sap	11.35891212
High	P20340	Ras-related protein Rab-6A OS=Homo sapiens OX=9606 G	11.34285982
High	Q969X5	Endoplasmic reticulum-Golgi intermediate compartment	11.34220199
High	Q15006	ER membrane protein complex subunit 2 OS=Homo sapie	11.31387776
High	Q5JPH6	Probable glutamate--tRNA ligase, mitochondrial OS=Hom	11.31191086
High	P40763	Signal transducer and activator of transcription 3 OS=Hom	11.28461597
High	P32119	Peroxiredoxin-2 OS=Homo sapiens OX=9606 GN=PRDX2 P	11.28425027
High	Q15393	Splicing factor 3B subunit 3 OS=Homo sapiens OX=9606 G	11.23917428
High	P60660	Myosin light polypeptide 6 OS=Homo sapiens OX=9606 G	11.21359597
High	Q14203	Dynactin subunit 1 OS=Homo sapiens OX=9606 GN=DCTN	11.19605906
High	P31350	Ribonucleoside-diphosphate reductase subunit M2 OS=H	11.15719575
High	Q9UKF6	Cleavage and polyadenylation specificity factor subunit 3	11.15248238
High	O00154	Cytosolic acyl coenzyme A thioester hydrolase OS=Homo	11.06301879
High	O15355	Protein phosphatase 1G OS=Homo sapiens OX=9606 GN=	11.01129344
High	P26373	60S ribosomal protein L13 OS=Homo sapiens OX=9606 GI	10.99606915
High	P61006	Ras-related protein Rab-8A OS=Homo sapiens OX=9606 G	10.96796656
High	O43837	Isocitrate dehydrogenase [NAD] subunit beta, mitochondr	10.94116716
High	P37802	Transgelin-2 OS=Homo sapiens OX=9606 GN=TAGLN2 PE=	10.88618338
High	O75663	TIP41-like protein OS=Homo sapiens OX=9606 GN=TIPRL	10.86057223
High	O95232	Luc7-like protein 3 OS=Homo sapiens OX=9606 GN=LUC7I	10.85936627
High	P30050	60S ribosomal protein L12 OS=Homo sapiens OX=9606 GI	10.67190798
High	Q8WVC6	Dephospho-CoA kinase domain-containing protein OS=Ho	10.60352724
High	Q9BYW2	Histone-lysine N-methyltransferase SETD2 OS=Homo sapi	10.56193621
High	P83731	60S ribosomal protein L24 OS=Homo sapiens OX=9606 GI	10.54581177
High	P68400	Casein kinase II subunit alpha OS=Homo sapiens OX=9606	10.51444743
High	P09913	Interferon-induced protein with tetratricopeptide repeats	10.50484173
High	Q9BV44	THUMP domain-containing protein 3 OS=Homo sapiens O	10.50224604
High	Q96KP4	Cytosolic non-specific dipeptidase OS=Homo sapiens OX=	10.45889377
High	P19367	Hexokinase-1 OS=Homo sapiens OX=9606 GN=HK1 PE=1 S	10.44960017
High	Q9UHB6	LIM domain and actin-binding protein 1 OS=Homo sapien	10.39046236
High	Q6P996	Pyridoxal-dependent decarboxylase domain-containing pr	10.36204118
High	Q14671	Pumilio homolog 1 OS=Homo sapiens OX=9606 GN=PUM	10.34873108
High	P56134	ATP synthase subunit f, mitochondrial OS=Homo sapiens	10.34135212
High	P09661	U2 small nuclear ribonucleoprotein A' OS=Homo sapiens	10.33585153
High	Q9HAV4	Exportin-5 OS=Homo sapiens OX=9606 GN=XPO5 PE=1 SV	10.29956405
High	P62249	40S ribosomal protein S16 OS=Homo sapiens OX=9606 GI	10.27571355
High	Q9UL25	Ras-related protein Rab-21 OS=Homo sapiens OX=9606 G	10.24045264
High	Q9NRG9	Aladin OS=Homo sapiens OX=9606 GN=AAAS PE=1 SV=1	10.23099209
High	Q9Y6B6	GTP-binding protein SAR1b OS=Homo sapiens OX=9606 G	10.20738041
High	P57088	Transmembrane protein 33 OS=Homo sapiens OX=9606 G	10.18836015
High	O43707	Alpha-actinin-4 OS=Homo sapiens OX=9606 GN=ACTN4 P	10.16999709
High	Q16795	NADH dehydrogenase [ubiquinone] 1 alpha subcomplex s	10.16108936
High	Q9Y5A9	YTH domain-containing family protein 2 OS=Homo sapien	10.13160037
High	Q02543	60S ribosomal protein L18a OS=Homo sapiens OX=9606 G	10.0778478
High	Q9BQA1	Methylosome protein 50 OS=Homo sapiens OX=9606 GN=	10.07254952
High	Q9Y5Q8	General transcription factor 3C polypeptide 5 OS=Homo s	10.06616587
High	P24539	ATP synthase F(0) complex subunit B1, mitochondrial OS=	10.06254396
High	P40429	60S ribosomal protein L13a OS=Homo sapiens OX=9606 G	10.03228348

High	Q12834	Cell division cycle protein 20 homolog OS=Homo sapiens (	10.01982331
High	Q15020	Squamous cell carcinoma antigen recognized by T-cells 3 (	10.0063374
High	Q96S52	GPI transamidase component PIG-S OS=Homo sapiens OX	9.966248637
High	O43264	Centromere/kinetochore protein zw10 homolog OS=Hom	9.950619616
High	P41091	Eukaryotic translation initiation factor 2 subunit 3 OS=Hor	9.932176201
High	Q13564	NEDD8-activating enzyme E1 regulatory subunit OS=Hom	9.893337169
High	P61254	60S ribosomal protein L26 OS=Homo sapiens OX=9606 GI	9.882063707
High	Q8WUA2	Peptidyl-prolyl cis-trans isomerase-like 4 OS=Homo sapier	9.881355417
High	P16278	Beta-galactosidase OS=Homo sapiens OX=9606 GN=GLB1	9.762933243
High	Q9Y305	Acyl-coenzyme A thioesterase 9, mitochondrial OS=Homo	9.745275694
High	P14635	G2/mitotic-specific cyclin-B1 OS=Homo sapiens OX=9606	9.707389769
High	P50416	Carnitine O-palmitoyltransferase 1, liver isoform OS=Hom	9.620224402
High	Q52LJ0	Protein FAM98B OS=Homo sapiens OX=9606 GN=FAM98I	9.59901879
High	P62263	40S ribosomal protein S14 OS=Homo sapiens OX=9606 GI	9.593589276
High	P23528	Cofilin-1 OS=Homo sapiens OX=9606 GN=CFL1 PE=1 SV=3	9.554182103
High	P62277	40S ribosomal protein S13 OS=Homo sapiens OX=9606 GI	9.505349789
High	P61313	60S ribosomal protein L15 OS=Homo sapiens OX=9606 GI	9.504549469
High	P84098	60S ribosomal protein L19 OS=Homo sapiens OX=9606 GI	9.486820636
High	Q13505	Metaxin-1 OS=Homo sapiens OX=9606 GN=MTX1 PE=1 SV	9.3723587
High	Q15286	Ras-related protein Rab-35 OS=Homo sapiens OX=9606 G	9.371892072
High	O15344	E3 ubiquitin-protein ligase Midline-1 OS=Homo sapiens O	9.316009162
High	Q9UI26	Importin-11 OS=Homo sapiens OX=9606 GN=IPO11 PE=1	9.289379307
High	Q9NUQ8	ATP-binding cassette sub-family F member 3 OS=Homo sa	9.280298204
High	Q13188	Serine/threonine-protein kinase 3 OS=Homo sapiens OX=	9.277904018
High	P49959	Double-strand break repair protein MRE11 OS=Homo sap	9.241556248
High	Q7L1Q6	Basic leucine zipper and W2 domain-containing protein 1	9.229271691
High	Q96P47	Arf-GAP with GTPase, ANK repeat and PH domain-contain	9.209724006
High	P25786	Proteasome subunit alpha type-1 OS=Homo sapiens OX=9	9.204446346
High	Q9NTK5	Obg-like ATPase 1 OS=Homo sapiens OX=9606 GN=OLA1	9.201876904
High	Q8WX92	Negative elongation factor B OS=Homo sapiens OX=9606	9.198805202
High	Q9BVI4	Nucleolar complex protein 4 homolog OS=Homo sapiens (	9.189137909
High	Q13613	Myotubularin-related protein 1 OS=Homo sapiens OX=96	9.17219395
High	Q92598	Heat shock protein 105 kDa OS=Homo sapiens OX=9606 (	9.158582525
High	P31942	Heterogeneous nuclear ribonucleoprotein H3 OS=Homo s	9.119262759
High	P50213	Isocitrate dehydrogenase [NAD] subunit alpha, mitochonc	9.116220804
High	Q13617	Cullin-2 OS=Homo sapiens OX=9606 GN=CUL2 PE=1 SV=2	9.035427683
High	Q9NVH1	DnaJ homolog subfamily C member 11 OS=Homo sapiens	9.033783603
High	O60313	Dynamin-like 120 kDa protein, mitochondrial OS=Homo s	9.01311206
High	Q14103	Heterogeneous nuclear ribonucleoprotein D0 OS=Homo s	9.00551031
High	Q8TEU7	Rap guanine nucleotide exchange factor 6 OS=Homo sapi	9.00305021
High	Q3LXA3	Triokinase/FMN cyclase OS=Homo sapiens OX=9606 GN=	8.965287325
High	Q07666	KH domain-containing, RNA-binding, signal transduction-a	8.963820808
High	P32189	Glycerol kinase OS=Homo sapiens OX=9606 GN=GK PE=1	8.932840702
High	P49321	Nuclear autoantigenic sperm protein OS=Homo sapiens O	8.8126561
High	Q9HDC9	Adipocyte plasma membrane-associated protein OS=Hom	8.761859637
High	P06737	Glycogen phosphorylase, liver form OS=Homo sapiens OX	8.751803088
High	P62750	60S ribosomal protein L23a OS=Homo sapiens OX=9606 G	8.746386814
High	Q16630	Cleavage and polyadenylation specificity factor subunit 6	8.741241974
High	P48739	Phosphatidylinositol transfer protein beta isoform OS=Ho	8.716456495
High	Q9HCC0	Methylcrotonoyl-CoA carboxylase beta chain, mitochondr	8.710119724

High	Q5W0B1	RING finger protein 219 OS=Homo sapiens OX=9606 GN=I	8.693822893
High	Q07020	60S ribosomal protein L18 OS=Homo sapiens OX=9606 GI	8.677591075
High	Q9P287	BRCA2 and CDKN1A-interacting protein OS=Homo sapiens	8.66941652
High	P61764	Syntaxin-binding protein 1 OS=Homo sapiens OX=9606 GI	8.649947381
High	P24534	Elongation factor 1-beta OS=Homo sapiens OX=9606 GN=	8.606924128
High	Q12849	G-rich sequence factor 1 OS=Homo sapiens OX=9606 GN=	8.597399416
High	O15027	Protein transport protein Sec16A OS=Homo sapiens OX=9	8.589699133
High	P12532	Creatine kinase U-type, mitochondrial OS=Homo sapiens (	8.588181894
High	P29992	Guanine nucleotide-binding protein subunit alpha-11 OS=	8.586031488
High	Q9H857	5'-nucleotidase domain-containing protein 2 OS=Homo sa	8.568433162
High	Q3MHD2	Protein LSM12 homolog OS=Homo sapiens OX=9606 GN=	8.563143355
High	P46777	60S ribosomal protein L5 OS=Homo sapiens OX=9606 GN=	8.545201435
High	P47756	F-actin-capping protein subunit beta OS=Homo sapiens O)	8.5350731
High	P56537	Eukaryotic translation initiation factor 6 OS=Homo sapien	8.492192416
High	O15270	Serine palmitoyltransferase 2 OS=Homo sapiens OX=9606	8.484685936
High	P50402	Emerin OS=Homo sapiens OX=9606 GN=EMD PE=1 SV=1	8.475214551
High	Q92621	Nuclear pore complex protein Nup205 OS=Homo sapiens	8.406689728
High	Q2TAL8	Glutamine-rich protein 1 OS=Homo sapiens OX=9606 GN=	8.39823493
High	Q96N67	Dedicator of cytokinesis protein 7 OS=Homo sapiens OX=9	8.395783447
High	O00505	Importin subunit alpha-4 OS=Homo sapiens OX=9606 GN=	8.377794269
High	Q16531	DNA damage-binding protein 1 OS=Homo sapiens OX=960	8.370794139
High	Q9C0B1	Alpha-ketoglutarate-dependent dioxygenase FTO OS=Hor	8.367449441
High	P50502	Hsc70-interacting protein OS=Homo sapiens OX=9606 GN	8.345646824
High	Q9NS69	Mitochondrial import receptor subunit TOM22 homolog (	8.340205457
High	Q5JTV8	Torsin-1A-interacting protein 1 OS=Homo sapiens OX=960	8.272865381
High	Q2VPK5	Cytoplasmic tRNA 2-thiolation protein 2 OS=Homo sapien	8.266717136
High	Q7L8L6	FAST kinase domain-containing protein 5, mitochondrial (	8.21756364
High	P50395	Rab GDP dissociation inhibitor beta OS=Homo sapiens OX	8.210200363
High	P62847	40S ribosomal protein S24 OS=Homo sapiens OX=9606 GI	8.177807002
High	Q13112	Chromatin assembly factor 1 subunit B OS=Homo sapiens	8.165302148
High	P36542	ATP synthase subunit gamma, mitochondrial OS=Homo sa	8.16478626
High	Q9Y3A5	Ribosome maturation protein SBDS OS=Homo sapiens OX	8.156984803
High	P45954	Short/branched chain specific acyl-CoA dehydrogenase, m	8.14892544
High	Q86WB0	Nuclear-interacting partner of ALK OS=Homo sapiens OX=	8.14018286
High	Q99798	Aconitate hydratase, mitochondrial OS=Homo sapiens OX	8.13611637
High	Q9Y5V3	Melanoma-associated antigen D1 OS=Homo sapiens OX=9	8.113718509
High	Q9UKX7	Nuclear pore complex protein Nup50 OS=Homo sapiens C	8.112386975
High	Q96L92	Sorting nexin-27 OS=Homo sapiens OX=9606 GN=SNX27 F	8.104606187
High	P62899	60S ribosomal protein L31 OS=Homo sapiens OX=9606 GI	8.095935361
High	P53350	Serine/threonine-protein kinase PLK1 OS=Homo sapiens (	8.064629442
High	B5ME19	Eukaryotic translation initiation factor 3 subunit C-like pro	8.033838736
High	P63010	AP-2 complex subunit beta OS=Homo sapiens OX=9606 G	8.001570379
High	P31153	S-adenosylmethionine synthase isoform type-2 OS=Homo	7.956926831
High	Q9Y613	FH1/FH2 domain-containing protein 1 OS=Homo sapiens (	7.944459068
High	Q9UHD2	Serine/threonine-protein kinase TBK1 OS=Homo sapiens (	7.921353787
High	Q12800	Alpha-globin transcription factor CP2 OS=Homo sapiens C	7.914224368
High	Q8WXF1	Paraspeckle component 1 OS=Homo sapiens OX=9606 GN	7.86585152
High	P23381	Tryptophan--tRNA ligase, cytoplasmic OS=Homo sapiens (	7.85612122
High	Q5VUA4	Zinc finger protein 318 OS=Homo sapiens OX=9606 GN=ZI	7.834862079
High	Q96HC4	PDZ and LIM domain protein 5 OS=Homo sapiens OX=960	7.827504184

High	Q15907	Ras-related protein Rab-11B OS=Homo sapiens OX=9606	7.811636789
High	Q15555	Microtubule-associated protein RP/EB family member 2 C	7.749152258
High	Q9UHD1	Cysteine and histidine-rich domain-containing protein 1 O	7.740529404
High	A6NDG6	Glycerol-3-phosphate phosphatase OS=Homo sapiens OX=	7.731672111
High	Q15003	Condensin complex subunit 2 OS=Homo sapiens OX=9606	7.609002454
High	Q9BT78	COP9 signalosome complex subunit 4 OS=Homo sapiens C	7.606089002
High	Q10567	AP-1 complex subunit beta-1 OS=Homo sapiens OX=9606	7.604121626
High	Q9UM00	Calcium load-activated calcium channel OS=Homo sapiens	7.58772766
High	P26038	Moesin OS=Homo sapiens OX=9606 GN=MSN PE=1 SV=3	7.566788946
High	Q92552	28S ribosomal protein S27, mitochondrial OS=Homo sapie	7.562306949
High	Q7L014	Probable ATP-dependent RNA helicase DDX46 OS=Homo	7.555848105
High	Q9UBX3	Mitochondrial dicarboxylate carrier OS=Homo sapiens OX	7.553542942
High	O00487	26S proteasome non-ATPase regulatory subunit 14 OS=H	7.552494672
High	Q9BUK6	Protein misato homolog 1 OS=Homo sapiens OX=9606 GN	7.516473748
High	Q9H583	HEAT repeat-containing protein 1 OS=Homo sapiens OX=9	7.494312091
High	Q04206	Transcription factor p65 OS=Homo sapiens OX=9606 GN=	7.428492877
High	Q9H078	Caseinolytic peptidase B protein homolog OS=Homo sapie	7.415887561
High	P27695	DNA-(apurinic or apyrimidinic site) lyase OS=Homo sapier	7.387328608
High	Q8WU90	Zinc finger CCCH domain-containing protein 15 OS=Homo	7.362540661
High	Q2NKX8	DNA excision repair protein ERCC-6-like OS=Homo sapiens	7.352463182
High	P14678	Small nuclear ribonucleoprotein-associated proteins B an	7.331119055
High	P55196	Afadin OS=Homo sapiens OX=9606 GN=AFDN PE=1 SV=3	7.308192137
High	P24666	Low molecular weight phosphotyrosine protein phosphat	7.30461535
High	Q96HS1	Serine/threonine-protein phosphatase PGAM5, mitochon	7.296441727
High	A6NMY6	Putative annexin A2-like protein OS=Homo sapiens OX=96	7.295568055
High	P48556	26S proteasome non-ATPase regulatory subunit 8 OS=Ho	7.283278326
High	Q9H7Z7	Prostaglandin E synthase 2 OS=Homo sapiens OX=9606 G	7.270694655
High	Q9UBB6	Neurochondrin OS=Homo sapiens OX=9606 GN=NCDN PE	7.204618756
High	Q9BTE3	Mini-chromosome maintenance complex-binding protein	7.182526972
High	P08134	Rho-related GTP-binding protein RhoC OS=Homo sapiens	7.155022606
High	Q96A65	Exocyst complex component 4 OS=Homo sapiens OX=960	7.154000804
High	P07910	Heterogeneous nuclear ribonucleoproteins C1/C2 OS=Ho	7.152355349
High	P49821	NADH dehydrogenase [ubiquinone] flavoprotein 1, mitoc	7.114964612
High	Q5VYK3	Proteasome adapter and scaffold protein ECM29 OS=Hom	7.099630577
High	Q96HY7	Probable 2-oxoglutarate dehydrogenase E1 component D	7.064176449
High	Q01650	Large neutral amino acids transporter small subunit 1 OS=	7.037725395
High	Q9NYL9	Tropomodulin-3 OS=Homo sapiens OX=9606 GN=TMOD3	6.981136799
High	P62873	Guanine nucleotide-binding protein G(I)/G(S)/G(T) subuni	6.97682236
High	Q9ULX3	RNA-binding protein NOB1 OS=Homo sapiens OX=9606 G	6.958507833
High	P52907	F-actin-capping protein subunit alpha-1 OS=Homo sapiens	6.957677066
High	O14545	TRAF-type zinc finger domain-containing protein 1 OS=Ho	6.907592736
High	Q9BW27	Nuclear pore complex protein Nup85 OS=Homo sapiens C	6.902766298
High	Q14168	MAGUK p55 subfamily member 2 OS=Homo sapiens OX=9	6.881919847
High	P13995	Bifunctional methylenetetrahydrofolate dehydrogenase/c	6.881559012
High	Q32P28	Prolyl 3-hydroxylase 1 OS=Homo sapiens OX=9606 GN=P3	6.863657148
High	O95470	Sphingosine-1-phosphate lyase 1 OS=Homo sapiens OX=9	6.861288089
High	O14773	Tripeptidyl-peptidase 1 OS=Homo sapiens OX=9606 GN=T	6.85304926
High	Q9NZW5	MAGUK p55 subfamily member 6 OS=Homo sapiens OX=9	6.852226301
High	P08237	ATP-dependent 6-phosphofructokinase, muscle type OS=I	6.849022268
High	P55209	Nucleosome assembly protein 1-like 1 OS=Homo sapiens	6.841892731

High	Q9UN86	Ras GTPase-activating protein-binding protein 2 OS=Homo	6.828388789
High	P34949	Mannose-6-phosphate isomerase OS=Homo sapiens OX=9	6.792634963
High	Q9GZT9	Egl nine homolog 1 OS=Homo sapiens OX=9606 GN=EGLN	6.790753151
High	Q9UJV9	Probable ATP-dependent RNA helicase DDX41 OS=Homo s	6.785168016
High	Q04726	Transducin-like enhancer protein 3 OS=Homo sapiens OX=	6.782124367
High	Q9Y3D9	28S ribosomal protein S23, mitochondrial OS=Homo sapie	6.767288687
High	P22570	NADPH:adrenodoxin oxidoreductase, mitochondrial OS=H	6.765619505
High	Q9NPI6	mRNA-decapping enzyme 1A OS=Homo sapiens OX=9606	6.762456262
High	Q9NUQ9	Protein FAM49B OS=Homo sapiens OX=9606 GN=FAM49I	6.749824052
High	Q9P0J1	[Pyruvate dehydrogenase [acetyl-transferring]]-phosphata	6.746419686
High	Q8IWS0	PHD finger protein 6 OS=Homo sapiens OX=9606 GN=PHF	6.727714943
High	Q15149	Plectin OS=Homo sapiens OX=9606 GN=PLEC PE=1 SV=3	6.69248674
High	Q9UGI8	Testin OS=Homo sapiens OX=9606 GN=TES PE=1 SV=1	6.688677597
High	P50914	60S ribosomal protein L14 OS=Homo sapiens OX=9606 GN	6.684595823
High	Q9HCN4	GPN-loop GTPase 1 OS=Homo sapiens OX=9606 GN=GPN1	6.57042354
High	Q96JB5	CDK5 regulatory subunit-associated protein 3 OS=Homo s	6.560207671
High	O43156	TELO2-interacting protein 1 homolog OS=Homo sapiens O	6.553462833
High	P46379	Large proline-rich protein BAG6 OS=Homo sapiens OX=96	6.55261238
High	P62829	60S ribosomal protein L23 OS=Homo sapiens OX=9606 GN	6.548520595
High	Q9UEW8	STE20/SPS1-related proline-alanine-rich protein kinase OS	6.547069028
High	Q9NVI1	Fanconi anemia group I protein OS=Homo sapiens OX=960	6.532403732
High	Q5H9R7	Serine/threonine-protein phosphatase 6 regulatory subur	6.529227405
High	Q9HB07	UPF0160 protein MYG1, mitochondrial OS=Homo sapiens	6.52667101
High	O43823	A-kinase anchor protein 8 OS=Homo sapiens OX=9606 GN	6.492763912
High	O60547	GDP-mannose 4,6 dehydratase OS=Homo sapiens OX=960	6.449234311
High	Q9BQ39	ATP-dependent RNA helicase DDX50 OS=Homo sapiens O	6.41930306
High	P55884	Eukaryotic translation initiation factor 3 subunit B OS=Hor	6.411739949
High	Q9BSD7	Cancer-related nucleoside-triphosphatase OS=Homo sapie	6.410095014
High	Q9UPN7	Serine/threonine-protein phosphatase 6 regulatory subur	6.399276542
High	P61225	Ras-related protein Rap-2b OS=Homo sapiens OX=9606 G	6.394118576
High	P04908	Histone H2A type 1-B/E OS=Homo sapiens OX=9606 GN=H	6.393843212
High	Q9Y2Z0	Protein SGT1 homolog OS=Homo sapiens OX=9606 GN=SU	6.357176717
High	P27816	Microtubule-associated protein 4 OS=Homo sapiens OX=9	6.348280452
High	Q8TB72	Pumilio homolog 2 OS=Homo sapiens OX=9606 GN=PUM2	6.339415975
High	Q9Y5K5	Ubiquitin carboxyl-terminal hydrolase isozyme L5 OS=Hor	6.331900726
High	Q9Y2X9	Zinc finger protein 281 OS=Homo sapiens OX=9606 GN=ZI	6.325381324
High	Q15833	Syntaxin-binding protein 2 OS=Homo sapiens OX=9606 GN	6.314079208
High	O95299	NADH dehydrogenase [ubiquinone] 1 alpha subcomplex s	6.281249265
High	Q9UJX3	Anaphase-promoting complex subunit 7 OS=Homo sapien	6.276919182
High	Q15459	Splicing factor 3A subunit 1 OS=Homo sapiens OX=9606 G	6.270112687
High	Q92888	Rho guanine nucleotide exchange factor 1 OS=Homo sapi	6.264184007
High	Q9Y2X3	Nucleolar protein 58 OS=Homo sapiens OX=9606 GN=NOI	6.262496781
High	Q04917	14-3-3 protein eta OS=Homo sapiens OX=9606 GN=YWHA	6.25436153
High	P26358	DNA (cytosine-5)-methyltransferase 1 OS=Homo sapiens O	6.247594885
High	Q9Y4K3	TNF receptor-associated factor 6 OS=Homo sapiens OX=9	6.220789568
High	Q99729	Heterogeneous nuclear ribonucleoprotein A/B OS=Homo	6.194729807
High	P62753	40S ribosomal protein S6 OS=Homo sapiens OX=9606 GN=	6.187531039
High	Q9BZE1	39S ribosomal protein L37, mitochondrial OS=Homo sapie	6.181378274
High	P60866	40S ribosomal protein S20 OS=Homo sapiens OX=9606 GN	6.165169358
High	Q96EK5	KIF1-binding protein OS=Homo sapiens OX=9606 GN=KIF1	6.162032982

High	Q07955	Serine/arginine-rich splicing factor 1 OS=Homo sapiens OX	6.146900481
High	Q9Y490	Talin-1 OS=Homo sapiens OX=9606 GN=TLN1 PE=1 SV=3	6.139808181
High	Q9H9A6	Leucine-rich repeat-containing protein 40 OS=Homo sapie	6.129249781
High	P01023	Alpha-2-macroglobulin OS=Homo sapiens OX=9606 GN=A	6.104254986
High	P49790	Nuclear pore complex protein Nup153 OS=Homo sapiens	6.092749917
High	P62906	60S ribosomal protein L10a OS=Homo sapiens OX=9606 G	6.086892178
High	Q12972	Nuclear inhibitor of protein phosphatase 1 OS=Homo sapi	6.076186279
High	Q9NRK6	ATP-binding cassette sub-family B member 10, mitochonc	6.067729224
High	P41214	Eukaryotic translation initiation factor 2D OS=Homo sapie	6.066330529
High	Q53GS9	U4/U6.U5 tri-snRNP-associated protein 2 OS=Homo sapie	6.048028713
High	Q92889	DNA repair endonuclease XPF OS=Homo sapiens OX=9606	6.046661764
High	P62913	60S ribosomal protein L11 OS=Homo sapiens OX=9606 GN	6.029474235
High	P48147	Prolyl endopeptidase OS=Homo sapiens OX=9606 GN=PR	6.016779785
High	Q9UNH7	Sorting nexin-6 OS=Homo sapiens OX=9606 GN=SNX6 PE=	6.003533511
High	O43396	Thioredoxin-like protein 1 OS=Homo sapiens OX=9606 GN	5.987695049
High	Q9UID3	Vacuolar protein sorting-associated protein 51 homolog C	5.954677021
High	P10586	Receptor-type tyrosine-protein phosphatase F OS=Homo	5.919360347
High	P42285	Exosome RNA helicase MTR4 OS=Homo sapiens OX=9606	5.918985923
High	P52747	Zinc finger protein 143 OS=Homo sapiens OX=9606 GN=Z	5.901355635
High	O75351	Vacuolar protein sorting-associated protein 4B OS=Homo	5.894691252
High	Q14683	Structural maintenance of chromosomes protein 1A OS=H	5.890600325
High	Q9BPU6	Dihydropyrimidinase-related protein 5 OS=Homo sapiens	5.84314931
High	P00374	Dihydrofolate reductase OS=Homo sapiens OX=9606 GN=	5.820639634
High	P25789	Proteasome subunit alpha type-4 OS=Homo sapiens OX=9	5.813743674
High	P14923	Junction plakoglobin OS=Homo sapiens OX=9606 GN=JUP	5.796380396
High	O43598	2'-deoxynucleoside 5'-phosphate N-hydrolase 1 OS=Homo	5.795880017
High	Q9P2I0	Cleavage and polyadenylation specificity factor subunit 2	5.793204768
High	Q9Y2Z4	Tyrosine--tRNA ligase, mitochondrial OS=Homo sapiens O	5.7931369
High	Q9H3P2	Negative elongation factor A OS=Homo sapiens OX=9606	5.787507271
High	Q9Y4P1	Cysteine protease ATG4B OS=Homo sapiens OX=9606 GN	5.779891912
High	A0FGR8	Extended synaptotagmin-2 OS=Homo sapiens OX=9606 G	5.772831889
High	P62937	Peptidyl-prolyl cis-trans isomerase A OS=Homo sapiens O	5.752254369
High	Q3KQV9	UDP-N-acetylhexosamine pyrophosphorylase-like protein	5.74875903
High	P46779	60S ribosomal protein L28 OS=Homo sapiens OX=9606 GN	5.740227471
High	Q9BTW9	Tubulin-specific chaperone D OS=Homo sapiens OX=9606	5.739932764
High	Q96H79	Zinc finger CCCH-type antiviral protein 1-like OS=Homo sa	5.726998728
High	Q8IYI6	Exocyst complex component 8 OS=Homo sapiens OX=960	5.716497414
High	P51665	26S proteasome non-ATPase regulatory subunit 7 OS=Ho	5.715343717
High	Q99961	Endophilin-A2 OS=Homo sapiens OX=9606 GN=SH3GL1 P	5.694171534
High	O00186	Syntaxin-binding protein 3 OS=Homo sapiens OX=9606 GN	5.647651439
High	Q12873	Chromodomain-helicase-DNA-binding protein 3 OS=Homo	5.646660905
High	P04004	Vitronectin OS=Homo sapiens OX=9606 GN=VTN PE=1 SV	5.61852391
High	Q96RS6	NudC domain-containing protein 1 OS=Homo sapiens OX=	5.610125442
High	O95793	Double-stranded RNA-binding protein Staufen homolog 1	5.606055944
High	Q9Y266	Nuclear migration protein nudC OS=Homo sapiens OX=96	5.605327341
High	Q69YN2	CWF19-like protein 1 OS=Homo sapiens OX=9606 GN=CW	5.567678628
High	Q6PJG6	BRCA1-associated ATM activator 1 OS=Homo sapiens OX=	5.530177984
High	Q15382	GTP-binding protein Rheb OS=Homo sapiens OX=9606 GN	5.522734005
High	Q8TEQ6	Gem-associated protein 5 OS=Homo sapiens OX=9606 GN	5.517076133
High	Q16543	Hsp90 co-chaperone Cdc37 OS=Homo sapiens OX=9606 G	5.513980631

High	P48047	ATP synthase subunit O, mitochondrial OS=Homo sapiens	5.487460588
High	P35611	Alpha-adducin OS=Homo sapiens OX=9606 GN=ADD1 PE=	5.480842938
High	Q01518	Adenylyl cyclase-associated protein 1 OS=Homo sapiens C	5.475474063
High	Q9H2M9	Rab3 GTPase-activating protein non-catalytic subunit OS=	5.44547886
High	O14979	Heterogeneous nuclear ribonucleoprotein D-like OS=Hom	5.444375528
High	Q9UBQ0	Vacuolar protein sorting-associated protein 29 OS=Homo	5.443333207
High	Q6PKG0	La-related protein 1 OS=Homo sapiens OX=9606 GN=LARI	5.44333041
High	O60231	Pre-mRNA-splicing factor ATP-dependent RNA helicase DH	5.442036247
High	Q9BQG0	Myb-binding protein 1A OS=Homo sapiens OX=9606 GN=I	5.429573822
High	P09211	Glutathione S-transferase P OS=Homo sapiens OX=9606 G	5.423496206
High	Q05048	Cleavage stimulation factor subunit 1 OS=Homo sapiens C	5.370794343
High	P30520	Adenylosuccinate synthetase isozyme 2 OS=Homo sapien	5.361443657
High	Q16836	Hydroxyacyl-coenzyme A dehydrogenase, mitochondrial (	5.356631624
High	Q14181	DNA polymerase alpha subunit B OS=Homo sapiens OX=9	5.336864685
High	P60174	Triosephosphate isomerase OS=Homo sapiens OX=9606 G	5.334533986
High	Q9Y4X5	E3 ubiquitin-protein ligase ARIH1 OS=Homo sapiens OX=9	5.330845894
High	Q02252	Methylmalonate-semialdehyde dehydrogenase [acylating	5.310189949
High	Q9BSJ2	Gamma-tubulin complex component 2 OS=Homo sapiens	5.290628263
High	O14880	Microsomal glutathione S-transferase 3 OS=Homo sapiens	5.264880366
High	Q6PHR2	Serine/threonine-protein kinase ULK3 OS=Homo sapiens (	5.264081883
High	P13667	Protein disulfide-isomerase A4 OS=Homo sapiens OX=960	5.251429365
High	Q96HE7	ERO1-like protein alpha OS=Homo sapiens OX=9606 GN=E	5.231952704
High	Q6IN85	Serine/threonine-protein phosphatase 4 regulatory subur	5.204969111
High	P25490	Transcriptional repressor protein YY1 OS=Homo sapiens C	5.200766314
High	P41240	Tyrosine-protein kinase CSK OS=Homo sapiens OX=9606 (	5.198904806
High	P39019	40S ribosomal protein S19 OS=Homo sapiens OX=9606 GI	5.197995691
High	O75396	Vesicle-trafficking protein SEC22b OS=Homo sapiens OX=9	5.192667961
High	Q15738	Sterol-4-alpha-carboxylate 3-dehydrogenase, decarboxyla	5.183322304
High	Q9ULT8	E3 ubiquitin-protein ligase HECTD1 OS=Homo sapiens OX=	5.172574505
High	O60762	Dolichol-phosphate mannosyltransferase subunit 1 OS=Hc	5.168770306
High	Q96CW5	Gamma-tubulin complex component 3 OS=Homo sapiens	5.168383265
High	P43490	Nicotinamide phosphoribosyltransferase OS=Homo sapier	5.163347048
High	P28074	Proteasome subunit beta type-5 OS=Homo sapiens OX=96	5.147947191
High	P52434	DNA-directed RNA polymerases I, II, and III subunit RPABC	5.110754339
High	Q92990	Glomulin OS=Homo sapiens OX=9606 GN=GLMN PE=1 SV	5.069879821
High	Q9Y2J2	Band 4.1-like protein 3 OS=Homo sapiens OX=9606 GN=E	5.059184791
High	Q15785	Mitochondrial import receptor subunit TOM34 OS=Homo	5.024247575
High	O60502	Protein O-GlcNAcase OS=Homo sapiens OX=9606 GN=OG	5.024052105
High	P51571	Translocon-associated protein subunit delta OS=Homo sa	5.016013078
High	Q9H4I3	TraB domain-containing protein OS=Homo sapiens OX=96	4.983402427
High	Q9NRG7	Epimerase family protein SDR39U1 OS=Homo sapiens OX=	4.957424488
High	P50897	Palmitoyl-protein thioesterase 1 OS=Homo sapiens OX=96	4.956637722
High	Q9UBT2	SUMO-activating enzyme subunit 2 OS=Homo sapiens OX	4.937646315
High	P11766	Alcohol dehydrogenase class-3 OS=Homo sapiens OX=960	4.93674172
High	Q9Y263	Phospholipase A-2-activating protein OS=Homo sapiens O	4.936666641
High	P42345	Serine/threonine-protein kinase mTOR OS=Homo sapiens	4.926933709
High	Q13228	Methanethiol oxidase OS=Homo sapiens OX=9606 GN=SE	4.919012953
High	Q12996	Cleavage stimulation factor subunit 3 OS=Homo sapiens C	4.913996294
High	Q13416	Origin recognition complex subunit 2 OS=Homo sapiens C	4.890759031
High	Q5VUJ6	Leucine-rich repeat and calponin homology domain-conta	4.884416445

High	P10253	Lysosomal alpha-glucosidase OS=Homo sapiens OX=9606	4.851089007
High	O75844	CAAX prenyl protease 1 homolog OS=Homo sapiens OX=9	4.848936747
High	P43897	Elongation factor Ts, mitochondrial OS=Homo sapiens OX	4.84863015
High	Q9Y223	Bifunctional UDP-N-acetylglucosamine 2-epimerase/N-ac	4.848114023
High	Q9NZJ7	Mitochondrial carrier homolog 1 OS=Homo sapiens OX=9	4.844196543
High	Q15126	Phosphomevalonate kinase OS=Homo sapiens OX=9606 G	4.831502516
High	P40938	Replication factor C subunit 3 OS=Homo sapiens OX=9606	4.830033235
High	Q7Z4Q2	HEAT repeat-containing protein 3 OS=Homo sapiens OX=9	4.82303264
High	Q8NCA5	Protein FAM98A OS=Homo sapiens OX=9606 GN=FAM98A	4.819014419
High	P61353	60S ribosomal protein L27 OS=Homo sapiens OX=9606 GN	4.8015965
High	Q96S44	EKC/KEOPS complex subunit TP53RK OS=Homo sapiens O	4.78994915
High	O14976	Cyclin-G-associated kinase OS=Homo sapiens OX=9606 GN	4.770830297
High	P06753	Tropomyosin alpha-3 chain OS=Homo sapiens OX=9606 G	4.747195836
High	P58004	Sestrin-2 OS=Homo sapiens OX=9606 GN=SESN2 PE=1 SV=	4.744004273
High	Q9UBS4	DnaJ homolog subfamily B member 11 OS=Homo sapiens	4.738975166
High	Q9Y6D9	Mitotic spindle assembly checkpoint protein MAD1 OS=H	4.738023809
High	P51648	Fatty aldehyde dehydrogenase OS=Homo sapiens OX=960	4.735182177
High	Q9BY32	Inosine triphosphate pyrophosphatase OS=Homo sapiens	4.727428371
High	Q9UNL2	Translocon-associated protein subunit gamma OS=Homo	4.670195478
High	P16118	6-phosphofructo-2-kinase/fructose-2,6-bisphosphatase 1	4.622151948
High	Q6DD88	Atlastin-3 OS=Homo sapiens OX=9606 GN=ATL3 PE=1 SV=	4.617652731
High	Q8WWY3	U4/U6 small nuclear ribonucleoprotein Prp31 OS=Homo s	4.605722072
High	Q9Y4P3	Transducin beta-like protein 2 OS=Homo sapiens OX=960	4.601712695
High	P62280	40S ribosomal protein S11 OS=Homo sapiens OX=9606 GN	4.593951276
High	Q9NVM4	Protein arginine N-methyltransferase 7 OS=Homo sapiens	4.571757391
High	Q16186	Proteasomal ubiquitin receptor ADRM1 OS=Homo sapien	4.53521248
High	P11802	Cyclin-dependent kinase 4 OS=Homo sapiens OX=9606 GN	4.527327786
High	P12694	2-oxoisovalerate dehydrogenase subunit alpha, mitochon	4.527243551
High	P49589	Cysteine--tRNA ligase, cytoplasmic OS=Homo sapiens OX=	4.522296747
High	P49841	Glycogen synthase kinase-3 beta OS=Homo sapiens OX=9	4.511449283
High	Q9NX58	Cell growth-regulating nucleolar protein OS=Homo sapien	4.506402551
High	Q15208	Serine/threonine-protein kinase 38 OS=Homo sapiens OX	4.500312917
High	Q15797	Mothers against decapentaplegic homolog 1 OS=Homo sa	4.490797478
High	Q14997	Proteasome activator complex subunit 4 OS=Homo sapien	4.488347665
High	Q9HC07	Transmembrane protein 165 OS=Homo sapiens OX=9606	4.472112434
High	Q9Y2W6	Tudor and KH domain-containing protein OS=Homo sapie	4.469544156
High	Q9NWV8	BRISC and BRCA1-A complex member 1 OS=Homo sapiens	4.460171442
High	P22087	rRNA 2'-O-methyltransferase fibrillarin OS=Homo sapiens	4.452225295
High	P43304	Glycerol-3-phosphate dehydrogenase, mitochondrial OS=	4.439481128
High	Q9Y512	Sorting and assembly machinery component 50 homolog	4.434932724
High	Q8IWF6	Protein DENND6A OS=Homo sapiens OX=9606 GN=DENN	4.430041182
High	P01859	Immunoglobulin heavy constant gamma 2 OS=Homo sapi	4.422197067
High	Q86UK7	E3 ubiquitin-protein ligase ZNF598 OS=Homo sapiens OX=	4.421246216
High	Q9BZE4	Nucleolar GTP-binding protein 1 OS=Homo sapiens OX=96	4.416008201
High	P08574	Cytochrome c1, heme protein, mitochondrial OS=Homo s	4.406824737
High	P14324	Farnesyl pyrophosphate synthase OS=Homo sapiens OX=9	4.406742207
High	Q8NHH9	Atlastin-2 OS=Homo sapiens OX=9606 GN=ATL2 PE=1 SV=	4.3982539
High	Q15691	Microtubule-associated protein RP/EB family member 1 O	4.385282104
High	O95163	Elongator complex protein 1 OS=Homo sapiens OX=9606	4.382365989
High	P50750	Cyclin-dependent kinase 9 OS=Homo sapiens OX=9606 GN	4.380995208

High	Q9GZT4	Serine racemase OS=Homo sapiens OX=9606 GN=SRR PE=	4.37788964
High	Q08257	Quinone oxidoreductase OS=Homo sapiens OX=9606 GN=	4.3400838
High	Q13418	Integrin-linked protein kinase OS=Homo sapiens OX=9606	4.337594246
High	O15260	Surfeit locus protein 4 OS=Homo sapiens OX=9606 GN=SU	4.328086988
High	P13473	Lysosome-associated membrane glycoprotein 2 OS=Hom	4.321481621
High	Q8N6T3	ADP-ribosylation factor GTPase-activating protein 1 OS=H	4.314885531
High	P08758	Annexin A5 OS=Homo sapiens OX=9606 GN=ANXA5 PE=1	4.313790013
High	P62070	Ras-related protein R-Ras2 OS=Homo sapiens OX=9606 G	4.306976932
High	Q8N680	Zinc finger and BTB domain-containing protein 2 OS=Hom	4.304956341
High	Q14669	E3 ubiquitin-protein ligase TRIP12 OS=Homo sapiens OX=	4.275069086
High	P02771	Alpha-fetoprotein OS=Homo sapiens OX=9606 GN=AFP PE	4.266241164
High	Q96EP5	DAZ-associated protein 1 OS=Homo sapiens OX=9606 GN=	4.240785569
High	Q9BQ95	Evolutionarily conserved signaling intermediate in Toll pat	4.236497135
High	P51003	Poly(A) polymerase alpha OS=Homo sapiens OX=9606 GN	4.211266141
High	Q8WYA6	Beta-catenin-like protein 1 OS=Homo sapiens OX=9606 G	4.211226585
High	Q96TA2	ATP-dependent zinc metalloprotease YME1L1 OS=Homo s	4.206558867
High	Q9BYD3	39S ribosomal protein L4, mitochondrial OS=Homo sapier	4.190238335
High	Q68EM7	Rho GTPase-activating protein 17 OS=Homo sapiens OX=9	4.160333443
High	Q13724	Mannosyl-oligosaccharide glucosidase OS=Homo sapiens	4.151687696
High	P06396	Gelsolin OS=Homo sapiens OX=9606 GN=GSN PE=1 SV=1	4.146971385
High	Q14746	Conserved oligomeric Golgi complex subunit 2 OS=Homo	4.146362618
High	P47914	60S ribosomal protein L29 OS=Homo sapiens OX=9606 GN	4.113137971
High	Q9NY33	Dipeptidyl peptidase 3 OS=Homo sapiens OX=9606 GN=D	4.092749917
High	Q96S55	ATPase WRNIP1 OS=Homo sapiens OX=9606 GN=WRNIP1	4.07473949
High	Q13435	Splicing factor 3B subunit 2 OS=Homo sapiens OX=9606 G	4.061130178
High	P51151	Ras-related protein Rab-9A OS=Homo sapiens OX=9606 G	4.050317309
High	O75794	Cell division cycle protein 123 homolog OS=Homo sapiens	4.048714804
High	Q9NRZ9	Lymphoid-specific helicase OS=Homo sapiens OX=9606 GI	4.021084681
High	Q6P1Q9	Methyltransferase-like protein 2B OS=Homo sapiens OX=9	3.981715692
High	Q96EE3	Nucleoporin SEH1 OS=Homo sapiens OX=9606 GN=SEH1L	3.952452372
High	Q6YHU6	Thyroid adenoma-associated protein OS=Homo sapiens O	3.950394387
High	P27361	Mitogen-activated protein kinase 3 OS=Homo sapiens OX=	3.940417992
High	P35268	60S ribosomal protein L22 OS=Homo sapiens OX=9606 GN	3.934793872
High	P00492	Hypoxanthine-guanine phosphoribosyltransferase OS=Ho	3.926751015
High	Q9Y316	Protein MEMO1 OS=Homo sapiens OX=9606 GN=MEMO1	3.925549281
High	P28072	Proteasome subunit beta type-6 OS=Homo sapiens OX=96	3.917573699
High	Q8NG31	Kinetochore scaffold 1 OS=Homo sapiens OX=9606 GN=KI	3.914352712
High	Q9BUI4	DNA-directed RNA polymerase III subunit RPC3 OS=Homo	3.912218582
High	Q99661	Kinesin-like protein KIF2C OS=Homo sapiens OX=9606 GN	3.912218582
High	O43929	Origin recognition complex subunit 4 OS=Homo sapiens C	3.91150953
High	Q9UBB9	Tuftelin-interacting protein 11 OS=Homo sapiens OX=960	3.909741947
High	Q7Z5L9	Interferon regulatory factor 2-binding protein 2 OS=Homo	3.906228219
High	Q13131	5'-AMP-activated protein kinase catalytic subunit alpha-1	3.88008459
High	Q16740	ATP-dependent Clp protease proteolytic subunit, mitocho	3.878768545
High	P46736	Lys-63-specific deubiquitinase BRCC36 OS=Homo sapiens	3.875133623
High	Q9GZZ1	N-alpha-acetyltransferase 50 OS=Homo sapiens OX=9606	3.87484417
High	P68431	Histone H3.1 OS=Homo sapiens OX=9606 GN=HIST1H3A F	3.861955112
High	Q9UPT9	Ubiquitin carboxyl-terminal hydrolase 22 OS=Homo sapie	3.838033384
High	P63241	Eukaryotic translation initiation factor 5A-1 OS=Homo sap	3.829505413
High	P31146	Coronin-1A OS=Homo sapiens OX=9606 GN=CORO1A PE=	3.828566099

High	Q8TC07	TBC1 domain family member 15 OS=Homo sapiens OX=96	3.825415051
High	A4D1P6	WD repeat-containing protein 91 OS=Homo sapiens OX=9	3.814741235
High	Q13362	Serine/threonine-protein phosphatase 2A 56 kDa regulato	3.809948582
High	P53582	Methionine aminopeptidase 1 OS=Homo sapiens OX=960	3.809411444
High	P41743	Protein kinase C iota type OS=Homo sapiens OX=9606 GN	3.802443787
High	Q96AG4	Leucine-rich repeat-containing protein 59 OS=Homo sapie	3.800793521
High	Q4KMP7	TBC1 domain family member 10B OS=Homo sapiens OX=9	3.800519085
High	O94966	Ubiquitin carboxyl-terminal hydrolase 19 OS=Homo sapie	3.794254459
High	Q96T88	E3 ubiquitin-protein ligase UHRF1 OS=Homo sapiens OX=9	3.790484985
High	P07741	Adenine phosphoribosyltransferase OS=Homo sapiens OX	3.780153614
High	Q8WUX9	Charged multivesicular body protein 7 OS=Homo sapiens	3.775892869
High	Q9Y2I8	WD repeat-containing protein 37 OS=Homo sapiens OX=9	3.775466937
High	P61009	Signal peptidase complex subunit 3 OS=Homo sapiens OX	3.744486287
High	O75688	Protein phosphatase 1B OS=Homo sapiens OX=9606 GN=	3.743282254
High	P54709	Sodium/potassium-transporting ATPase subunit beta-3 OS	3.73072061
High	Q01085	Nucleolysin TIAR OS=Homo sapiens OX=9606 GN=TIAL1 P	3.730253627
High	Q8NBX0	Saccharopine dehydrogenase-like oxidoreductase OS=Hor	3.729321164
High	Q5JWF2	Guanine nucleotide-binding protein G(s) subunit alpha iso	3.723308471
High	Q16629	Serine/arginine-rich splicing factor 7 OS=Homo sapiens OX	3.721475035
High	Q8WUK0	Phosphatidylglycerophosphatase and protein-tyrosine ph	3.712422191
High	O94973	AP-2 complex subunit alpha-2 OS=Homo sapiens OX=9606	3.705313376
High	P13693	Translationally-controlled tumor protein OS=Homo sapien	3.703801818
High	O00264	Membrane-associated progesterone receptor component	3.7000571
High	Q9P0J0	NADH dehydrogenase [ubiquinone] 1 alpha subcomplex s	3.699404516
High	P46776	60S ribosomal protein L27a OS=Homo sapiens OX=9606 G	3.696156225
High	P50579	Methionine aminopeptidase 2 OS=Homo sapiens OX=960	3.684029655
High	P10398	Serine/threonine-protein kinase A-Raf OS=Homo sapiens	3.67943832
High	Q13685	Angio-associated migratory cell protein OS=Homo sapiens	3.672441847
High	Q16850	Lanosterol 14-alpha demethylase OS=Homo sapiens OX=9	3.661543506
High	O14579	Coatomer subunit epsilon OS=Homo sapiens OX=9606 GN	3.655411257
High	P17152	Transmembrane protein 11, mitochondrial OS=Homo sap	3.642827742
High	Q7Z6Z7	E3 ubiquitin-protein ligase HUWE1 OS=Homo sapiens OX=	3.633203617
High	Q16762	Thiosulfate sulfurtransferase OS=Homo sapiens OX=9606	3.631903614
High	P36915	Guanine nucleotide-binding protein-like 1 OS=Homo sapie	3.629116983
High	Q9BQ70	Transcription factor 25 OS=Homo sapiens OX=9606 GN=T	3.624702262
High	P28482	Mitogen-activated protein kinase 1 OS=Homo sapiens OX	3.61960784
High	P29966	Myristoylated alanine-rich C-kinase substrate OS=Homo s	3.606951534
High	Q7KZI7	Serine/threonine-protein kinase MARK2 OS=Homo sapien	3.604848408
High	Q96JH7	Deubiquitinating protein VCIP135 OS=Homo sapiens OX=9	3.600394266
High	Q9H0D6	5'-3' exoribonuclease 2 OS=Homo sapiens OX=9606 GN=X	3.587371479
High	Q9UBD5	Origin recognition complex subunit 3 OS=Homo sapiens O	3.582528307
High	Q15637	Splicing factor 1 OS=Homo sapiens OX=9606 GN=SF1 PE=1	3.558147824
High	O75153	Clustered mitochondria protein homolog OS=Homo sapie	3.54041544
High	Q86UV5	Ubiquitin carboxyl-terminal hydrolase 48 OS=Homo sapie	3.540004744
High	O00273	DNA fragmentation factor subunit alpha OS=Homo sapien	3.538201442
High	Q13642	Four and a half LIM domains protein 1 OS=Homo sapiens	3.537152964
High	Q6RW13	Type-1 angiotensin II receptor-associated protein OS=Hor	3.529589509
High	O00159	Unconventional myosin-Ic OS=Homo sapiens OX=9606 GN	3.505681324
High	Q9H3N1	Thioredoxin-related transmembrane protein 1 OS=Homo	3.501000636
High	O15397	Importin-8 OS=Homo sapiens OX=9606 GN=IPO8 PE=1 SV	3.500863005

High	Q86YP4	Transcriptional repressor p66-alpha OS=Homo sapiens OX	3.496481687
High	Q02241	Kinesin-like protein KIF23 OS=Homo sapiens OX=9606 GN	3.496073196
High	Q96C19	EF-hand domain-containing protein D2 OS=Homo sapiens	3.482084614
High	Q96C36	Pyrroline-5-carboxylate reductase 2 OS=Homo sapiens OX	3.465973894
High	O15091	Mitochondrial ribonuclease P catalytic subunit OS=Homo	3.465466244
High	Q9NR50	Translation initiation factor eIF-2B subunit gamma OS=Ho	3.458795309
High	Q14157	Ubiquitin-associated protein 2-like OS=Homo sapiens OX=	3.458420756
High	Q8WVB6	Chromosome transmission fidelity protein 18 homolog OS	3.438064237
High	Q969N2	GPI transamidase component PIG-T OS=Homo sapiens OX	3.431816141
High	Q00536	Cyclin-dependent kinase 16 OS=Homo sapiens OX=9606 G	3.428773934
High	Q92504	Zinc transporter SLC39A7 OS=Homo sapiens OX=9606 GN	3.427593132
High	Q6NUM9	All-trans-retinol 13,14-reductase OS=Homo sapiens OX=9	3.379863945
High	O43719	HIV Tat-specific factor 1 OS=Homo sapiens OX=9606 GN=	3.377371574
High	O00411	DNA-directed RNA polymerase, mitochondrial OS=Homo s	3.372839048
High	Q9BYB4	Guanine nucleotide-binding protein subunit beta-like prot	3.364350408
High	Q5JTH9	RRP12-like protein OS=Homo sapiens OX=9606 GN=RRP1	3.352030542
High	Q969S3	Zinc finger protein 622 OS=Homo sapiens OX=9606 GN=Z	3.351347305
High	Q5RKV6	Exosome complex component MTR3 OS=Homo sapiens O	3.324313291
High	Q6P3W7	SCY1-like protein 2 OS=Homo sapiens OX=9606 GN=SCYL	3.321754848
High	Q96PZ0	Pseudouridylate synthase 7 homolog OS=Homo sapiens C	3.317945223
High	Q6P9B9	Integrator complex subunit 5 OS=Homo sapiens OX=9606	3.314462652
High	P61081	NEDD8-conjugating enzyme Ubc12 OS=Homo sapiens OX=	3.309006968
High	Q8IYS1	Peptidase M20 domain-containing protein 2 OS=Homo sa	3.289199983
High	O15372	Eukaryotic translation initiation factor 3 subunit H OS=Ho	3.263683192
High	Q96EY1	DnaJ homolog subfamily A member 3, mitochondrial OS=	3.255550543
High	Q0VDF9	Heat shock 70 kDa protein 14 OS=Homo sapiens OX=9606	3.247875308
High	Q9UKG1	DCC-interacting protein 13-alpha OS=Homo sapiens OX=9	3.233446476
High	Q15363	Transmembrane emp24 domain-containing protein 2 OS=	3.229147988
High	O15160	DNA-directed RNA polymerases I and III subunit RPAC1 OS	3.22848601
High	P49761	Dual specificity protein kinase CLK3 OS=Homo sapiens OX	3.220547817
High	Q9NZB2	Constitutive coactivator of PPAR-gamma-like protein 1 OS	3.181048688
High	Q9NZ01	Very-long-chain enoyl-CoA reductase OS=Homo sapiens C	3.176395372
High	P68402	Platelet-activating factor acetylhydrolase IB subunit beta	3.166852888
High	Q9UGV2	Protein NDRG3 OS=Homo sapiens OX=9606 GN=NDRG3 P	3.157952711
High	Q8N8S7	Protein enabled homolog OS=Homo sapiens OX=9606 GN	3.143210711
High	Q9NYJ8	TGF-beta-activated kinase 1 and MAP3K7-binding protein	3.143150321
High	P49792	E3 SUMO-protein ligase RanBP2 OS=Homo sapiens OX=96	3.13768969
High	P29218	Inositol monophosphatase 1 OS=Homo sapiens OX=9606	3.137272472
High	P04792	Heat shock protein beta-1 OS=Homo sapiens OX=9606 GN	3.129830279
High	P85037	Forkhead box protein K1 OS=Homo sapiens OX=9606 GN=	3.126600337
High	O95861	3'(2'),5'-bisphosphate nucleotidase 1 OS=Homo sapiens O	3.118558278
High	Q96SI9	Spermatid perinuclear RNA-binding protein OS=Homo sap	3.109355464
High	Q9Y4R8	Telomere length regulation protein TEL2 homolog OS=Ho	3.107627093
High	Q6Y7W6	GRB10-interacting GYF protein 2 OS=Homo sapiens OX=9	3.107515821
High	O43148	mRNA cap guanine-N7 methyltransferase OS=Homo sapie	3.089108911
High	P21127	Cyclin-dependent kinase 11B OS=Homo sapiens OX=9606	3.085941264
High	Q6PCE3	Glucose 1,6-bisphosphate synthase OS=Homo sapiens OX	3.082736124
High	Q6IA86	Elongator complex protein 2 OS=Homo sapiens OX=9606	3.08055579
High	O75312	Zinc finger protein ZPR1 OS=Homo sapiens OX=9606 GN=	3.079720105
High	Q6UXN9	WD repeat-containing protein 82 OS=Homo sapiens OX=9	3.066512712

High	Q9HCU5	Prolactin regulatory element-binding protein OS=Homo sa	3.066259701
High	P61201	COP9 signalosome complex subunit 2 OS=Homo sapiens O	3.055475677
High	Q96F07	Cytoplasmic FMR1-interacting protein 2 OS=Homo sapien	3.051684859
High	Q3ZCQ8	Mitochondrial import inner membrane translocase subun	3.039101323
High	Q27J81	Inverted formin-2 OS=Homo sapiens OX=9606 GN=INF2 P	3.037962313
High	Q9Y6M9	NADH dehydrogenase [ubiquinone] 1 beta subcomplex su	3.037015416
High	Q9Y2H1	Serine/threonine-protein kinase 38-like OS=Homo sapiens	3.035598929
High	P23443	Ribosomal protein S6 kinase beta-1 OS=Homo sapiens OX	3.027334408
High	Q9Y639	Neuroplastin OS=Homo sapiens OX=9606 GN=NPTN PE=1	3.023100049
High	P51553	Isocitrate dehydrogenase [NAD] subunit gamma, mitocho	3.02017857
High	Q14847	LIM and SH3 domain protein 1 OS=Homo sapiens OX=960	3.019723859
High	Q9UJU6	Drebrin-like protein OS=Homo sapiens OX=9606 GN=DBN	3.014528617
High	Q96GM8	Target of EGR1 protein 1 OS=Homo sapiens OX=9606 GN=	2.995248844
High	P30048	Thioredoxin-dependent peroxide reductase, mitochondria	2.990124366
High	Q5VV42	Threonylcarbamoyladenosine tRNA methylthiotransferase	2.982132281
High	P62266	40S ribosomal protein S23 OS=Homo sapiens OX=9606 GN	2.98088371
High	P42766	60S ribosomal protein L35 OS=Homo sapiens OX=9606 GN	2.970616222
High	O76021	Ribosomal L1 domain-containing protein 1 OS=Homo sapi	2.970055046
High	Q8N122	Regulatory-associated protein of mTOR OS=Homo sapiens	2.963371105
High	Q13573	SNW domain-containing protein 1 OS=Homo sapiens OX=	2.957424488
High	O00629	Importin subunit alpha-3 OS=Homo sapiens OX=9606 GN=	2.955134092
High	P00918	Carbonic anhydrase 2 OS=Homo sapiens OX=9606 GN=CA	2.938924676
High	Q00796	Sorbitol dehydrogenase OS=Homo sapiens OX=9606 GN=S	2.936762069
High	Q86X55	Histone-arginine methyltransferase CARM1 OS=Homo sap	2.924453039
High	P00846	ATP synthase subunit a OS=Homo sapiens OX=9606 GN=M	2.923359556
High	O60331	Phosphatidylinositol 4-phosphate 5-kinase type-1 gamma	2.916855857
High	Q9BZX2	Uridine-cytidine kinase 2 OS=Homo sapiens OX=9606 GN=	2.915066425
High	Q9BVL4	Selenoprotein O OS=Homo sapiens OX=9606 GN=SELENO	2.902395671
High	Q96JJ7	Protein disulfide-isomerase TMX3 OS=Homo sapiens OX=9	2.897223385
High	Q9NQH7	Xaa-Pro aminopeptidase 3 OS=Homo sapiens OX=9606 GN	2.883359386
High	Q8N6R0	Methyltransferase-like protein 13 OS=Homo sapiens OX=9	2.881404635
High	Q9Y217	Myotubularin-related protein 6 OS=Homo sapiens OX=960	2.876801925
High	Q13155	Aminoacyl tRNA synthase complex-interacting multifuncti	2.863279433
High	Q9UPT5	Exocyst complex component 7 OS=Homo sapiens OX=960	2.856048884
High	Q14139	Ubiquitin conjugation factor E4 A OS=Homo sapiens OX=9	2.854182286
High	P43487	Ran-specific GTPase-activating protein OS=Homo sapiens	2.851705903
High	Q9Y679	Ancient ubiquitous protein 1 OS=Homo sapiens OX=9606	2.845576027
High	Q13057	Bifunctional coenzyme A synthase OS=Homo sapiens OX=	2.844360366
High	O95197	Reticulon-3 OS=Homo sapiens OX=9606 GN=RTN3 PE=1 S	2.841336019
High	P28838	Cytosol aminopeptidase OS=Homo sapiens OX=9606 GN=	2.835052627
High	Q9UJA5	tRNA (adenine(58)-N(1))-methyltransferase non-catalytic	2.826522357
High	P62854	40S ribosomal protein S26 OS=Homo sapiens OX=9606 GN	2.824198367
High	Q9UHJ6	Sedoheptulokinase OS=Homo sapiens OX=9606 GN=SHPK	2.811634074
High	Q9BUT1	3-hydroxybutyrate dehydrogenase type 2 OS=Homo sapie	2.808548986
High	Q15185	Prostaglandin E synthase 3 OS=Homo sapiens OX=9606 G	2.808269607
High	Q9H269	Vacuolar protein sorting-associated protein 16 homolog O	2.805208242
High	P29144	Tripeptidyl-peptidase 2 OS=Homo sapiens OX=9606 GN=T	2.798876103
High	Q5T447	E3 ubiquitin-protein ligase HECTD3 OS=Homo sapiens OX=	2.794795636
High	Q9HAU0	Pleckstrin homology domain-containing family A member	2.792365633
High	O00203	AP-3 complex subunit beta-1 OS=Homo sapiens OX=9606	2.791021483

High	Q15102	Platelet-activating factor acetylhydrolase IB subunit gamn	2.785156152
High	O96005	Cleft lip and palate transmembrane protein 1 OS=Homo s	2.780153614
High	P28070	Proteasome subunit beta type-4 OS=Homo sapiens OX=96	2.774174009
High	Q9H0W8	Protein SMG9 OS=Homo sapiens OX=9606 GN=SMG9 PE=	2.772884917
High	P12270	Nucleoprotein TPR OS=Homo sapiens OX=9606 GN=TPR P	2.762959209
High	P08708	40S ribosomal protein S17 OS=Homo sapiens OX=9606 GN	2.758951849
High	Q9BV20	Methylthioribose-1-phosphate isomerase OS=Homo sapie	2.758702613
High	Q8WWC4	m-AAA protease-interacting protein 1, mitochondrial OS=	2.757955761
High	Q9GZS3	WD repeat-containing protein 61 OS=Homo sapiens OX=9	2.7490923
High	Q9HCG8	Pre-mRNA-splicing factor CWC22 homolog OS=Homo sapi	2.741841807
High	Q13425	Beta-2-syntrophin OS=Homo sapiens OX=9606 GN=SNTB2	2.736127323
High	Q8NFQ8	Torsin-1A-interacting protein 2 OS=Homo sapiens OX=960	2.715569266
High	P46063	ATP-dependent DNA helicase Q1 OS=Homo sapiens OX=9	2.711527199
High	O96019	Actin-like protein 6A OS=Homo sapiens OX=9606 GN=ACT	2.708853238
High	Q06546	GA-binding protein alpha chain OS=Homo sapiens OX=960	2.701365217
High	P53990	IST1 homolog OS=Homo sapiens OX=9606 GN=IST1 PE=1	2.691649051
High	Q8WUF5	RelA-associated inhibitor OS=Homo sapiens OX=9606 GN=	2.685500772
High	P49207	60S ribosomal protein L34 OS=Homo sapiens OX=9606 GN	2.684239509
High	Q9C0C7	Activating molecule in BECN1-regulated autophagy protei	2.682145511
High	O96000	NADH dehydrogenase [ubiquinone] 1 beta subcomplex su	2.6712128
High	Q9NR12	PDZ and LIM domain protein 7 OS=Homo sapiens OX=960	2.653451441
High	Q01968	Inositol polyphosphate 5-phosphatase OCRL-1 OS=Homo	2.653255945
High	Q5SRD1	Putative mitochondrial import inner membrane transloca	2.634699251
High	Q96RF0	Sorting nexin-18 OS=Homo sapiens OX=9606 GN=SNX18 P	2.632271454
High	P00966	Argininosuccinate synthase OS=Homo sapiens OX=9606 G	2.619672763
High	P08621	U1 small nuclear ribonucleoprotein 70 kDa OS=Homo sap	2.602407566
High	Q9H7H0	Methyltransferase-like protein 17, mitochondrial OS=Hom	2.602059991
High	O95219	Sorting nexin-4 OS=Homo sapiens OX=9606 GN=SNX4 PE=	2.585360853
High	Q8N5A5	Zinc finger CCCH-type with G patch domain-containing pro	2.566070234
High	O60645	Exocyst complex component 3 OS=Homo sapiens OX=960	2.5551748
High	Q92609	TBC1 domain family member 5 OS=Homo sapiens OX=960	2.553307534
High	Q9Y314	Nitric oxide synthase-interacting protein OS=Homo sapien	2.549750892
High	Q6P2H3	Centrosomal protein of 85 kDa OS=Homo sapiens OX=960	2.541815564
High	Q5VZE5	N-alpha-acetyltransferase 35, NatC auxiliary subunit OS=H	2.527536103
High	Q9BY77	Polymerase delta-interacting protein 3 OS=Homo sapiens	2.526951195
High	Q9H4L7	SWI/SNF-related matrix-associated actin-dependent regul	2.520856752
High	Q9H9T3	Elongator complex protein 3 OS=Homo sapiens OX=9606	2.520280765
High	Q9Y467	Sal-like protein 2 OS=Homo sapiens OX=9606 GN=SALL2 P	2.519993057
High	P02786	Transferrin receptor protein 1 OS=Homo sapiens OX=9606	2.498803758
High	O95905	Protein ecdysoneless homolog OS=Homo sapiens OX=960	2.484523559
High	Q92599	Septin-8 OS=Homo sapiens OX=9606 GN=SEPT8 PE=1 SV=	2.48148606
High	Q9HD45	Transmembrane 9 superfamily member 3 OS=Homo sapie	2.466355021
High	Q9P253	Vacuolar protein sorting-associated protein 18 homolog O	2.463189134
High	O43318	Mitogen-activated protein kinase kinase kinase 7 OS=Hom	2.451242171
High	Q8IYQ7	Threonine synthase-like 1 OS=Homo sapiens OX=9606 GN	2.451119437
High	O15479	Melanoma-associated antigen B2 OS=Homo sapiens OX=9	2.438421632
High	Q14691	DNA replication complex GINS protein PSF1 OS=Homo sap	2.438064237
High	Q8IVD9	NudC domain-containing protein 3 OS=Homo sapiens OX=	2.434860848
High	Q9UNN5	FAS-associated factor 1 OS=Homo sapiens OX=9606 GN=F	2.413187731
High	P33981	Dual specificity protein kinase TTK OS=Homo sapiens OX=	2.412850502

High	Q9UI10	Translation initiation factor eIF-2B subunit delta OS=Hom	2.412176829
High	Q9NW64	Pre-mRNA-splicing factor RBM22 OS=Homo sapiens OX=9	2.409715596
High	P83881	60S ribosomal protein L36a OS=Homo sapiens OX=9606 G	2.405276054
High	Q15118	[Pyruvate dehydrogenase (acetyl-transferring)] kinase iso	2.402414498
High	Q9UNS2	COP9 signalosome complex subunit 3 OS=Homo sapiens C	2.401209493
High	Q8IWZ3	Ankyrin repeat and KH domain-containing protein 1 OS=H	2.398454746
High	Q9UBE0	SUMO-activating enzyme subunit 1 OS=Homo sapiens OX	2.394479477
High	A6NHR9	Structural maintenance of chromosomes flexible hinge dc	2.384681343
High	O60678	Protein arginine N-methyltransferase 3 OS=Homo sapiens	2.378408324
High	Q9NXE4	Sphingomyelin phosphodiesterase 4 OS=Homo sapiens O)	2.377682339
High	O75792	Ribonuclease H2 subunit A OS=Homo sapiens OX=9606 GI	2.37161107
High	Q8NEZ5	F-box only protein 22 OS=Homo sapiens OX=9606 GN=FB)	2.366834646
High	Q8NI27	THO complex subunit 2 OS=Homo sapiens OX=9606 GN=T	2.364616796
High	Q9NUL3	Double-stranded RNA-binding protein Staufen homolog 2	2.358300303
High	Q9NTJ5	Phosphatidylinositide phosphatase SAC1 OS=Homo sapier	2.354675998
High	Q9UHY7	Enolase-phosphatase E1 OS=Homo sapiens OX=9606 GN=	2.343422709
High	P98175	RNA-binding protein 10 OS=Homo sapiens OX=9606 GN=I	2.342657263
High	Q9BZV1	UBX domain-containing protein 6 OS=Homo sapiens OX=9	2.338944915
High	Q7Z2Y8	Interferon-induced very large GTPase 1 OS=Homo sapiens	2.332360294
High	Q9NRN7	L-aminoadipate-semialdehyde dehydrogenase-phosphopa	2.330962199
High	P62888	60S ribosomal protein L30 OS=Homo sapiens OX=9606 GI	2.32339783
High	Q9BTE7	DCN1-like protein 5 OS=Homo sapiens OX=9606 GN=DCU	2.321481621
High	Q9H773	dCTP pyrophosphatase 1 OS=Homo sapiens OX=9606 GN=	2.316772794
High	O60566	Mitotic checkpoint serine/threonine-protein kinase BUB1	2.315783205
High	Q9Y5Y2	Cytosolic Fe-S cluster assembly factor NUBP2 OS=Homo s	2.306360974
High	Q9UBK8	Methionine synthase reductase OS=Homo sapiens OX=96	2.290560426
High	Q7Z5K2	Wings apart-like protein homolog OS=Homo sapiens OX=9	2.288192771
High	P49257	Protein ERGIC-53 OS=Homo sapiens OX=9606 GN=LMAN1	2.287182
High	Q9H6R7	WD repeat and coiled-coil-containing protein OS=Homo s	2.286257522
High	P57772	Selenocysteine-specific elongation factor OS=Homo sapie	2.282163133
High	Q96EY7	Pentatricopeptide repeat domain-containing protein 3, m	2.274823699
High	O60333	Kinesin-like protein KIF1B OS=Homo sapiens OX=9606 GN	2.271727402
High	Q9HBI1	Beta-parvin OS=Homo sapiens OX=9606 GN=PARVB PE=1	2.26520017
High	Q86YR5	G-protein-signaling modulator 1 OS=Homo sapiens OX=96	2.255315937
High	Q9ULC4	Malignant T-cell-amplified sequence 1 OS=Homo sapiens	2.254769015
High	Q8WUM0	Nuclear pore complex protein Nup133 OS=Homo sapiens	2.239577517
High	P12081	Histidine--tRNA ligase, cytoplasmic OS=Homo sapiens OX=	2.239502125
High	O00743	Serine/threonine-protein phosphatase 6 catalytic subunit	2.229589868
High	Q8IV08	Phospholipase D3 OS=Homo sapiens OX=9606 GN=PLD3 I	2.213390527
High	Q8WVX9	Fatty acyl-CoA reductase 1 OS=Homo sapiens OX=9606 GI	2.208660296
High	P00156	Cytochrome b OS=Homo sapiens OX=9606 GN=MT-CYB P	2.205790884
High	O95551	Tyrosyl-DNA phosphodiesterase 2 OS=Homo sapiens OX=9	2.204050501
High	Q14527	Helicase-like transcription factor OS=Homo sapiens OX=9	2.195111255
High	Q8TCJ2	Dolichyl-diphosphooligosaccharide--protein glycosyltrans	2.138226703
High	Q9P0I2	ER membrane protein complex subunit 3 OS=Homo sapie	2.134126472
High	P43307	Translocon-associated protein subunit alpha OS=Homo sa	2.133949076
High	Q96J01	THO complex subunit 3 OS=Homo sapiens OX=9606 GN=T	2.132827249
High	Q8ND04	Protein SMG8 OS=Homo sapiens OX=9606 GN=SMG8 PE=	2.124302238
High	Q9NT62	Ubiquitin-like-conjugating enzyme ATG3 OS=Homo sapier	2.109187901
High	P43155	Carnitine O-acetyltransferase OS=Homo sapiens OX=9606	2.106959888

High	Q9UBV2	Protein sel-1 homolog 1 OS=Homo sapiens OX=9606 GN=	2.105517785
High	P09429	High mobility group protein B1 OS=Homo sapiens OX=960	2.103584024
High	P15153	Ras-related C3 botulinum toxin substrate 2 OS=Homo sap	2.090925599
High	O14974	Protein phosphatase 1 regulatory subunit 12A OS=Homo s	2.079302866
High	Q9NV88	Integrator complex subunit 9 OS=Homo sapiens OX=9606	2.077067043
High	Q15070	Mitochondrial inner membrane protein OXA1L OS=Homo	2.069356559
High	Q15061	WD repeat-containing protein 43 OS=Homo sapiens OX=9	2.067678468
High	Q8IWT6	Volume-regulated anion channel subunit LRRC8A OS=Hon	2.057545474
High	Q9UBQ5	Eukaryotic translation initiation factor 3 subunit K OS=Ho	2.051342568
High	Q8N3R9	MAGUK p55 subfamily member 5 OS=Homo sapiens OX=9	2.031376979
High	Q96DV4	39S ribosomal protein L38, mitochondrial OS=Homo sapie	2.029792641
High	P59046	NACHT, LRR and PYD domains-containing protein 12 OS=H	2.018951941
High	Q5T160	Probable arginine--tRNA ligase, mitochondrial OS=Homo s	2.01313873
Medium	P21399	Cytoplasmic aconitate hydratase OS=Homo sapiens OX=9	2.001566281
Medium	Q9Y3Q3	Transmembrane emp24 domain-containing protein 3 OS=	1.991825816
Medium	Q13557	Calcium/calmodulin-dependent protein kinase type II sub	1.981715692
Medium	Q9H8Y8	Golgi reassembly-stacking protein 2 OS=Homo sapiens OX	1.973058372
Medium	P67870	Casein kinase II subunit beta OS=Homo sapiens OX=9606	1.971835581
Medium	Q5VWZ2	Lysophospholipase-like protein 1 OS=Homo sapiens OX=9	1.971428747
Medium	P23634	Plasma membrane calcium-transporting ATPase 4 OS=Ho	1.966576245
Medium	Q9ULV4	Coronin-1C OS=Homo sapiens OX=9606 GN=CORO1C PE=	1.961379838
Medium	Q9NP92	39S ribosomal protein S30, mitochondrial OS=Homo sapie	1.956637722
Medium	Q15018	BRISC complex subunit Abraxas 2 OS=Homo sapiens OX=9	1.951169913
Medium	P62851	40S ribosomal protein S25 OS=Homo sapiens OX=9606 GN	1.950394387
Medium	Q9Y2R4	Probable ATP-dependent RNA helicase DDX52 OS=Homo	1.948847478
Medium	Q00535	Cyclin-dependent-like kinase 5 OS=Homo sapiens OX=960	1.932442649
Medium	Q15750	TGF-beta-activated kinase 1 and MAP3K7-binding protein	1.926648298
Medium	Q2VIQ3	Chromosome-associated kinesin KIF4B OS=Homo sapiens	1.924453039
Medium	P00367	Glutamate dehydrogenase 1, mitochondrial OS=Homo sap	1.912218582
Medium	Q70CQ1	Ubiquitin carboxyl-terminal hydrolase 49 OS=Homo sapie	1.904481958
Medium	Q9UKZ1	CCR4-NOT transcription complex subunit 11 OS=Homo sa	1.887060024
Medium	Q08379	Golgin subfamily A member 2 OS=Homo sapiens OX=9606	1.878112015
Medium	Q6ZNE9	RUN and FYVE domain-containing protein 4 OS=Homo sap	1.878112015
Medium	Q9Y5Q9	General transcription factor 3C polypeptide 3 OS=Homo s	1.876148359
Medium	O95071	E3 ubiquitin-protein ligase UBR5 OS=Homo sapiens OX=96	1.869023308
Medium	P49748	Very long-chain specific acyl-CoA dehydrogenase, mitoch	1.861381566
Medium	P82930	28S ribosomal protein S34, mitochondrial OS=Homo sapie	1.849857838
Medium	Q6NSI4	RPA-related protein RADX OS=Homo sapiens OX=9606 GN	1.849550591
Medium	Q15005	Signal peptidase complex subunit 2 OS=Homo sapiens OX	1.843148099
Medium	Q9NZL9	Methionine adenosyltransferase 2 subunit beta OS=Homo	1.840432807
Medium	Q02218	2-oxoglutarate dehydrogenase, mitochondrial OS=Homo s	1.837435593
Medium	Q15063	Periostin OS=Homo sapiens OX=9606 GN=POSTN PE=1 SV	1.835052627
Medium	Q8IZP0	Abl interactor 1 OS=Homo sapiens OX=9606 GN=ABI1 PE=	1.827105302
Medium	Q6PRD1	Probable G-protein coupled receptor 179 OS=Homo sapie	1.805208242
Medium	Q68CQ7	Glycosyltransferase 8 domain-containing protein 1 OS=Ho	1.797239313
Medium	P05026	Sodium/potassium-transporting ATPase subunit beta-1 OS	1.78968148
Medium	P36957	Dihydrolipoyllysine-residue succinyltransferase componen	1.788345599
Medium	Q5VWQ8	Disabled homolog 2-interacting protein OS=Homo sapiens	1.786216701
Medium	Q5JXC2	Migration and invasion-inhibitory protein OS=Homo sapie	1.77702355
Medium	P30038	Delta-1-pyrroline-5-carboxylate dehydrogenase, mitochon	1.766242637

Medium	Q96T37	RNA-binding protein 15 OS=Homo sapiens OX=9606 GN=I	1.744004273
Medium	Q9UH99	SUN domain-containing protein 2 OS=Homo sapiens OX=9	1.728855682
Medium	Q8WWK9	Cytoskeleton-associated protein 2 OS=Homo sapiens OX=	1.721475035
Medium	P37837	Transaldolase OS=Homo sapiens OX=9606 GN=TALDO1 PI	1.714217726
Medium	Q9NRC1	Suppressor of tumorigenicity 7 protein OS=Homo sapiens	1.708187313
Medium	P18887	DNA repair protein XRCC1 OS=Homo sapiens OX=9606 GN	1.695078838
Medium	O15357	Phosphatidylinositol 3,4,5-trisphosphate 5-phosphatase 2	1.693789492
Medium	O60563	Cyclin-T1 OS=Homo sapiens OX=9606 GN=CCNT1 PE=1 SV	1.683191248
Medium	P20042	Eukaryotic translation initiation factor 2 subunit 2 OS=Hor	1.679230772
Medium	Q5PRF9	Protein Smaug homolog 2 OS=Homo sapiens OX=9606 GN	1.676335464
Medium	P11387	DNA topoisomerase 1 OS=Homo sapiens OX=9606 GN=TC	1.671620397
Medium	Q6P1M0	Long-chain fatty acid transport protein 4 OS=Homo sapier	1.660946264
Medium	Q9BV86	N-terminal Xaa-Pro-Lys N-methyltransferase 1 OS=Homo	1.655804284
Medium	P07203	Glutathione peroxidase 1 OS=Homo sapiens OX=9606 GN	1.647817482
Medium	O14818	Proteasome subunit alpha type-7 OS=Homo sapiens OX=9	1.635073966
Medium	Q9NTI5	Sister chromatid cohesion protein PDS5 homolog B OS=H	1.634137785
Medium	Q8TEA1	Putative methyltransferase NSUN6 OS=Homo sapiens OX=	1.632085261
Medium	P11274	Breakpoint cluster region protein OS=Homo sapiens OX=9	1.62948691
Medium	Q9UPY3	Endoribonuclease Dicer OS=Homo sapiens OX=9606 GN=I	1.627087997
Medium	Q08170	Serine/arginine-rich splicing factor 4 OS=Homo sapiens O)	1.624153564
Medium	Q8IX18	Probable ATP-dependent RNA helicase DHX40 OS=Homo :	1.613322716
Medium	P82675	28S ribosomal protein S5, mitochondrial OS=Homo sapier	1.611898798
Medium	P09960	Leukotriene A-4 hydrolase OS=Homo sapiens OX=9606 GI	1.608183076
Medium	Q96ER9	Coiled-coil domain-containing protein 51 OS=Homo sapie	1.602581458
Medium	Q13085	Acetyl-CoA carboxylase 1 OS=Homo sapiens OX=9606 GN	1.598771833
Medium	Q9UIU6	Homeobox protein SIX4 OS=Homo sapiens OX=9606 GN=!	1.596022036
Medium	Q96BM9	ADP-ribosylation factor-like protein 8A OS=Homo sapiens	1.595166283
Medium	O60879	Protein diaphanous homolog 2 OS=Homo sapiens OX=960	1.586197483
Medium	Q9Y570	Protein phosphatase methylesterase 1 OS=Homo sapiens	1.581201709
Medium	Q969Y2	tRNA modification GTPase GTPBP3, mitochondrial OS=Ho	1.574139855
Medium	Q9UI12	V-type proton ATPase subunit H OS=Homo sapiens OX=96	1.560193789
Medium	Q96HU8	GTP-binding protein Di-Ras2 OS=Homo sapiens OX=9606 (	1.559563234
Medium	Q6P2I3	Fumarylacetoacetate hydrolase domain-containing protei	1.558304864
Medium	Q96CT7	Coiled-coil domain-containing protein 124 OS=Homo sapi	1.552686891
Medium	Q86WI3	Protein NLRC5 OS=Homo sapiens OX=9606 GN=NLRC5 PE	1.550213153
Medium	P11233	Ras-related protein Ral-A OS=Homo sapiens OX=9606 GN	1.542875374
Medium	Q9BY44	Eukaryotic translation initiation factor 2A OS=Homo sapie	1.541815564
Medium	Q5T5X7	BEN domain-containing protein 3 OS=Homo sapiens OX=9	1.535361441
Medium	Q92997	Segment polarity protein dishevelled homolog DVL-3 OS=	1.532096053
Medium	Q3B7T1	Erythroid differentiation-related factor 1 OS=Homo sapier	1.521433504
Medium	Q8TDN6	Ribosome biogenesis protein BRX1 homolog OS=Homo sa	1.520280765
Medium	P23284	Peptidyl-prolyl cis-trans isomerase B OS=Homo sapiens O)	1.515557792
Medium	P25788	Proteasome subunit alpha type-3 OS=Homo sapiens OX=9	1.500863005
Medium	Q86YT6	E3 ubiquitin-protein ligase MIB1 OS=Homo sapiens OX=96	1.496754229
Medium	O60343	TBC1 domain family member 4 OS=Homo sapiens OX=960	1.467500414
Medium	Q7Z4H7	HAUS augmin-like complex subunit 6 OS=Homo sapiens O	1.465719995
Medium	Q99490	Arf-GAP with GTPase, ANK repeat and PH domain-contain	1.464073259
Medium	O75718	Cartilage-associated protein OS=Homo sapiens OX=9606 (	1.462558717
Medium	Q9UDR5	Alpha-aminoadipic semialdehyde synthase, mitochondrial	1.453333975
Medium	Q13242	Serine/arginine-rich splicing factor 9 OS=Homo sapiens O)	1.444059562

Medium	P07339	Cathepsin D OS=Homo sapiens OX=9606 GN=CTSD PE=1 S	1.443335738
Medium	Q9Y6X3	MAU2 chromatid cohesion factor homolog OS=Homo sap	1.443094731
Medium	P08579	U2 small nuclear ribonucleoprotein B'' OS=Homo sapiens	1.442132038
Medium	Q9BW60	Elongation of very long chain fatty acids protein 1 OS=Hor	1.436637591
Medium	Q9UBQ7	Glyoxylate reductase/hydroxypyruvate reductase OS=Hor	1.429924295
Medium	Q9NYB0	Telomeric repeat-binding factor 2-interacting protein 1 OS	1.429223631
Medium	Q9BZ23	Pantothenate kinase 2, mitochondrial OS=Homo sapiens (	1.422623108
Medium	Q9Y5A7	NEDD8 ultimate buster 1 OS=Homo sapiens OX=9606 GN=	1.410273744
Medium	Q9BXW6	Oxysterol-binding protein-related protein 1 OS=Homo sap	1.386475297
Medium	O95801	Tetratricopeptide repeat protein 4 OS=Homo sapiens OX=	1.379968105
Medium	O75165	DnaJ homolog subfamily C member 13 OS=Homo sapiens	1.379447555
Medium	Q9HBE4	Interleukin-21 OS=Homo sapiens OX=9606 GN=IL21 PE=1	1.370998381
Medium	Q99797	Mitochondrial intermediate peptidase OS=Homo sapiens	1.370386555
Medium	P15927	Replication protein A 32 kDa subunit OS=Homo sapiens O	1.367947833
Medium	Q96GC9	Vacuole membrane protein 1 OS=Homo sapiens OX=9606	1.35349825
Medium	Q53TS8	C2 calcium-dependent domain-containing protein 6 OS=H	1.34930402
Medium	O15084	Serine/threonine-protein phosphatase 6 regulatory ankyr	1.348431261
Medium	Q99447	Ethanolamine-phosphate cytidylyltransferase OS=Homo s	1.345630909
Medium	Q9NWX5	Ankyrin repeat and SOCS box protein 6 OS=Homo sapiens	1.336770365
Medium	Q96MR6	Cilia- and flagella-associated protein 57 OS=Homo sapiens	1.333201316
Medium	Q5JVF3	PCI domain-containing protein 2 OS=Homo sapiens OX=96	1.328734567
Medium	Q99714	3-hydroxyacyl-CoA dehydrogenase type-2 OS=Homo sapie	1.318397001
Medium	P29474	Nitric oxide synthase, endothelial OS=Homo sapiens OX=9	1.315064917
Medium	Q2TAY7	WD40 repeat-containing protein SMU1 OS=Homo sapiens	1.298000525
Medium	P46087	Probable 28S rRNA (cytosine(4447)-C(5))-methyltransfera	1.288952396
Medium	Q9Y4C1	Lysine-specific demethylase 3A OS=Homo sapiens OX=960	1.288361462
Medium	Q99426	Tubulin-folding cofactor B OS=Homo sapiens OX=9606 GN	1.2817475
Medium	Q9UHQ9	NADH-cytochrome b5 reductase 1 OS=Homo sapiens OX=	1.278931698
Medium	Q969X6	U3 small nucleolar RNA-associated protein 4 homolog OS=	1.274496731
Medium	Q86UE4	Protein LYRIC OS=Homo sapiens OX=9606 GN=MTDH PE=	1.27205229
Medium	Q7RTP6	[F-actin]-monooxygenase MICAL3 OS=Homo sapiens OX=9	1.264241463
Medium	Q9BYD2	39S ribosomal protein L9, mitochondrial OS=Homo sapien	1.262568799
Medium	P15531	Nucleoside diphosphate kinase A OS=Homo sapiens OX=9	1.262409834
Medium	P36268	Inactive glutathione hydrolase 2 OS=Homo sapiens OX=96	1.251734427
Medium	O14776	Transcription elongation regulator 1 OS=Homo sapiens OX	1.251424383
Medium	Q92925	SWI/SNF-related matrix-associated actin-dependent regul	1.250186415
Medium	Q9NRY5	Protein FAM114A2 OS=Homo sapiens OX=9606 GN=FAM1	1.24910608
Medium	Q5PSV4	Breast cancer metastasis-suppressor 1-like protein OS=Ho	1.245498771
Medium	Q7RTY1	Monocarboxylate transporter 9 OS=Homo sapiens OX=96	1.233884717
Medium	Q6PML9	Zinc transporter 9 OS=Homo sapiens OX=9606 GN=SLC30	1.218316415
Medium	Q96BV0	Zinc finger protein 775 OS=Homo sapiens OX=9606 GN=Z	1.217312432
Medium	Q9Y5I4	Protocadherin alpha-C2 OS=Homo sapiens OX=9606 GN=I	1.210771943
Medium	Q13217	DnaJ homolog subfamily C member 3 OS=Homo sapiens O	1.205093893
Medium	P48681	Nestin OS=Homo sapiens OX=9606 GN=NES PE=1 SV=2	1.203772686
Medium	Q5T0B9	Zinc finger protein 362 OS=Homo sapiens OX=9606 GN=Z	1.198664904
Medium	Q93050	V-type proton ATPase 116 kDa subunit a isoform 1 OS=Ho	1.197021145
Medium	O43309	Zinc finger and SCAN domain-containing protein 12 OS=H	1.196474604
Medium	Q9NZP5	Olfactory receptor 5AC2 OS=Homo sapiens OX=9606 GN=	1.186152246
Medium	Q96M15	Putative uncharacterized protein IGF2BP2-AS1 OS=Homo	1.180192935
Medium	Q9NU22	Midasin OS=Homo sapiens OX=9606 GN=MDN1 PE=1 SV=	1.174249419

Medium	Q9H6R4	Nucleolar protein 6 OS=Homo sapiens OX=9606 GN=NOL6	1.172114017
Medium	Q9HCS7	Pre-mRNA-splicing factor SYF1 OS=Homo sapiens OX=960	1.16615195
Medium	P49757	Protein numb homolog OS=Homo sapiens OX=9606 GN=N	1.162285332
Medium	Q9Y383	Putative RNA-binding protein Luc7-like 2 OS=Homo sapien	1.156829535
Medium	P35237	Serpin B6 OS=Homo sapiens OX=9606 GN=SERPINB6 PE=1	1.145815456
Medium	P09543	2',3'-cyclic-nucleotide 3'-phosphodiesterase OS=Homo sa	1.140741583
Medium	Q9UP83	Conserved oligomeric Golgi complex subunit 5 OS=Homo	1.133476374
Medium	Q92542	Nicastrin OS=Homo sapiens OX=9606 GN=NCSTN PE=1 SV	1.133417323
Medium	Q8TF72	Protein Shroom3 OS=Homo sapiens OX=9606 GN=SHROO	1.129011186
Medium	P02788	Lactotransferrin OS=Homo sapiens OX=9606 GN=LTF PE=1	1.122571059
Medium	P53597	Succinate--CoA ligase [ADP/GDP-forming] subunit alpha, r	1.115204636
Medium	Q13123	Protein Red OS=Homo sapiens OX=9606 GN=IK PE=1 SV=3	1.111202933
Medium	Q86VV8	Rotatin OS=Homo sapiens OX=9606 GN=RTTN PE=1 SV=3	1.107849722
Medium	Q7L9L4	MOB kinase activator 1B OS=Homo sapiens OX=9606 GN=	1.095500845
Medium	Q9NR77	Peroxisomal membrane protein 2 OS=Homo sapiens OX=9	1.08396739
Medium	P49916	DNA ligase 3 OS=Homo sapiens OX=9606 GN=LIG3 PE=1 S	1.07494588
Medium	Q9NPL8	Complex I assembly factor TIMMDC1, mitochondrial OS=H	1.074327006
Medium	Q13421	Mesothelin OS=Homo sapiens OX=9606 GN=MSLN PE=1 S	1.061980903
Medium	Q8NCM8	Cytoplasmic dynein 2 heavy chain 1 OS=Homo sapiens OX	1.057347188
Medium	Q8WXI7	Mucin-16 OS=Homo sapiens OX=9606 GN=MUC16 PE=1 S	1.054137676
Medium	P49354	Protein farnesyltransferase/geranylgeranyltransferase typ	1.048079926
Medium	P51572	B-cell receptor-associated protein 31 OS=Homo sapiens C	1.045660991
Medium	Q86Z14	Beta-klotho OS=Homo sapiens OX=9606 GN=KLB PE=1 SV	1.041770686
Low	O75943	Cell cycle checkpoint protein RAD17 OS=Homo sapiens O)	1.035410413
Low	Q9NPF4	Probable tRNA N6-adenosine threonylcarbamoyltransfera	1.03498655
Low	Q6PIJ6	F-box only protein 38 OS=Homo sapiens OX=9606 GN=FB)	1.026595026
Low	Q9H0L4	Cleavage stimulation factor subunit 2 tau variant OS=Hom	1.021773818
Low	Q8WUA7	TBC1 domain family member 22A OS=Homo sapiens OX=9	1.017547849
Low	P35610	Sterol O-acyltransferase 1 OS=Homo sapiens OX=9606 GN	1.004847623
Low	Q9H9Y2	Ribosome production factor 1 OS=Homo sapiens OX=9606	1.001130636
Low	Q66K74	Microtubule-associated protein 1S OS=Homo sapiens OX=	1.001087096
Low	P01024	Complement C3 OS=Homo sapiens OX=9606 GN=C3 PE=1	1.001000028
Low	Q9C0D3	Protein zyg-11 homolog B OS=Homo sapiens OX=9606 GN	0.994819487
Low	P62910	60S ribosomal protein L32 OS=Homo sapiens OX=9606 GN	0.973875483
Low	A2A2Z9	Ankyrin repeat domain-containing protein 18B OS=Homo	0.957030927
Low	Q9UPS8	Ankyrin repeat domain-containing protein 26 OS=Homo s	0.949620244
Low	Q86YN1	Dolichyldiphosphatase 1 OS=Homo sapiens OX=9606 GN=	0.946537395
Low	Q5TZA2	Rootletin OS=Homo sapiens OX=9606 GN=CROCC PE=1 SV	0.946153573
Low	Q96GD0	Pyridoxal phosphate phosphatase OS=Homo sapiens OX=9	0.913996294
Low	Q9NYK5	39S ribosomal protein L39, mitochondrial OS=Homo sapie	0.908333042
Low	Q9H000	Probable E3 ubiquitin-protein ligase makorin-2 OS=Homo	0.901356274
Low	P67812	Signal peptidase complex catalytic subunit SEC11A OS=Ho	0.900664722
