# Supplementary figures and images for: SARS-COV-2 protein NSP9 promotes cytokine production by targeting TBK1
Source: Front Immunol. 2023 Oct 2;14:1211816. doi: 10.3389/fimmu.2023.1211816 (PMC10580797; doi:10.3389/fimmu.2023.1211816)

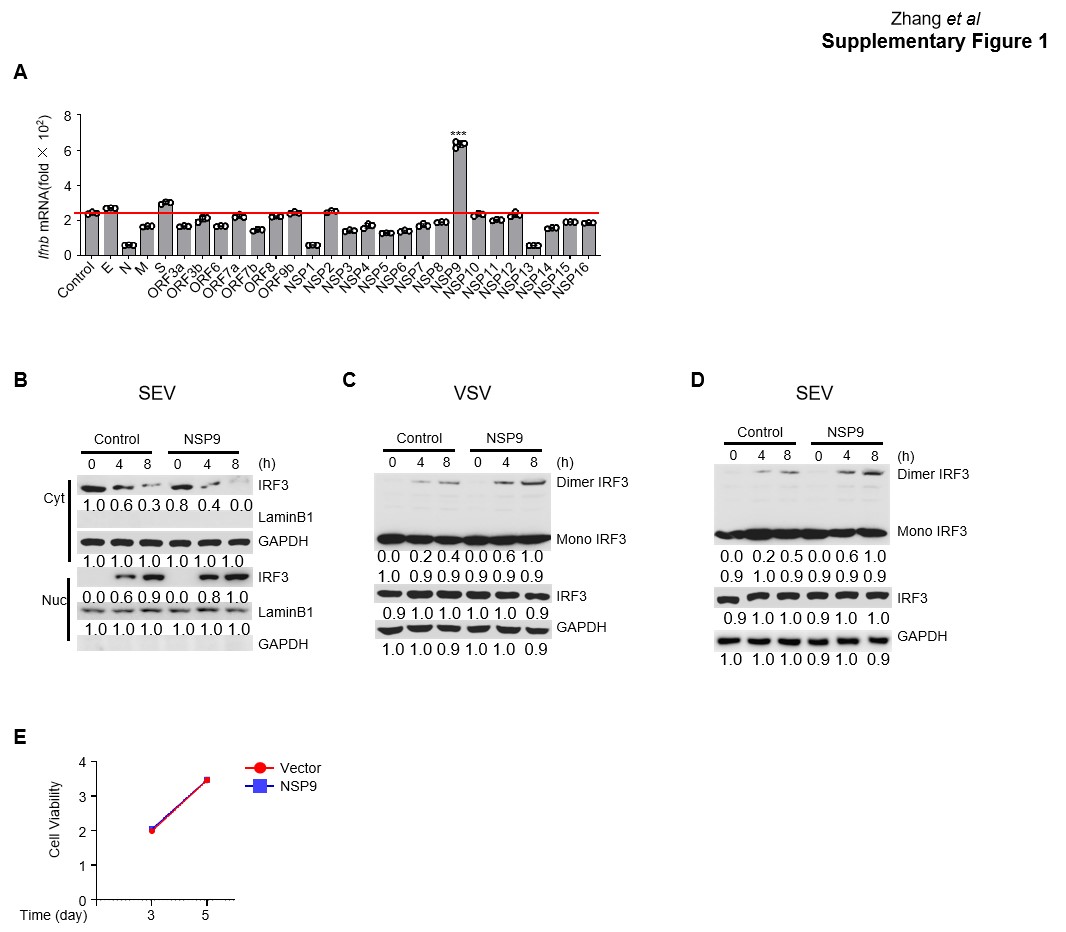

Supplement: Supplementary file 5 [file Image_1.jpeg]

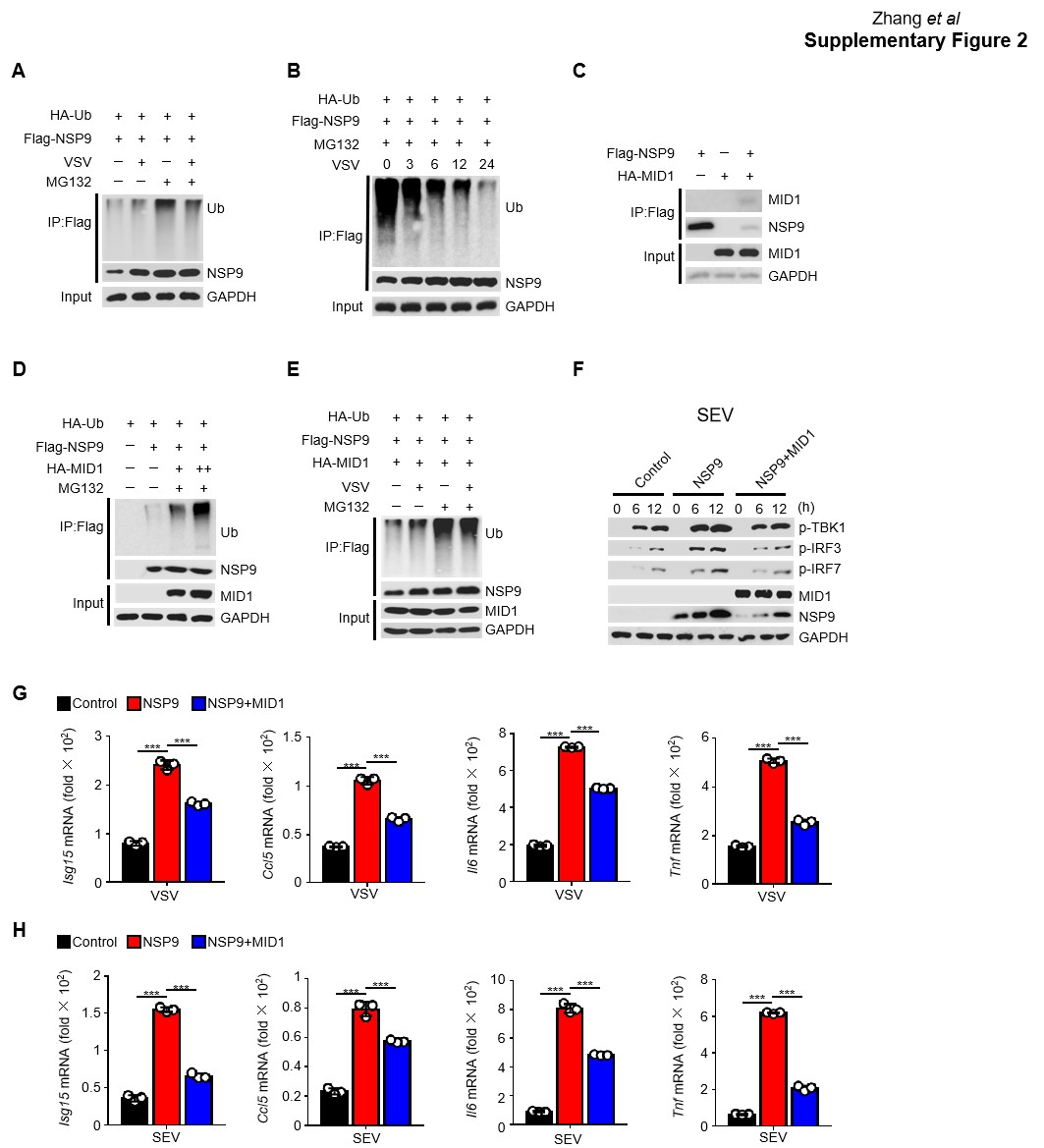

Supplement: Supplementary file 6 [file Image_2.jpeg]

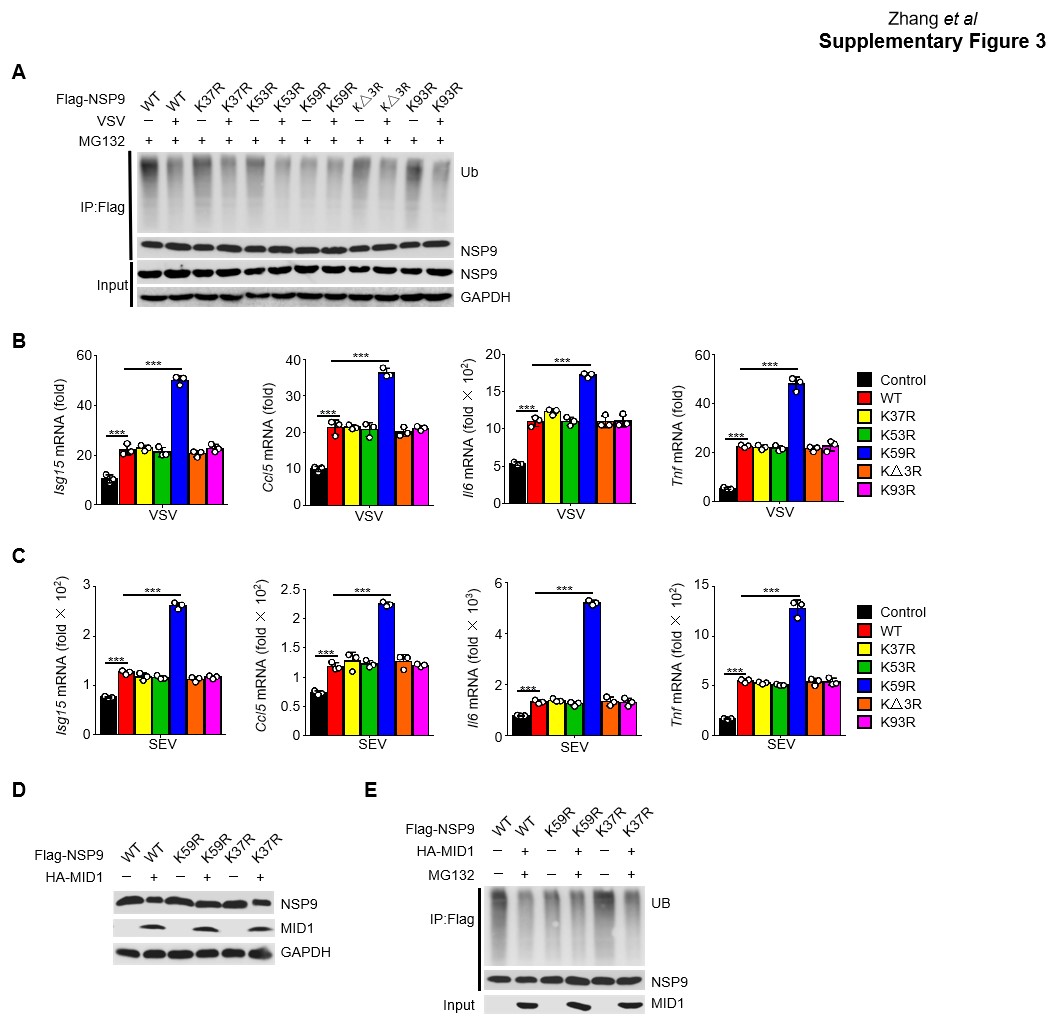

Supplement: Supplementary file 7 [file Image_3.jpeg]

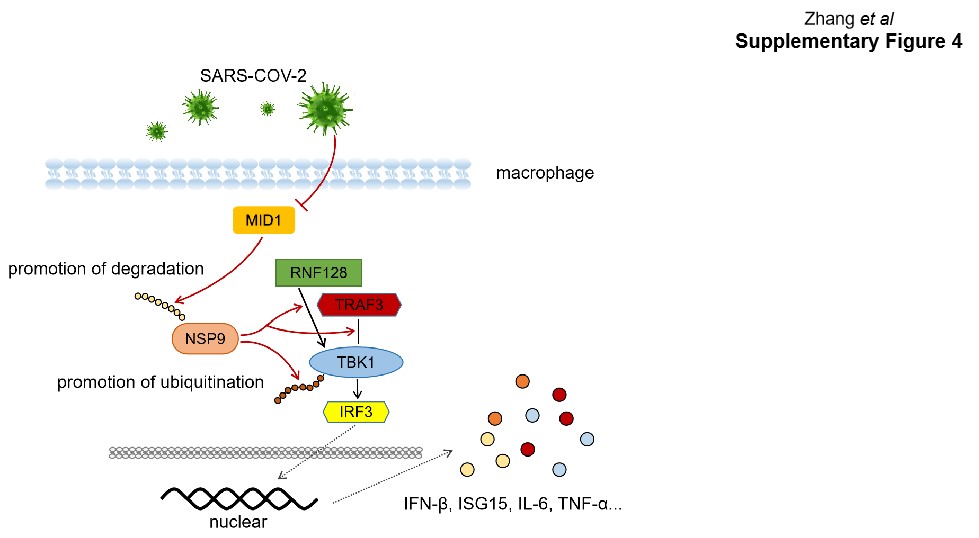

Supplement: Supplementary file 8 [file Image_4.jpeg]
